# Enhancing the Air Stability of Dimolybdenum Paddlewheel Complexes: Redox Tuning through Fluorine Substituents

**DOI:** 10.1021/acs.inorgchem.2c02746

**Published:** 2022-11-18

**Authors:** Imogen A. Z. Squire, Christopher A. Goult, Benedict C. Thompson, Elias Alexopoulos, Adrian C. Whitwood, Theo F. N. Tanner, Luke A. Wilkinson

**Affiliations:** Department of Chemistry, University of York, Heslington, York YO10 5DD, U.K.

## Abstract

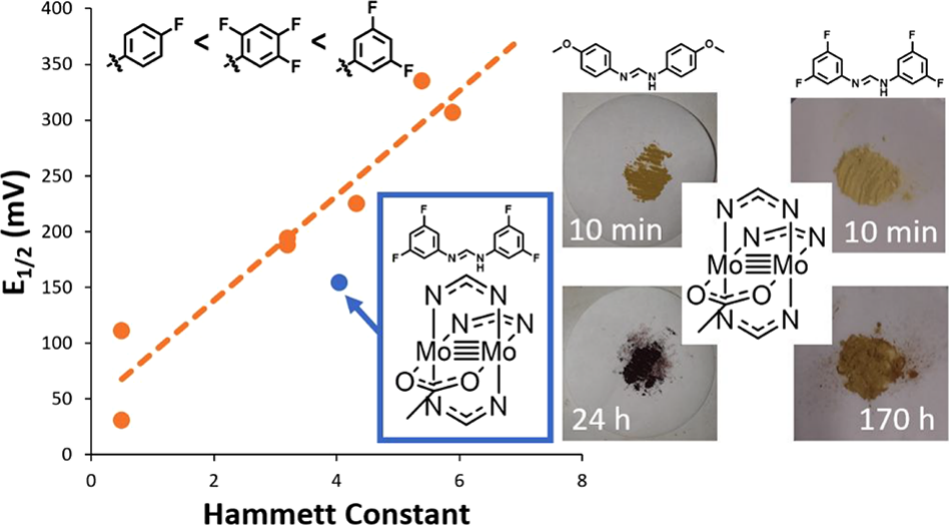

The optical and electrochemical properties of quadruply bonded dimolybdenum paddlewheel complexes (Mo_2_PWCs) make them ideal candidates for incorporation into functional materials or devices, but one of the greatest bottlenecks for this is their poor stability toward atmospheric oxygen. By tuning the potential at which the Mo_2_ core is oxidized, it was possible to increase the tolerance of Mo_2_PWCs to air. A series of homoleptic Mo_2_PWCs bearing fluorinated formamidinate ligands have been synthesized and their electrochemical properties studied. The oxidation potential of the complexes was tuned in a predictable fashion by controlling the positions of the fluorine substituents on the ligands, as guided by a Hammett relationship. Studies into the air stability of the resulting complexes by multinuclear NMR spectroscopy show an increased tolerance to atmospheric oxygen with increasingly electron-withdrawing ligands. The heteroleptic complex Mo_2_(D^F^ArF)_3_(OAc) [where D^F^ArF = 3,5-(difluorophenyl)formamidinate] shows remarkable tolerance to oxygen in the solid state and in chloroform solutions. Through the employment of easily accessible ligands, the stability of the Mo_2_ core toward oxygen has been enhanced, thereby making Mo_2_PWCs with electron-withdrawing ligands more attractive candidates for the development of functional materials.

## Abstract 2

Herein the oxidation potential of quadruply bonded dimolybdenum paddlewheel complexes was tuned using fluorinated formamidinate ligands. Using this strategy, the oxidation potential was increased such that reaction of the Mo_2_ core with oxygen became less favorable. The stability of even heteroleptic complexes bearing kinetically labile ligands was demonstrated by NMR spectroscopy in “oxygenated” solvents.

## Introduction

**Figure 1:**
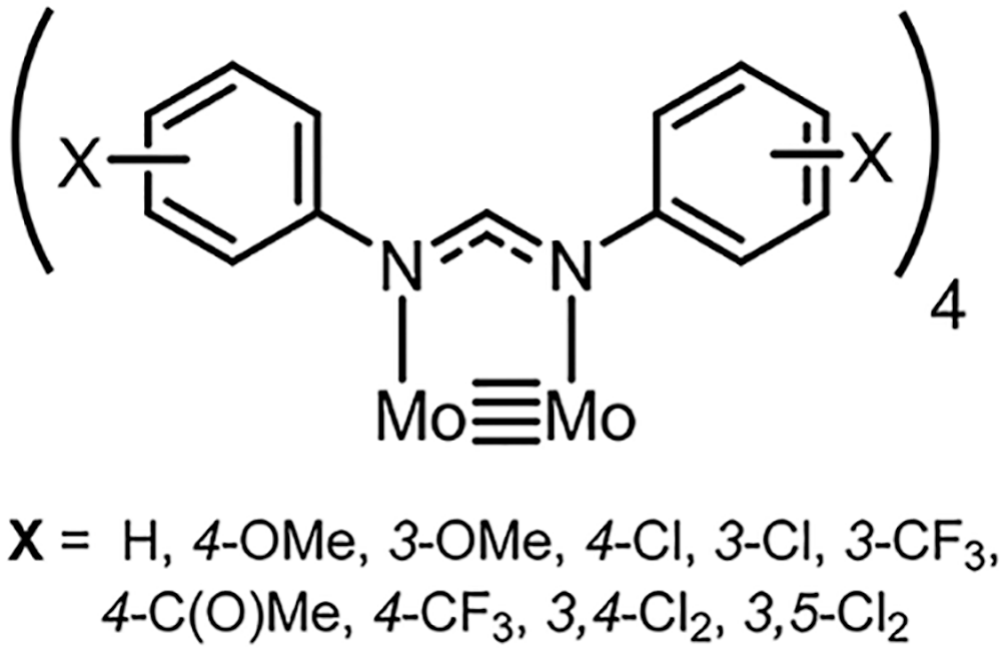
General structure of a homoleptic dimolybdenum tetraformamidinate complex [Mo_2_(NN)_4_].

Quadruply-bonded dimolybdenum paddlewheel complexes (Mo_2_PWCs) have enormous potential as components for functional materials. Their 3D structure allows the construction of large molecular arrays through combination with a broad scope of bridging ligands.^1−3^ They are redox-active, undergoing one-electron oxidation processes, often at easily accessible potentials. Additionally, the strong interaction between the Mo_2_-δ and π systems of equatorial chelating ligands [e.g., carboxylates (^−^O_2_CR) and formamidinates (^−^NN; Figure 1)] is ideal for facilitating and studying electron transfer in mixed-valence architectures^4−8^ and in photoexcited states.^9,10^ Consequently, Mo_2_PWCs are starting to find application in solar energy conversion,^11,12^ molecular electronics,^13^ and catalysis.^14,15^ However, there is one important caveat. Mo_2_PWCs are air-sensitive,^16^ reacting readily with atmospheric oxygen (O_2_), which could be seen as problematic for their translation into the aforementioned technologies. Therefore, a family of air-tolerant dimolybdenum compounds that could be further functionalized toward an intended application is highly desirable. One of the most stable Mo_2_PWCs is Mo_2_(OAc)_4_, which in its crystalline state is stable under an ambient atmosphere for around 1 week before visible discoloration occurs. The origin of this increased air stability stems from intermolecular Mo–O axial interactions that propagate through the material and hinder the approach of oxygen.^17^ Thus, using either Lewis basic or sterically bulky ligands to protect the axial position of the paddlewheel complex could be a viable method for enhancing the air stability.

An alternative approach toward increasing O_2_ tolerance, and the focus of this study, is to increase the oxidation potential of the metallic core such that the reaction with O_2_ becomes thermodynamically unfavorable.^18^ Tuning the oxidation potential of paddlewheel complexes can be achieved primarily by systematic variation of the ancillary ligands. In the homologous series Mo_2_(D_2_C-C≡C-Ph)_4_, where the donor atoms (D) were NN, ON, NS, and OO, the oxidation potential of the Mo_2_PWCs in *N*,*N*-dimethylformamide solutions could be tuned between −0.322 V (NN donor) and +0.282 V (OO donor), a range of 0.6 V.^19^ The hpp ligand (where Hhpp = 1,3,4,6,7,8- hexahydro-2*H*-pyrimido[1,2-*a*]pyrimidine) is a strong Lewis base and has been used to stabilize the W_2_^6+^ core in the most easily ionizable complexes known.^20^ Recently, the hpp ligand has been employed alongside formamidinate ligands to tune the Mo^4+/5+^_2_ couple from −0.381 V in Mo_2_(DAniF)_4_ [DAniF^–^ = di-4-anisylformamidinate] to potentials as low as −1.795 V versus ferrocene/ferrocenium (Fc/Fc^+^) in Mo_2_(hpp)_4_. Remote substituents on the ligands can also influence the properties of the dimetal core. This was illustrated as a linear free-energy relationship between the Hammett constant of the ligand substituents and the oxidation potential of the Mo_2_ core in a family of homoleptic formamidinate complexes, Mo_2_(NN)_4_ [where *E*_1/2_(*p*-OMe) = 0.244 V through to *E*_1/2_(*p*-CF_3_) = 0.795 V vs Ag/AgCl; Figure 1].^21^ Interestingly, the UV/visible spectrum (in CH_2_Cl_2_) of species with the strongest electron-withdrawing substituents (3,5-Cl_2_, 2σ_*m*_ = 0.74) remained unchanged, even after being exposed to air for 1 week.^22^ The same free-energy relationship was also observed between remote substituents on the ligands and the extent of charge stabilization (Δ*E*_1/2_) in mixed-valence dimers, of the form [Mo_2_(NN)_3_]_2_(μ-O_2_CC_6_H_4_CO_2_)^+^.^23^ The electronic properties of Ru_2_PWCs can also be varied with fluorinated ancillary ligands, which has been shown on multiple occasions.^24−27^ In one particular example, fluorinated and chlorinated benzoates were employed to achieve a remarkable fine-tuning of the oxidation potential of Ru^II,II^_2_PWCs between −0.039 and 0.36 V vs Fc/Fc^+^ in tetrahydrofuran (THF).^28^ Indeed, the authors also noted that those species with *E*_1/2_ > 0.3 V showed increased stability to O_2_.

Substituted formamidinates and benzoates have both been shown to effectively tune the properties of paddlewheel complexes^29,30^ and clusters.^31,32^ However, there has not yet been a systematic study employing fluorinated formamidinates to control the properties of Mo_2_PWCs. Fluorine is the ideal substituent to tune Mo_2_PWCs for several reasons. First, it is the most electronegative element with a considerable electron-withdrawing effect (σ_p_ = 0.062; σ_m_ = 0.337). Other functionalities such as cyano (σ_m_ = 0.56) or nitro (σ_m_ = 0.71) groups may exhibit stronger electron-withdrawing effects, but they are also potentially reactive, which may limit further application of the complexes. In contrast, the C–F bond is exceptionally strong, with a bond dissociation energy of 127.2 ± 0.7 kcal mol^–1^ in fluorobenzene (C–H in benzene is 112.9 ± 0.6 kcal mol^–1^), meaning that any subsequent complexes are unlikely to undergo deleterious side reactions at the ligand periphery.^33^ Second, the fluorine atom is small, with an atomic radius (vdW = van der Waals) only slightly larger than that of hydrogen (*r*_vdW_: F = 1.47 Å; H = 1.20 Å). Thus, the substitution of protons with fluorine atoms will have the minimal steric influence on the overall structures, unlike chloro analogues, which are significantly larger (*r*_vdW_ = 1.75 Å).^33^ In one report, a family of homoleptic dichromium tetraformamidinates with various fluorine-substitution patterns were studied, and it was found that the fluorination pattern had no influence on the Cr–Cr bond length of the complexes.^30^ Finally, the ^19^F nucleus is NMR-active with 100% natural abundance *I* = ^1^/_2_ and so provides a useful quantitative probe for NMR spectroscopic characterization or a further study of molecular dynamics. We envision that Mo_2_PWCs bearing kinetically inert^34^ formamidinate ligands with carefully selected fluorination patterns would represent a powerful approach toward achieving Mo_2_PWCs with enhanced air stability and therefore have the potential to be translated into functional materials. Using Hammett constants (σ) as a guide, we demonstrate that we can predictably tune the electronic properties (specifically the oxidation potential) of Mo_2_PWCs by varying the positions and quantity of fluorine atoms on the formamidinate ligands. We also employ NMR spectroscopy as a tool to follow decomposition of the complexes and demonstrate an increased tolerance to atmospheric oxygen with increasing σ. We also demonstrate that this strategy can be applied to heteroleptic complexes [Mo_2_(NN)_3_(OAc)], which are key synthons for accessing functional materials.

## Results and Discussion

### Synthesis

#### Ligand Design, Synthesis, and Characterization

**Figure 2:**
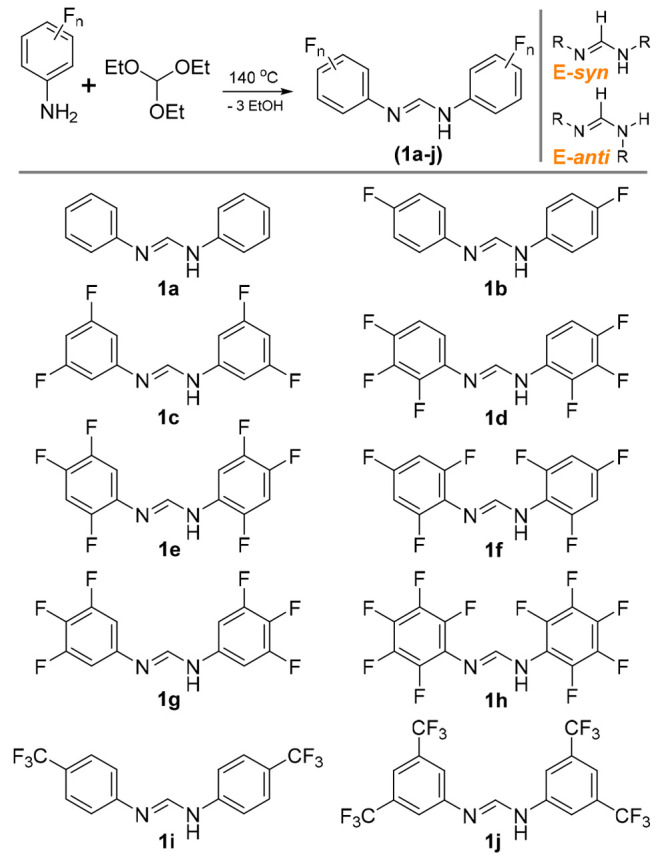
General synthetic pathway to formamidines and the series of ligands (**1a**–**1j**) featured in this paper.

In the chemistry of Mo_2_PWCs, carboxylate ligands are known to be kinetically labile (particularly if they bear strong electron-withdrawing groups),^35,36^ whereas formamidinate ligands are more persistent because of their increased Lewis basicity.^34^ Therefore, formamidinates are arguably more desirable ligand choices in the pursuit of robust functional materials. With that in mind, a family of formamidine ligands with different fluorine-substitution patterns were synthesized. All formamidine ligands described herein, including novel compounds **1d** and **1e**, can be synthesized following a well-established condensation reaction between the corresponding aniline and triethylorthoformate (Figure 2),^37^ generating off-white microcrystalline solids in yields varying from 50 to 80%. Each ligand has been characterized by ^1^H and ^19^F NMR spectroscopy in acetone-*d*_6_, electrospray ionization mass spectrometry (ESI MS), and elemental analysis, and in the case of **1c**–**1f**, single-crystal X-ray structures have been obtained and are displayed in the Supporting Information (SI). Single crystals were obtained from either recrystallization of the compound in toluene/hexane (2:1) or by vapor diffusion of hexane into a toluene solution. Compounds **1c**–**1e** all crystallize in an *E*-syn conformation where the aryl groups point away from the NCN bridge.^37^ However, **1f** buckles considerably and adopts the *E*-anti configuration, presumably to minimize any steric interaction between the *o*-fluorine atoms on the rings, although the *E*-syn configuration is still observed in the reported crystal structure of **1h**.^38^ Notably, for compounds **1c**–**1f**, we observe intermolecular hydrogen bonding in the packing of the single-crystal solid-state structure.

**Scheme 1:**
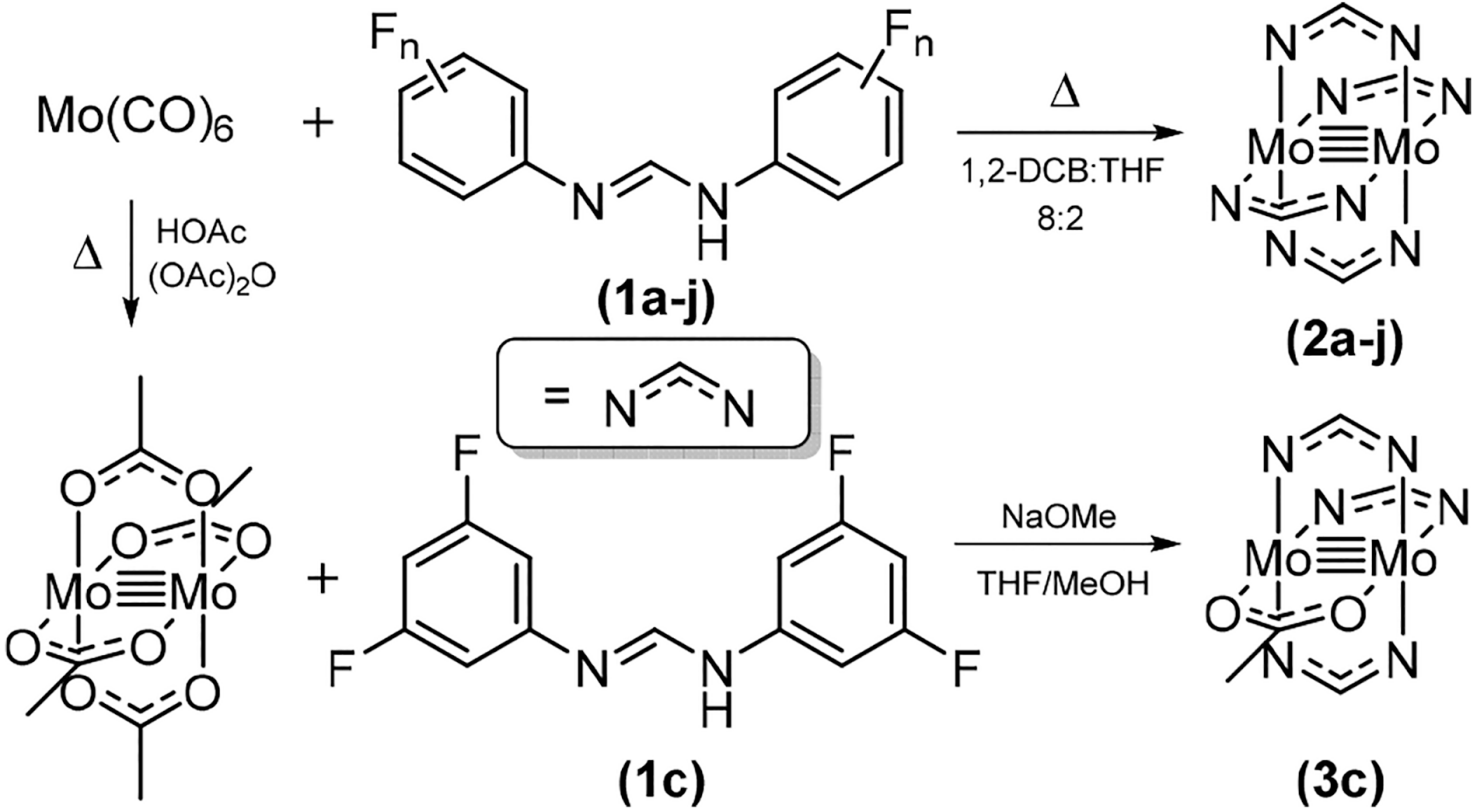
Synthetic Pathway to Dimolybdenum Tetraformamidinates (**2a**–**2j**) and the Heteroleptic Complex **3c**

The synthesis of homoleptic paddlewheel complexes of the form Mo_2_(NN)_4_ can be achieved either by (i) reacting the free ligand (**1x**) with Mo(CO)_6_ at ca. 160 °C or (ii) by reacting anionic ligand salts (i.e., **Li-1x**) with Mo_2_(OAc)_4_ at room temperature.^22^ We exclusively employ route (i) as described in Scheme 1, and although the yields appear low (9–30%), they are comparable to those from route (ii) when the synthesis of Mo_2_(OAc)_4_ from Mo(CO)_6_ is factored in. The heteroleptic complex **3c** was synthesized by reacting Mo_2_(OAc)_4_ with exactly 3 equiv of **1c** in THF and exactly 3 equiv of NaOMe in MeOH.^39^ Deviation from exact quantities will generate intractable mixtures of **3c** with either the tetraformamidinate (**2c**) or bisformamidinate complexes. All compounds present as canary-yellow solids with varying degrees of solubility in organic solvents, although THF will dissolve all but **2h**. In general, matrix-assisted laser desorption/ionization time-of-flight (MALDI-TOF) mass spectrometry (MS) showed a molecular ion ([M]^+^) for each compound at the expected *m*/*z* values, and the ^1^H and ^19^F NMR spectra displayed the resonances expected for each species. However, in most of the homoleptic complexes, the MALDI-TOF MS spectrum displayed an additional feature with an *m*/*z* value 88 units higher than the molecular-ion peak, which can be attributed to a Lewis adduct of some kind, where the Lewis base is coordinating to the axial site of the Mo_2_ unit. This adduct signal appears to be absent from the MALDI-TOF MS spectrum of **2h**, which features the most sterically demanding ligand in the series. The ^1^H and ^19^F NMR spectra also suggest evidence for adduct formation, although to varying extents within the series; compound **2c** provides the clearest example of this (Figures S31 and 32). At this point, the identity of this Lewis base remains unclear, but simulating an additional C_4_H_8_O_2_ to the mass of the expected molecular-ion fits well with the experimental observations. This would suggest either dioxane or ethyl acetate, but because neither of these compounds was included in the synthetic procedure, we can only speculate as to their origin. Regardless, this observation hints strongly at the potential for these compounds to act as Lewis acids, which may have downstream applications in catalysis.^40,41^

#### Molecular Structure

**Figure 3:**
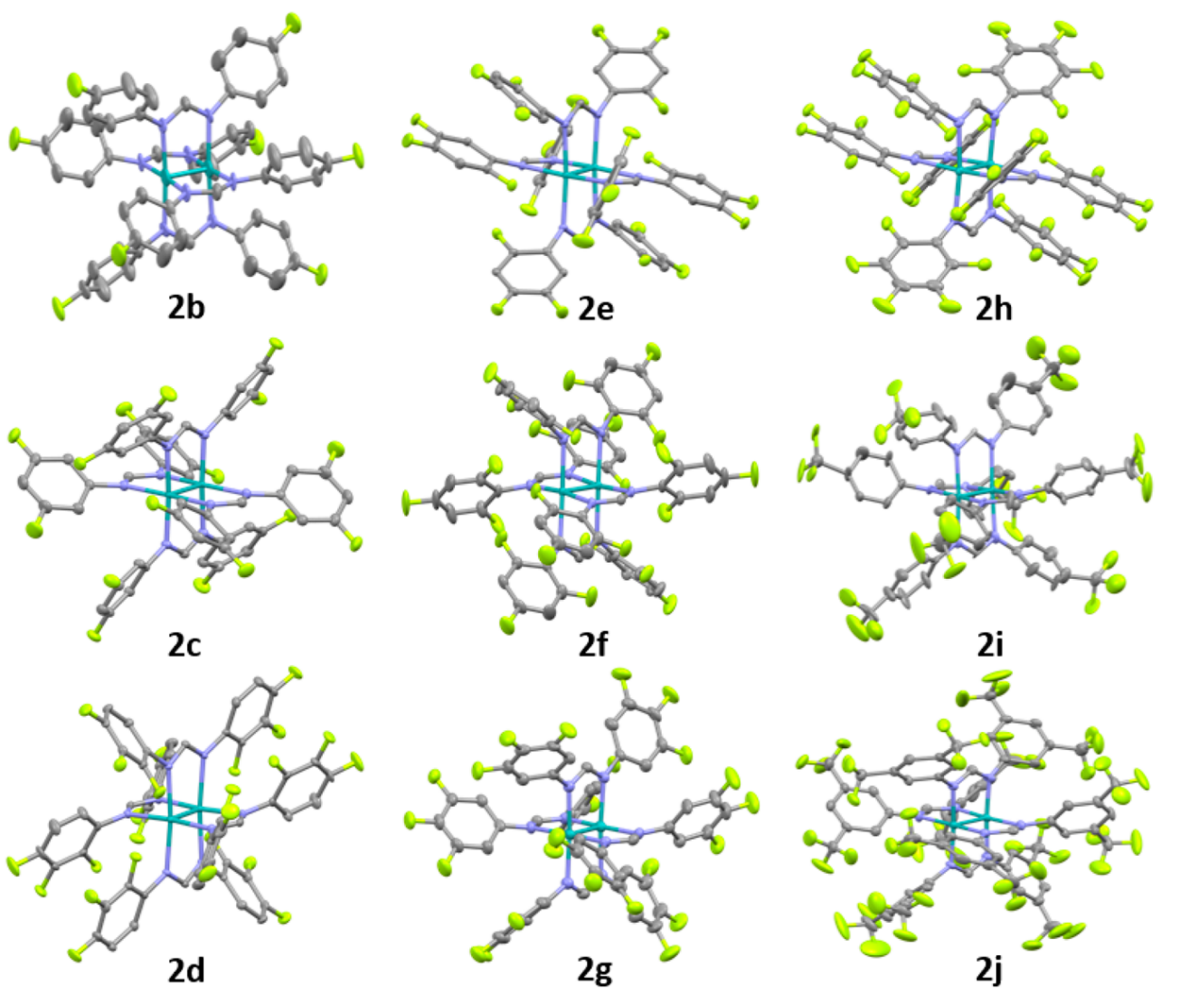
Visualizations of the X-ray crystal structures of compounds **2b**–**2j**. Hydrogen atoms and solvents of crystallization are removed for clarity, and thermal ellipsoids are reported at 50% probability. For disordered components, the highest occupancy conformation is shown. Color code: gray, carbon; lilac, nitrogen; teal, molybdenum; lime green, fluorine.

Crystals suitable for X-ray diffraction (XRD) studies were obtained for compounds **2b**–**2j** (Figure 3). In most cases, crystals were obtained by the slow diffusion of hexane into a THF solution of the complex. However, **2g** crystallized by cooling a hot saturated 1,2-difluorobenzene (DFB) solution, and **2e** was crystallized by the slow diffusion of hexane into a DFB solution of the complex. The unit cells exhibited a range of symmetries that indicate the sensitivity of the packing structure to the nature of the arene substitution pattern and the solvent system that the crystal was grown from. Importantly, none of the structures showed evidence of axial coordination, although many did crystallize with solvent molecules in interstitial sites. The Mo–Mo bond lengths occur in the range 2.0924(7)–2.1035(13) Å, placing these complexes within the normal range for Mo_2_PWCs (Table 1).^42^

**Table 1:** Mo–Mo Bond Lengths for Complexes **2a**–**2j** Obtained from Experimental Crystallography Data or from Gas-Phase Quantum-Chemical Calculations [DFT and B3LYP/def2_SV(P)]

compound	crystallographic Mo–Mo bond length/Å	crystallographic average Mo–N bond length/Å	calculated Mo–Mo bond length/Å	calculated average Mo–N bond length/Å
**2a**	2.0944(8)^a^	2.1582[15]^a^	2.0816	2.1882
**2b**	2.0934(7)	2.1577[24]	2.0816	2.1868
**2c**	2.0992(7)	2.1617[7]	2.0804	2.1883
**2d**	2.1024(12)	2.1674[18]	2.0802	2.1886
**2e**	2.0999(52)	2.1579[14]	2.0894	2.1936
**2f**	2.1022(59)	2.1693[19]	2.0877	2.2016
**2g**	2.0924(7)	2.1574[21]	2.0804	2.1871
**2h**	2.0999(7)	2.1765[10]	2.0871	2.2025
**2i**	2.1035(13)	2.1624[47]	2.0806	2.1891
**2j**	2.0952(8)	2.1630[2]	2.0802	2.1886
**3c**			2.0766	2.1674
				2.1370 (Mo–O)

a Obtained from ref (21).

In a series of previously reported terephthalate-bridged “dimers of dimers”, [Mo_2_(NN)_3_]_2_(μ-O_2_CC_6_H_4_CO_2_) where NN = para-functionalized diarylformamidinate ligands, the Mo–Mo bond length was found to decrease with the electron-withdrawing ability of the para substituent.^23^ However, in a series of homoleptic formamidinate complexes, the remote substituents appeared to have little influence on the geometry about the dimolybdenum core.^21^ Using the data collected herein, the Mo–Mo and average Mo–N bond lengths were plotted against the Hammett constants for each ligand, defined as ∑*n*(*x*σ_*m*_ + *y*σ_*p*_), where *n* = twice the number of formamidinate ligands (i.e., 2 × 4 for **2x** and 2 × 3 for **3c**), σ_*m*_-F = 0.337, σ_*p*_-F = 0.062, σ_*m*_-CF_3_ = 0.43, σ_*p*_-CF_3_ = 0.54, and *x* and *y* represent the number of meta and para substituents, respectively. However, in this case, no observable trend is apparent in the Mo–Mo and Mo–N distances across the series. It is important to note that Hammett parameters do not account for substituents in an ortho position because the steric effects of the substituent can influence the observed parameters. Removing compounds containing *o*-fluoro substituents (**2d**–**2f** and **2h**) from the data analysis did not lead to a stronger positive correlation between the Hammett parameter and the bond lengths, as determined by *R*^2^ in a least-squares fit analysis.

**Figure 4:**
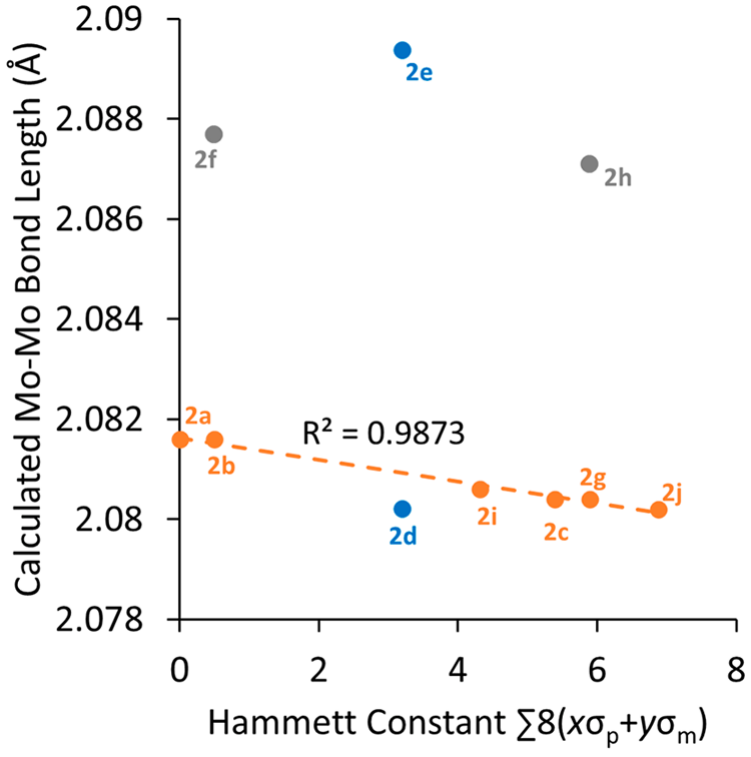
Least-squares regression plot showing the relationship between the calculated Mo–Mo bond length (Å) and Hammett constant. Blue data points contain one *o*-fluorine per arene, and gray data points contain two. *R*^2^ was calculated by excluding datapoints **2d**–**f** and **2h**.

Curious to see if these observations were largely a consequence of packing effects, the optimal geometries of the complexes in the gas phase were calculated using density functional theory (DFT) with the B3LYP functional and def2_SV(P) basis set. The resulting Mo–Mo bond lengths (Table 1) were plotted versus the Hammett constants as above (Figure 4). At first glance, there appeared to be only a weak positive correlation between the Mo–Mo bond lengths and Hammett constants. However, excluding species containing *o*-fluorine substituents (**2d**–**2f** and **2h**) from the data led to a much stronger correlation (*R*^2^ = 0.9873). The large difference in the calculated Mo–Mo bond length between **2d** and **2e** is surprising considering the similarity of their electronics, so there is likely a significant steric influence at play. Interestingly, there also appears to be a similar discrepancy in the crystallographic data for these compounds, but because they were grown from different solvent systems and have different space groups, it is hard to draw meaningful conclusions from that observation. These calculations suggest that remote substituents can have a significant influence on the length of the quadruple bond, although crystal packing effects dominate the solid-state geometries. This also highlights that, even though the vdW radius of fluorine is small, it, nevertheless, exerts a significant steric influence on the overall molecular structure when occupying the ortho position. Similarly, regardless of how the data were selected, there was no correlation between the ligand substituents and average Mo–N bond lengths for either the crystallographic or calculated data. The Mo–Mo bond length in **3c** was calculated to be notably shorter than any in the **2x** series, and this trend was also observed in other comparisons of homoleptic Mo_2_(NN)_4_ versus heteroleptic Mo_2_(NN)_3_(OAc) complexes in the literature.^23^ Formamidinate ligands are primarily π donors, meaning that the ligands donate electron density to the δ* orbital on the Mo_2_ core, thus lengthening the Mo–Mo bond. Therefore, the shorter Mo–Mo bond length in **3c** versus **2c** can be rationalized as simply a case of fewer ligands donating to the δ* orbital.

#### Electronic Structure

**Figure 5:**
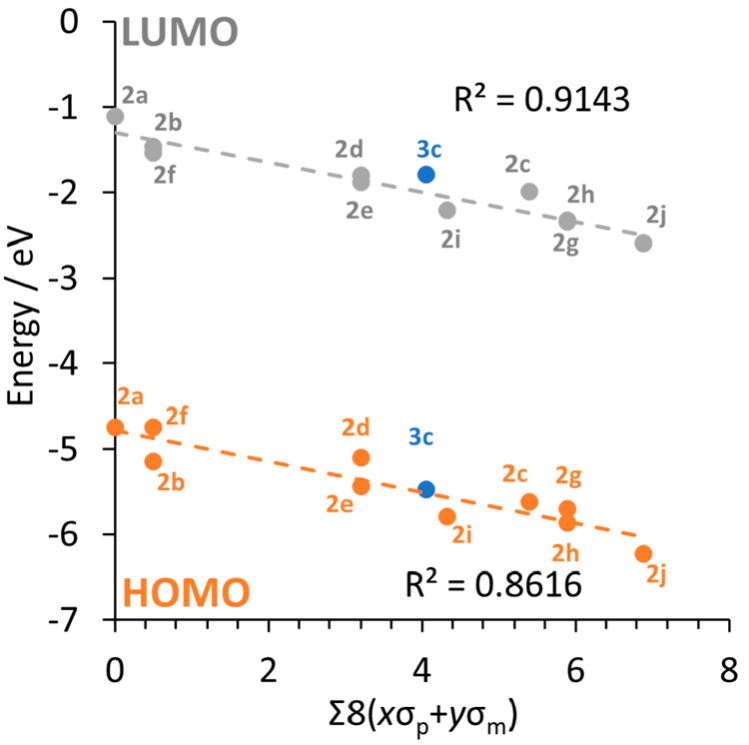
Least-squares regression plot showing the relationship between the calculated energy (PBE0/def2_TZVPP) of the frontier molecular orbitals for compounds **2a**–**2j** and their corresponding Hammett constants (assuming σ_ortho_ = 0). *R*^2^ values were calculated without including data for **3c**.

**Figure 6:**
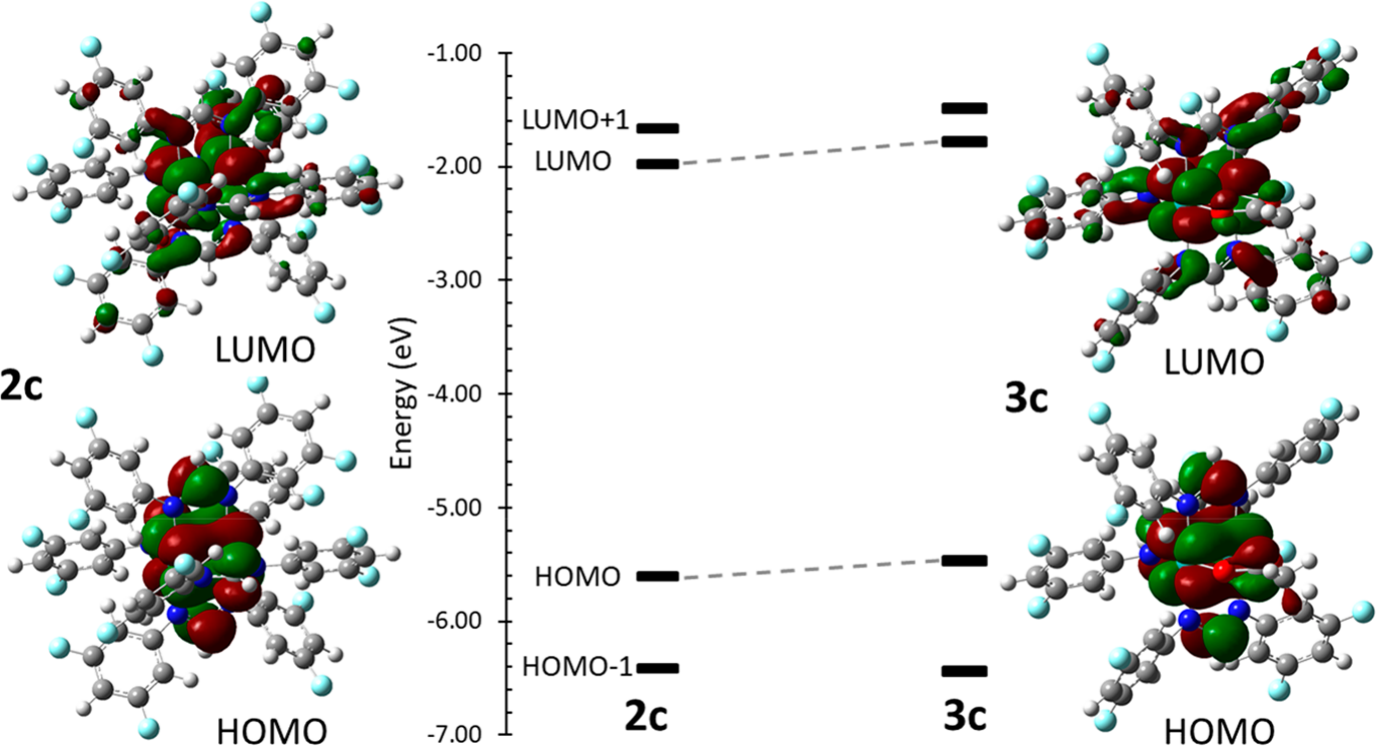
Calculated (PBE0/def2_TZVPP) frontier molecular orbitals and energy-level diagrams of **2c** (left) and **3c** (right) displayed with a course cube grid and an isovalue of 0.02. The HOMO and LUMO show mostly Mo_2_-δ and Mo_2_-δ* character, respectively.

Using the optimized geometries for complexes **2a**–**2j**, single-point calculations were performed using the PBE0 functional and def2_TZVPP basis set to interrogate the frontier molecular orbitals. The frontier molecular orbitals for all complexes can be found in Figures S96–S106. As is characteristic of most Mo_2_PWCs, the highest occupied molecular orbital (HOMO) in each case is observed to be largely Mo_2_-δ in character with out-of-phase mixing with the carbon-based p orbital on the NCN bridge of the formamidinate ligand. The lowest unoccupied molecular orbital (LUMO) in each case is largely Mo_2_-δ* but with a little mixing of the π* orbitals of the formamidinate ligands. Natural bond orbital (NBO) analysis performed on complexes **2a**–**2j** and **3c** showed the % Mo_2_-δ character to be consistent throughout the series, contributing 87–88% and 86% to the HOMO, respectively. The strong electron-withdrawing nature of the fluorine substituents is sufficient to reduce the electron density donated from the formamidinate ligands onto the dimolybdenum core, and thus the Mo_2_-δ orbital is stabilized to an extent that is largely determined by the Hammett constant of the ligands (Figure 5). Importantly, the Mo_2_-δ* orbital is also stabilized to a similar extent across the series, and, consequently, there is only a slight variation in the HOMO–LUMO energy gap, which averages out at 3.51 eV. The same trend was reported in the earlier studies on Mo_2_(NN)_4_ complexes with an average HOMO–LUMO gap of 4.78 eV,^21^ but it is likely that these energies differ only as a consequence of employing different computational methods. This consistency of the HOMO–LUMO gap across the series can also be observed experimentally. By eye, each compound is a very similar light-yellow powder, and the UV/visible absorption spectra typically show a weak transition at ca. 410 nm, which can be assigned as the Mo_2_-δ → δ* transition. This assignment is supported by quantum-chemical calculations using time-dependent DFT (TD-DFT), which showed that the lowest-energy excitation of complexes **2a**–**2j** could be identified nearly exclusively as the HOMO–LUMO transition in all complexes. Additionally, the energy of this transition was calculated to be similar for all complexes, with less than 30 nm variation across the series. The higher-energy transitions in the absorption spectrum (<300 nm) are more intense and can be assigned as combinations of Mo_2_-δ → ligand−π* metal-to-ligand charge-transfer and ligand-based π–π* transitions. These assignments are also supported by TD-DFT analysis. As with the homoleptic analogues, the HOMO and LUMO of **3c** are Mo_2_-δ and Mo_2_-δ*, respectively (Figure 6). Notably, the HOMO energy is 0.139 eV higher in energy than the corresponding homoleptic complex **2c** at the same level of theory (PBE0/def2-TZVPP), and the reasons for this are likely the loss of a single electron-withdrawing fluoroformamidinate ligand and the replacement of two nitrogen-donor atoms for oxygen.

**Figure 7:**
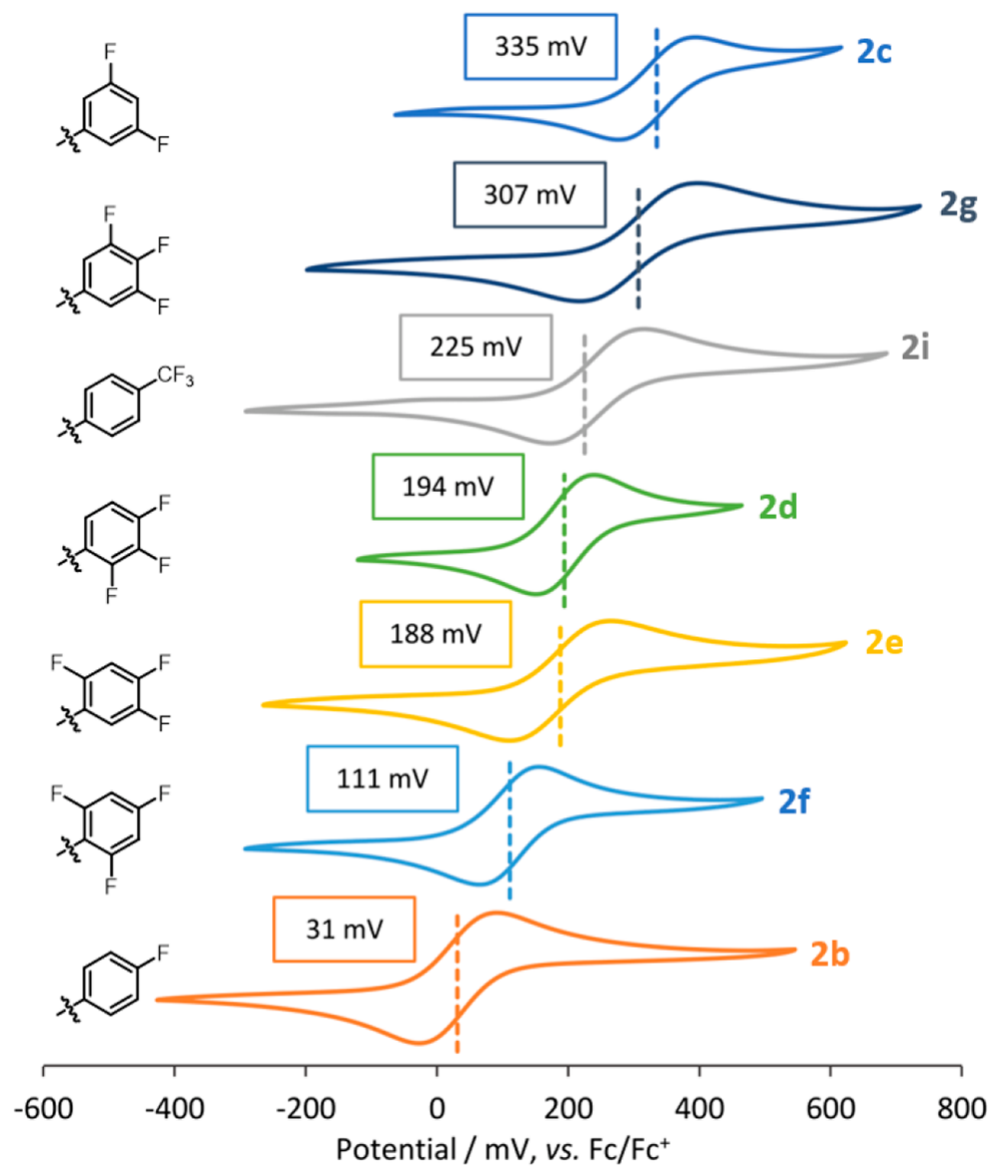
Cyclic voltammetry data for each compound annotated with their fluorine substitution pattern. In all cases, the initial scan direction was from low to high potential.

Compounds **2b**–**2j** were analyzed by cyclic voltammetry in a solution of THF using N^n^Bu_4_PF_6_ (0.1 M) as the supporting electrolyte (Figure 7). All potentials are reported versus the Fc/Fc^+^ redox couple and have been corrected for solution resistance effects (Rs) using Rs values estimated from alternating-current impedance spectroscopy.^43^ For all compounds besides **2j** and **2h**, a one-electron oxidation was observed. **2h** was insoluble in THF and produced no current response, whereas **2j** was soluble but also showed no redox events in THF. Compound **2j** [3,5-(CF_3_)_2_] has the largest overall Hammett constant of the series (σ = 6.9) and the lowest-energy HOMO according to the quantum-chemical calculations; we suggest that this is because the oxidation potential lies outside the solvent window of THF. Unfortunately, we were unable to test this hypothesis because **2j** is insoluble in all common solvents with electrochemical windows reaching higher potentials including acetonitrile and 1,1,1,3,3,3-hexafluoropropan-2-ol. In all cases where the Mo_2_^4+^/Mo_2_^5+^ couple was observed, it was chemically reversible (*i*_pa_/*i*_pc_ ∼ 1); however, in some cases, Δ*E*_p_ was >100 mV and increased with increasing scan rate, thereby indicating variable electrochemical reversibility. The relevant data are summarized in Table 2.

**Table 2:** Electrochemical Data for Compounds **2b**–**2g** and **2i** Recorded at 100 mV s^–1^ in a 0.1 M Solution of N^n^Bu_4_PF_6_ in Anhydrous THF^a^

compound	*E*_pa_	*E*_pc_	Δ*E*_p_	*E*_1/2_	*i*_pa_/*i*_pc_
**2b**	91	–28	119	31	0.98
**2c**	394	276	118	335	1.22
**2d**	238	149	89	194	1.16
**2e**	264	111	153	188	1.13
**2f**	157	66	91	111	1.08
**2g**	397	217	179	307	1.13
**2i**	316	173	143	225	1.16
3c*	223	84	139	154	1.02
2c*	398	279	119	338	1.12

a All potentials are reported in mV versus Fc/Fc^+^. *Data obtained in CH_2_Cl_2_.

**Figure 8:**
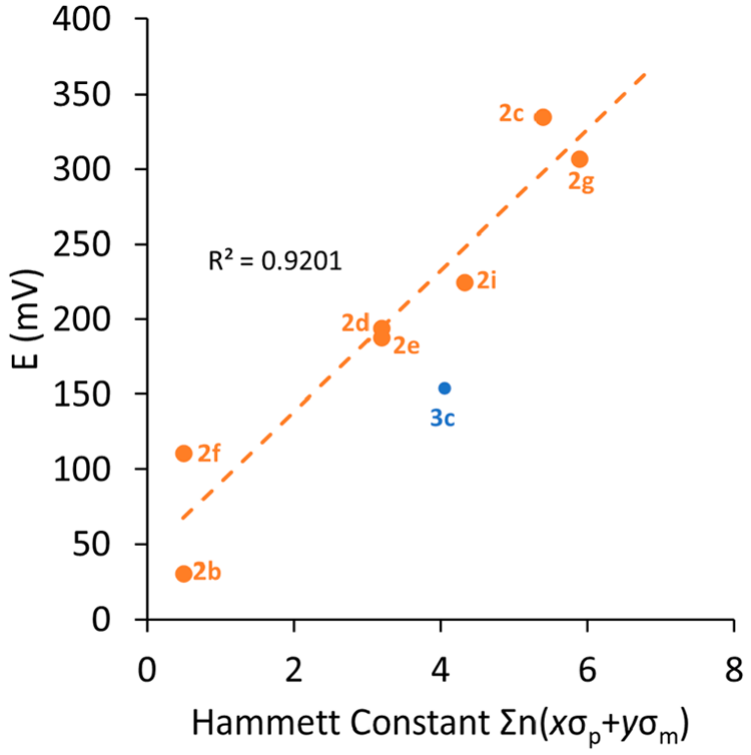
Least-squares regression plot showing the relationship between the oxidation potential of compounds **2b**–**2g**, **2i**, and **3c** and the corresponding Hammett constant. *R*^2^ values were calculated without including data for **3c**

As expected, *E*_1/2_ for the complexes in this series largely mirrors the relationship calculated for the HOMO energies. For example, compound **2b** contains only a single fluorine atom in the para position per aryl group and has an overall Hammett constant of σ = 0.5. In this case, there is little perturbation of the HOMO energy, and thus oxidation of the Mo_2_ core occurs at relatively low potentials (*E*_1/2_ = 31 mV). Conversely, compound **2c** has two fluorine atoms occupying the meta positions on each arene, with an overall Hammett constant of σ = 5.4. In this case, there is significant stabilization of the HOMO energy, which makes the complex much harder to oxidize (*E*_1/2_ = 335 mV). Figure 8 was constructed under the assumption that σ_ortho_ = 0. Compounds **2d** and **2e** both have a single *o*-fluorine substituent on each ring, while compound **2f** has two on each ring. These clearly have an influence on the redox potential of the Mo_2_ core, particularly in a comparison of **2b** and **2f**, which are formally assigned with the same Hammett constant (σ = 0.5). In previous reports of a family of fluorinated diruthenium benzoates, the authors were able to assign a pseudo-Hammett parameter of σ_ortho_ = 0.2, which was valid because the ortho substituents were sufficiently removed from the Ru_2_ core that the steric components were almost negligible.^28,44^ However, for the Mo_2_PWCs herein, this is not the case, as shown from the aforementioned gas-phase calculations, so assigning a pseudo-Hammett constant in this case is not useful.

Compound **3c** has only three formamidinate ligands, so with a reduction in steric bulk around the Mo_2_ core, there is an increased likelihood that THF could coordinate axially. This additional electron density would make the Mo_2_ core easier to oxidize and would manifest itself as an artificially low *E*_1/2_ value for the complex. Therefore, the electrochemical data for **3c** (and **2c** for comparison purposes) were obtained in CH_2_Cl_2_. Pleasingly, the *E*_1/2_ value for **2c** in CH_2_Cl_2_ (338 mV) is very similar to that in THF (335 mV), which indicates that, in THF solutions, no THF is bound to the axial position of the paddlewheel complex. Considering the trend in the data for **2b**–**2i**, this is likely the case for the whole series and therefore means that a reasonable comparison can be drawn between **3c** and the homoleptic complexes **2x** despite being in different solvents. As anticipated from analysis of the computational results, *E*_1/2_ for **3c** was lower than that for **2c** (by ca. 18 mV), which is a consequence of fewer electron-withdrawing ligands around the core.

#### Evaluating the Air Stability

**Figure 9:**
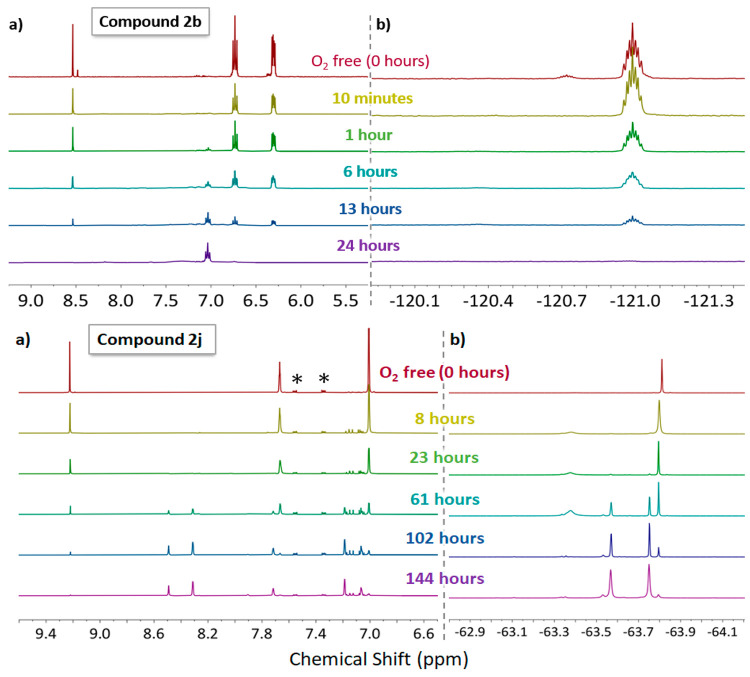
(a) ^1^H and (b) ^19^F NMR spectra of **2b** (top) and **2j** (bottom) showing oxidative decomposition of the respective compounds over time. *Residual 1,2-dichlorobenzene.

In order to test our hypothesis that strongly withdrawing ligands can lead to oxygen-tolerant Mo_2_PWCs, we monitored the degradation of selected complexes upon exposure to atmospheric oxygen using NMR spectroscopy. Our focus was initially on two complexes at either end of the Hammett spectrum, namely, **2b** (4-F) and **2j** [3,5-(CF_3_)_2_], with the latter bearing the most electron-withdrawing substituents. To obtain a baseline spectrum for each compound (Figure 9, 0 h), the NMR spectra were recorded in a J. Young NMR tube under a nitrogen atmosphere using acetone-*d*_6_, which had been previously freeze–pump–thaw-degassed and stored under nitrogen (no efforts were made to exclude moisture). For subsequent measurements, a separate solution was prepared (from the same batch of the sample) in air using a standard NMR tube and acetone-*d*_6_ straight from the bottle. The timer was started upon dissolution of the complex.

In an aerated solution, **2b** becomes light brown within a few minutes upon exposure to air. However, the NMR spectra illustrated that this considerable color change did not correspond to complete loss of the characteristic resonances. Even after 6 h, the resonances corresponding to **2b** were still the dominant signals in the NMR spectra. In the first 9 min after the compound was exposed to air, the small secondary resonances (attributed to an axially coordinated Lewis adduct of **2b**) disappeared from the ^1^H and ^19^F NMR spectra, and this was accompanied by a reduction in the intensity of the major product resonances. It is possible that this represents displacement of the axial ligand by the incoming oxygen and could hint toward an initial mechanistic step in the oxidative decomposition pathway. Alternatively, the Lewis adduct would be more electron-rich than the free complex and thus more easily oxidized and is likely consumed more quickly in the reaction. After 13 h, an opaque dark-brown solution had formed and very little **2b** was observed in the NMR spectra. The decomposition of **2j** was significantly slower and showed that, even after 23 h, a significant proportion of **2j** remained in the sample, despite the solution turning from yellow to light brown. In the ^1^H NMR spectrum, the resonances associated with **2j** were no longer the major peaks in the spectrum after 61 h, and by 144 h, the solution was dark brown and the resonances from **2j** were no longer easily distinguished from the baseline. Unfortunately, we were unable to observe the decomposition products via MALDI-TOF MS, and this was also the case for all examples herein. The species formed upon decomposition is unknown, but there is no indication that the pathway involves a loss of ligand **1j** because this has a characteristic formamidine (C–H) resonance at δH = 9.66, which does not appear at any point. To quantify the rate of decay for **2b** versus **2j**, we repeated these measurements with the inclusion of an internal standard of known concentration, 1,3,5-tris(trifluoromethyl)benzene (0.6 mmol dm^–3^). The decay curves are displayed in Figures S109 and S111 and appear to show a first-order decay with rate constants [as determined from a plot of ln(**2x**) vs time] of 0.049 h^–1^ for **2b** and 0.021 h^–1^ for **2j**, demonstrating a clear decrease in the rate of decay for the more electron-deficient Mo_2_ core of **2j**.

Complex **2j** shows an increased resistance to oxygen compared to **2b**, which can be attributed to the increasingly withdrawing ligands, leading to an overall stabilization of the Mo_2_-δ orbital. However, homoleptic complexes such as **2b**–**2j** are not overly useful in the construction of functional materials because the formamidinate ligand is kinetically inert. Conversely, heteroleptic compounds of the form Mo_2_(NN)_3_OAc are key starting materials for the construction of functionalized Mo_2_PWCs because the ^–^OAc ligand is kinetically labile and can be substituted for a more functional ligand with ease.^34,39^ Unfortunately, this increased lability of the ligands is often accompanied by an increased sensitivity under atmospheric conditions. The most commonly employed formamidinate ligand in Mo_2_ chemistry is DAniF^–^, which features a methoxy functionality in the 4 positon on the arene.^34,44^ While there are many advantages to this ligand (good solubility profile, good for growing crystals, etc.), Mo_2_(DAniF)_3_OAc remains quite air-sensitive. Curious to see if our strategies could increase the oxygen tolerance of Mo_2_(NN)_3_OAc-type complexes, **3c** was subjected to similar NMR experiments and compared to Mo_2_(DAniF)_3_OAc. Ligand **1c** was chosen specifically because the corresponding homoleptic complex (**2c**) yielded the highest measured oxidation potential in the series and thus is likely to represent the most stable option. As before, the NMR sample of **3c** was prepared using benchtop acetone-*d*_6_ under ambient conditions (see the SI for NMR spectra). Unfortunately, decomposition was rapid, and no trace of the starting material was observed after 7.5 h (Figure S113). Dissociation of carboxylates from Mo_2_PWCs is known to be solvent-dependent,^36^ and so the stability of **3c** and Mo_2_(DAniF)_3_OAc in CDCl_3_ was also explored. In this case, decomposition took significantly longer, with significant quantities of **3c** observable in the spectrum after 98 h in “oxygenated” CDCl_3_ (Figure S114), compared to complete decomposition of Mo_2_(DAniF)_3_OAc within 4 h. A quantitative analysis of this decomposition also described a first-order decay with rate constants of 0.016 h^–1^ for **3c**, which is over an order-of-magnitude slower decay than that of Mo_2_(DAniF)_3_OAc (0.44 h^–1^; Figure S119). The stability of **3c** in the solid state was also analyzed. A batch of solid, powdered **3c** was exposed to ambient atmosphere and monitored over time. At regular intervals, a sample was taken and transferred to a Schlenk tube, and an NMR sample was prepared in acetone-*d*_6_ under oxygen-free conditions, thereby “quenching” the reaction of the solid material with oxygen. Over the course of 114 h, only a slight darkening of the color was observed in the exposed batch of **3c**, and the final ^1^H NMR spectrum remained identical with the original (details are given in the SI). A second sample was left undisturbed for 170 h (Figure S120), and some darkening was observed. However, agitation of this sample at this point generated a much lighter-yellow powder, indicating that oxidation had only occurred on the exposed surface and that the bulk remained unoxidized. This was confirmed by NMR spectroscopy, which shows a seemingly unchanged spectrum even after 216 h. This is particularly remarkable because we observed powdered Mo_2_(DAniF)_3_(OAc), a well-known analogue of **3c**, to darken to a deep-red color within 60 min upon exposure to air and to black within 24 h (Figure S122). Full decomposition of the bulk of Mo_2_(DAniF)_3_OAc was confirmed by ^1^H NMR spectroscopy (Figure S123).

## Conclusion

The exceptional redox and optical properties of Mo_2_PWCs make them promising candidates for the development of functional materials; however, their sensitivity to oxygen is a severe roadblock toward that end. Herein, we have developed a family of homoleptic paddlewheel complexes derived from easily synthesized ligands with various fluorine substitution patterns. We have shown that the redox properties (*E*_1/2_) of the subsequent Mo_2_PWCs can be tuned predictably according to well-known Hammett parameters. Moreover, oxidation of the Mo_2_ core still occurs at easily accessible potentials that are relevant for application in functional materials. Studies into the oxidative decomposition of selected complexes by NMR spectroscopy showed an exceptional increase to the persistence (and a concomitant decrease of the observed decay constant) of complexes with higher *E*_1/2_ in the presence of atmospheric oxygen. Remarkably, an increased stability to oxygen was observed, even in heteroleptic complexes with kinetically labile acetate ligands. Notably, increasing the redox potential did not generate Mo_2_PWCs which are indefinitely stable to oxygen, which implies that there is a strong kinetic component to the oxidative decomposition process, and overcoming this obstacle to air stability will be the subject of future studies. However, we have unambiguously demonstrated that, through judicious choice of a fluorination pattern on the ligands, Mo_2_PWCs with remarkable tolerance to atmospheric oxygen can be accessed, which are particularly stable in the solid state. Furthermore, complexes with bespoke and functionally useful electrochemical properties can be designed and prepared with ease, paving the way for translating Mo_2_PWCs into functional materials.

## Experimental Section

### Materials and Methods

All reagents were purchased from commercial sources and used without further purification. Anhydrous THF and hexane were obtained from an Innovative Technology Inc. PureSolv solvent purification system. Anhydrous 1,2-dichlorobenzene, CDCl_3_, CD_2_Cl_2_, and deuterated dimethyl sulfoxide (DMSO-*d*_6_) were obtained by drying over calcium hydride overnight before distillation. Anhydrous ethanol was obtained via distillation over magnesium and iodine. Acetone-*d*_6_ and 1,2-difluorobenzene (DFB) were deoxygenated via three freeze–pump–thaw cycles under nitrogen. ^1^H, ^13^C, and ^19^F NMR spectra were recorded on a JEOL ECX400 or a JEOL ECS400 spectrometer, operating at 400 MHz for ^1^H, 100 MHz for ^13^C, and 376 MHz for ^19^F, or a Bruker AVIII300NB Ultrashield spectrometer, operating at 300 MHz for ^1^H and 282 MHz for ^19^F, respectively; all spectral data were acquired at 298 K. Chemical shifts (δ) are quoted in parts per million (ppm). The residual solvent peaks δ_H_ 7.27 and δ_C_ 77.16 for CDCl_3_, δ_H_ 5.32 and δ_C_ 53.84 for CD_2_Cl_2_, δ_H_ 2.50 and δ_C_ 39.52 for DMSO-*d*_6_, and δ_H_ 2.05 and δ_C_ 29.84 for acetone-*d*_6_ were used as references. Coupling constants (*J*) are reported in hertz to the nearest 0.1 Hz. The multiplicity abbreviations used were as follows: s, singlet; d, doublet; t, triplet; q, quartet; m, multiplet; br s, broad singlet. Mass spectra (low and high resolution) were obtained by the University of York Mass Spectrometry Service, using electrospray ionization (see the SI) on a Bruker Daltonics Micro-TOF spectrometer or a solariX XR FTMS MALDI-TOF spectrometer using dithranol as the matrix in each case. Dithranol was obtained from commercial sources and used as received. CHN elemental microanalysis was performed by Graeme McAllister at the University of York using an Exeter Analytical Inc. CE-440 analyzer. UV/visible absorption spectra were recorded on a Jasco Y-560 UV/visible spectrophotometer using an airtight quartz cuvette fitted with a J. Young trap. Cyclic voltammetry experiments were recorded on a Gamry reference 600 (Gamry Instruments, Warminter, PA) under an atmosphere of dry argon in a solution of THF with 0.1 M N^n^Bu_4_PF_6_. A standard three-electrode setup was employed and consisted of a 3 mm platinum disk working electrode and two separate platinum wires for the reference and auxiliary electrodes. All potentials were referenced to the Fc/Fc^+^ redox couple and corrected for pseudopotential drift and cell resistance using values obtained from impedance measurements. Single-crystal XRD data were collected on a single source from two available sources (Cu Kα radiation, λ = 1.54184 Å; Mo Kα radiation, λ = 0.71073 Å) with an Oxford Diffraction SuperNova X-ray diffractometer. Crystals were cooled to 110 K with an Oxford Instruments CryoJet. The data were collected and refined by Adrian Whitwood and Theo Tanner, University of York. Oxidative decomposition of compounds **2b**, **2j**, and **3c** and Mo_2_(DAniF)_3_(OAc) was followed by time-course measurements of the ^1^H and ^19^F NMR spectra where appropriate. A quantitative measurement of the decomposition of each species was obtained through a comparison to an inert, nonvolatile internal standard of known concentration [1,3,5-tris(trifluoromethyl)benzene, 1.07 × 10^–2^ mol dm^–3^]. More specific details of the calculations can be found in the SI and Tables S1–S7.

### Synthesis

The formamidinate ligands, where R = Ph (**1a**), 4-F (**1b**), 3,5-F_2_ (**1c**), 2,3,5-F_3_ (**1d**), 3,4,5-F_3_ (**1g**), 2,3,4,5,6-F_5_ (**1h**), 4-CF_3_ (**1i**), and 3,5-(CF_3_)_2_ (**1j**), were previously reported.^37,45,46^

#### General Procedure for the Synthesis of Formamidine Ligands (**1a**–**1j**)

In a round-bottom flask, triethylorthoformate and the corresponding aniline were combined in a molar ratio of 1:2. The reaction mixture was heated to 140 °C for ca. 6 h before it was allowed to cool to room temperature. The crude product was recrystallized from toluene or a toluene/petroleum ether mixture, and the solids were washed with petroleum ether. Recrystallization was repeated until a colorless microcrystalline solid was obtained or until the product was analytically pure by ^1^H NMR spectroscopy and CHN microanalysis. Crystals suitable for XRD were grown either from recrystallization from a mixture of toluene/hexane (2:1) or by the slow diffusion of hexane into a toluene solution of the compound.

Nota bene: The reaction rate can be enhanced with the addition of an acid catalyst. However, care must be taken because this can lead to the formation of ligand salts, which can be difficult to remove via standard purification. For this reason, a catalyst (*p*-tolylsulfonic acid, ca. 2 mg) was used only when noncatalytic conditions proved to be stubborn.

#### *N*,*N*′-Bis(4-fluorophenyl)formamidine (**1b**)

Triethylorthoformate (2.3 mL, 14 mmol), 4-fluoroaniline (2.7 mL, 28 mmol), and *p*-tolylsulfonic acid were used. A colorless microcrystalline solid was obtained. Yield: 65.6% (2.15 g, 9.26 mmol). ^1^H NMR (400 MHz, CDCl_3_): δ_H_ 8.03 (s, 1H, NC(*H*)N), 7.00 (d, *J* = 6.7 Hz, 8H, *m*-C–*H*, *o*-C–*H*). ^19^F NMR (376 MHz, CDCl_3_): δ_F_ −120.1. ^1^H NMR (400 MHz, (CD_3_)_2_SO): δ_H_ 9.64 (s, 1H, N–*H*), 8.05 (br s, 1H, NC(*H*)N), 7.17 (br s, *o*-C–*H*) 7.07 (t, *J* = 8.8 Hz, 8H, *m*-C–*H*). ^19^F NMR (376 MHz, (CD_3_)_2_SO): δ_F_ −121.6. ^1^H NMR (400 MHz, (CD_3_)_2_CO): δ_H_ 8.74 (s, 1H, N–*H*), 8.10 (s, 1H, NC(*H*)N), 7.27–6.90 (m, 8H, *m*-C–*H*, *o*-C–*H*). ^19^F NMR (376 MHz, (CD_3_)_2_CO): δ_F_ −123.1. ESI MS. Calcd for C_13_H_10_F_2_N_2_: *m*/*z* 232.0812 ([M]^+^). Found: *m*/*z* 233.0884 ([MH]^+^). Elem anal. Calcd for C_17_H_8_F_12_N_2_: C, 67.2; H, 4.3; N, 12.0. Found: C, 67.0; H, 4.7; N, 12.0.

#### *N*,*N*′-Bis(3,5-difluorophenyl)formamidine (**1c**)

Triethylorthoformate (2.3 mL, 14 mmol) and 3,5-difluoroaniline (3.67 g, 28.2 mmol) were used. Yield: 82.2% (3.11 g, 11.6 mmol). ^1^H NMR (400 MHz, CDCl_3_): δ_H_ 8.02 (s, 1H, NC(*H*)N), 6.64 (br s, 4H, *o*-C–*H*), 6.56 (tt, *J* = 8.9 and 2.3 Hz, 2H, *p*-C–*H*). ^19^F NMR (376 MHz, CDCl_3_): δ_F_ −108.6. ^1^H NMR (400 MHz, (CD_3_)_2_SO): δ_H_ 10.25 (s, 1H, N–*H*), 8.38 (s, 1H, NC(*H*)N), 7.02–6.73 (m, 6H, *p*-C–*H*, *o*-C–*H*) ^19^F NMR (376 MHz, (CD_3_)_2_SO): δ_F_ −109.3, −110.4. ^1^H NMR (400 MHz, (CD_3_)_2_CO): δ_H_ 9.26 (s, 1H, NC(*H*)N), 8.31 (s, 1H, N–*H*), 6.93 (s, 4H, *o*-C–*H*), 6.67 (tt, *J* = 9.2 and 2.4 Hz, 2H, *p*-C–*H*) ^19^F NMR (376 MHz, (CD_3_)_2_CO): δ_F_ −111.2. ESI MS. Calcd for C_13_H_8_F_4_N_2_: *m*/*z* 268.0624 ([M]^+^). Found: 269.0696 ([MH]^+^). Elem anal. Calcd for C_13_H_8_N_2_F_4_: C, 58.2; H, 3.0; N, 10.4. Found: C, 58.5; H, 3.1; N, 10.3. Single crystals were grown by vapor diffusion of hexane into a concentrated toluene solution.

#### *N*,*N*′-Bis(2,3,4-trifluorophenyl)formamidine (**1d**)

Triethylorthoformate (1.7 mL, 10 mmol) and 2,3,4-trifluoroaniline (3.00 g, 20.4 mmol) were used. Yield: 65.2% (2.02 g, 6.65 mmol). ^1^H NMR (400 MHz, (CD_3_)_2_CO): δ_H_ 9.00 (s, 1H, N–*H*), 8.17 (s, 1H, NC(*H*)N), 7.16 (s, 4H, *o*-C–*H*, *m*-C–*H*). ^19^F NMR (376 MHz, (CD_3_)_2_CO): δ_F_ −144.0, −151.3, −162.7. ESI MS. Calcd for C_13_H_6_F_6_N_2_: *m*/*z* 304.0435 ([M]^+^). Found: *m*/*z* 305.0519 ([MH]^+^). Elem anal. Calcd for C_13_H_6_F_6_N_2_: C, 51.3; H, 2.0; N, 9.2. Found: C, 51.8; H, 2.0; N, 9.0.

#### *N*,*N*′-Bis(2,4,5-trifluorophenyl)formamidine (**1e**)

Triethylorthoformate (2.2 mL, 13 mmol) and 2,4,5-trifluoroaniline (3.87 g, 26.28 mmol) were used. Yield: 35.6% (1.42 g, 4.68 mmol). ^1^H NMR (400 MHz, CDCl_3_): δ_H_ 8.00 (s, 1H, NC(*H*)N), 7.16–6.78 (m, 4H, *o*-C–*H*, *m*-C–*H*). ^19^F NMR (376 MHz, CDCl_3_): δ_F_ −130.2, −140.2, −157.7. ^1^H NMR (400 MHz, (CD_3_)_2_SO): δ_H_ 9.52 (s, 1H, N–*H*), 7.61 (s, 1H, NC(*H*)N), 7.21–6.81 (m, 4H, *o*-C–*H*, *m*-C–*H*). ^19^F NMR (376 MHz, (CD_3_)_2_SO): δ_F_ −128.7, −141.5, −142.3. ^1^H NMR (400 MHz, (CD_3_)_2_CO): δ_H_ 9.03 (s, 1H, N–*H*), 8.17 (s, 1H, NC(*H*)N), 7.49–7.09 (m, 4H, *o*-C–*H*, *m*-C–*H*). ^19^F NMR (376 MHz, (CD_3_)_2_CO): δ_F_ −130.2, −132.9, −143.5. ESI MS. Calcd for C_13_H_6_F_6_N_2_: *m*/*z* 304.0435 ([M]^+^). Found: *m*/*z* 305.0504 ([MH]^+^). Elem anal. Calcd for C_13_H_6_F_6_N_2_: C, 51.2; H, 2.0; N, 9.2. Found: C, 51.3; H, 1.8; N, 8.9.

#### *N*,*N*′-Bis(2,4,6-trifluorophenyl)formamidine (**1f**)

Triethylorthoformate (1.7 mL, 10 mmol) and 2,4,6-trifluoroaniline (3.00 g, 20.4 mmol) were used. Yield: 70.5% (2.18 g, 7.18 mmol). ^1^H NMR (400 MHz, CDCl_3_): δ_H_ 8.27 (s, 1H, NC(*H*)N), 6.79–6.69 (m, 4H, *m*-C–*H*). ^19^F NMR (376 MHz, CDCl_3_): δ_F_ −114.2, −121.6. ^1^H NMR (400 MHz, (CD_3_)_2_SO): δ_H_ 9.24 (s, 1H, N–*H*), 7.97 (s, 1H, NC(*H*)N), 7.23 (s, 4H, *m*-C–*H*). ^19^F NMR (376 MHz, (CD_3_)_2_SO): δ_F_ −110.6, −113.8, −117.2, −122.9. ^1^H NMR (400 MHz, (CD_3_)_2_CO): δ_H_ 8.10 (s, 1H, NC(*H*)N), 6.97 (s, 4H, *m*-C–*H*). ^19^F NMR (376 MHz, (CD_3_)_2_CO): δ_F_ −114.8, −123.4. ESI MS. Calcd for C_13_H_6_F_6_N_2_: *m*/*z* 304.0435 ([M]^+^). Found: *m*/*z* 305.0508 ([MH]^+^). Elem anal. Calcd for C_13_H_6_F_6_N_2_: C, 51.2; H, 2.0; N, 9.2. Found: C, 51.4; H, 2.0; N, 9.0. Single crystals were grown by recrystallization of the compound from toluene/hexane (2:1).

#### *N*,*N*′-Bis(3,4,5-trifluorophenyl)formamidine (**1g**)

Triethylorthoformate (0.50 g, 3.4 mmol) and 3,4,5-trifluoroaniline (1.00 g, 6.80 mmol) were used. Yield: 35.0% (0.36 g, 1.19 mmol). ^1^H NMR (300 MHz, (CD_3_)_2_CO): δ_H_ 10.20 (s, 1H, N–*H*), 8.29 (s, 1H, NC(*H*)N), 7.15 (m, 4H, *o*-C–*H*). ^19^F NMR (282 MHz, (CD_3_)_2_CO): δ_F_ −135.4, −169.4. ESI MS. Calcd for C_13_H_6_F_6_N_2_: *m*/*z* 304.04 ([M]^+^). Found: 305.05 ([MH]^+^). Elem anal. Calcd for C_13_H_6_N_2_F_6_: C, 51.3; H, 2.0; N, 9.2. Found: C, 51.4; H, 1.9; N, 9.3.

#### *N*,*N*′-Bis(2,3,4,5,6-pentafluorophenyl)formamidine (**1h**)

Triethylorthoformate (0.95 mL, 5.7 mmol) and 2,3,4,5,6-pentafluoroaniline (2.10 g, 11.5 mmol) were used. Yield: 56.0% (1.22 g, 3.25 mmol). ^1^H NMR (400 MHz, CDCl_3_): δ_H_ 8.29 (s, 1H, NC(*H*)N). ^19^F NMR (376 MHz, CDCl_3_): δ_F_ −153.6, −161.3, −162.1. ^1^H NMR (400 MHz, (CD_3_)_2_SO): two species observed in a 1:1.3 minor/major ratio; δ_H_ 10.10 (s, 1H, N–*H*), 8.19 (s, 1H, NC(*H*)N). ^19^F NMR (376 MHz, (CD_3_)_2_SO): δ_F_ −144.3 (minor), −155.0 (major), −157.7 (minor), −163.2 (major), −164.8 (major), −165.8 (minor). APCI MS. Calcd for C_13_H_2_F_10_N_2_: *m*/*z* 376.0058 ([M]^+^). Found: *m*/*z* 377.0122 ([MH]^+^). Elem anal. Calcd for C_13_H_2_F_10_N_2_: C, 41.5; H, 0.5; N, 7.5. Found: C, 41.0; H, 0.42; N, 8.5.

#### *N*,*N*′-Bis(4-ααα-trifluorotolyl)formamidine (**1i**)

Triethylorthoformate (2.3 mL, 14 mmol) and 4-trifluoromethylaniline (3.5 mL, 28 mmol) were used. Yield: 56.3% (2.62 g, 7.87 mmol). ^1^H NMR (400 MHz, CDCl_3_): δ_H_ 8.19 (s, 1H, NC(*H*)N), 7.58 (d, *J* = 8.2 Hz, 4H, *m*-C–*H*), 7.15 (d, *J* = 8.2 Hz, 4H, *o*-C–*H*). ^19^F NMR (376 MHz, CDCl_3_): δ_F_ −61.8. ^1^H NMR (400 MHz, (CD_3_)_2_SO): δ_H_ 10.33 (s, 1H, N–*H*), 8.41 (s, 1H, NC(*H*)N), 7.69–7.21 (m, 8H, *m*-C–*H*, *o*-C–*H*). ^19^F NMR (376 MHz, (CD_3_)_2_SO): δ_F_ −59.9. ^1^H NMR (400 MHz, (CD_3_)_2_CO): δ_H_ 9.38 (s, 1H, N–*H*), 8.43 (s, 1H, NC(*H*)N), 7.72–7.32 (m, 8H, *m*-C–*H*, *o*-C–*H*). ^19^F NMR (376 MHz, (CD_3_)_2_CO): δ_F_ −62.1. ESI MS. Calcd for C_15_H_10_F_6_N_2_: *m*/*z* 332.0748 ([M]^+^). Found: *m*/*z* 333.0818 ([MH]^+^). Elem anal. Calcd for C_15_H_10_F_6_N_2_: C, 54.2; H, 3.0; N, 8.4. Found: C, 54.0; H, 3.0; N, 8.2.

#### *N*,*N*′-Bis[3,5-bis(αααα′α′α′-hexafluoro)xylyl]formamidine (**1j**)

Triethylorthoformate (0.28 mL, 2.3 mmol) and 3,5-bis(trifluoromethyl)aniline (0.70 mL, 4.5 mmol) were used. Yield: 51.6% (0.54 g, 1.15 mmol). ^1^H NMR (400 MHz, CDCl_3_): δ_H_ 8.11 (s, 1H, NC(*H*)N), 7.63 (m, 6H, *p*-C–*H*, *o*-C–*H*). ^19^F NMR (376 MHz, CDCl_3_): δ_F_ −62.9. ^1^H NMR (400 MHz, (CD_3_)_2_SO): δ_H_ 10.59 (s, 1H, N–*H*), 8.67 (s, 1H, NC(*H*)N), 7.76 (m, 8H, *p*-C–*H*, *o*-C–*H*). ^19^F NMR (376 MHz, (CD_3_)_2_SO): δ_F_ −61.3. ESI MS. Calcd for C_17_H_8_F_12_N_2_: *m*/*z* 468.0496 ([M]^+^). Found: *m*/*z* 469.0573 ([MH]^+^). Elem anal. Calcd for C_17_H_8_F_12_N_2_: C, 43.6; H, 1.7; N, 6.0. Found: C, 44.0; H, 1.7; N, 6.3.

#### General Procedure for Synthesis of Homoleptic Dimolybdenum Paddlewheel Complexes

Mo(CO)_6_ and formamidine ligand (**1x**) were added to a Schlenk tube in a molar ratio of 1:2. A reflux condenser was fitted, and the whole system was placed under an inert atmosphere. 1,2-Dichlorobenzene (ca. 30 mL) and tetrahydrofuran (2–4 mL) were added by cannula or syringe, and the resulting solution was stirred under reflux at 160 °C for 15 h. The solvents were then removed via vacuum distillation (at ca. 70 °C), giving a black/green solid, to which ethanol (ca. 20 mL) was added. The slurry was then sonicated and stirred, before allowing the precipitate to settle and decanting off the solution via a filter cannula. The solids were washed with ethanol a further two-to-three times to give a yellow solid, which was dried in vacuo to remove residual ethanol.

#### Dimolybdenum Tetrakis[*N*,*N*′-bis(4-fluorophenyl)formamidinate] (**2b**)

Molybdenum hexacarbonyl (0.94 g, 3.6 mmol) and **1b** (1.65 g, 7.11 mmol) were used. After ethanol washes, the solution was washed with 10 mL of diethyl ether, the solvent decanted, and the product dried in vacuo. A yellow powder was obtained. Yield: 30.0%. ^1^H NMR (400 MHz, CDCl_3_): δ_H_ 8.36 (s, 4H, NC(*H*)N), 6.66 (dd, *J* = 12.7 and 8.5 Hz, 16H, *m*-C–*H*), 6.15–6.09 (m, 16H, *o*-C–*H*). ^19^F NMR (376 MHz, CDCl_3_): δ_F_ −119.8. ^1^H NMR (400 MHz, (CD_3_)_2_CO): δ_H_ 8.57 (s, 4H, NC(*H*)N), 6.78–6.72 (m, 16H, *m*-C–*H*), 6.36–6.31 (m, 16H, *o*-C–*H*). ^19^F NMR (376 MHz, (CD_3_)_2_CO): δ_F_ −121.66 (m). MALDI-TOF MS. Calcd for C_52_H_36_F_8_Mo_2_N_8_: *m*/*z* 1120.10 ([M]^+^). Found: *m*/*z* 1120.10 ([M]^+^). Single crystals were grown by the slow diffusion of hexane into a THF solution of **2b**.

#### Dimolybdenum Tetrakis[*N*,*N*′-bis(3,5-difluorophenyl)formamidinate] (**2c**)

Molybdenum hexacarbonyl (0.99 g, 3.8 mmol) and **1c** (2.01 g, 7.48 mmol) were used. Yield: 16.8% (0.39 g, 0.31 mmol). ^1^H NMR (400 MHz, (CD_3_)_2_CO): δ_H_ 8.84 (s, 4H, NC(*H*)N), 6.74 (tt, *J* = 9.0 and 2.1 Hz, 8H, *p*-C–*H*), 6.12–6.08 (m, 16H, *o*-C–*H*). ^19^F NMR (376 MHz, (CD_3_)_2_CO): δ_F_ −110.1 (t, *J* = 8.8 Hz). MALDI-TOF MS. Calcd for C_52_H_28_F_16_Mo_2_N_8_: *m*/*z* 1264.03 ([M]^+^). Found: *m*/*z* 1264.03 ([M]^+^). Single crystals were grown by the slow diffusion of hexane into a THF solution of **2c**.

#### Dimolybdenum Tetrakis[*N*,*N*′-bis(2,3,4-trifluorophenyl)formamidinate] (**2d**)

Molybdenum hexacarbonyl (0.65 g, 2.5 mmol) and **1d** (1.50 g, 4.93 mmol) were used. The solution was heated for 44 h and washed with toluene in addition to ethanol washes. Yield: 29.7% (0.51 g, 0.37 mmol). ^1^H NMR (400 MHz, (CD_3_)_2_CO): δ_H_ 8.87 (s, 4H, NC(*H*)N), 7.02–6.93 (m, 8H), 6.63 (s, 8H). MALDI-TOF MS. Calcd for C_52_H_20_F_24_Mo_2_N_8_: *m*/*z* 1407.95 ([M]^+^). Found: *m*/*z* 1407.95 ([M]^+^). Single crystals were grown by the slow diffusion of hexane into a THF solution of **2d**.

#### Dimolybdenum Tetrakis[*N*,*N*′-bis(2,4,5-trifluorophenyl)formamidinate] (**2e**)

Molybdenum hexacarbonyl (0.56 g, 2.1 mmol) and **1e** (1.30 g, 4.27 mmol) were used. Yield: 9.2% (0.14 g, 0.10 mmol). ^1^H NMR (400 MHz, (CD_3_)_2_CO): δ_H_ 8.84 (s, 4H, NC(*H*)N), 7.07–6.98 (m, 8H), 6.87–6.78 (m, 8H). ^19^F NMR (376 MHz, (CD_3_)_2_CO): δ_F_ −131.7, −141.8, −143.8. MALDI-TOF MS. Calcd for C_52_H_20_F_24_Mo_2_N_8_: *m*/*z* 1407.95 ([M]^+^). Found: *m*/*z* 1407.95 ([M]^+^). Single crystals were grown by the slow diffusion of hexane into a DFB solution of **2e**.

#### Dimolybdenum Tetrakis[*N*,*N*′-bis(2,4,6-trifluorophenyl)formamidinate] (**2f**)

Molybdenum hexacarbonyl (0.87 g, 3.3 mmol) and **1f** (2.00 g, 6.57 mmol) were used. Yield: 28.7% (0.66 g, 0.47 mmol). ^1^H NMR (400 MHz, THF-*d*_8_): δ_H_ 8.67 (s, 4H, NC(*H*)N), 6.58 (br, 16H, *m*-C–*H*). ^19^F NMR (376 MHz, THF-*d*_8_): δ_F_ −116.4 (t, *J* = 8.9 Hz), −121.3. MALDI-TOF MS. Calcd for C_52_H_20_F_24_Mo_2_N_8_: *m*/*z* 1407.95 ([M]^+^). Found: *m*/*z* 1407.95 ([M]^+^). Single crystals were grown by the slow diffusion of hexane into a THF solution of **2f**

#### Dimolybdenum Tetrakis[*N*,*N*′-bis(3,4,5-trifluorophenyl)formamidinate] (**2g**)

Molybdenum hexacarbonyl (0.26 g, 0.99 mmol) and **1g** (0.60 g, 2.0 mmol) were used. Yield: 29.0% (0.20 g, 0.14 mmol). ^1^H NMR (300 MHz, (CD_3_)_2_SO): δ_H_ 8.57 (s, 4H, NC(*H*)N), 6.51 (dd, *J* = 9.9 and 6.3 Hz, 16H, *o*-C–*H*). ^19^F NMR (282 MHz, (CD_3_)_2_SO): δ_F_ −135.6 (dd, *J* = 22.2 and 10.2 Hz), −166.9 to −169.4 (m). MALDI-TOF MS. Calcd for C_52_H_20_F_24_Mo_2_N_8_: *m*/*z* 1407.95 ([M]^+^). Found: 1407.95 ([M]^+^). Single crystals were grown by recrystallization from a hot DFB solution of **2g**.

#### Dimolybdenum Tetrakis[*N*,*N*′-bis(2,3,4,5,6-pentafluorophenyl)formamidinate] (**2h**)

Molybdenum hexacarbonyl (0.42 g, 1.6 mmol) and **1h** (1.20 g, 3.19 mmol) were used. The solution was heated for 21 h. Yield: 19.6% (0.26 g, 0.16 mmol). ^1^H NMR (400 MHz, (CD_3_)_2_CO): δ_H_ 9.04 (s, 4H, NC(*H*)N). ^19^F NMR (376 MHz, (CD_3_)_2_CO): δ_F_ −154.5 (d, *J* = 20.0 Hz), −162.5 (t, *J* = 21.3 Hz), −165.4 (t, *J* = 20.3 Hz). MALDI-TOF MS. Calcd for C_52_H_4_F_40_Mo_2_N_8_: *m*/*z* 1695.80 ([M]^+^). Found: *m*/*z* 1695.80 ([M]^+^). Single crystals were grown by recrystallization from a THF solution of **2h** at −40 °C.

#### Dimolybdenum Tetrakis[*N*,*N*′-bis(4-ααα-trifluorotolyl)formamidinate] (**2i**)

Molybdenum hexacarbonyl (0.80 g, 3.0 mmol) and **1i** (2.00 g, 6.03 mmol) were used. Yield: 9.8% (0.22 g, 0.15 mmol). ^1^H NMR (400 MHz, CD_2_Cl_2_): δ_H_ 8.67 (s, 4H, NC(*H*)N), 7.24 (d, *J* = 8.3 Hz, 16H, *m*-C–*H*), 6.31 (d, *J* = 8.4 Hz, 16H, *o*-C–*H*). ^19^F NMR (376 MHz, CD_2_Cl_2_): δ_F_ −62.5. ^1^H NMR (400 MHz, (CD_3_)_2_CO): δ_H_ 8.98 (s, 4H, NC(*H*)N), 7.31 (d, *J* = 8.4 Hz, 16H, *m*-C–*H*), 6.57 (d, *J* = 8.4 Hz, 16H, *o*-C–*H*). ^19^F NMR (376 MHz, (CD_3_)_2_CO): δ_F_ −62.6. MALDI-TOF MS. Calcd for C_60_H_36_F_24_Mo_2_N_8_: *m*/*z* 1520.08 ([M]^+^). Found: *m*/*z* 1520.08 ([M]^+^). Single crystals were grown by the slow diffusion of hexane into a THF solution of **2i**.

#### Dimolybdenum Tetrakis[*N*,*N*′-bis[3,5-bis(ααα-trifluoro)tolyl]formamidinate] (**2j**)

Dolybdenum hexacarbonyl (0.57 g, 2.2 mmol) and **2j** (2.00 g, 4.28 mmol) were used. The crude reaction product was washed with methanol. Yield: 17.9% (0.39 g, 0.19 mmol). ^1^H NMR (400 MHz, (CD_3_)_2_CO): δ_H_ 9.25 (s, 4H, NC(*H*)N), 7.70 (t, *J* = 1.6 Hz, 8H, *p*-C–*H*), 7.04 (d, *J* = 1.6 Hz, 16H, *o*-C–*H*). ^19^F NMR (376 MHz, (CD_3_)_2_CO): δ_F_ −63.7. MALDI-TOF MS. Calcd for C_68_H_28_F_48_Mo_2_N_8_: *m*/*z* 2063.98 ([M]^+^). Found: *m*/*z* 2063.98 ([M]^+^). Single crystals were grown by the slow diffusion of hexane into a THF solution of **2j**.

#### Dimolybdenum Tris[*N*,*N*′-bis(3,5-difluorophenyl)formamidinate] Monoacetate (**3c**)

Following a previously reported procedure,^39^ Mo_2_(OAc)_4_ (0.5 g, 1.17 mmol) was added to a Schlenk tube along with **1c** (0.94 g, 3.50 mmol) and placed under an inert atmosphere. THF (25 mL) was added, and then, with stirring, a NaOMe solution (7 mL, 0.5 M in MeOH) was added dropwise. The reaction was left to stir for 16 h before the solvent was removed in vacuo. Ethanol (20 mL) was added, and the flask was sonocated to ensure that all material was off the side of the Schlenk tube. The mother liquor was decanted off, yielding a yellow precipitate, which was washed once more with ethanol (20 mL) and once with hexane (10 mL). Evacuation of the resulting pale-yellow precipitate gave **3c** as a fine powder. Yield: 66% (0.81 g, 0.77 mmol). ^1^H NMR (400 MHz, CDCl_3_): δ_H_ 8.48 (s, 2H, *trans*-NC(*H*)N), 8.44 (s, 1H, *cis*-NC(*H*)N), 6.52 (tt, *J* = 8.8 and 2 Hz, 4H, *trans*-*p*-C–*H*), 6.17 (dd, *J* = 8 and 1.6 Hz, 8H, *trans*-*o*-C–*H*), 5.70 (dd, *J* = 8.4 and 2.4 Hz, 4H, *cis*-*o*-C–*H*), 2.73 (s, 3H, C*H*_3_). ^19^F NMR (376 MHz, CDCl_3_): δ_F_ −107.8, −108.2. MALDI-TOF MS. Calcd for C_41_H_24_F_12_Mo_2_N_6_O_2_: *m*/*z* 1052.56 ([M]^+^). Found: *m*/*z* 1055.98 ([M]^+^).

### Quantum-Chemical Calculations

All calculations were performed using the *Gaussian 16*, revision A.03, package. NBO analysis was performed using the *NBO 7.0* package. The initial geometry optimization calculations were performed at the B3LYP/def2_SV(P) level, followed by frequency calculations at the same level. Local minima were identified by the absence of imaginary frequency vibrations. In the (RI-)BP86/def2_SV(P) calculations, a 60-electron quasi-relativistic effective core potential replaced the core electrons of molybdenum. No symmetry constraints were applied during optimizations. Single-point calculations (performed using the *Gaussian 16*, revision A.03, package) on the B3LYP/def2_SV(P)-optimized geometries were performed using the hybrid PBE0 functional and the flexible def2_TZVPP basis set. TD-DFT calculations were performed using the hybrid PBE0 functional and flexible def2_TZVPP basis set using input geometries optimized at the B3LYP/def2_SV(P) level. The first 10 excitations were performed. These calculations were undertaken on the Viking Cluster, which is a high-performance computer facility provided by the University of York.

## Supporting Information Available

Calculated coordinates (XYZ)
Experimental procedures, spectra, crystallographic tables, and quantum-chemical calculations (PDF)

## Supplementary Material

ic2c02746_si_001.xyz
