# Supplementary material for: Enhancing the Air Stability of Dimolybdenum Paddlewheel Complexes: Redox Tuning through Fluorine Substituents
Source: Inorg Chem. 2022 Nov 18;61(48):19144–55. doi: 10.1021/acs.inorgchem.2c02746 (PMC9727734; doi:10.1021/acs.inorgchem.2c02746)
Supplement: Supplementary file 2 — ic2c02746_si_002.pdf [file ic2c02746_si_002.pdf]

Enhancing air-stability of dimolybdenum paddlewheel complexes: redox tuning through fluorine substituents

Imogen A. Z. Squire,[1] Christopher A. Goult,[1] Benedict C. Thompson,[1] Elias Alexopoulos,[1] Adrian C. Whitwood,[1] Theo F. N. Tanner[1] and Luke A. Wilkinson*[1]

Department of Chemistry, University of York, Heslington, York YO10 5DD, U.K

Contents

Enhancing air-stability of dimolybdenum paddlewheel complexes: redox tuning through fluorine substituents 1

Characterisation Data 4

Compound 1b 5

Compound 1c 7

Compound 1d 9

Compound 1e 11

Compound 1f 13

Compound 1g 15

Compound 1h 17

Compound 1i 19

Compound 1j 21

Compound 2b 23

Compound 2c 25

Compound 2d 27

Compound 2e 29

Compound 2f 31

Compound 2g 33

Compound 2h 35

Compound 2i 37

Compound 2j 39

Compound 3c 41

UV/Visible Absorption Spectra 43

ATR Infrared Spectra 48

Crystallography Data 49
Crystal Data for 1c 50
Crystal Data for 1d 52
Crystal Data for 1e 54
Crystal Data for 1f 56
Crystal Data for 2b 58
Crystal Data for 2c 60
Crystal Data for 2d 62
Crystal Data for 2e 64
Crystal Data for 2f 66
Crystal Data for 2g 68
Crystal Data for 2h 70
Crystal Data for 2i 72
Crystal Data for 2j 74
Electrochemistry 76
Compound 2b 76
Compound 2c 76
Compound 2d 77
Compound 2e 78
Compound 2f 78
Compound 2g 79
Compound 2i 79
Compound 3c 80
Compound 2c vs 3c in DCM 80
Quantum Chemical Calculations 81
General Computational Details 81
Molecular Orbital Diagram 81
Compound 2a 82
Compound 2b 83
Compound 2c 84
Compound 2d 85
Compound 2e 86
Compound 2f 87
Compound 2g 88
Compound 2h 89

Compound 2i 90
Compound 2j 91
Compound 3c 92
Evaluating stability to O_2 93
Solid-state decomposition of 3c compared to $Mo_2(DAniF)_3OAc$. 103
References 105

Characterisation Data

Below are the ^{1}H and ^{19}F NMR and mass spectra for compounds **1a-j**, **2a-j** and **3c**. For the NMR spectra, each spectrum is labelled with the nucleus (^{1}H or ^{19}F) and the solvent used. In the MALDI spectra of **2a-j** and **3c**, the top panel is the experimentally observed spectrum and the bottom panels are the simulated spectra. In each case, the peak indicated by the black line is the monoisotopic mass.

Compound 1b

^{1}H in $(CD_3)_2CO$

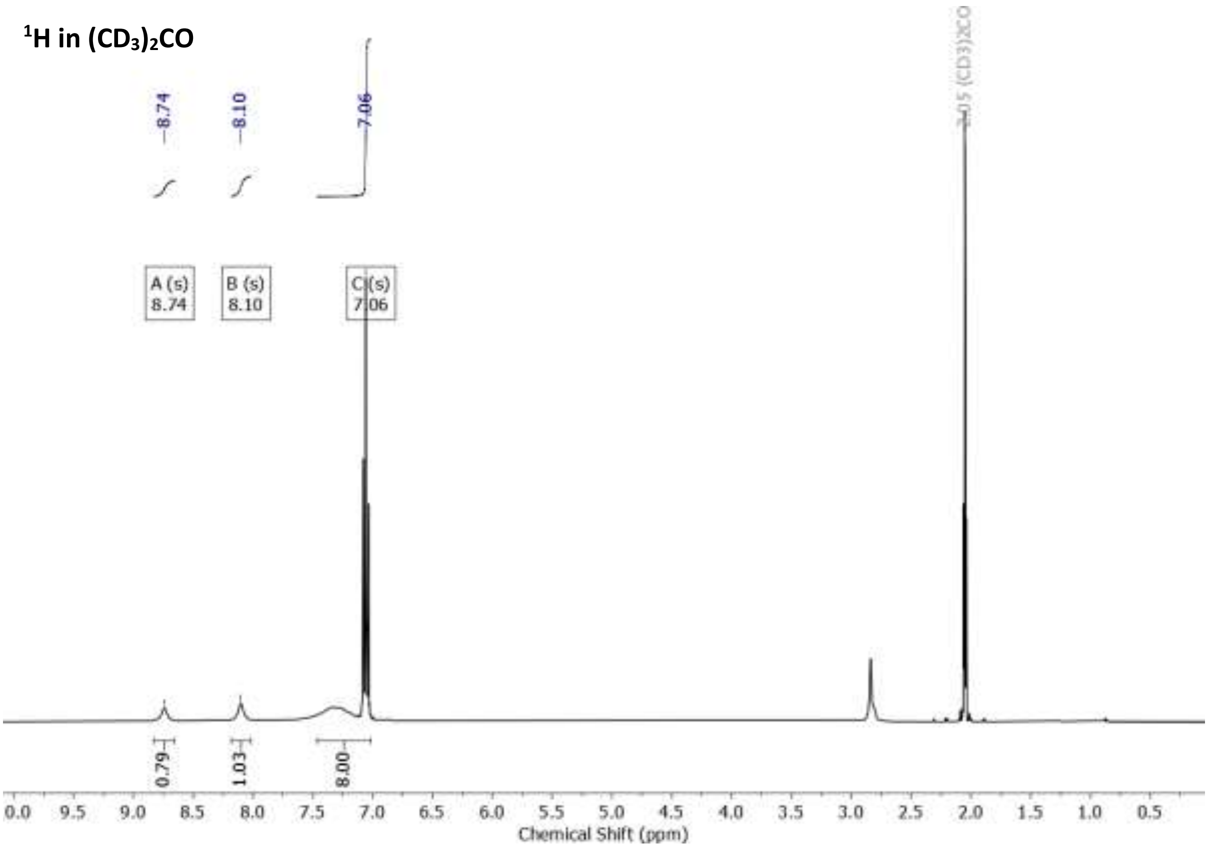

Figure S1: ^{1}H NMR spectrum of **1b** in $(CD_3)_2CO$

^{19}F in $(CD_3)_2CO$

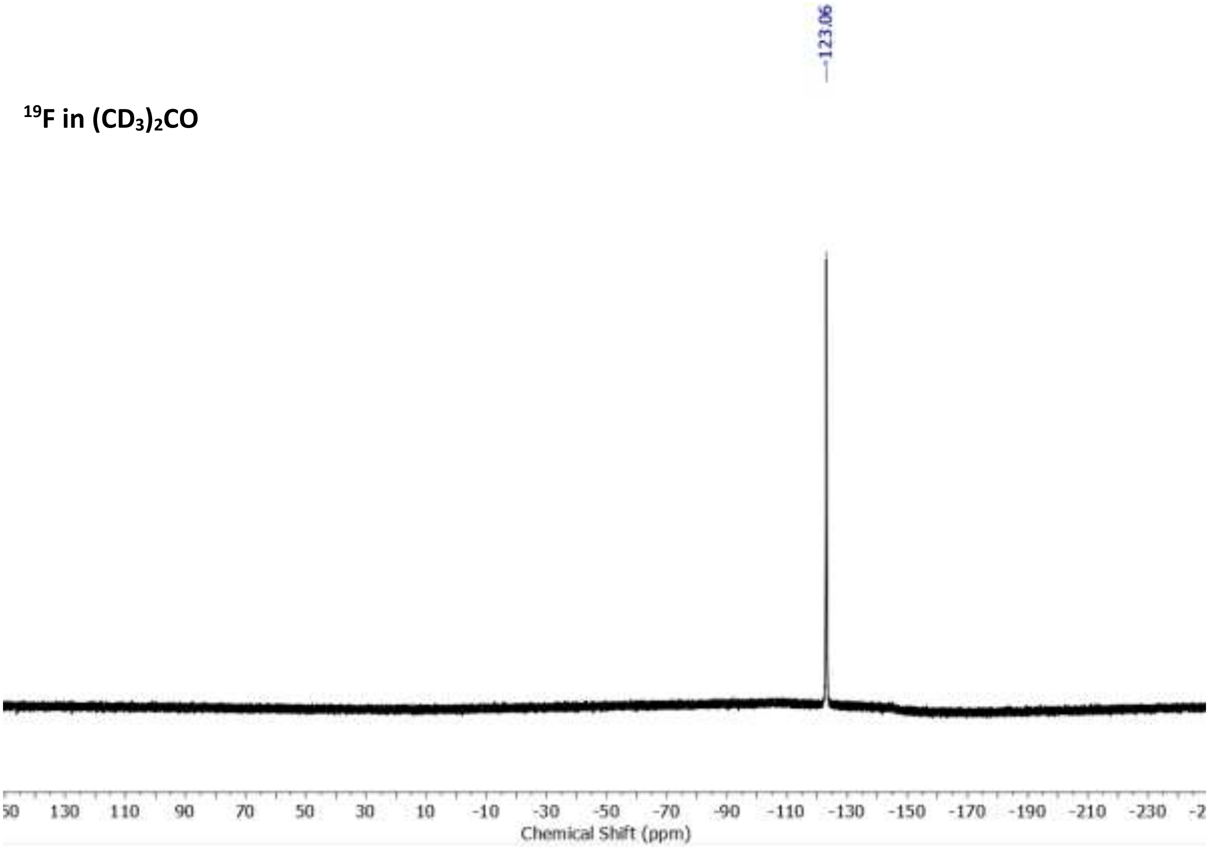

Figure S2: ^{19}F NMR spectrum of **1b** in $(CD_3)_2CO$

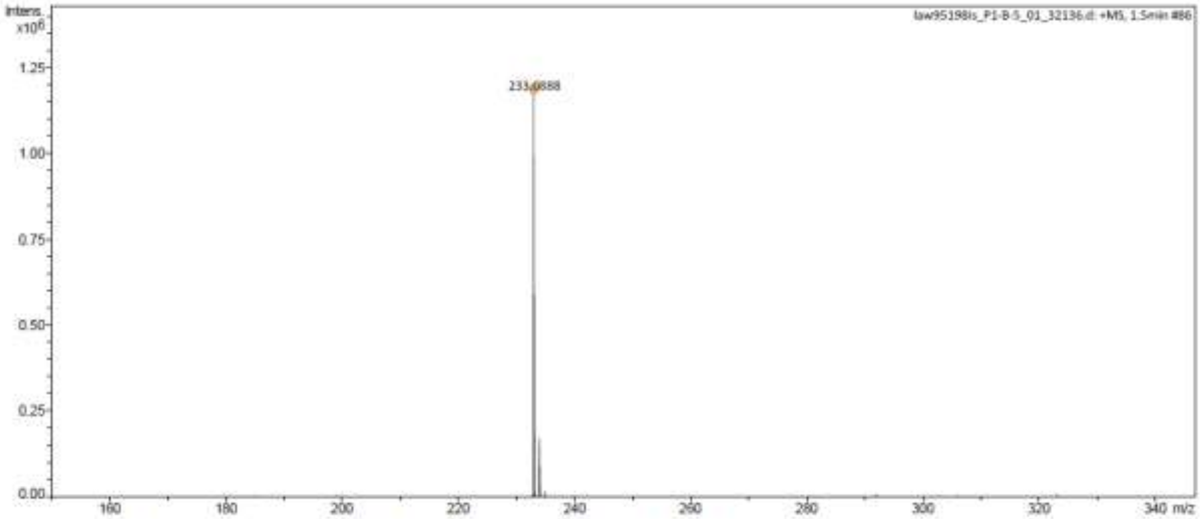

Meas. m/z	#	Ion Formula	m/z	err [ppm]	err [mDa]	mSigma	Mean err [ppm]
233.0888	1	C13H11F2N2	233.0885	-1.3	-0.3	1.7	-2.7

Figure S3: ESI mass spectrum of **1b**

Compound 1c

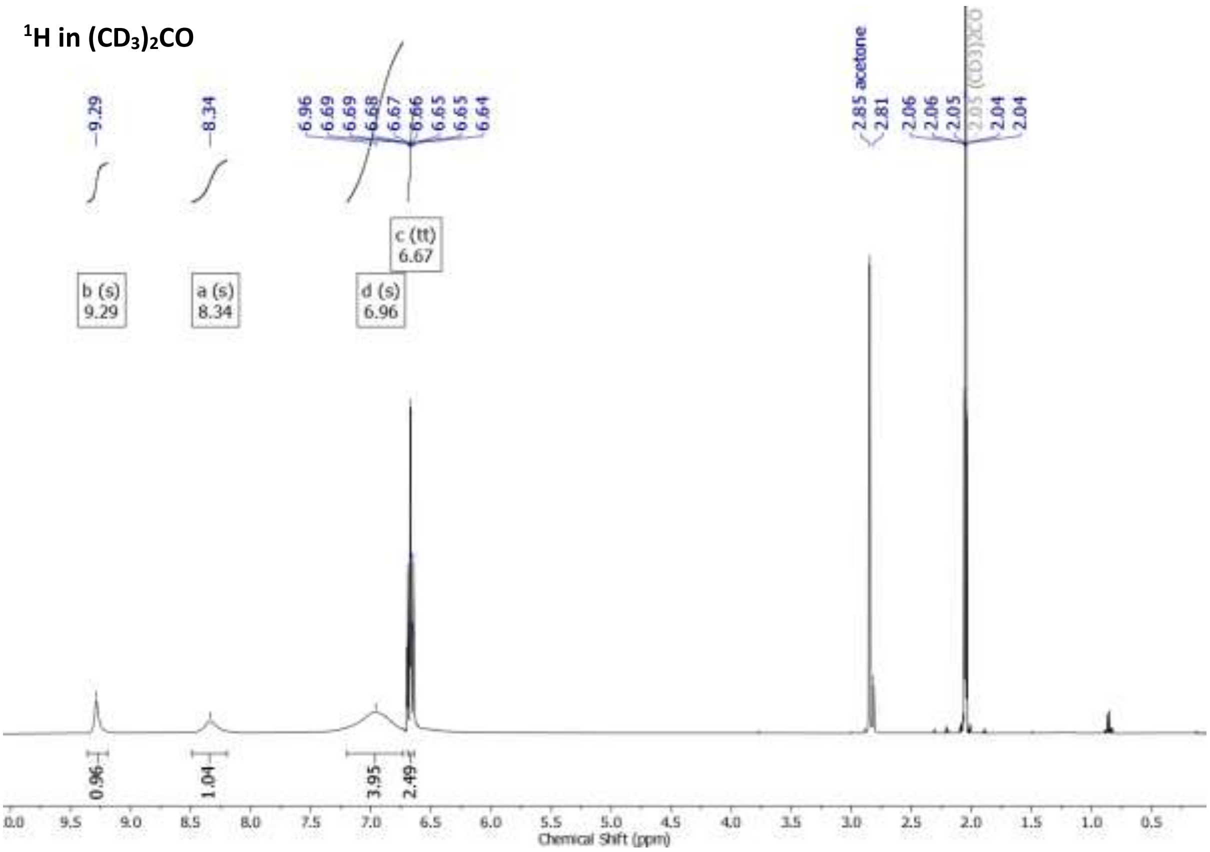

Figure S4: ^{1}H NMR spectrum of **1c** in $(CD_3)_2CO$

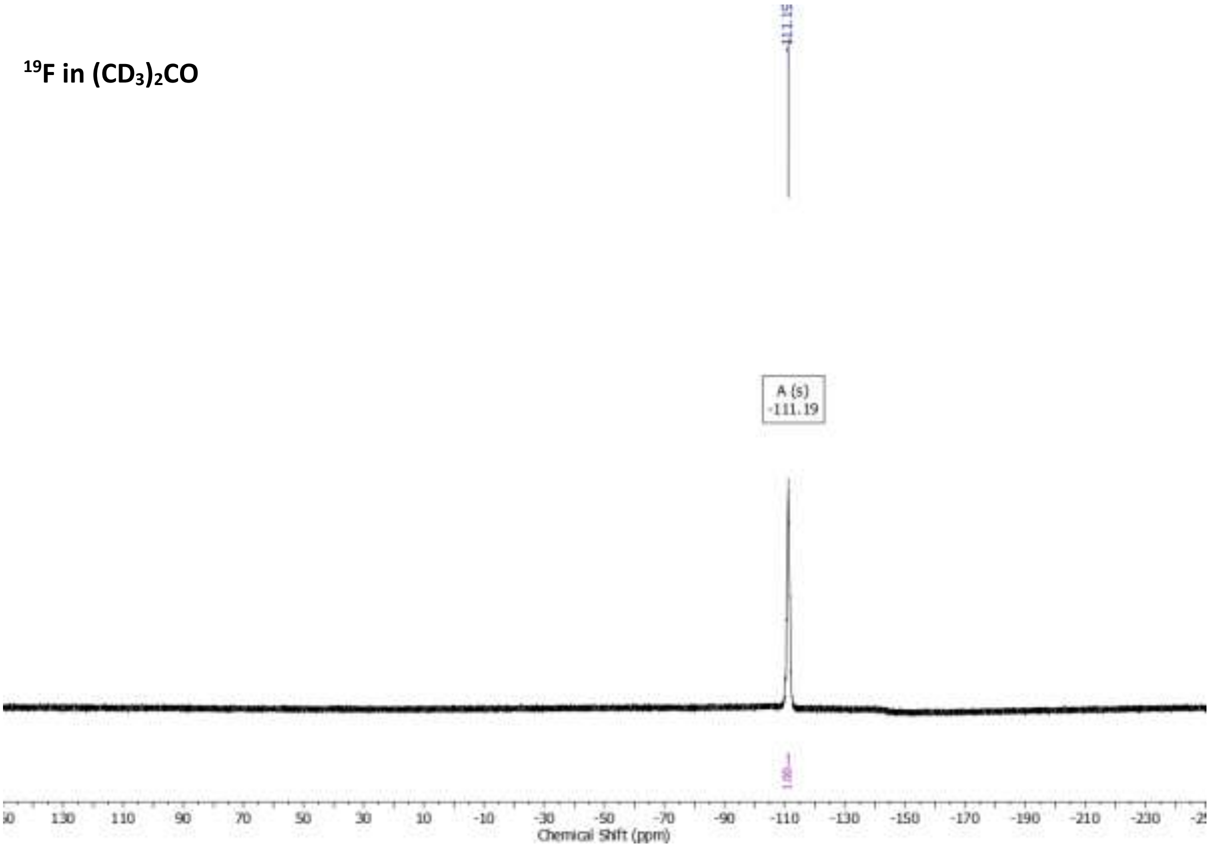

Figure S5: ^{19}F NMR spectrum of **1c** in $(CD_3)_2CO$

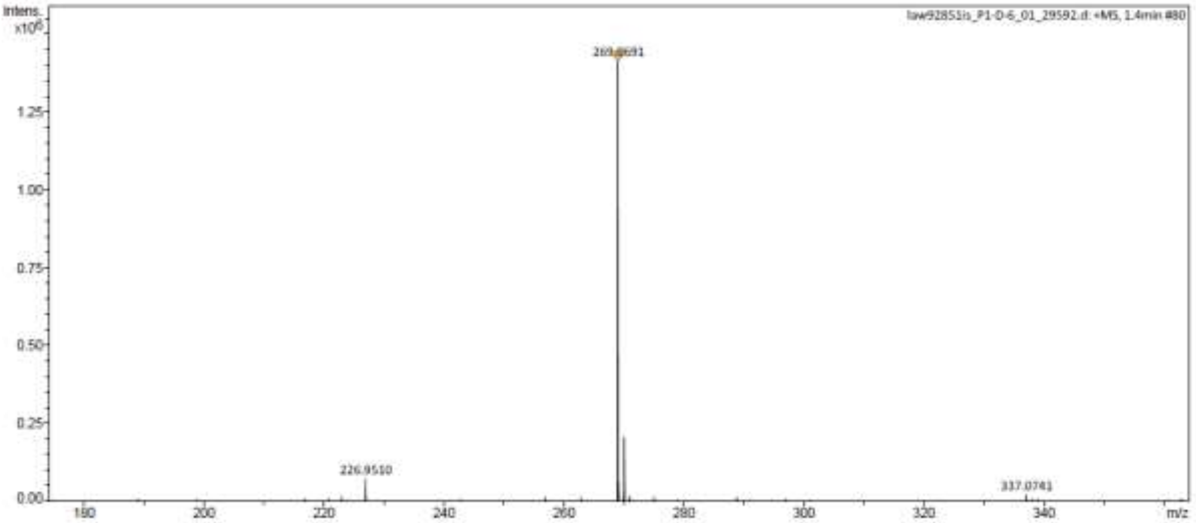

Meas. m/z	#	Ion Formula	m/z	err [ppm]	err [mDa]	mSigma	Mean err [ppm]
269.0691	1	C13H9F4N2	269.0696	2.2	0.6	1.1	1.1

Figure S6: ESI mass spectrum of **1c**

Compound 1d

^{1}H in $(CD_3)_2CO$

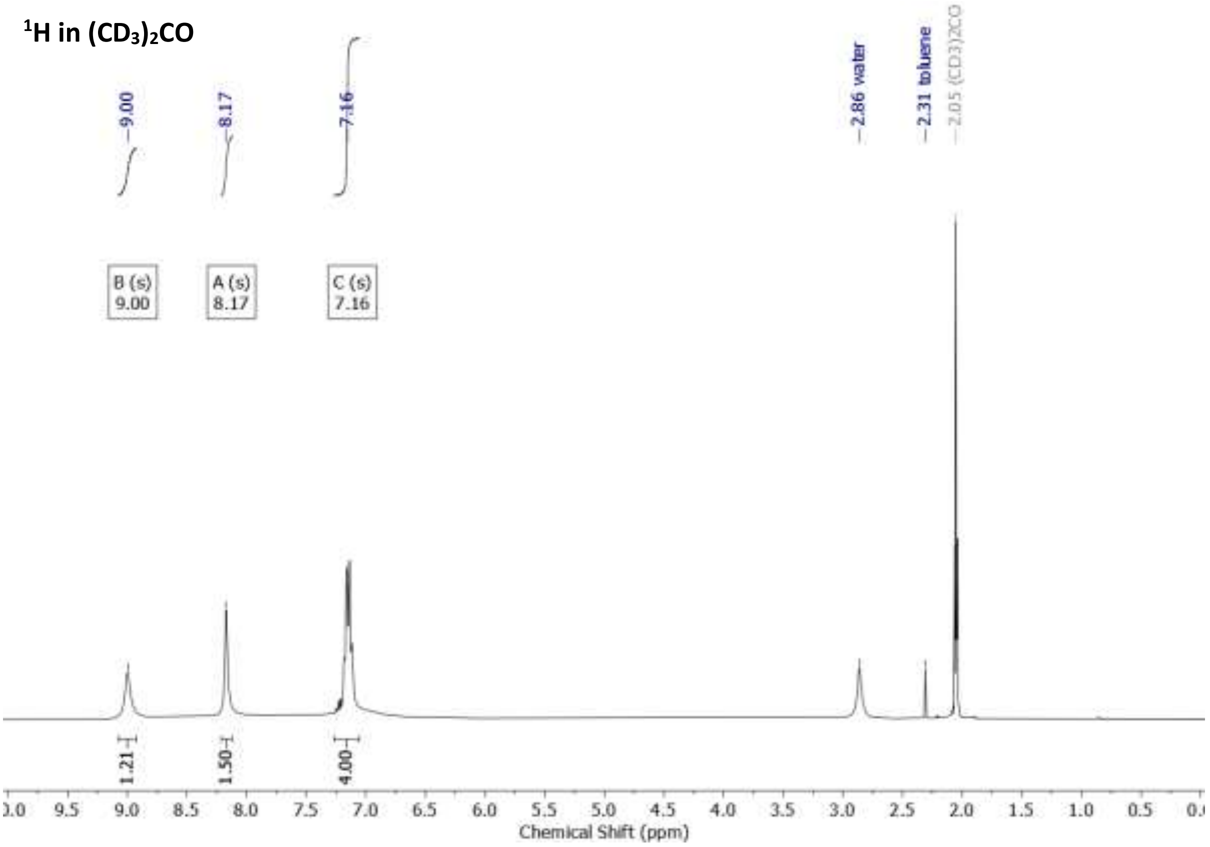

Figure S7: ^{1}H NMR spectrum of **1d** in $(CD_3)_2CO$

^{19}F in $(CD_3)_2CO$

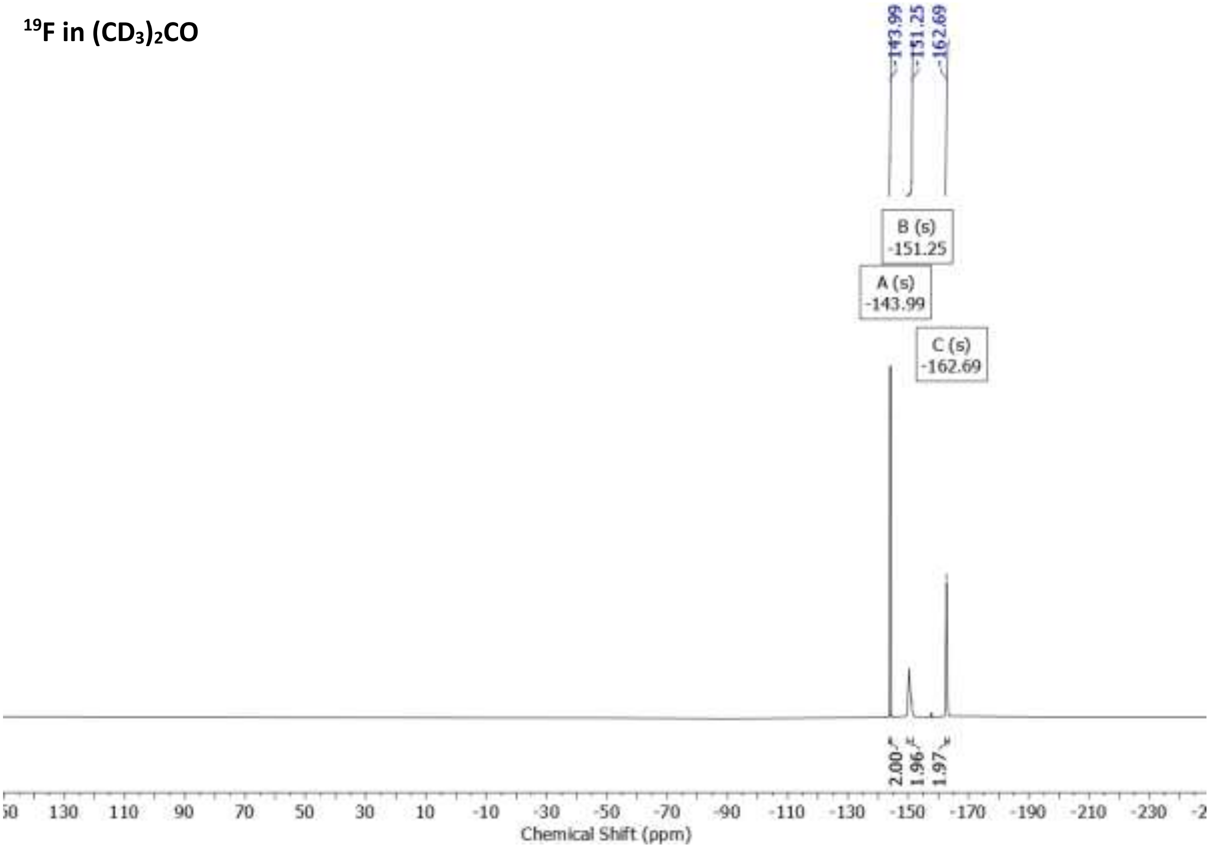

Figure S8: ^{19}F NMR spectrum of **1d** in $(CD_3)_2CO$

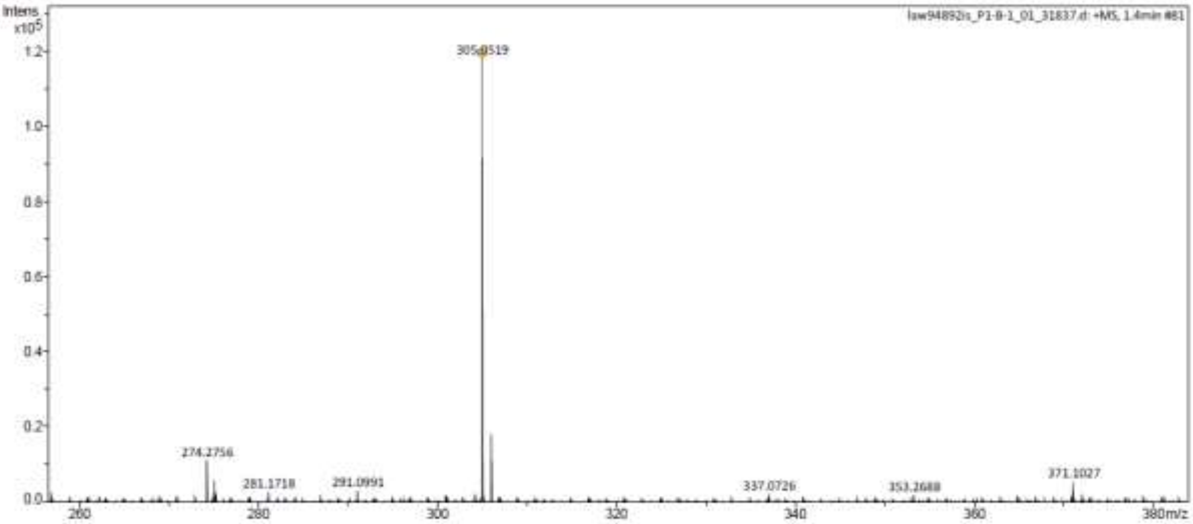

Meas. m/z	#	Ion Formula	m/z	err [ppm]	err [mDa]	mSigma	Mean err [ppm]
305.0519	1	C13H7F6N2	305.0508	-3.5	-1.1	3.7	-1.3

Figure S9: ESI mass spectrum of **1d**

Compound 1e

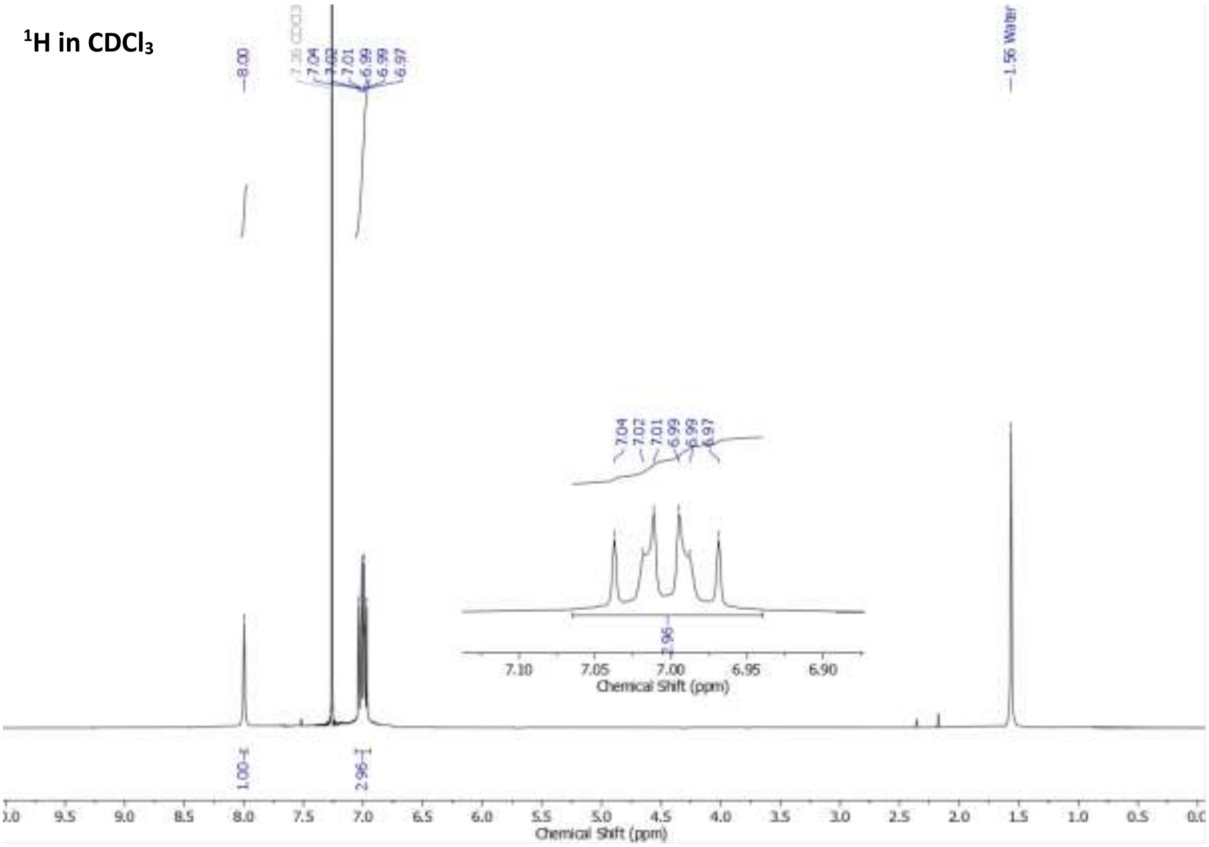

Figure S10: ^{1}H NMR spectrum of **1e** in CDCl$_3$

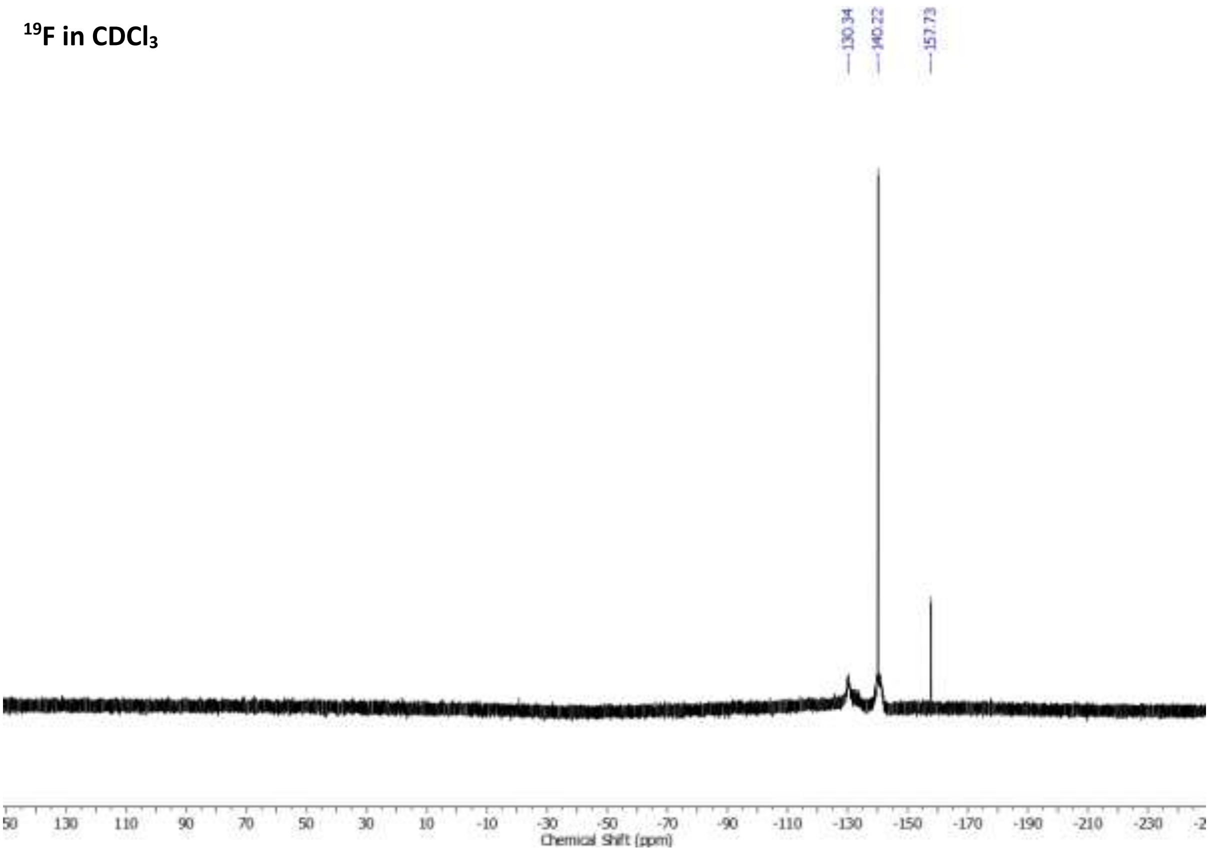

Figure S11: ^{19}F NMR spectrum of **1e** in CDCl$_3$

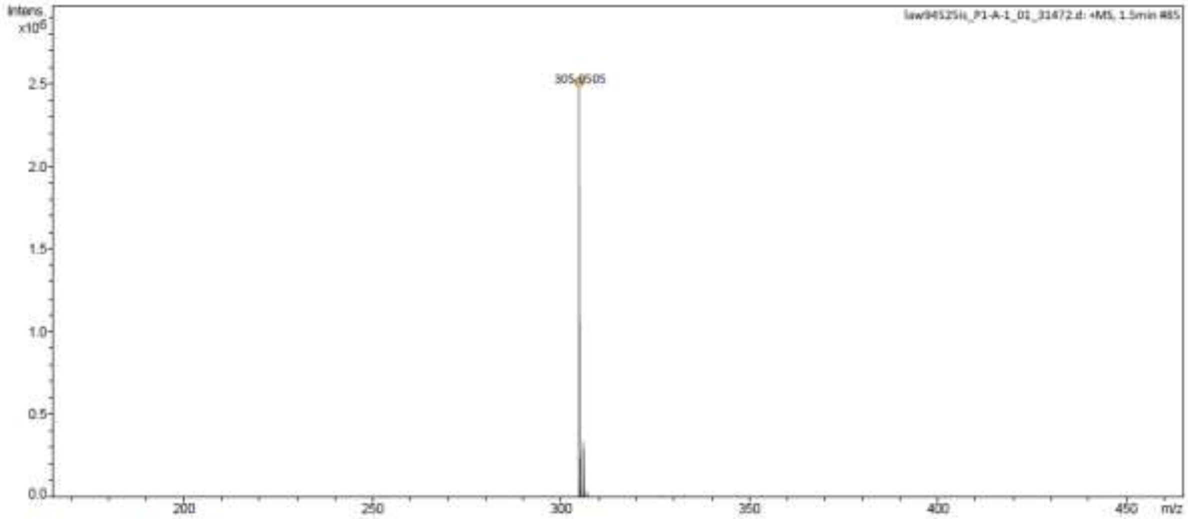

Meas. m/z	#	Ion Formula	m/z	err [ppm]	err [mDa]	mSigma	Mean err [ppm]
305.0505	1	C13H7F6N2	305.0508	0.9	0.3	7.3	0.3

Figure S12: ESI mass spectrum of **1e**

Compound 1f

^{1}H in $CDCl_3$

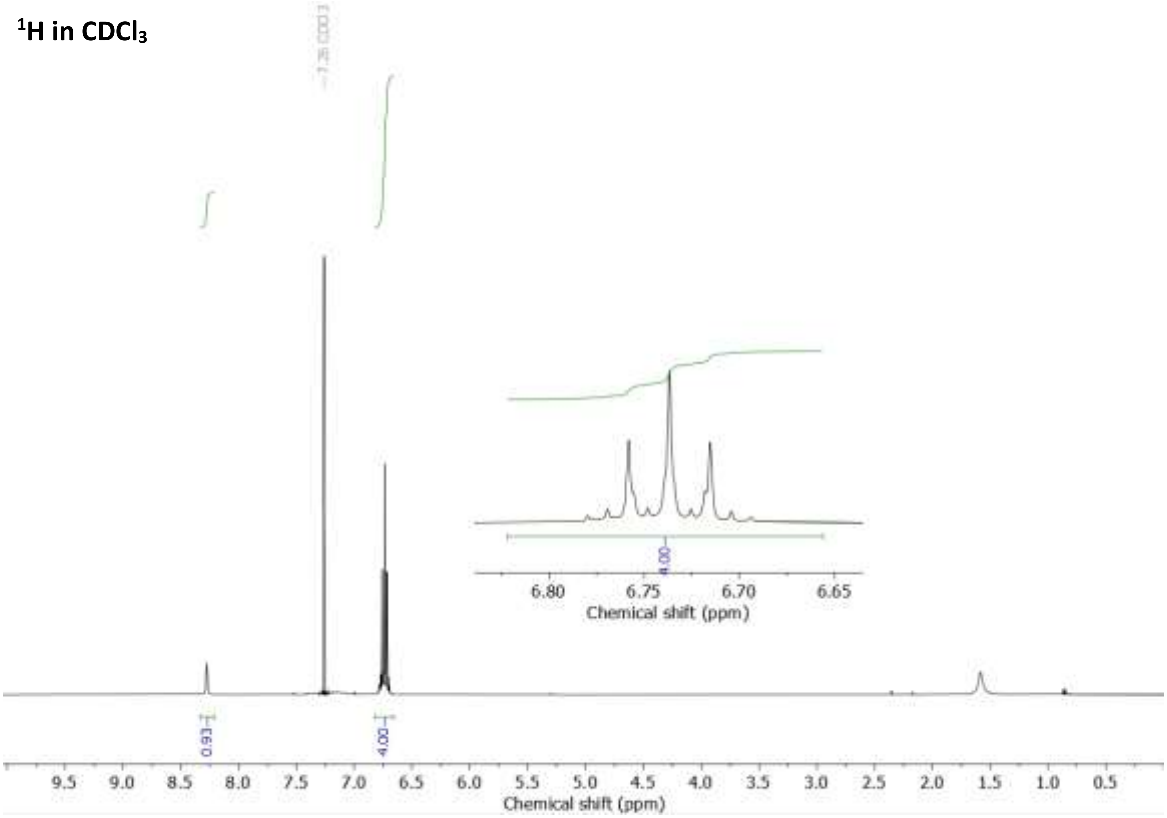

Figure S13: ^{1}H NMR spectrum of **1f** in $CDCl_3$

^{19}F in $CDCl_3$

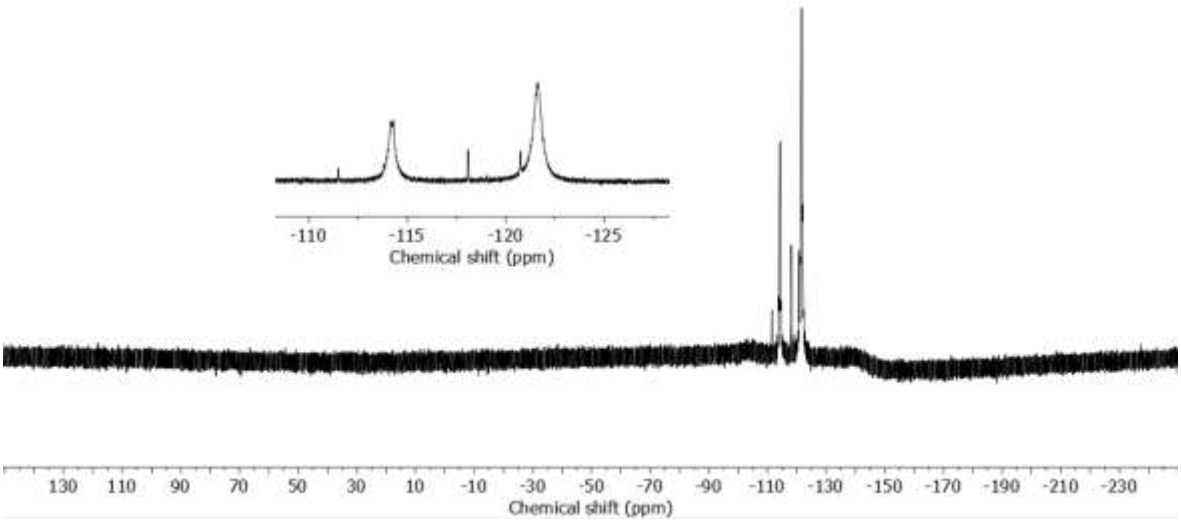

Figure S14: ^{19}F NMR spectrum of **1f** in $CDCl_3$

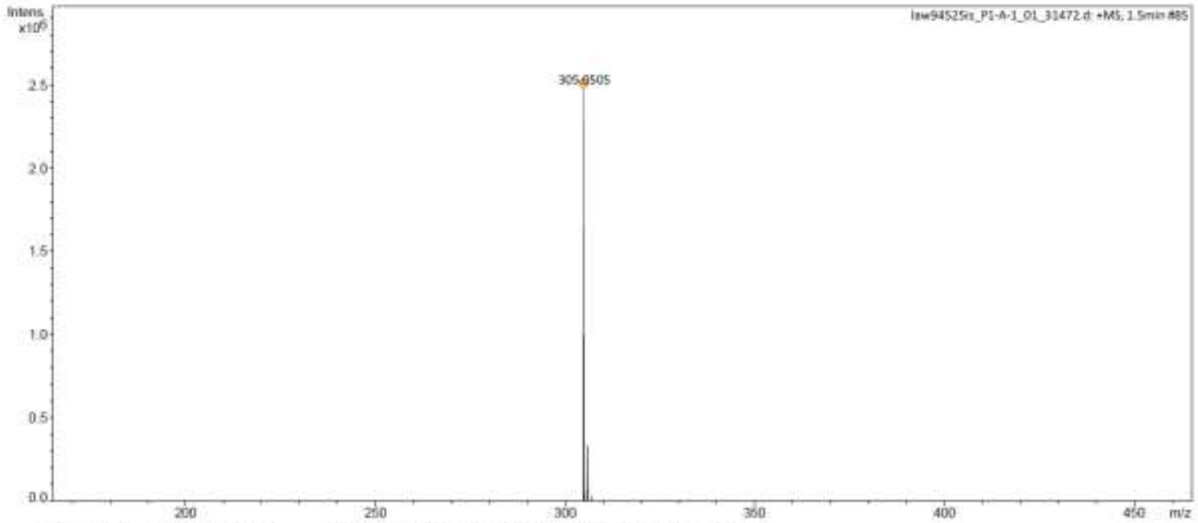

Meas. m/z	#	Ion Formula	m/z	err [ppm]	err [mDa]	mSigma	Mean err [ppm]
305.0505	1	C13H7F6N2	305.0508	0.9	0.3	7.3	0.3

Figure S15: ESI mass spectrum of **1f**

Compound 1g

^1H in $(CD_3)_2CO$

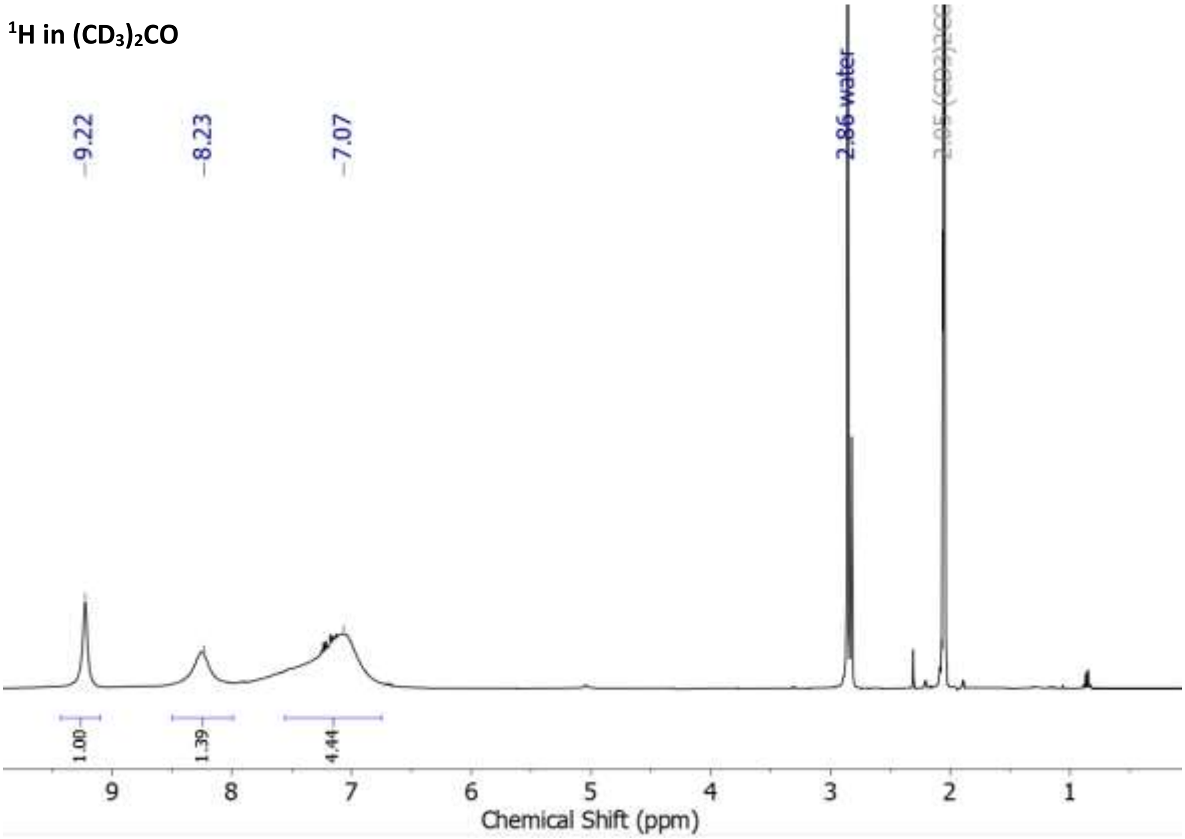

Figure S16: ^1H NMR spectrum of **1g** in $(CD_3)_2CO$

^{19}F in $(CD_3)_2CO$

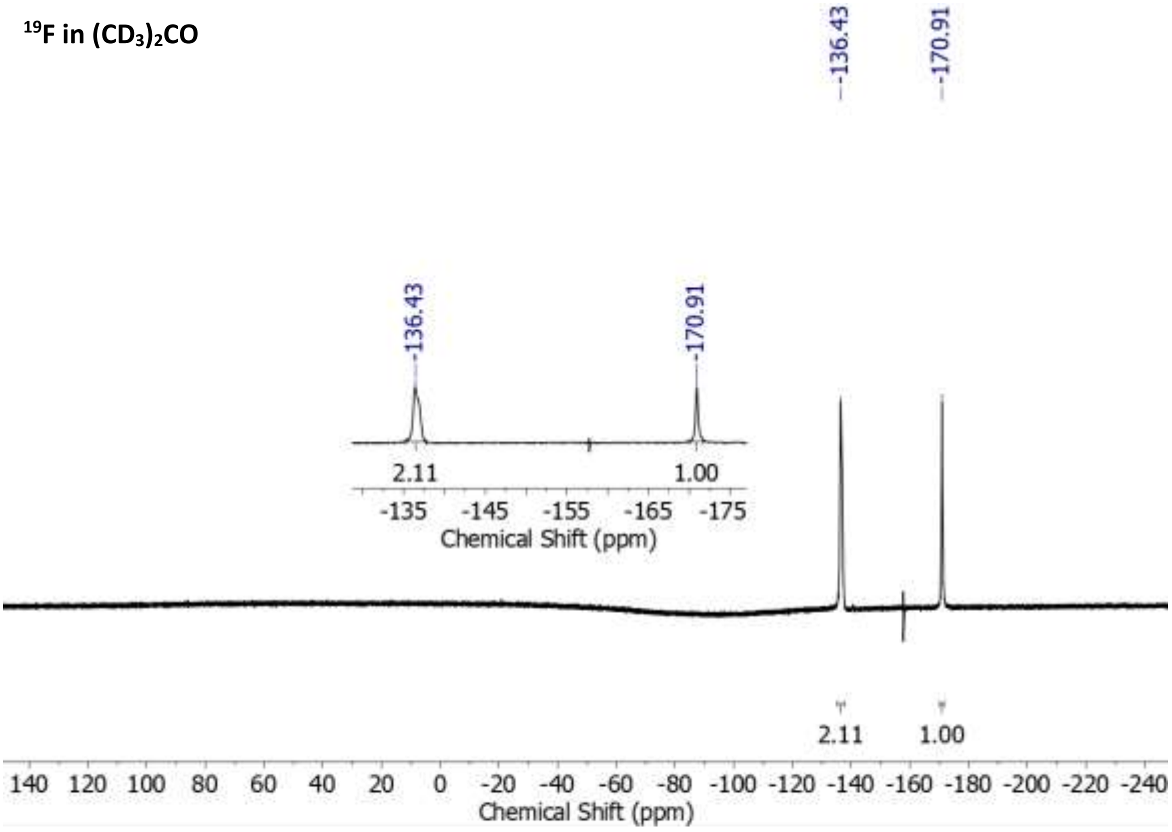

Figure S17: ^{19}F NMR spectrum of **1g** in $(CD_3)_2CO$

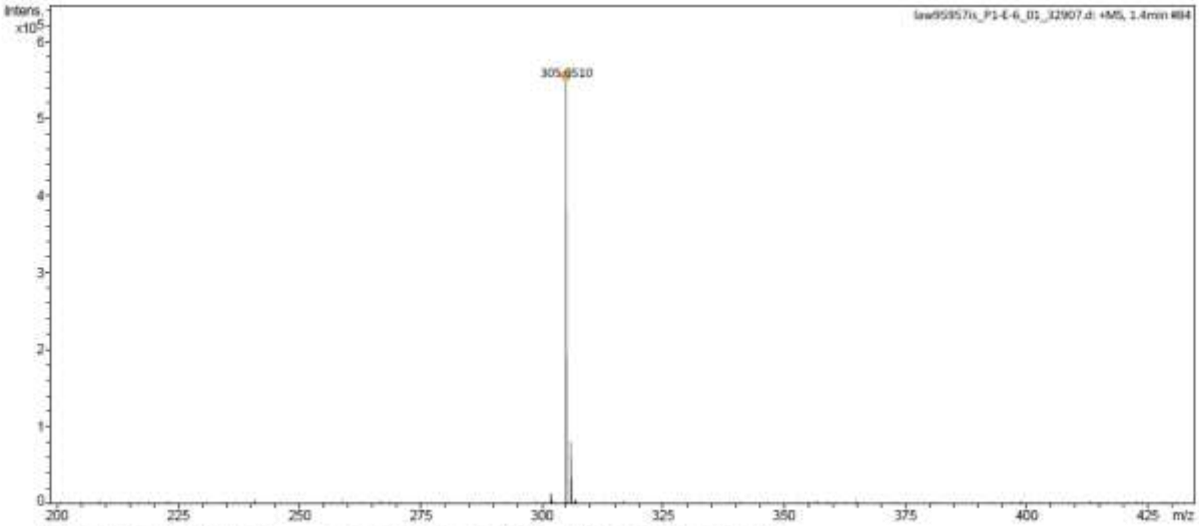

Meas. m/z	#	Ion Formula	m/z	err [ppm]	err [mDa]	mSigma	Mean err [ppm]
305.0510	1	C13H7F6N2	305.0508	-0.7	-0.2	1.2	-0.7

Figure S18: ESI mass spectrum of **1g**

Compound 1h

^{1}H in $CDCl_3$

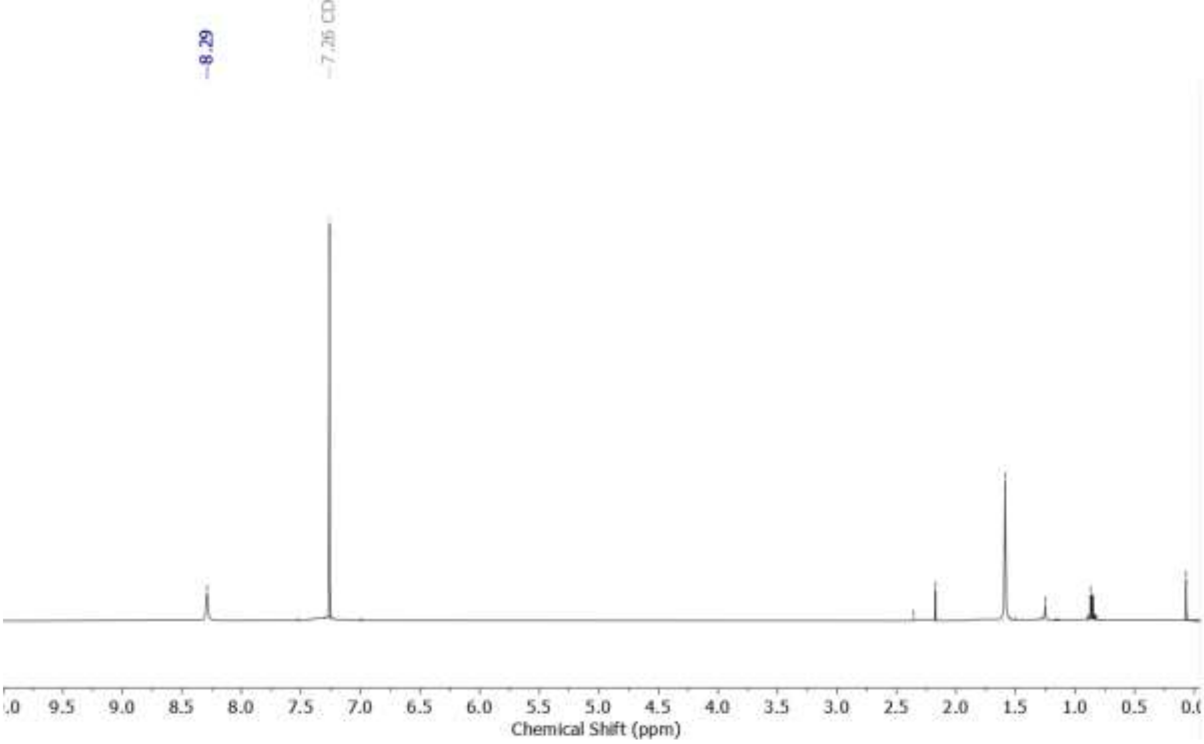

Figure S19: ^{1}H NMR spectrum of **1h** in $CDCl_3$

^{19}F in $CDCl_3$

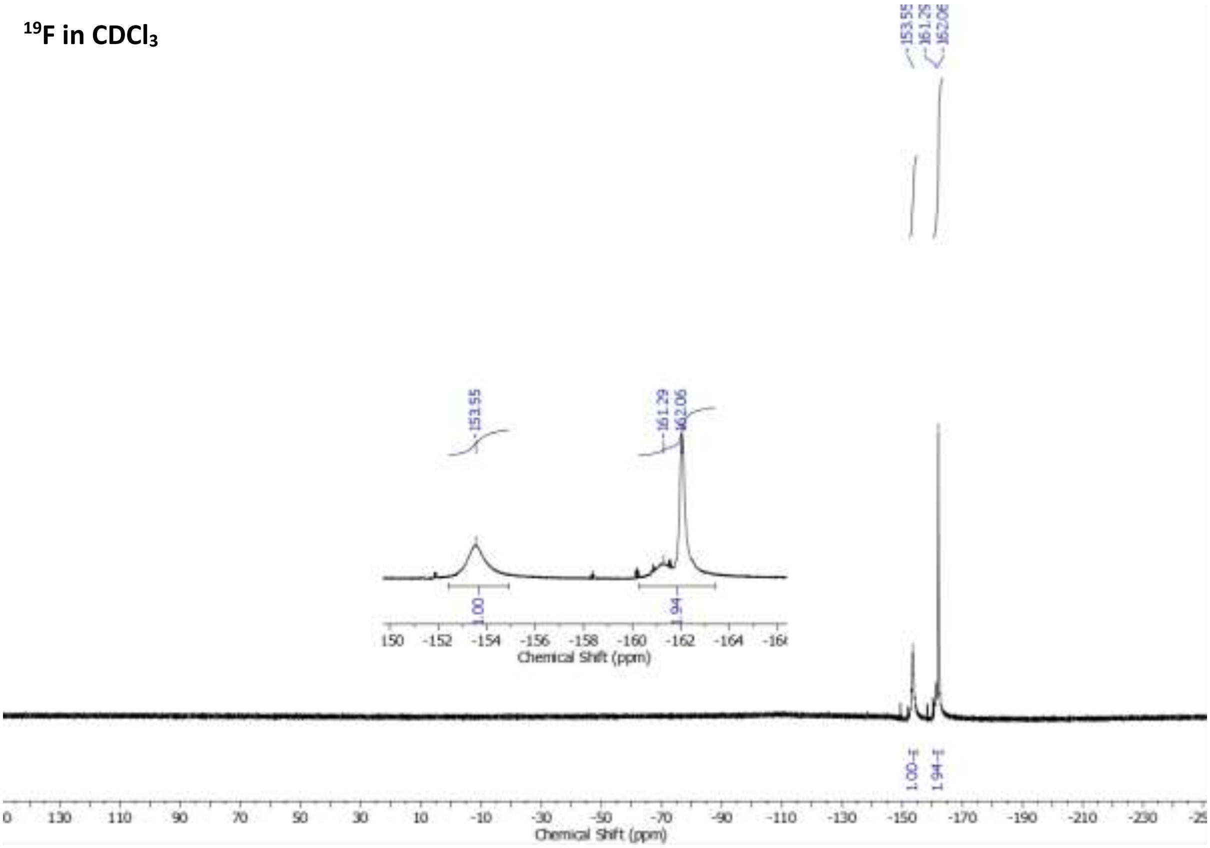

Figure S20: ^{1}H NMR spectrum of **1h** in $CDCl_3$

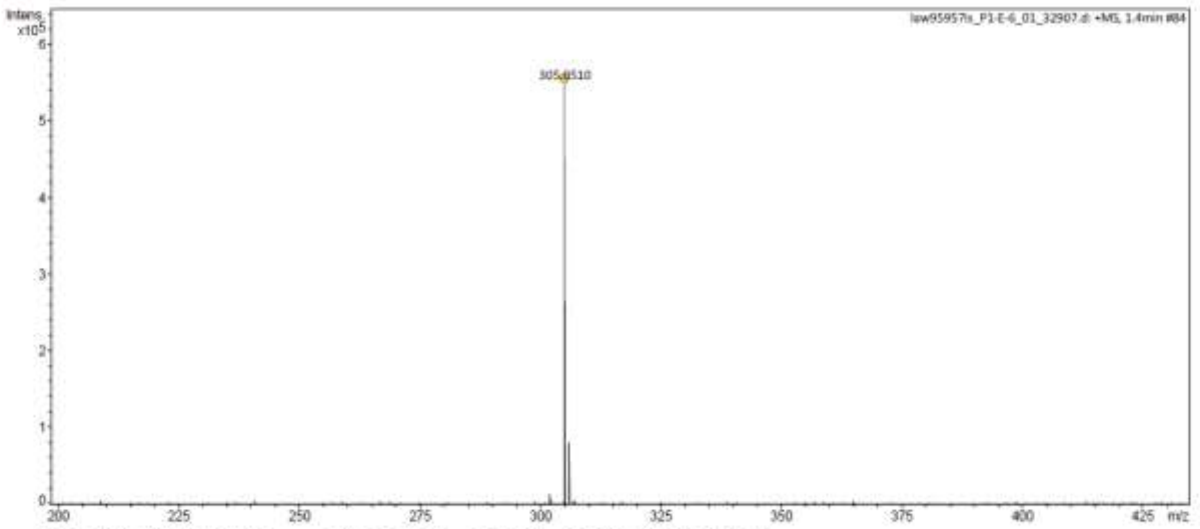

Meas. m/z	#	Ion Formula	m/z	err [ppm]	err [mDa]	mSigma	Mean err [ppm]
305.0510	1	C13H7F6N2	305.0508	-0.7	-0.2	1.2	-0.7

Figure S21: ESI mass spectrum of **1h**

Compound 1i

^1H in $(CD_3)_2CO$

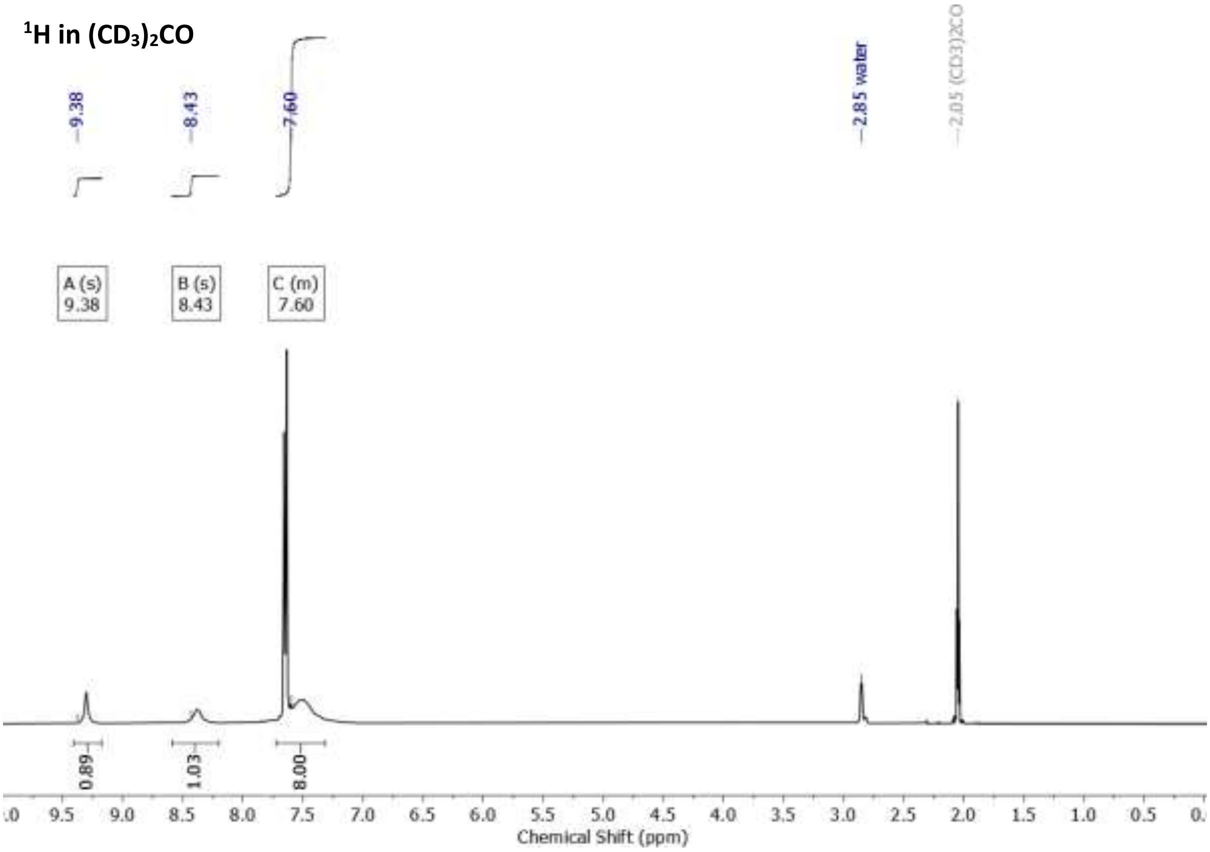

Figure S22: ^1H NMR spectrum of **1i** in $(CD_3)_2CO$

^{19}F in $(CD_3)_2CO$

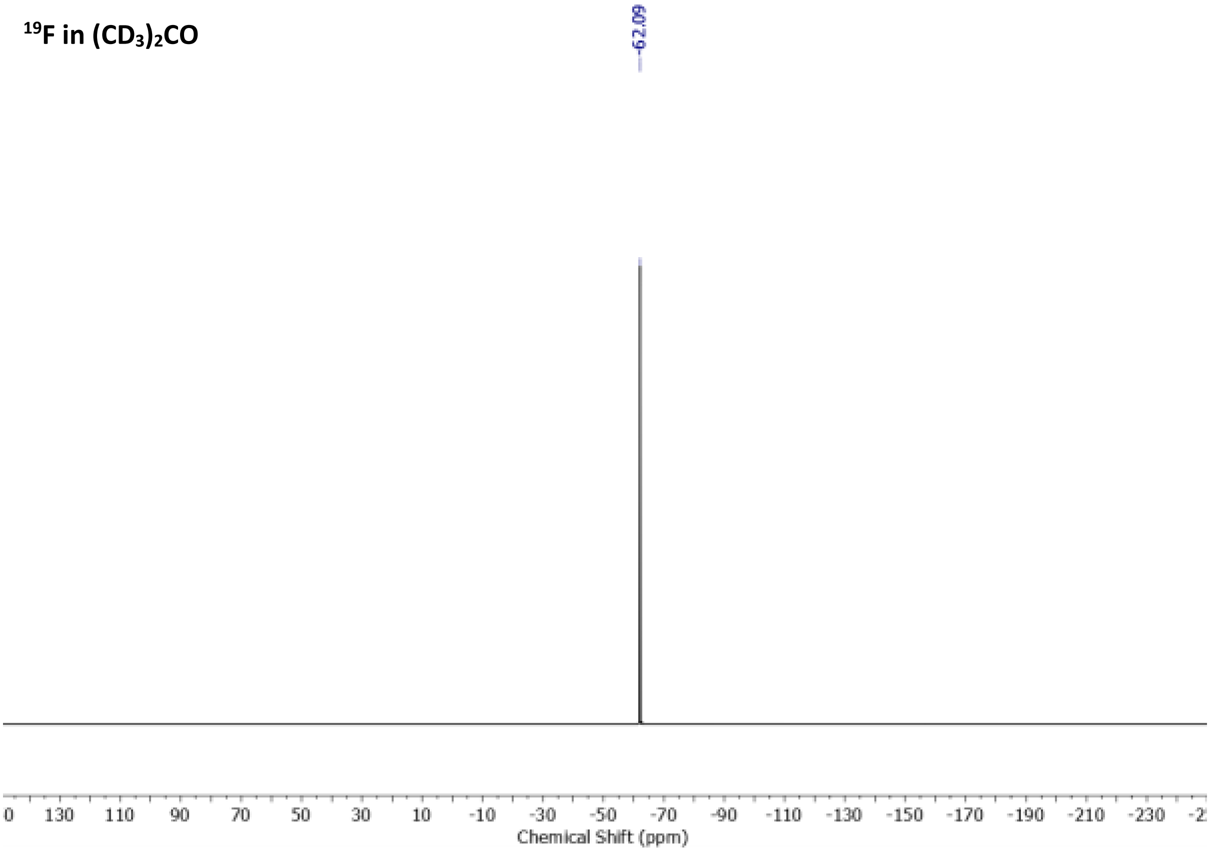

Figure S23: ^{19}F NMR spectrum of **1i** in $(CD_3)_2CO$

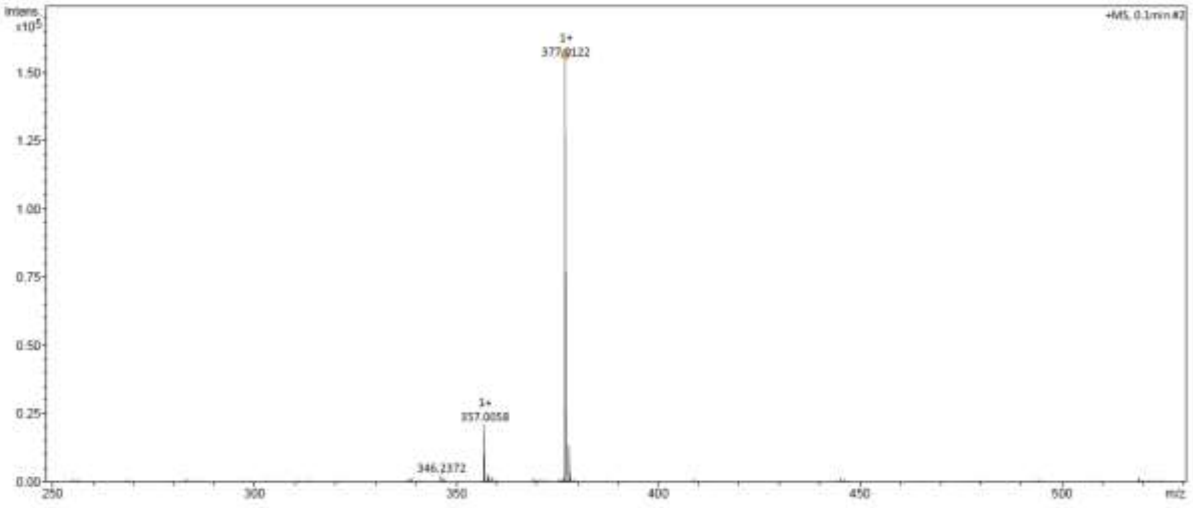

Meas. m/z	#	Ion Formula	m/z	err [ppm]	err [mDa]	mSigma	Mean err [ppm]
377.012176	1	C13H3F10N2	377.013107	-2.5	-0.9	35.4	2.2

Figure S24: ESI mass spectrum of **1i**

Compound 1j

^1H in $(CD_3)_2CO$

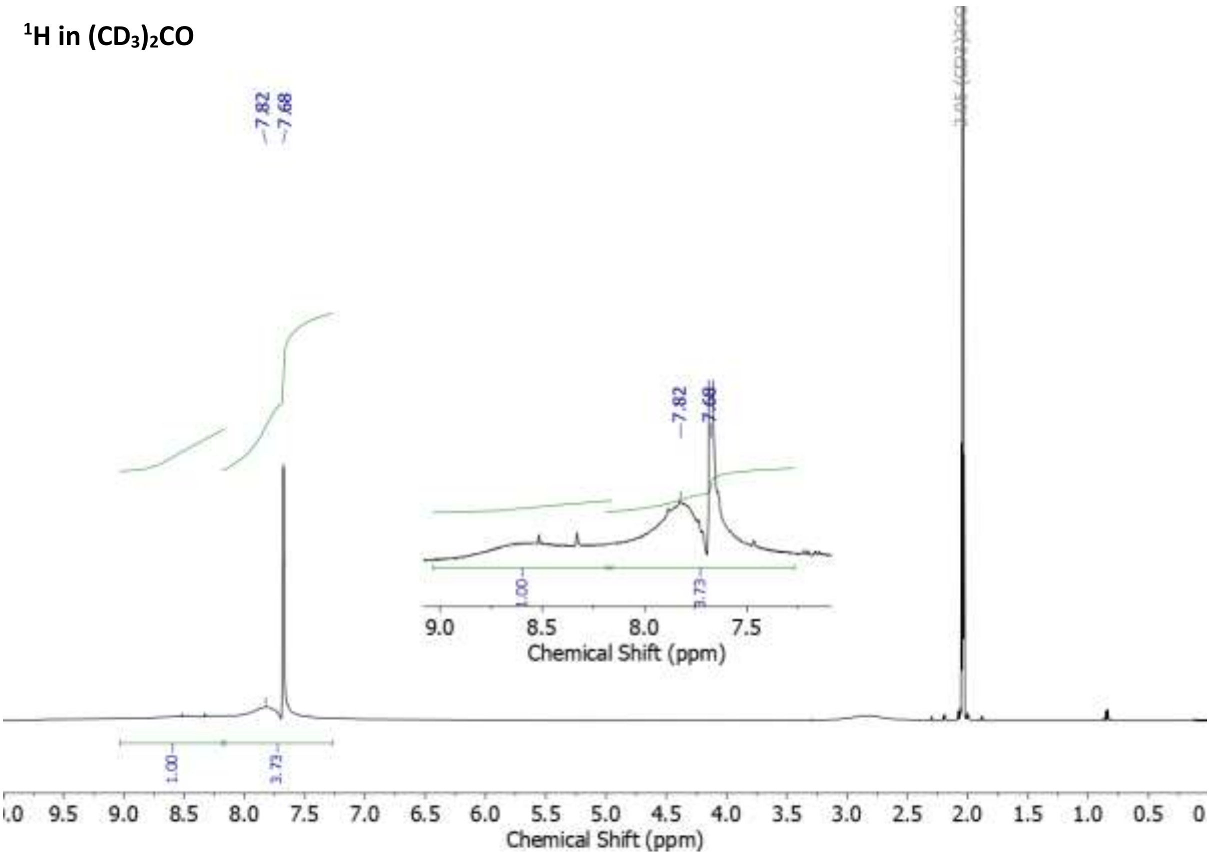

Figure S25: ^1H NMR spectrum of **1j** in $(CD_3)_2CO$

^{19}F in $(CD_3)_2CO$

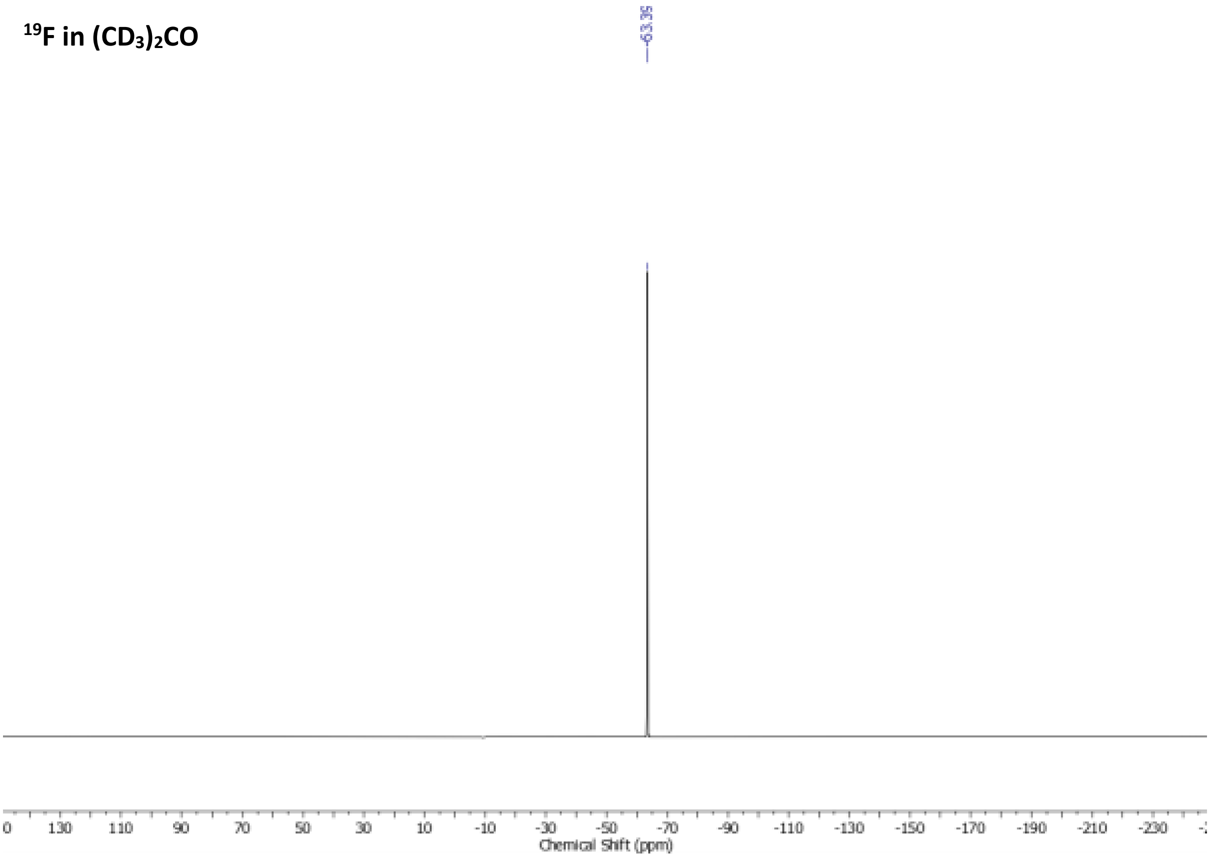

Figure S26: ^{19}F NMR spectrum of **1j** in $(CD_3)_2CO$

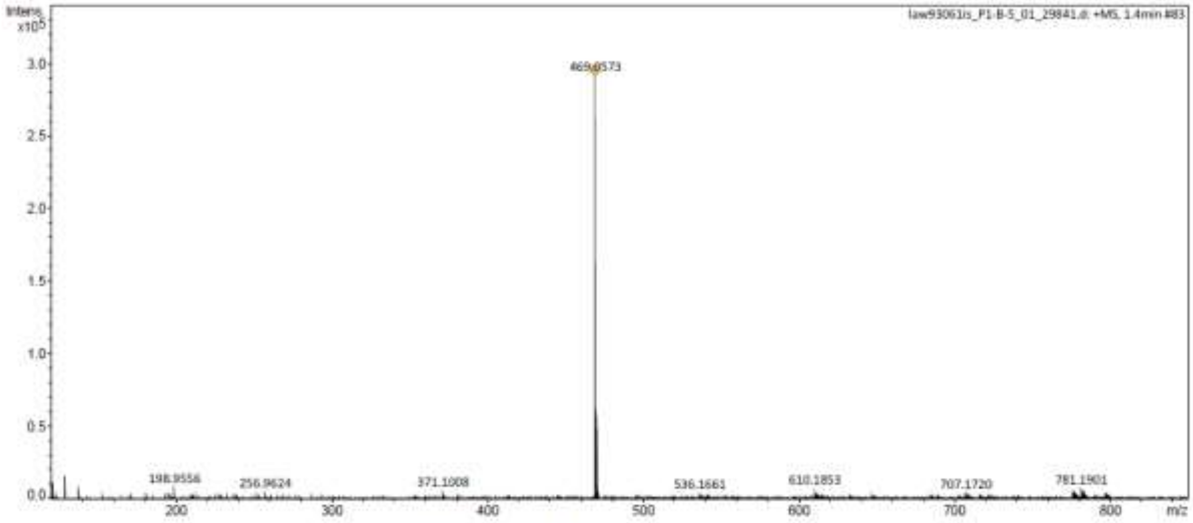

Meas. m/z	#	Ion Formula	m/z	err [ppm]	err [mDa]	mSigma	Mean err [ppm]
469.0573	1	C17H9F12N2	469.0569	-0.9	-0.4	1.7	-1.1

Figure S27: ESI mass spectrum of **1j** in $(CD_3)_2CO$

Compound 2b

^{1}H in $(CD_3)_2CO$

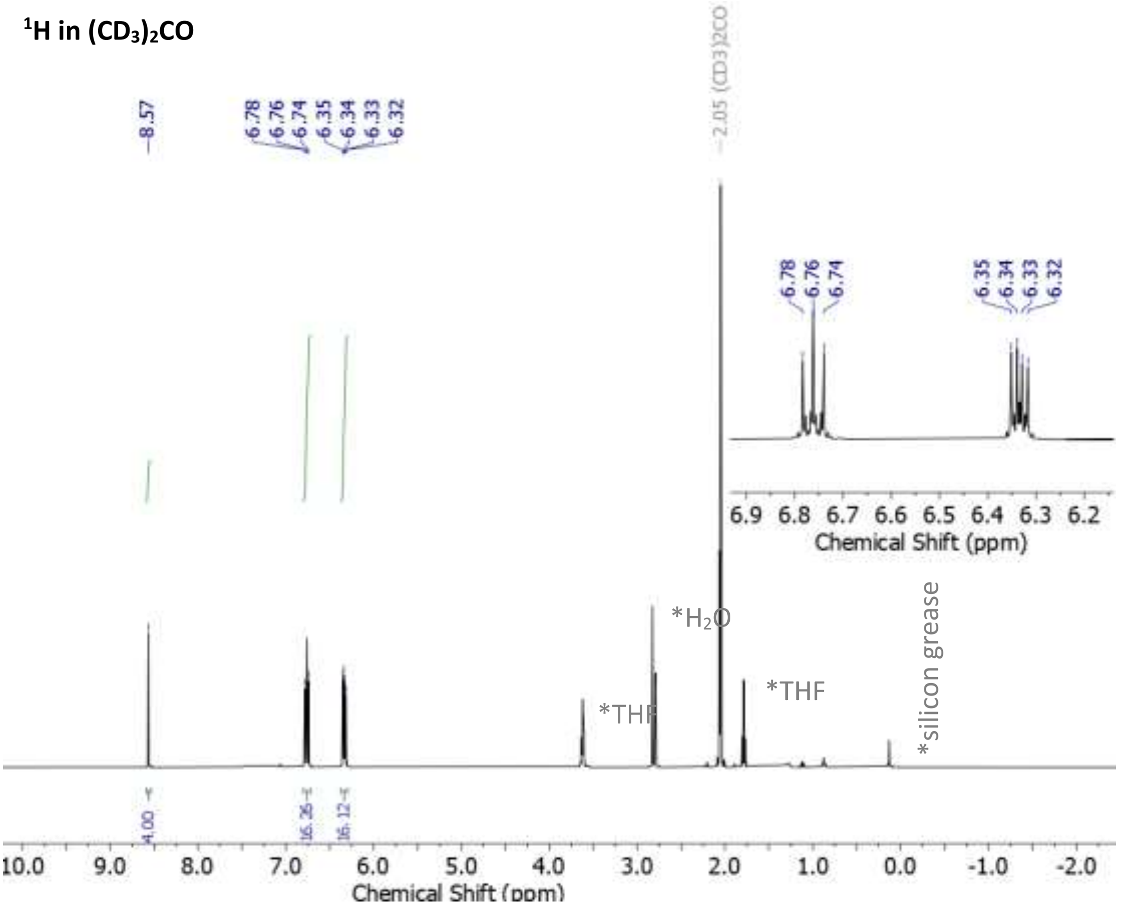

Figure S28: ^{1}H NMR spectrum of **2b** in $(CD_3)_2CO$

^{19}F in $(CD_3)_2CO$

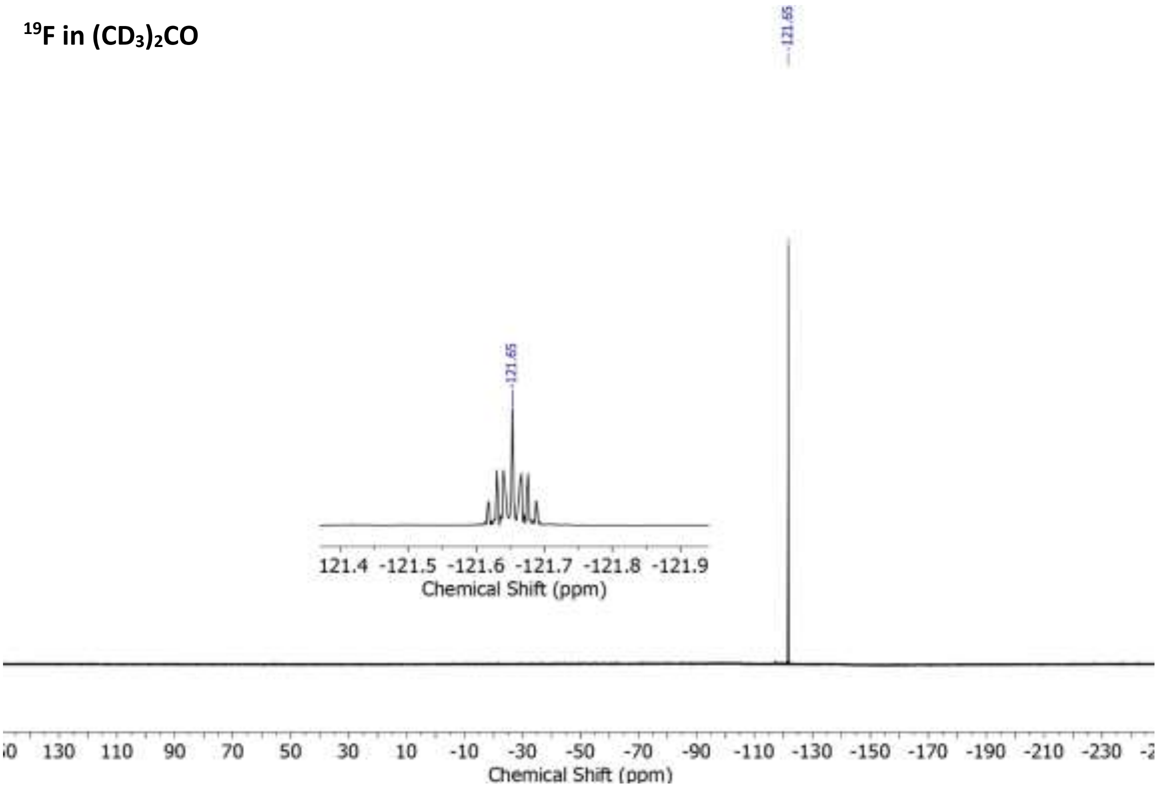

Figure S29: ^{1}H NMR spectrum of **2b** in $(CD_3)_2CO$

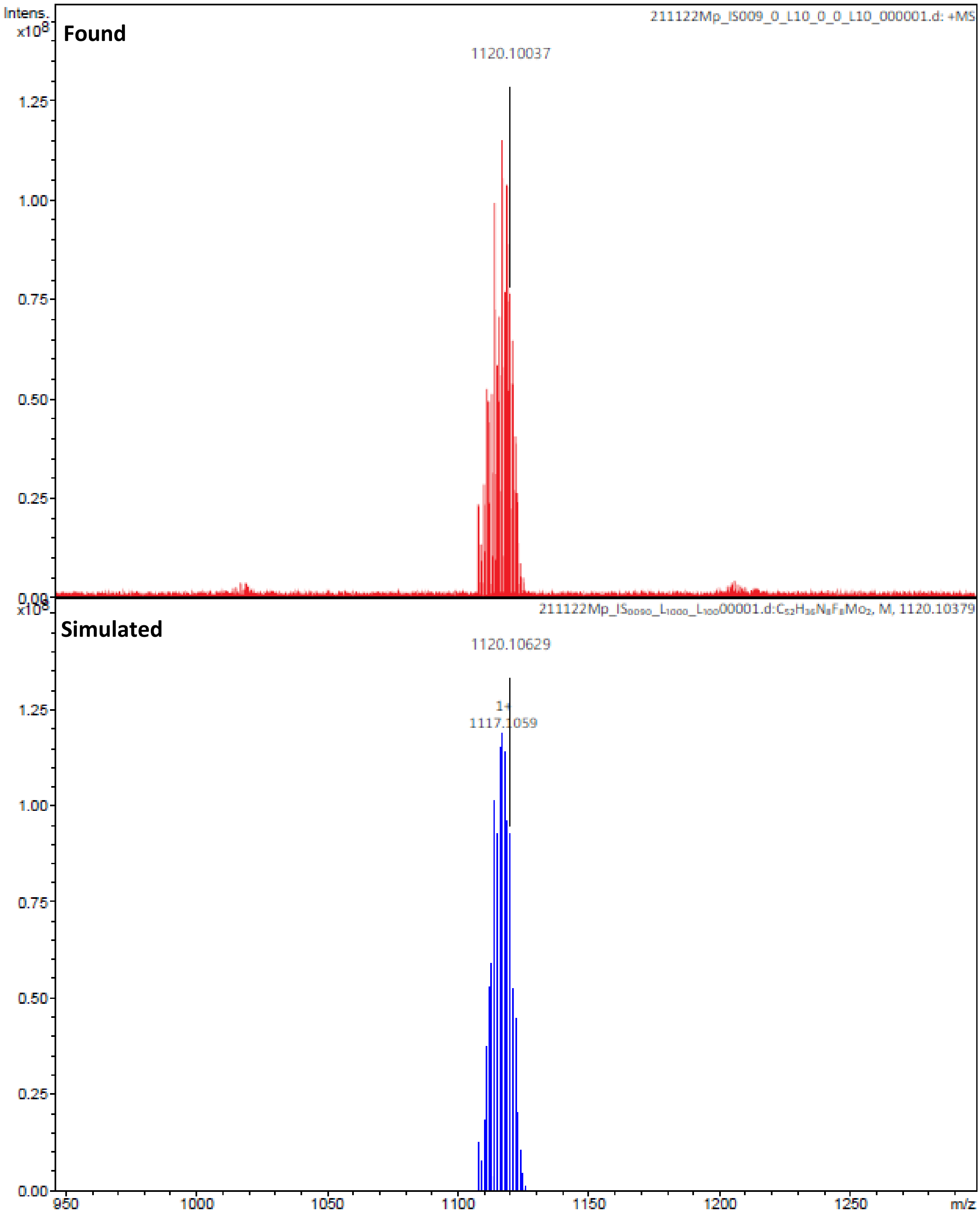

Figure S30: MALDI-TOF mass spectrum of **2b** with dithranol matrix. Top panel is experimental data and bottom panel is the predicted spectrum

Compound 2c

^{1}H in $(CD_3)_2CO$

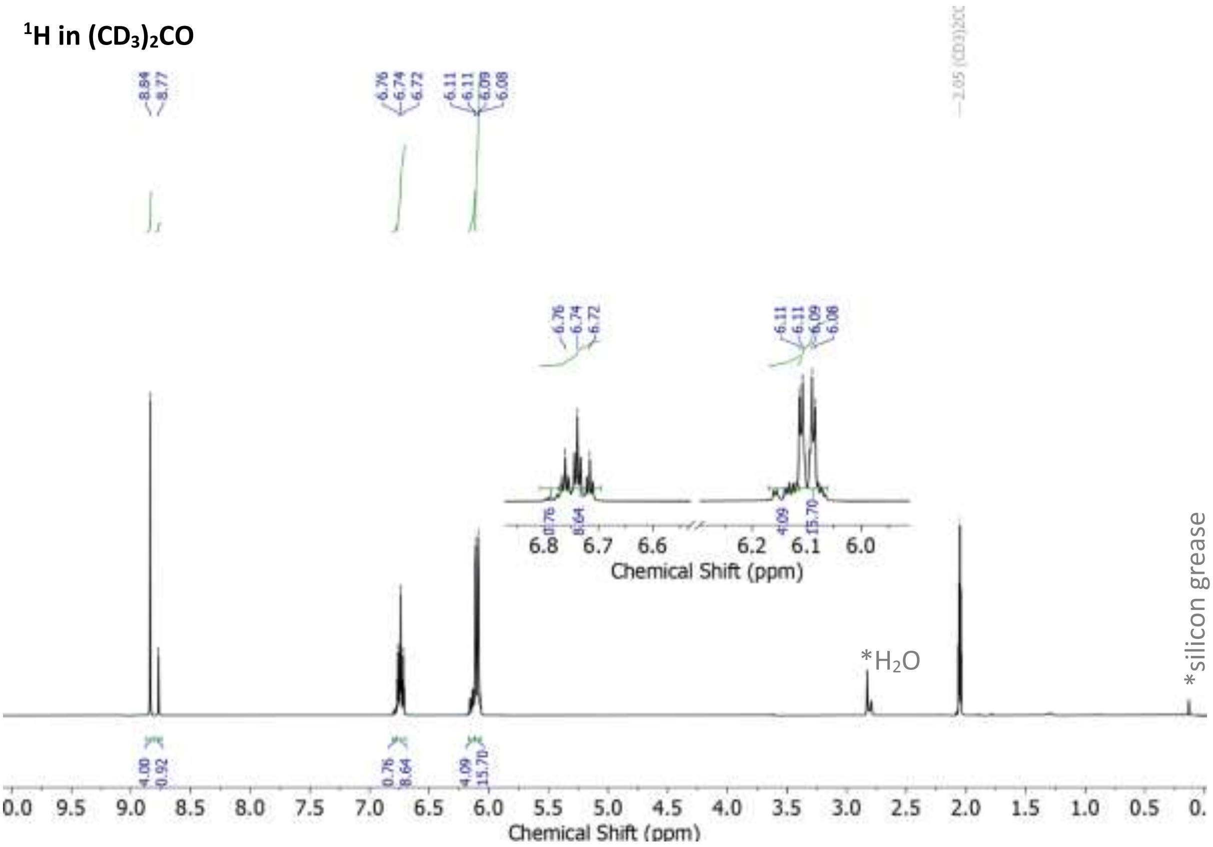

Figure S31: ^{1}H NMR spectrum of **2c** in $(CD_3)_2CO$

^{19}F in $(CD_3)_2CO$

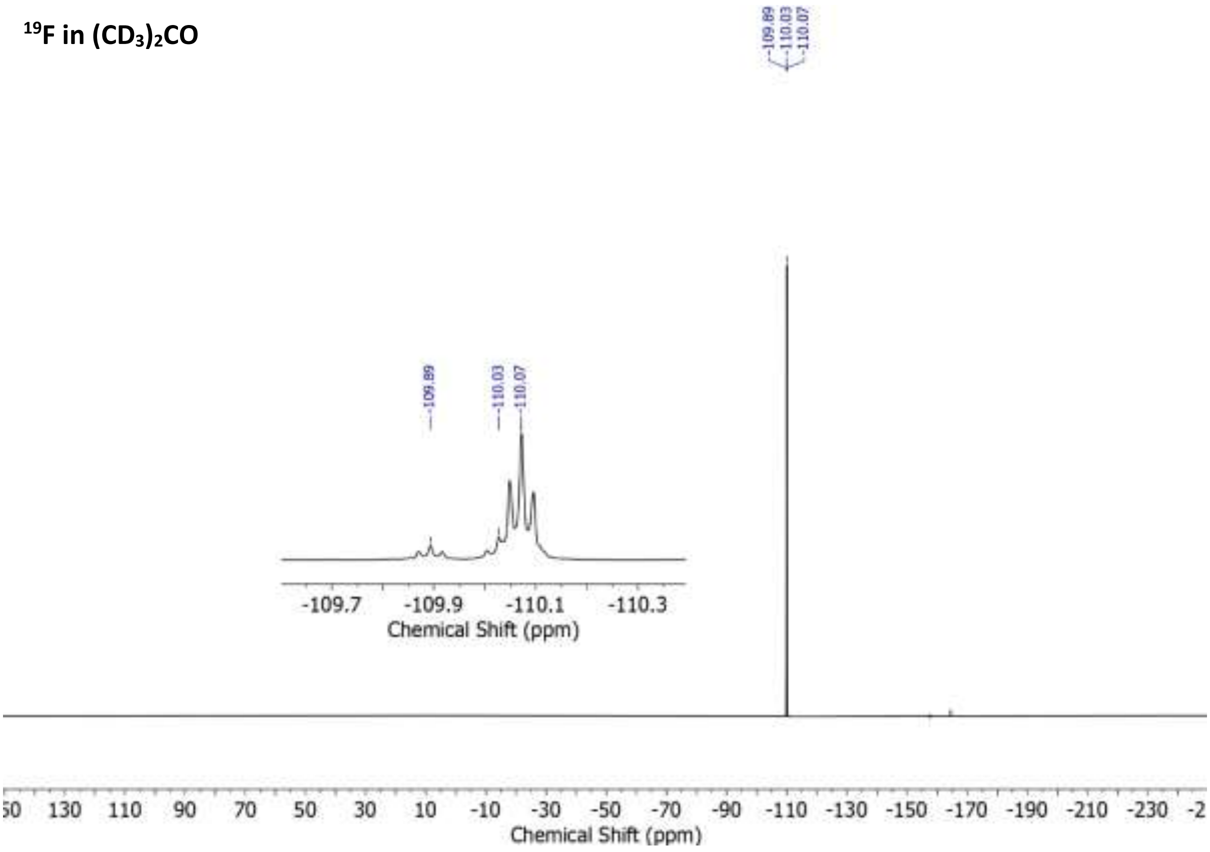

Figure S32: ^{19}F NMR spectrum of **2c** in $(CD_3)_2CO$

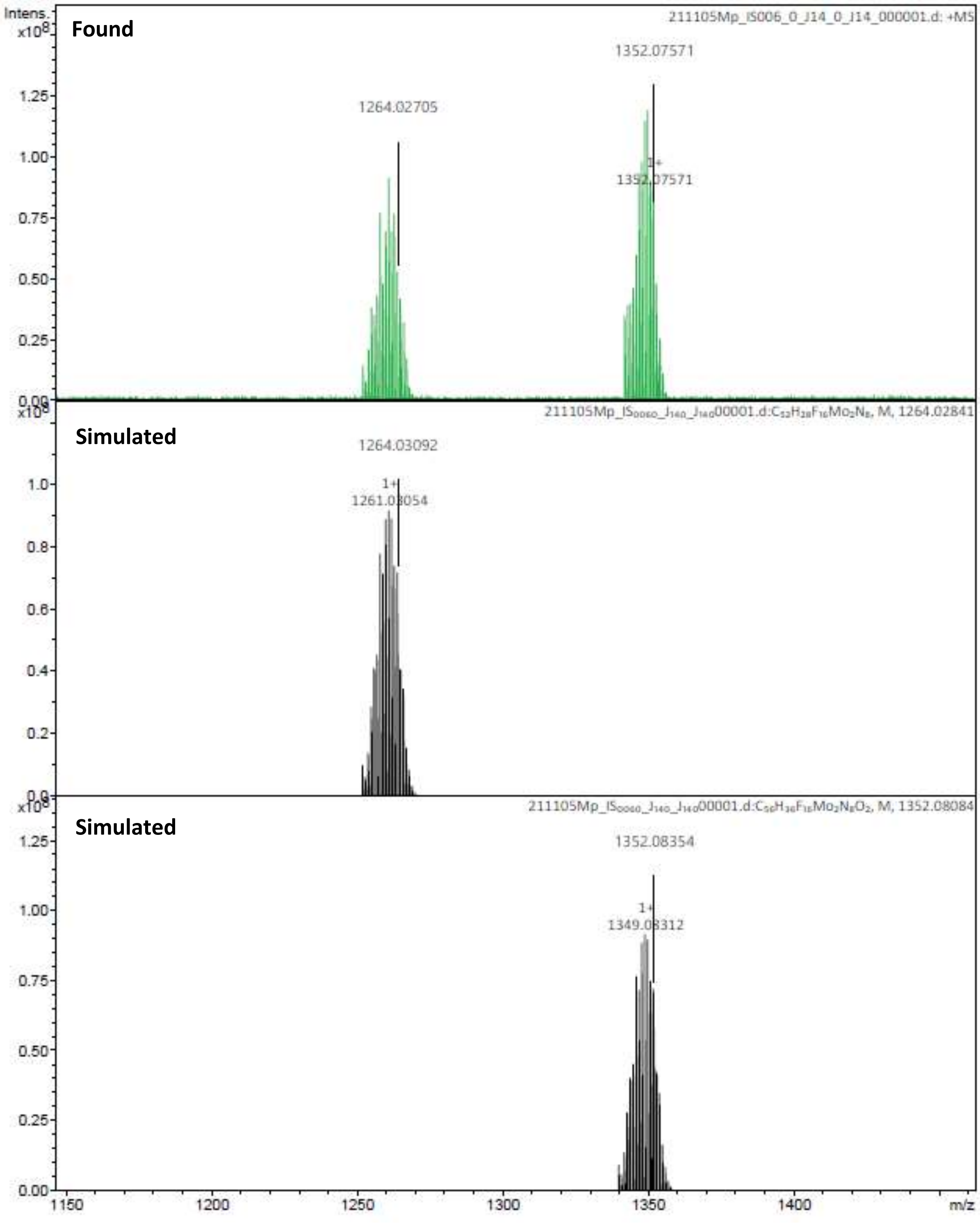

Figure S33: MALDI-TOF mass spectrum of **2c** with dithranol matrix. Top panel is experimental data and bottom two panels are the predicted spectra

Compound 2d

^{1}H in $(CD_3)_2CO$

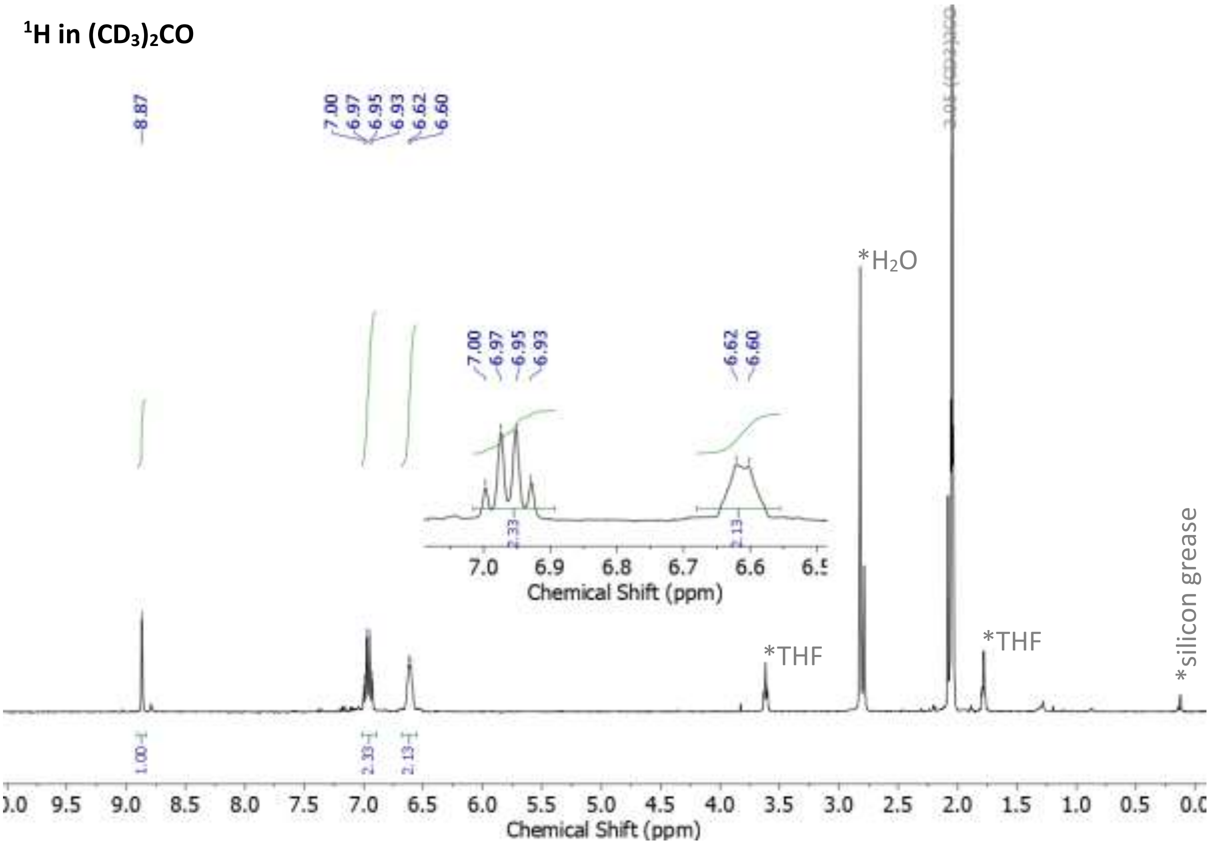

Figure S34: ^{1}H NMR spectrum of **2d** in $(CD_3)_2CO$

^{19}F in $(CD_3)_2CO$

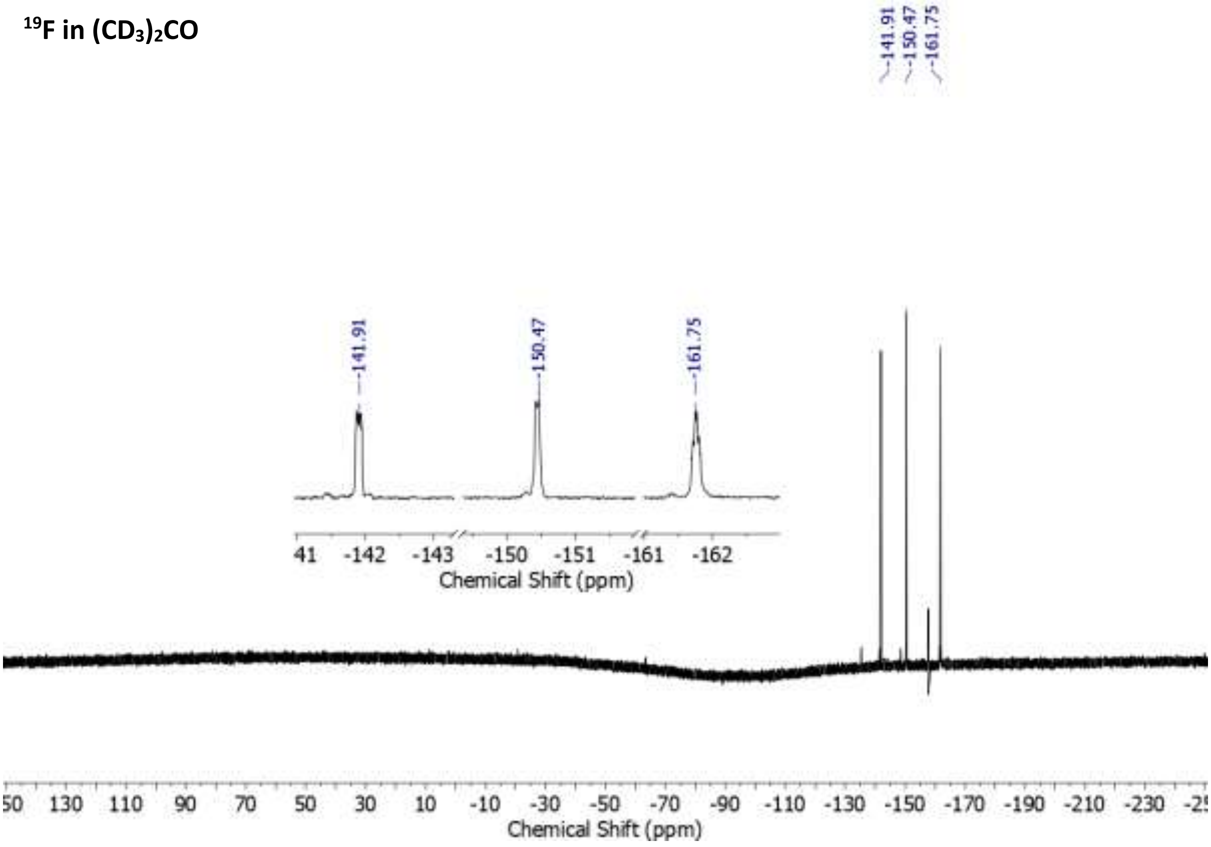

Figure S35: ^{19}F NMR spectrum of **2d** in $(CD_3)_2CO$

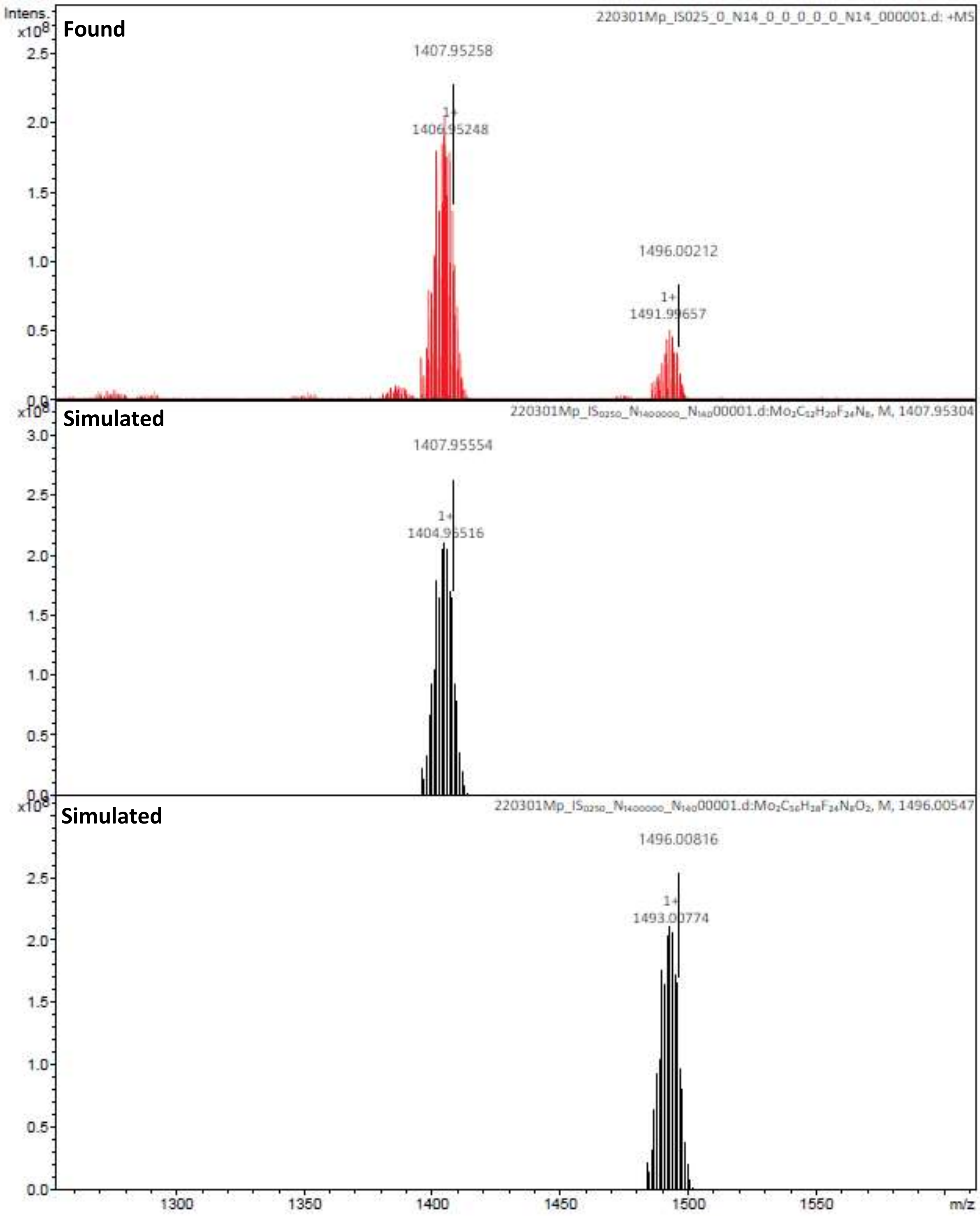

Figure S36: MALDI-TOF mass spectrum of **2d** with dithranol matrix. Top panel is experimental data and bottom two panels are the predicted spectra

Compound 2e

^{1}H in $(CD_3)_2CO$

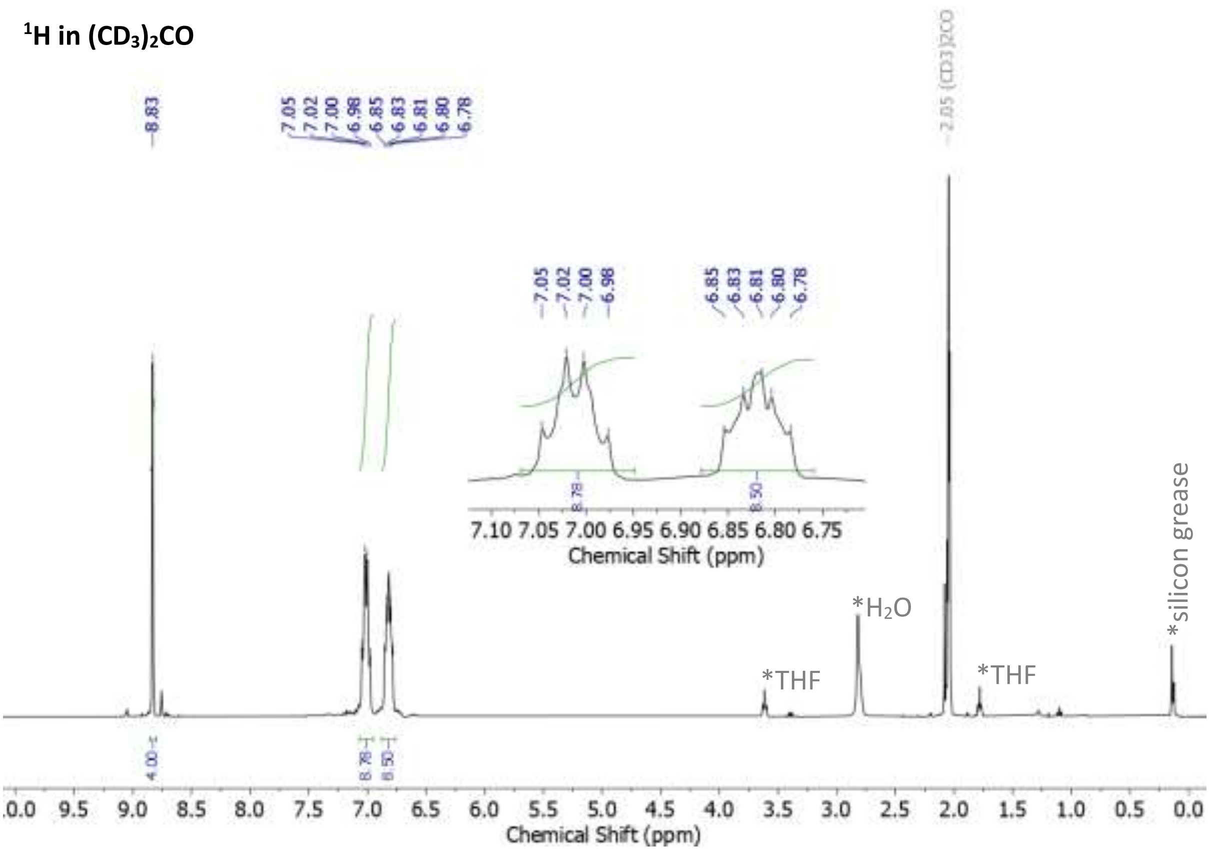

Figure S37: ^{1}H NMR spectrum of **2e** in $(CD_3)_2CO$

^{19}F in $(CD_3)_2CO$

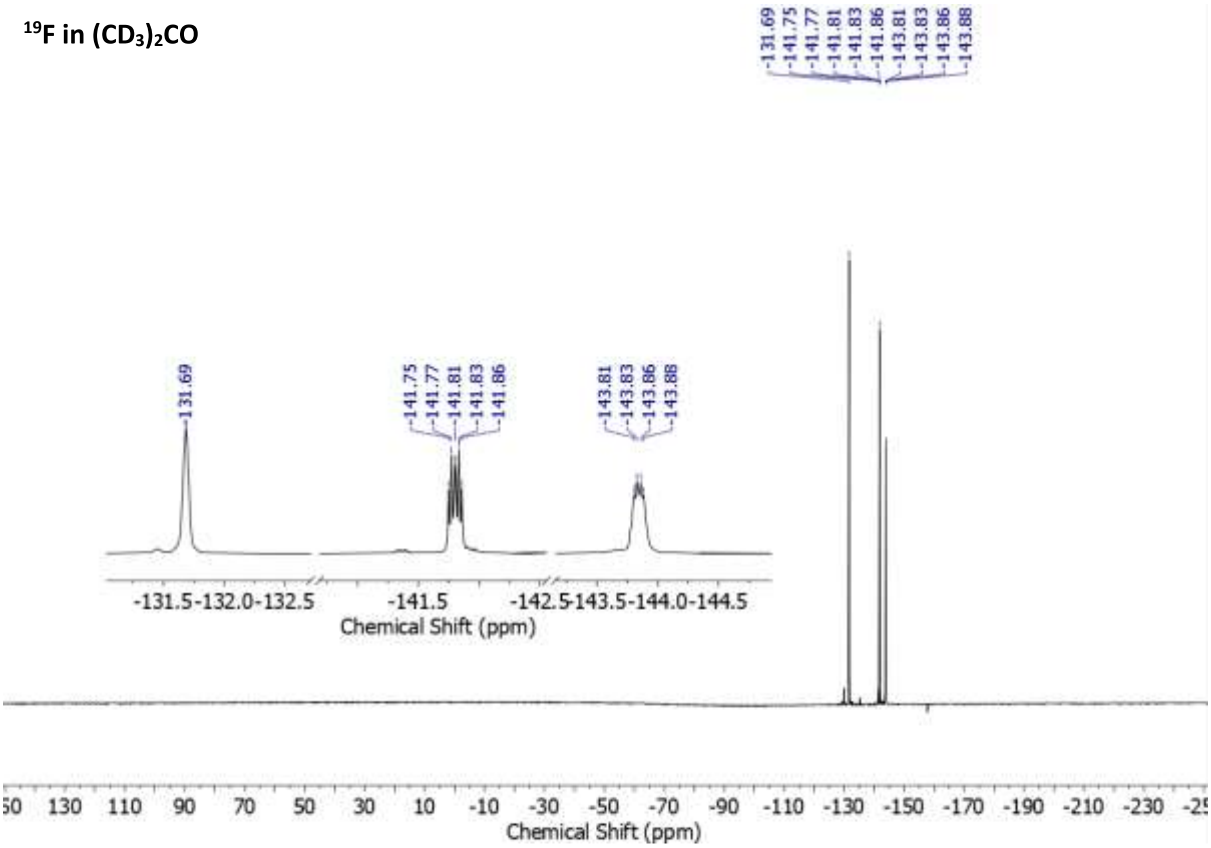

Figure S38: ^{19}F NMR spectrum of **2e** in $(CD_3)_2CO$

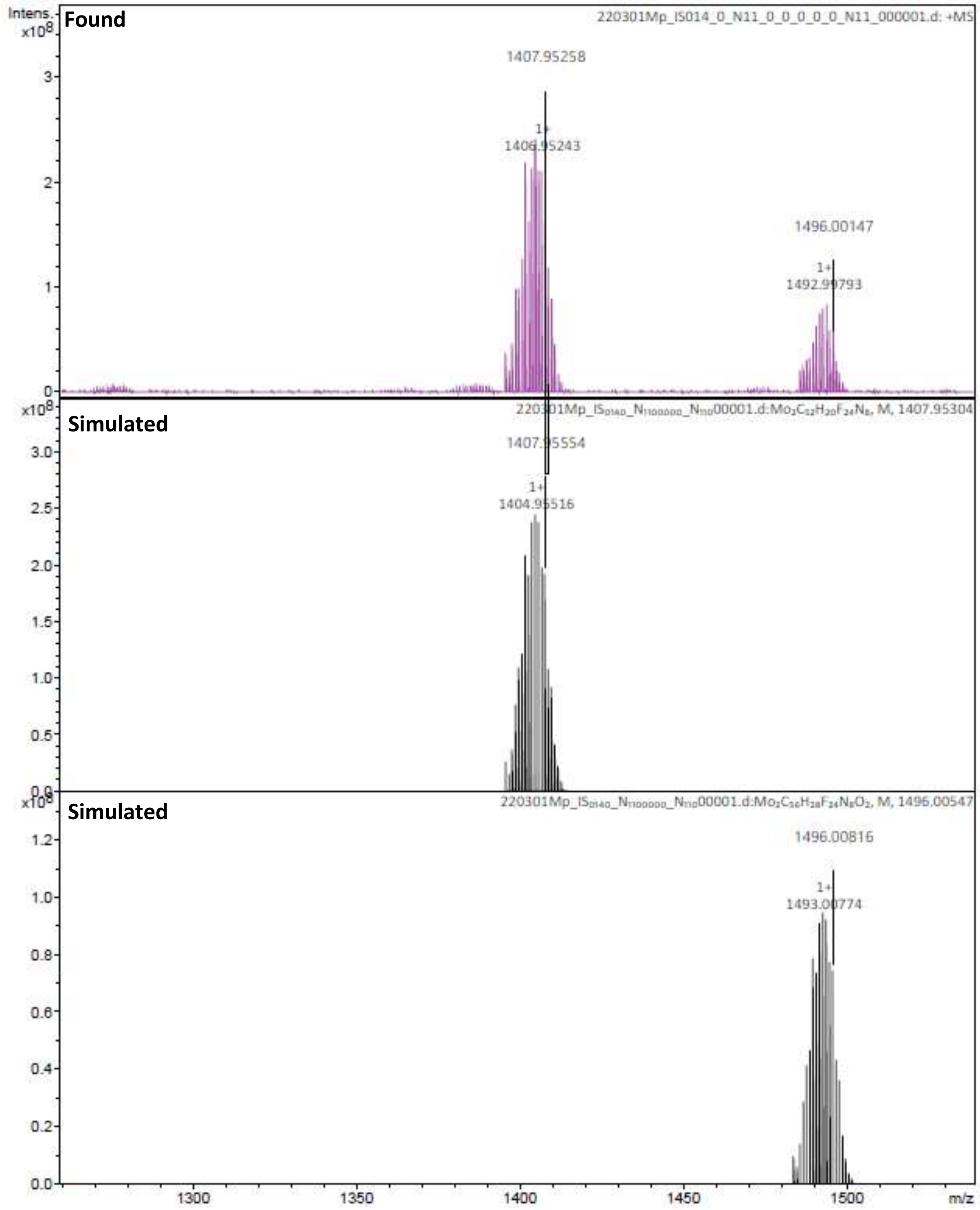

Figure S39: MALDI-TOF mass spectrum of **2e** with dithranol matrix. Top panel is experimental data and bottom two panels are the predicted spectra

Compound 2f

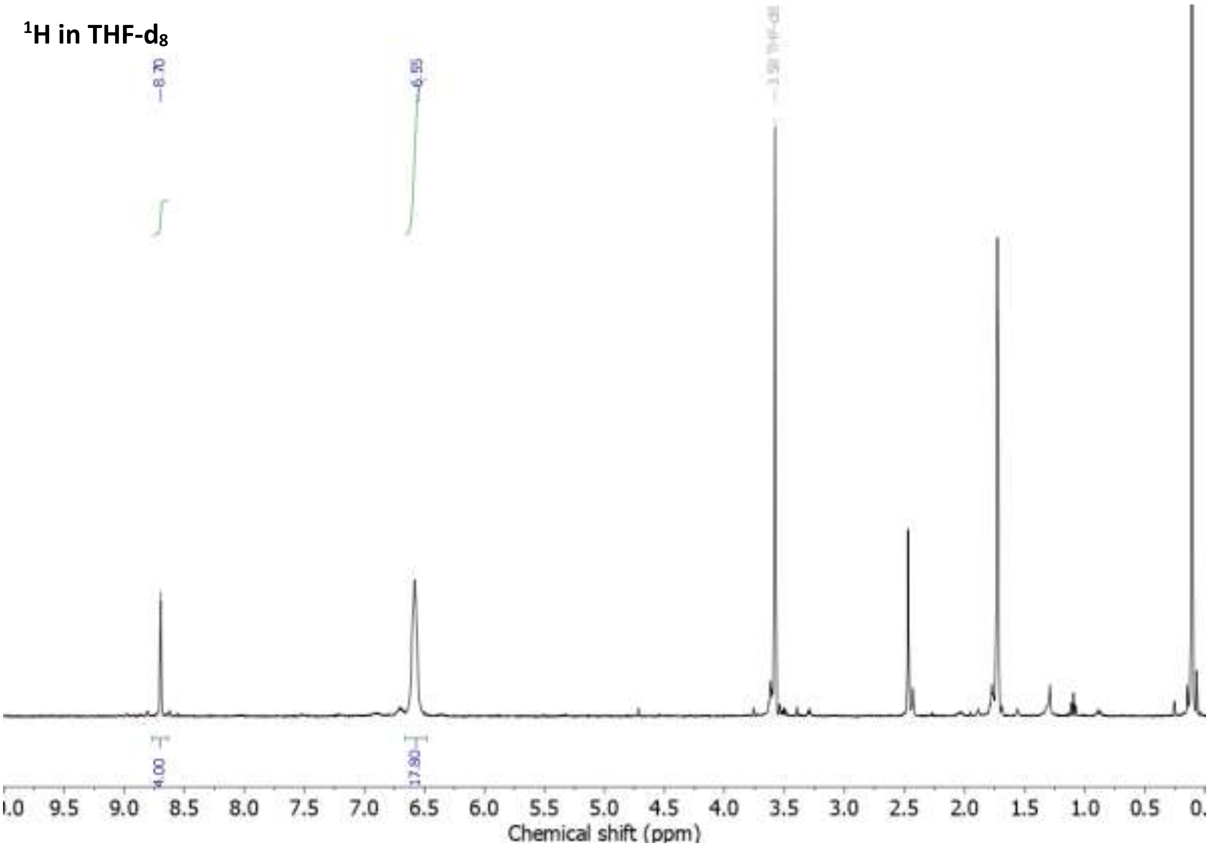

Figure S40: ^{1}H NMR spectrum of **2f** in THF-d$_8$

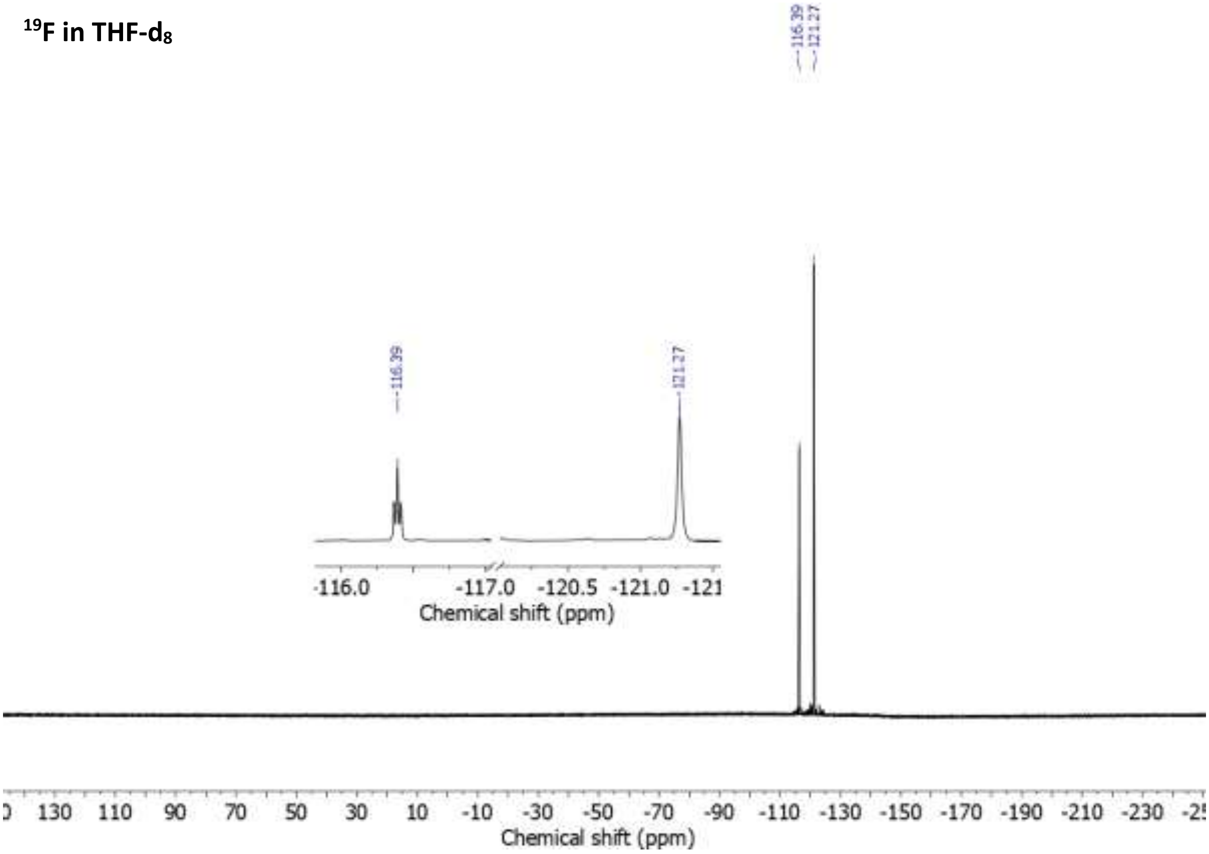

Figure S41: ^{19}F NMR spectrum of **2f** in THF-d$_8$

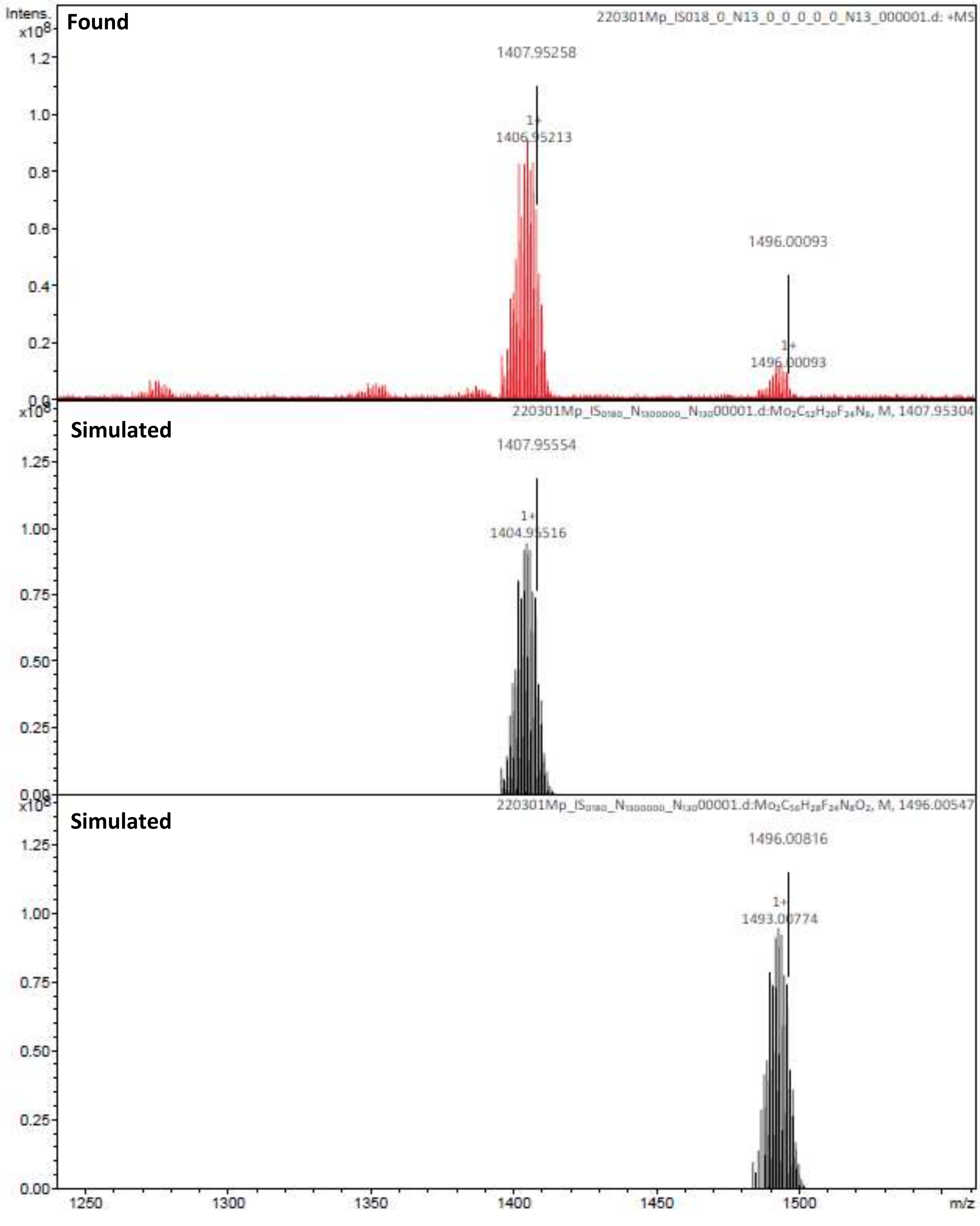

Figure S42: MALDI-TOF mass spectrum of **2f** with dithranol matrix. Top panel is experimental data and bottom two panels are the predicted spectra

Compound 2g

^{1}H in $(CD_3)_2CO$

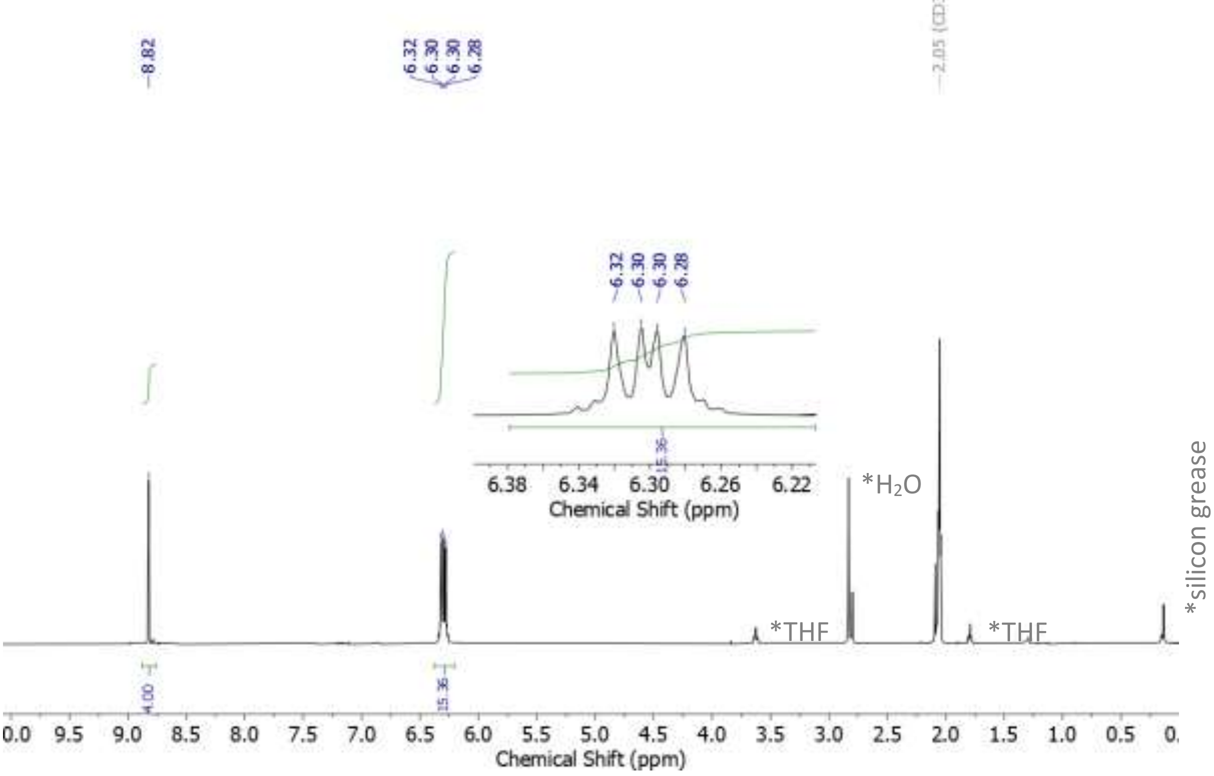

Figure S43: ^{1}H NMR spectrum of **2g** in $(CD_3)_2CO$

^{19}F in $(CD_3)_2CO$

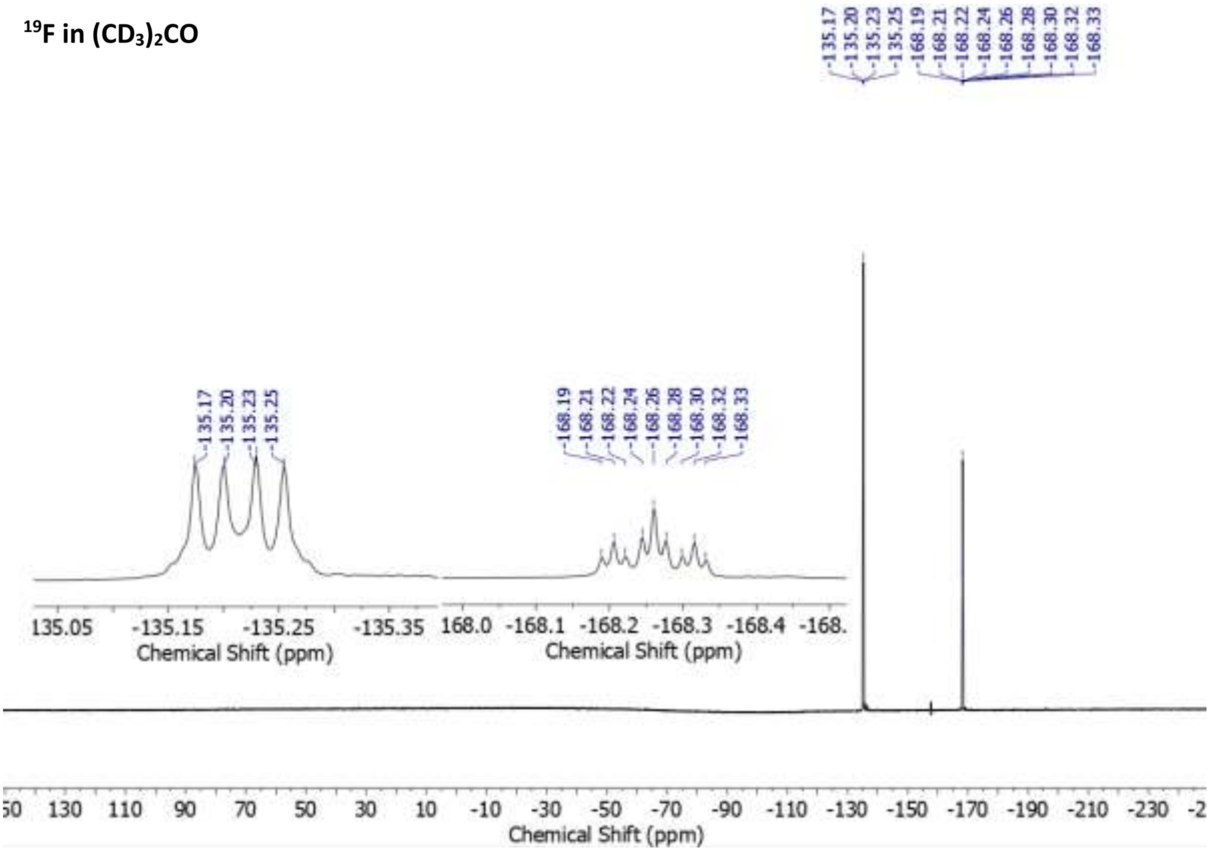

Figure S44: ^{19}F NMR spectrum of **2g** in $(CD_3)_2CO$

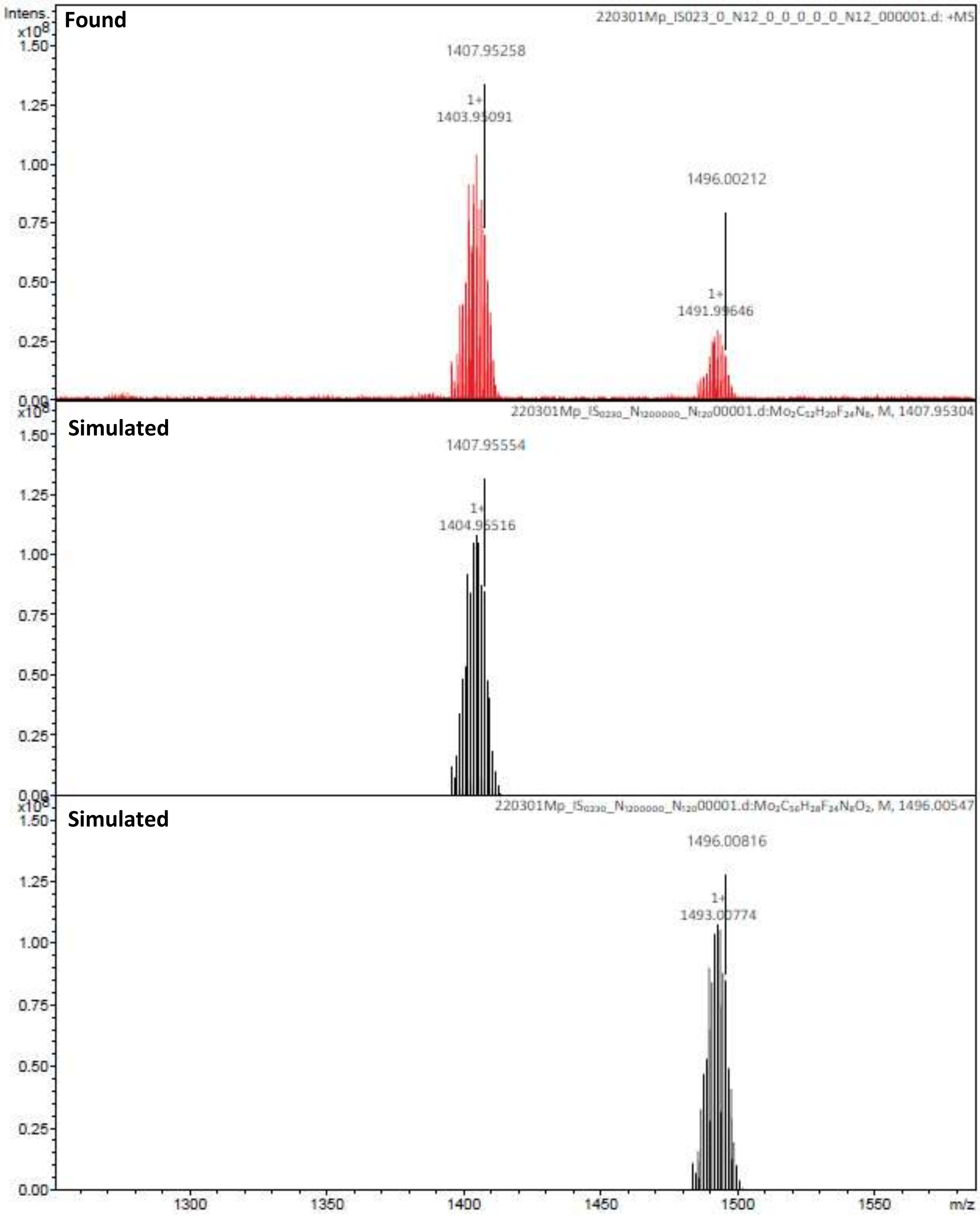

Figure S45: MALDI-TOF mass spectrum of **2g** with dithranol matrix. Top panel is experimental data and bottom two panels are the predicted spectra

Compound 2h

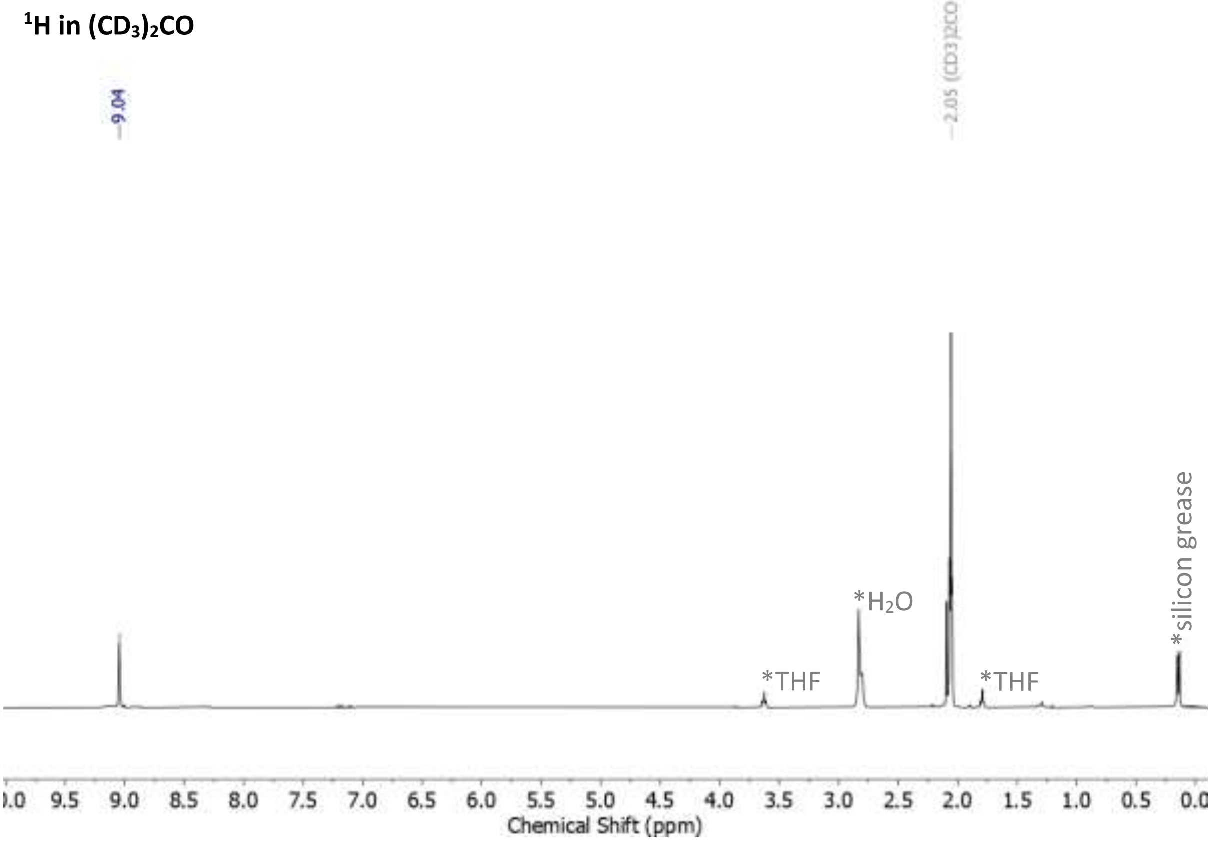

Figure S46: ^{1}H NMR spectrum of **2h** in $(CD_3)_2CO$

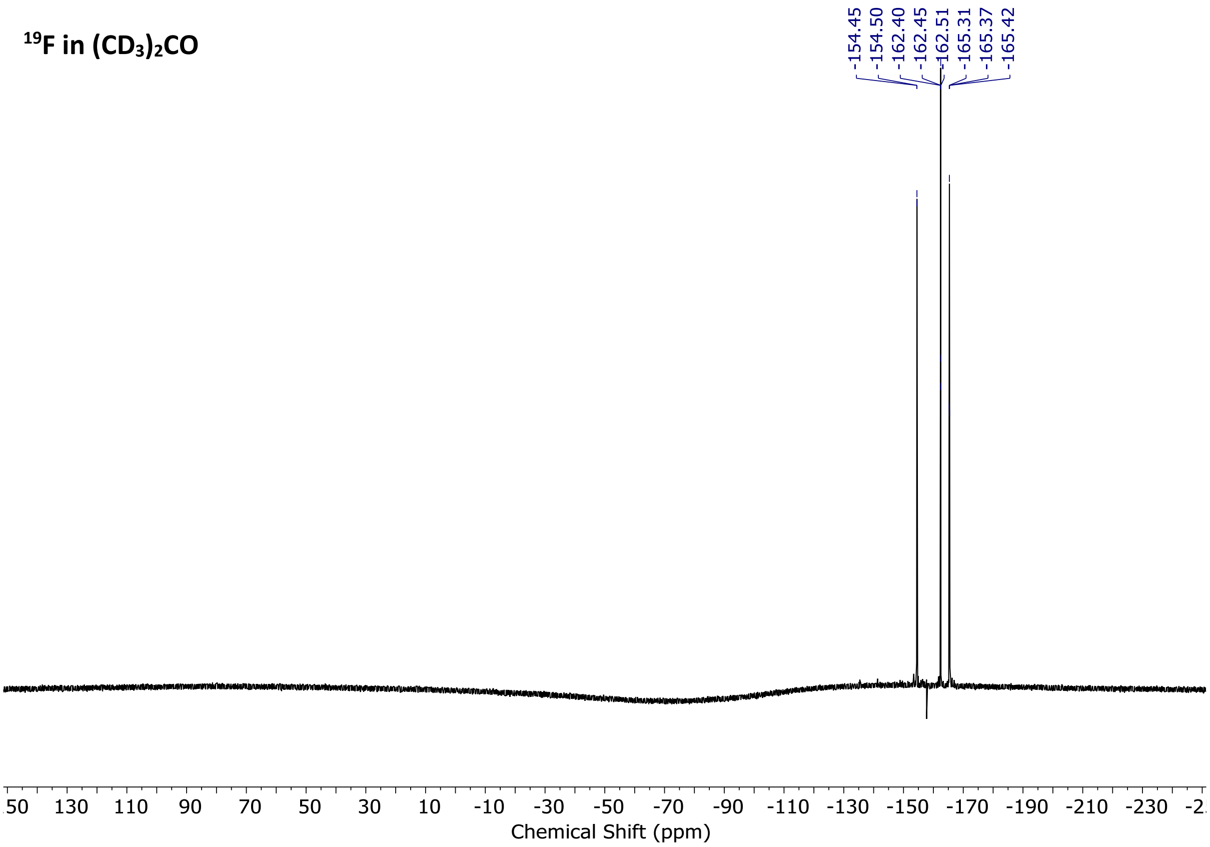

Figure S47: ^{19}F NMR spectrum of **2h** in $(CD_3)_2CO$

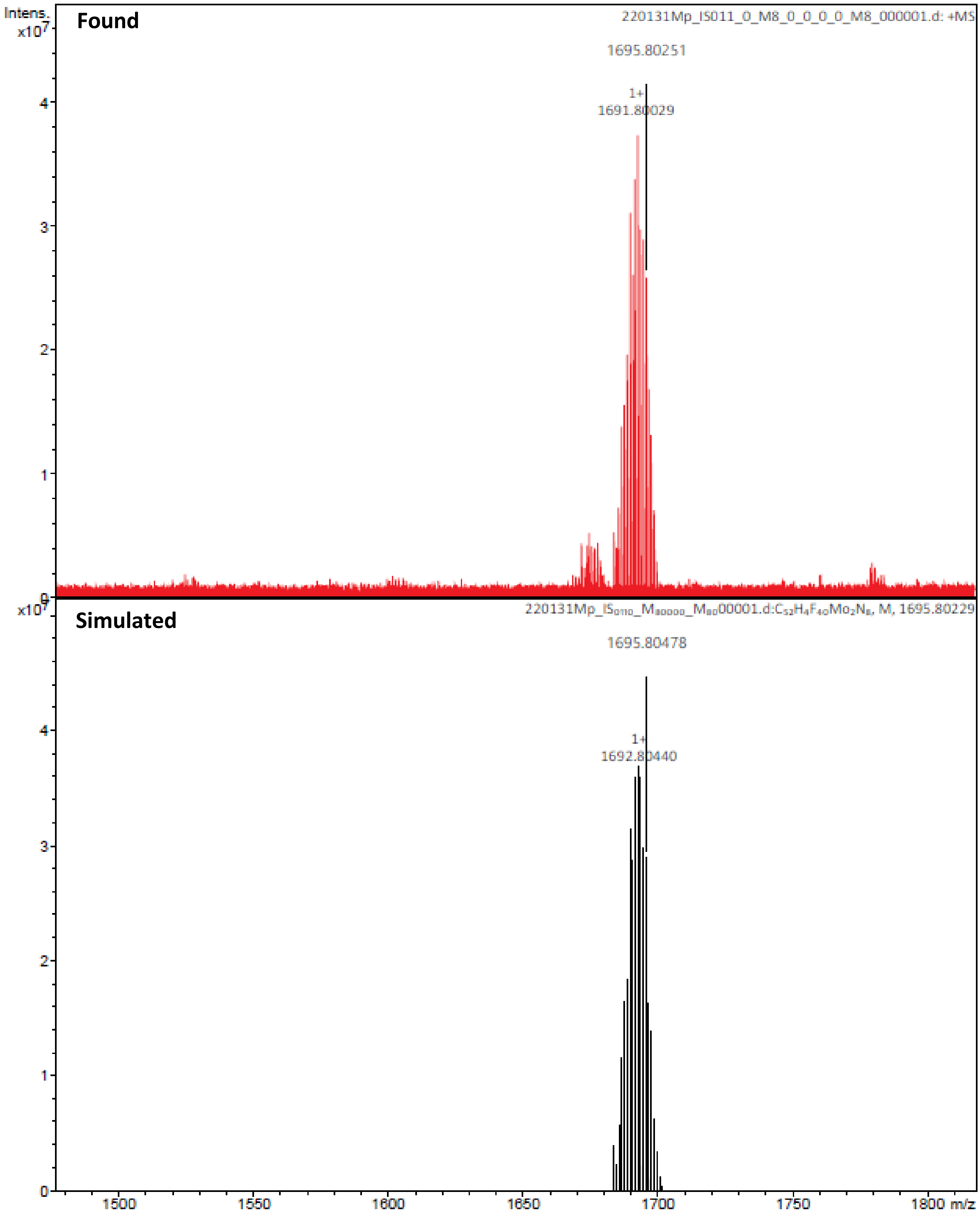

Figure S48: MALDI-TOF mass spectrum of **2h** with dithranol matrix. Top panel is experimental data and bottom panel is the predicted spectra

Compound 2i

^{1}H in $(CD_3)_2CO$

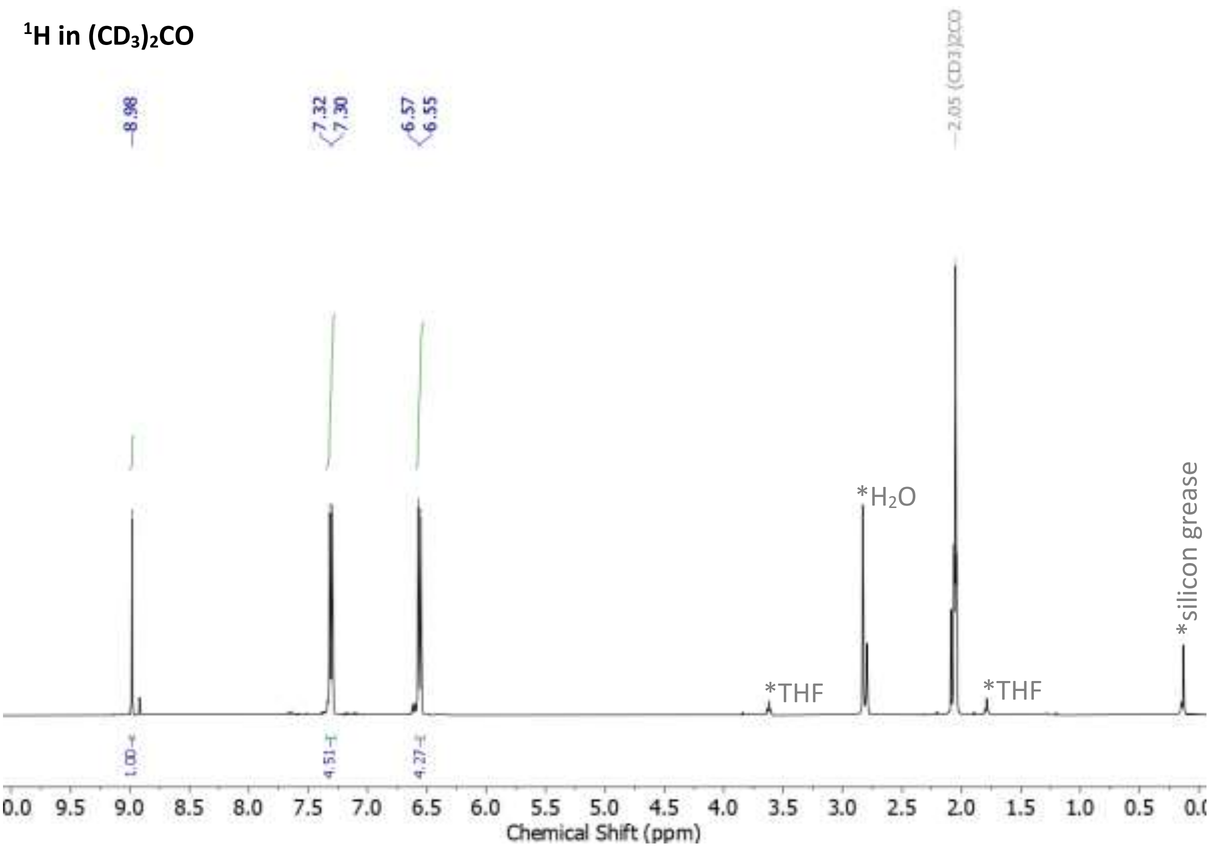

Figure S49: ^{1}H NMR spectrum of **2i** in $(CD_3)_2CO$

^{19}F in $(CD_3)_2CO$

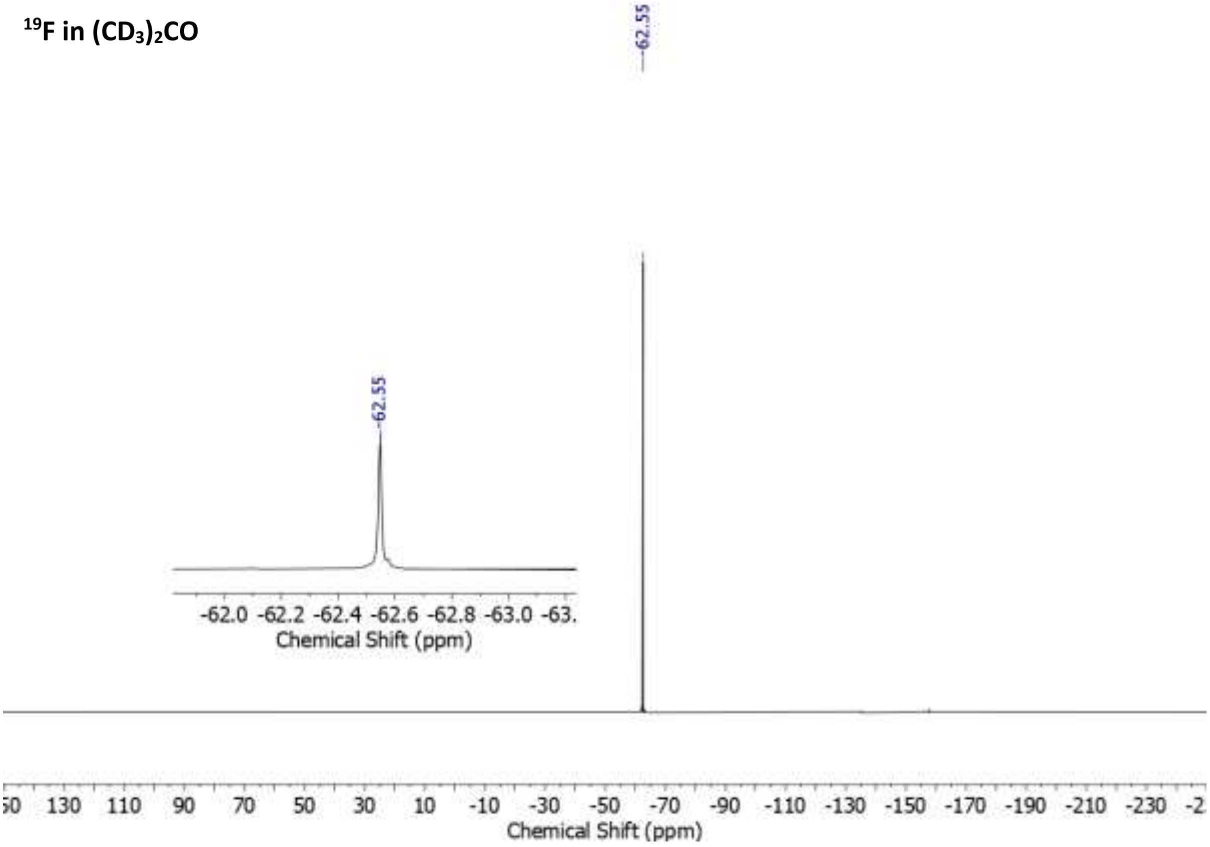

Figure S50: ^{1}H NMR spectrum of **2i** in $(CD_3)_2CO$

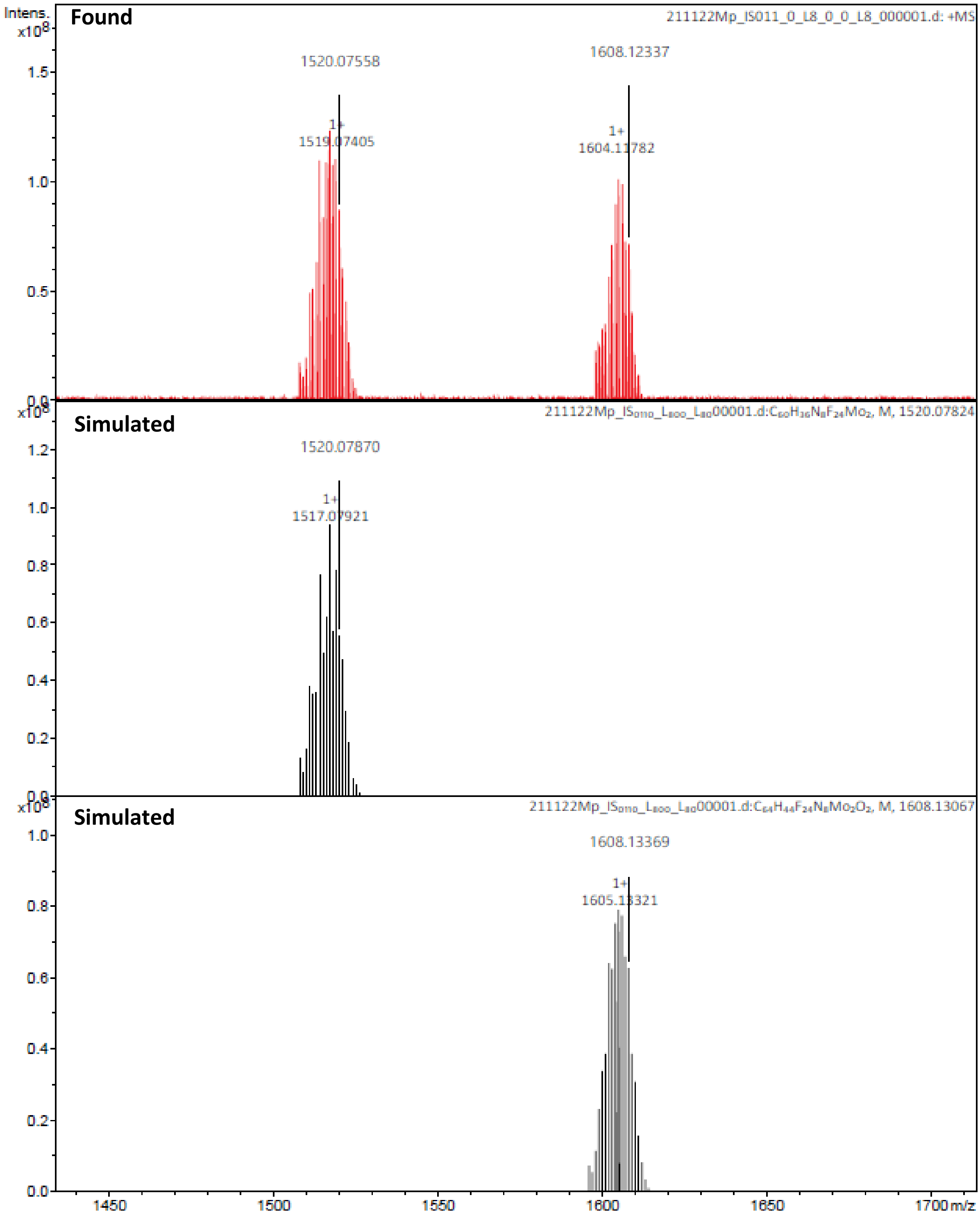

Figure S51: MALDI-TOF mass spectrum of **2i** with dithranol matrix. Top panel is experimental data and bottom two panels are the predicted spectra

Compound 2j

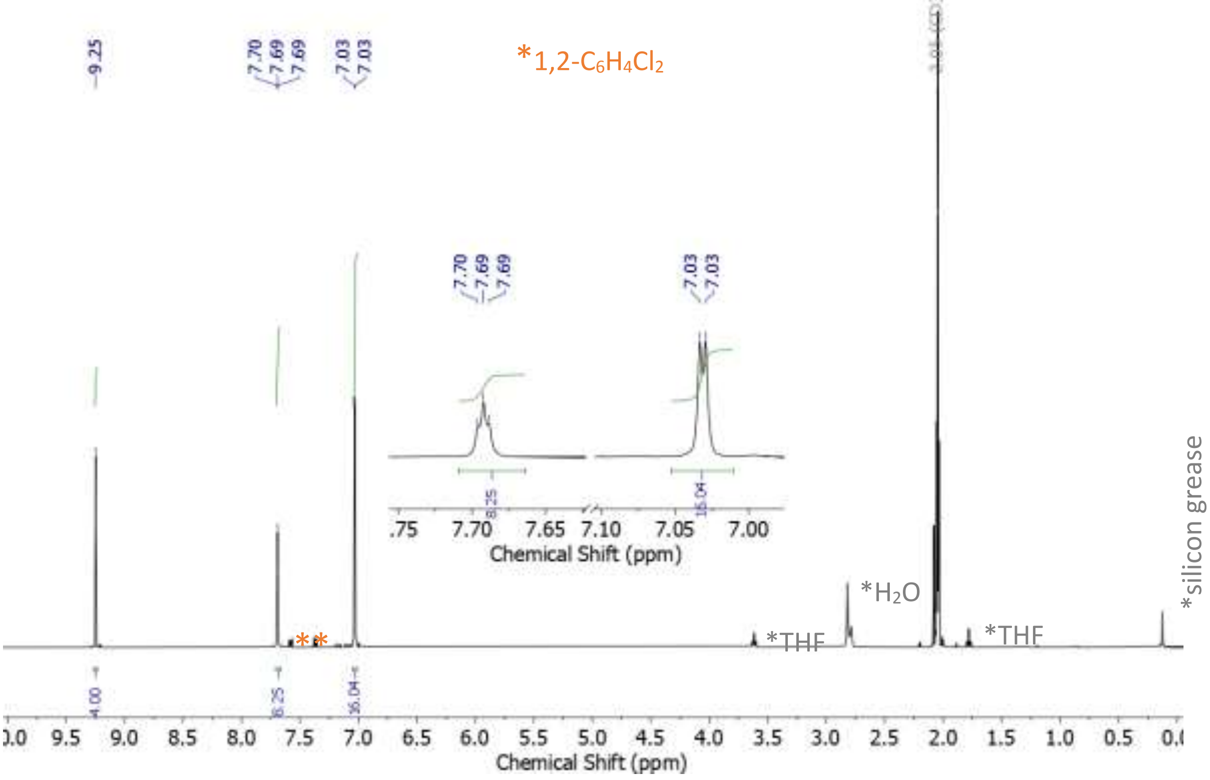

Figure S52: ^{1}H NMR spectrum of **2j** in $(CD_3)_2CO$

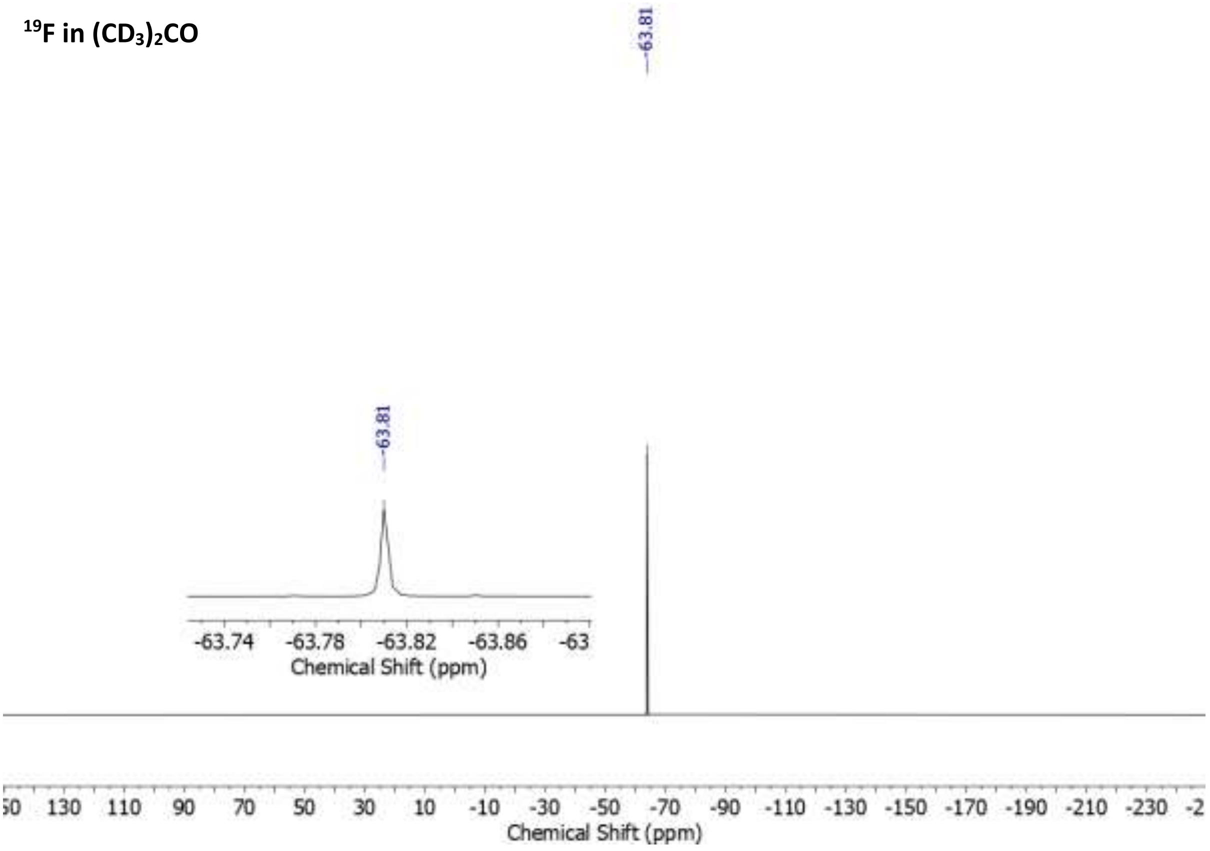

Figure S53: ^{19}F NMR spectrum of **2j** in $(CD_3)_2CO$

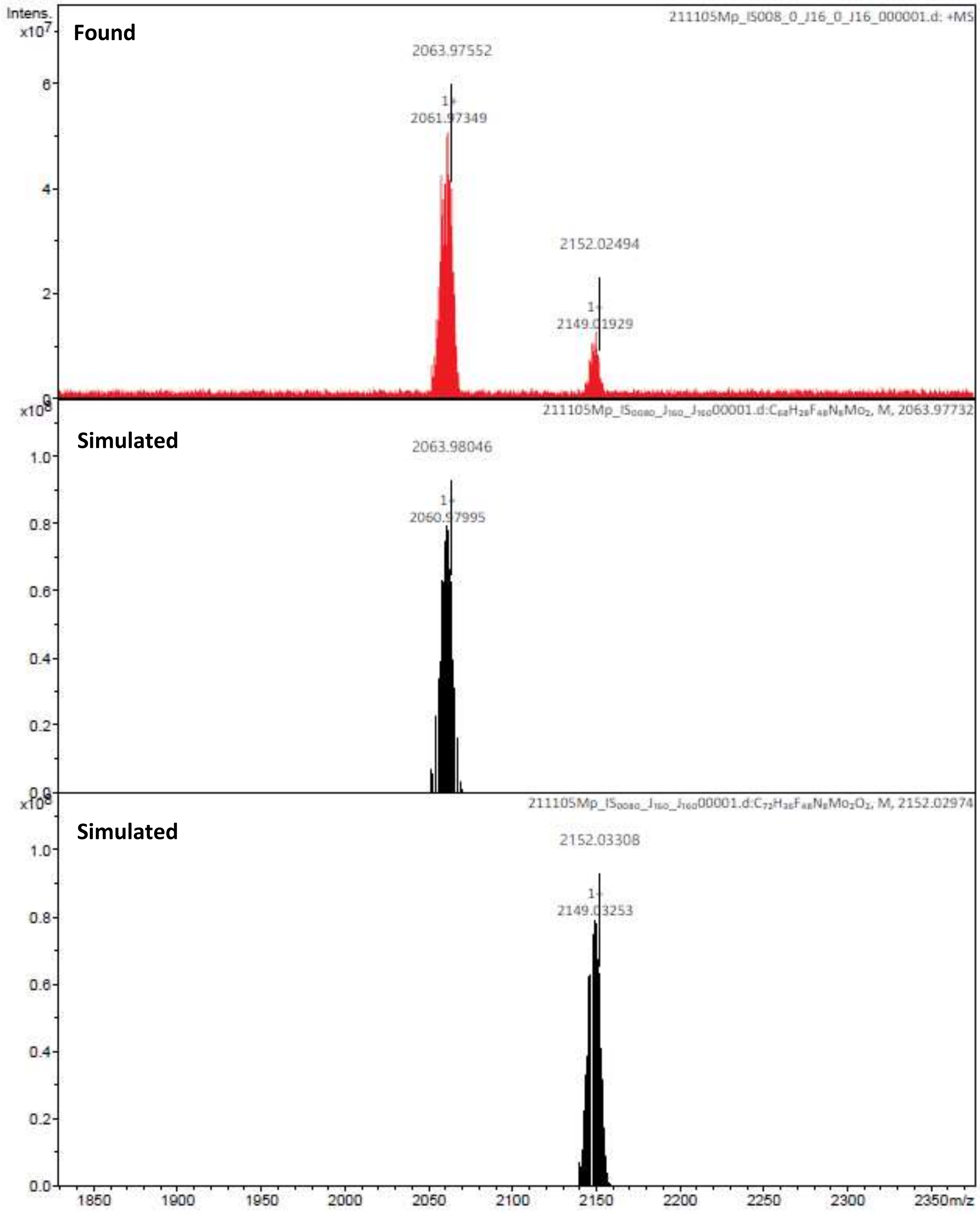

Figure S54: MALDI-TOF mass spectrum of **2j** with dithranol matrix. Top panel is experimental data and bottom two panels are the predicted spectra

Compound 3c

^{1}H in $CDCl_3$

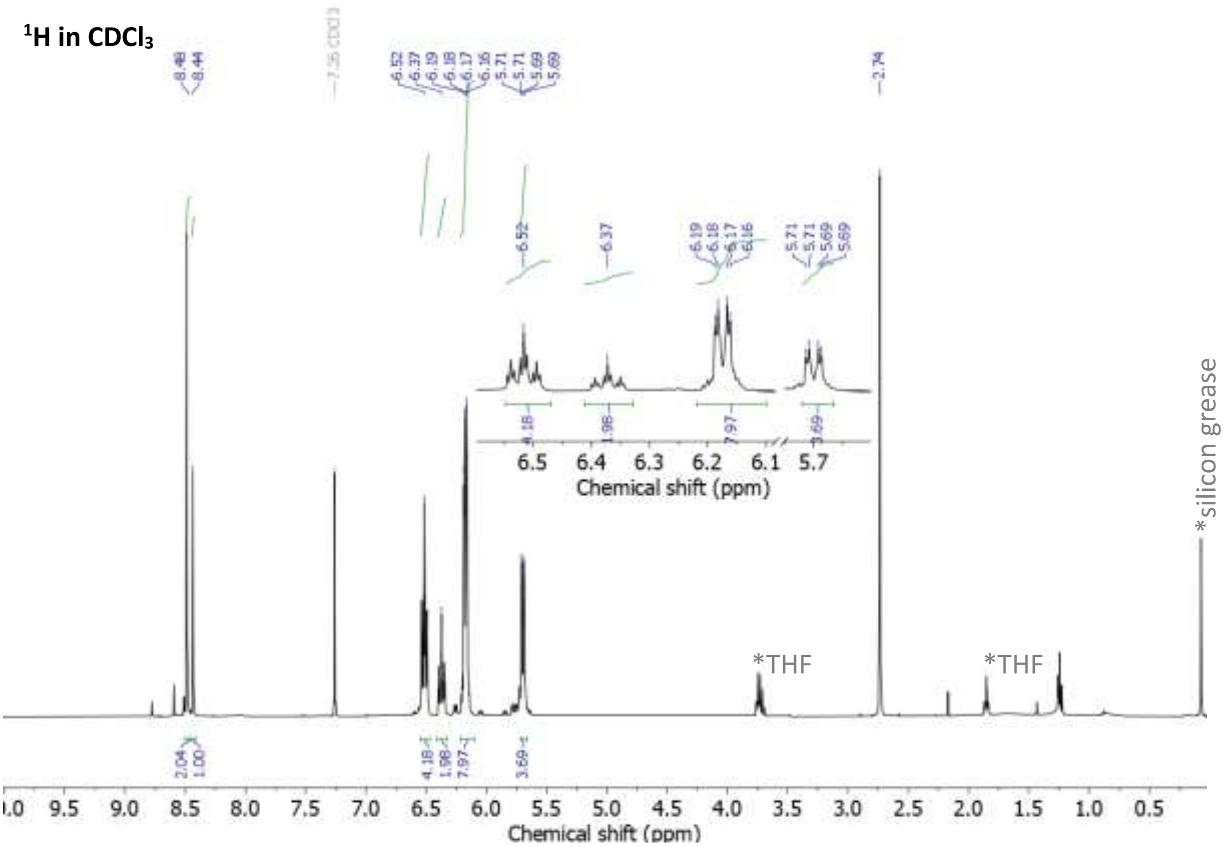

Figure S55: ^{1}H NMR spectrum of **3c** in $CDCl_3$

^{19}F in $CDCl_3$

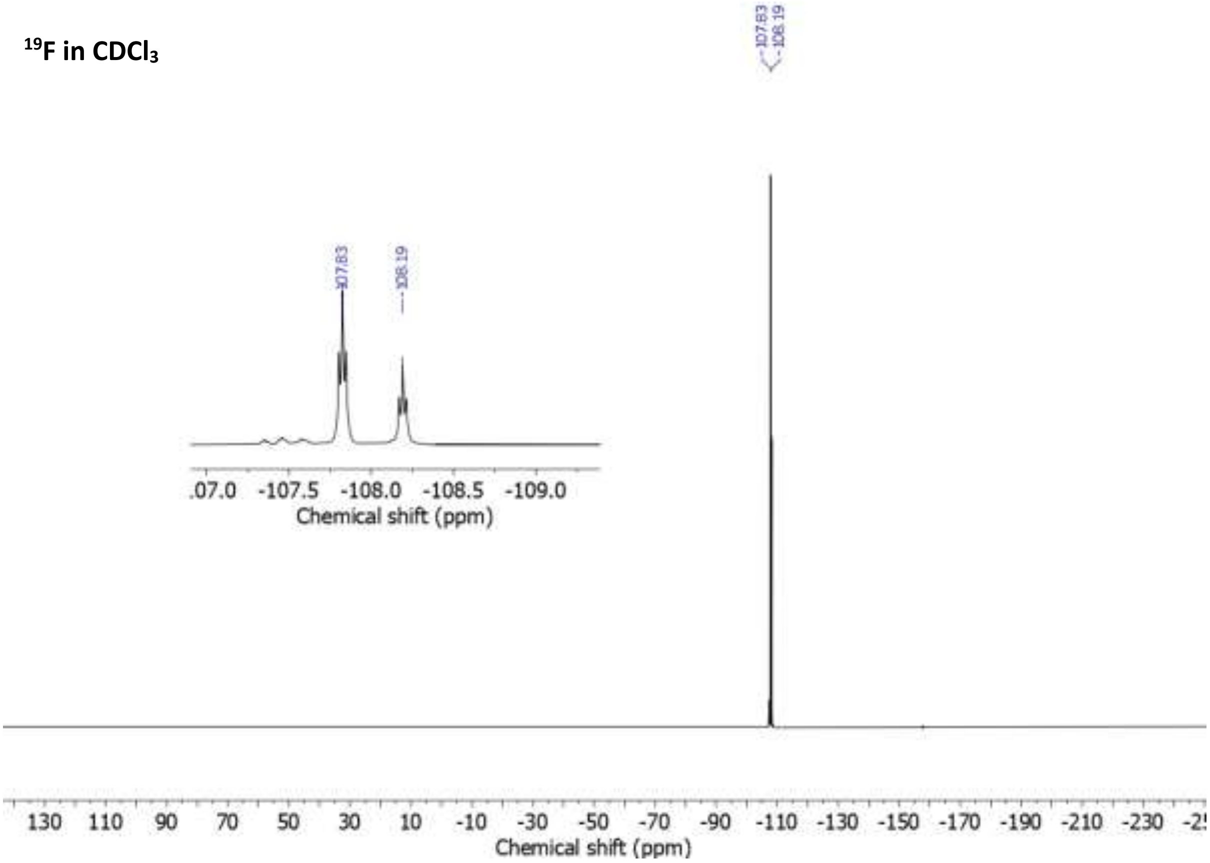

Figure S56: ^{19}F NMR spectrum of **3c** in $CDCl_3$

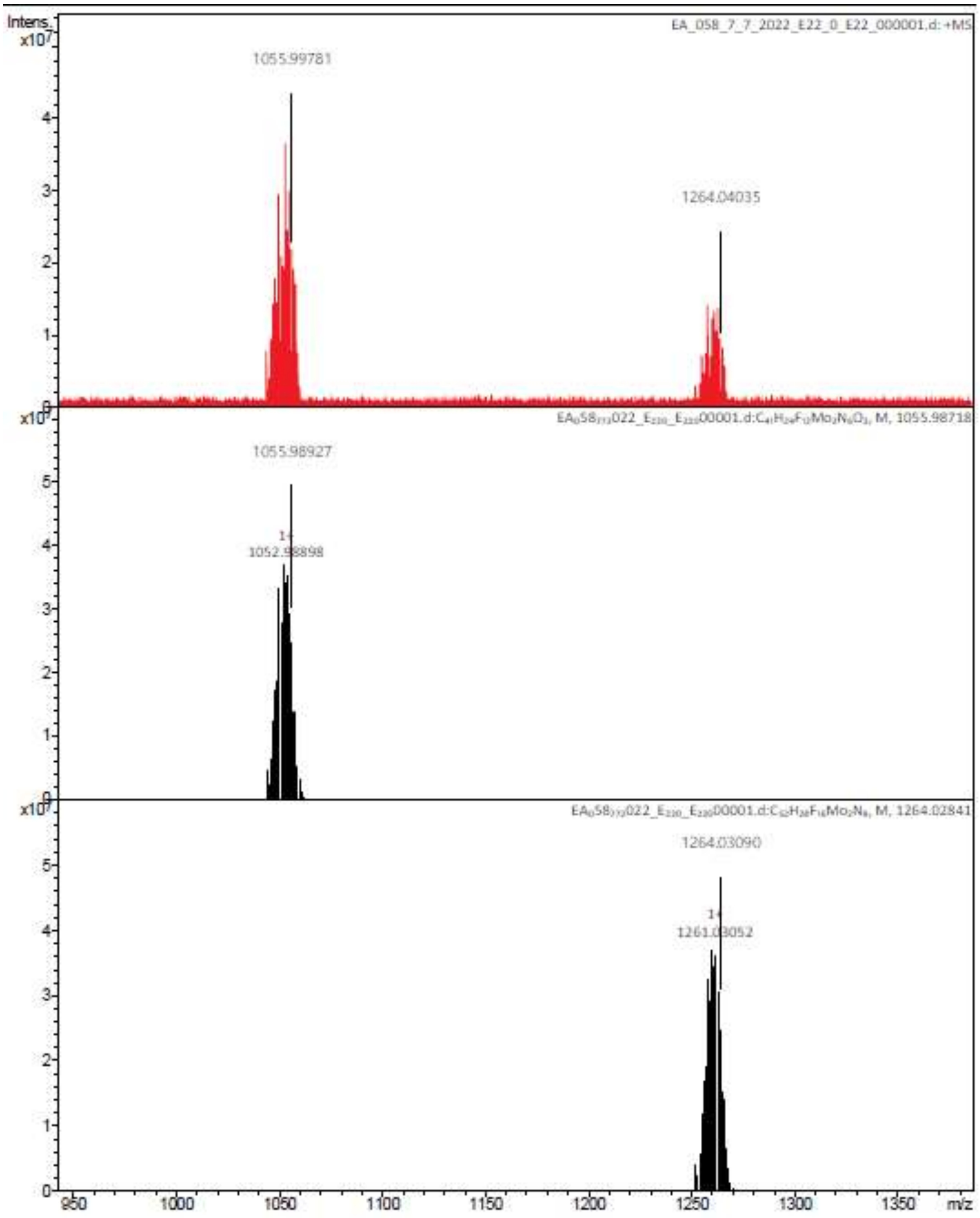

Figure S57: MALDI-TOF mass spectrum of 3c with dithranol matrix. Top panel is experimental data and bottom two panels are the predicted spectra

UV/Visible Absorption Spectra

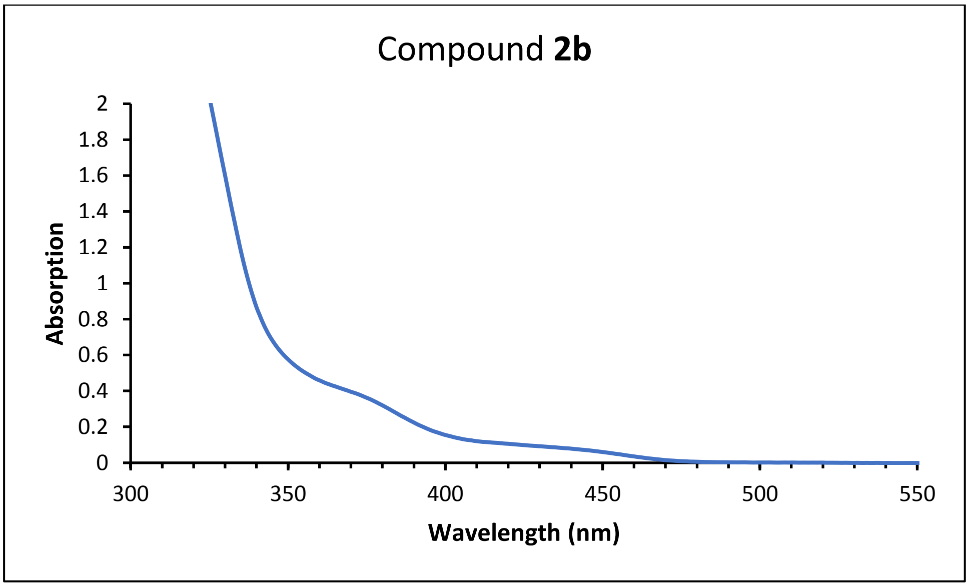

Figure S58: UV/Vis absorption spectrum of **2b** in THF

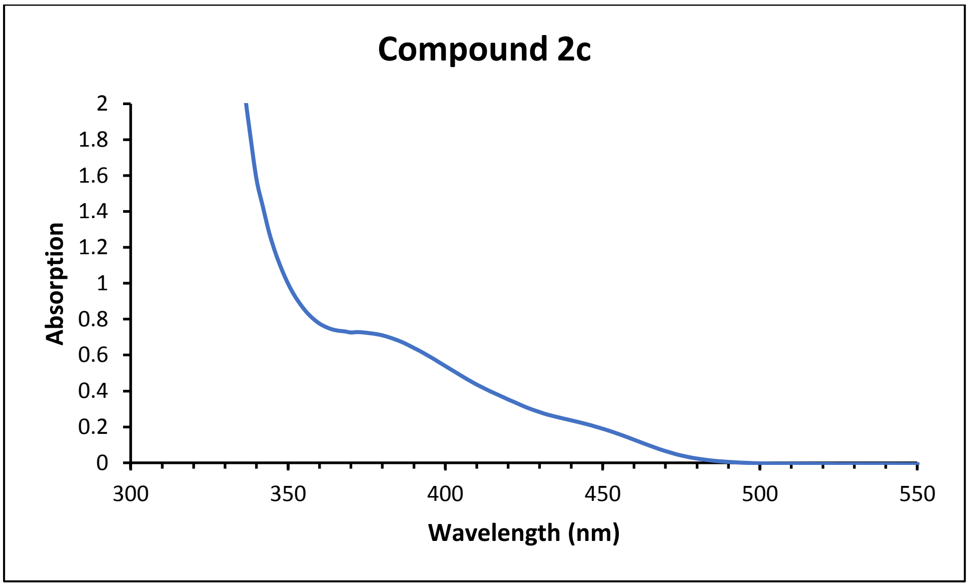

Figure S59: UV/Vis absorption spectrum of **2c** in THF

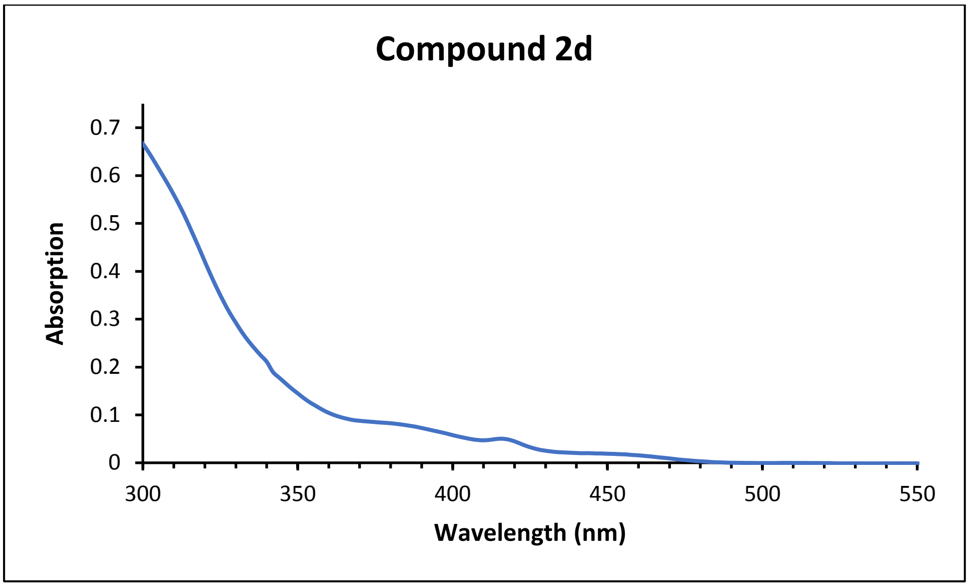

Figure S60: UV/Vis absorption spectrum of **2d** in THF

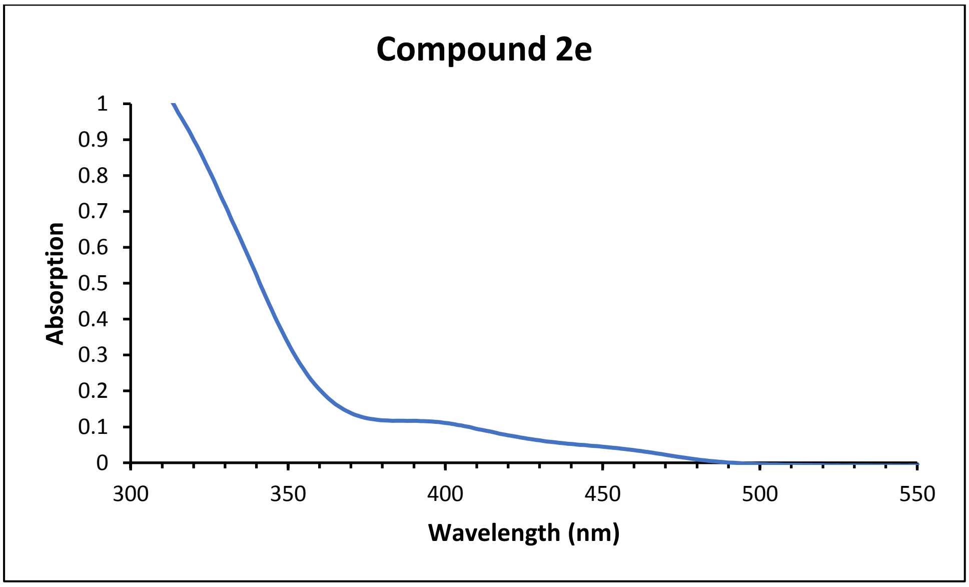

Figure S61: UV/Vis absorption spectrum of **2e** in THF

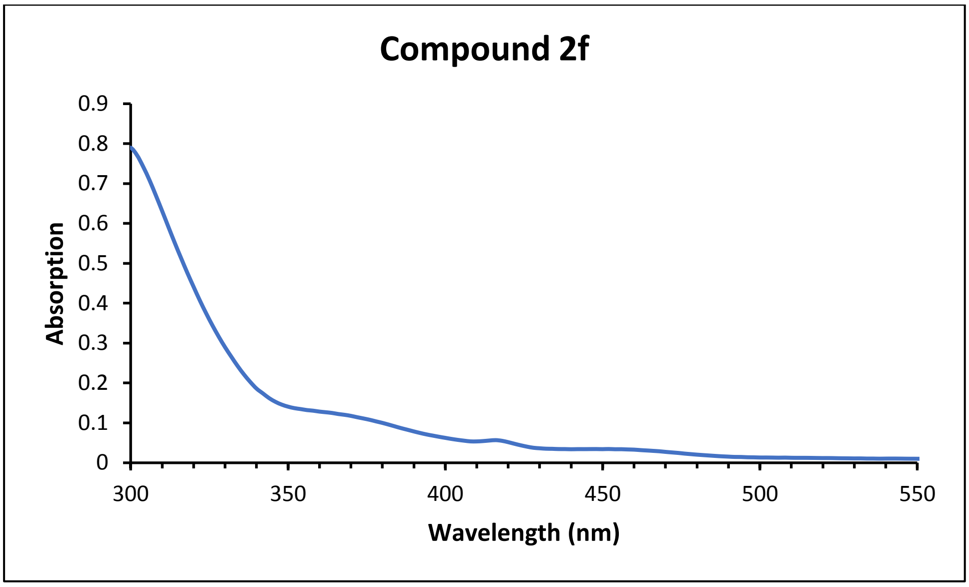

Figure S62: UV/Vis absorption spectrum of **2f** in THF

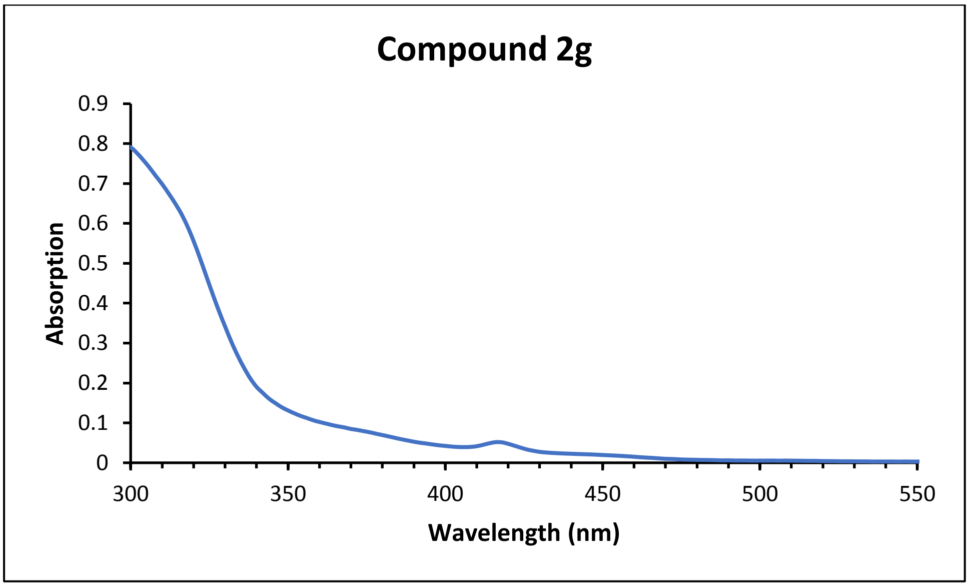

Figure S63: UV/Vis absorption spectrum of **2g** in THF

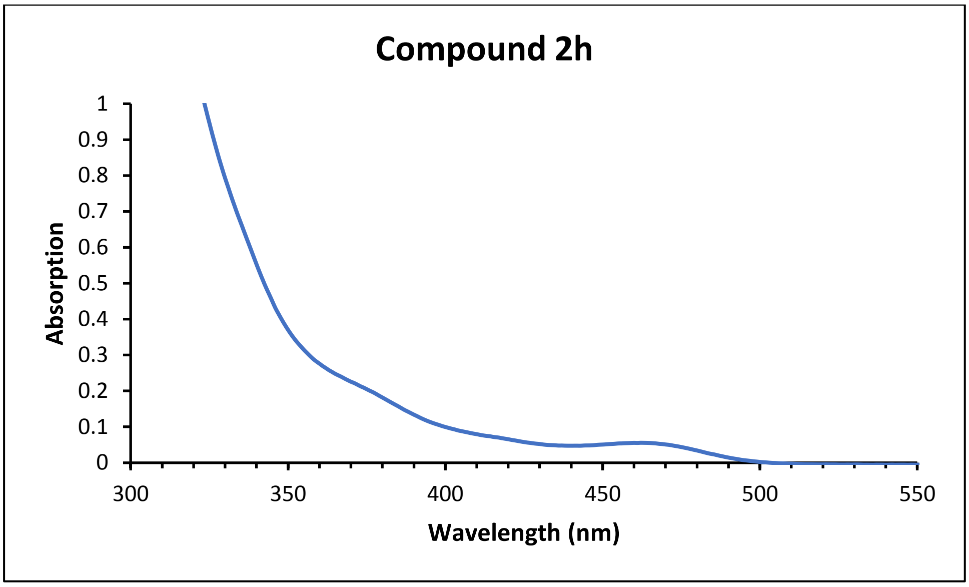

Figure S64: UV/Vis absorption spectrum of **2h** in THF

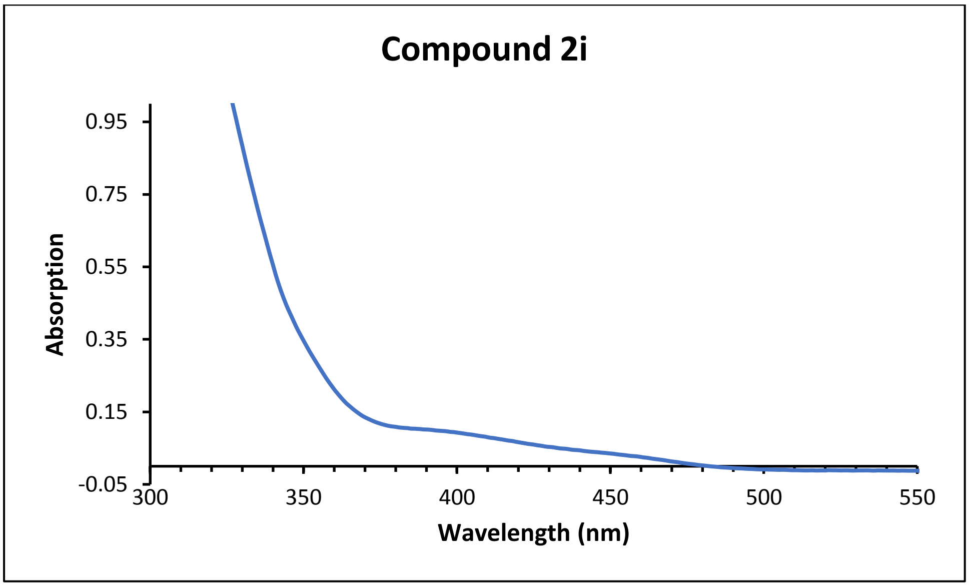

Figure S65: UV/Vis absorption spectrum of **2i** in THF

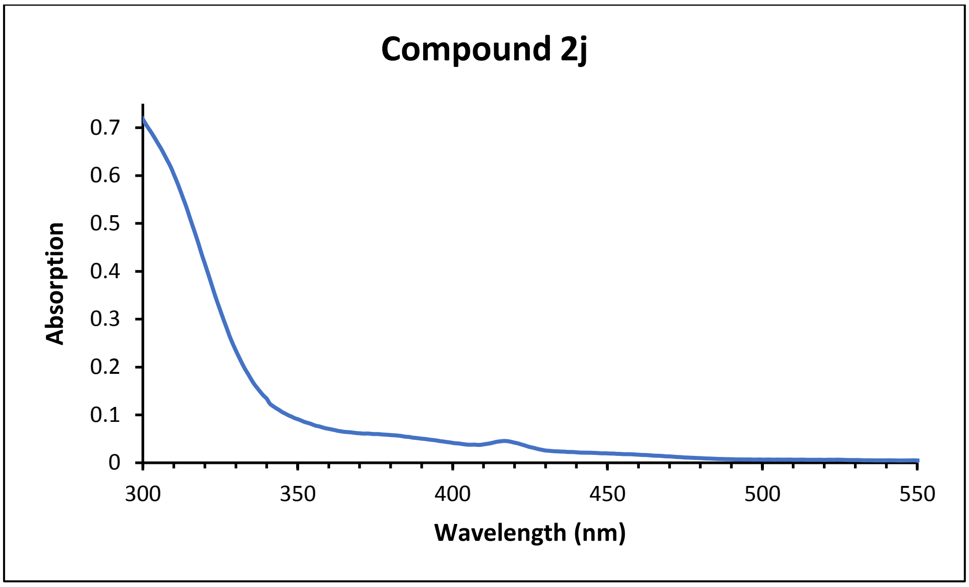

Figure S66: UV/Vis absorption spectrum of **2j** in THF

ATR Infrared Spectra

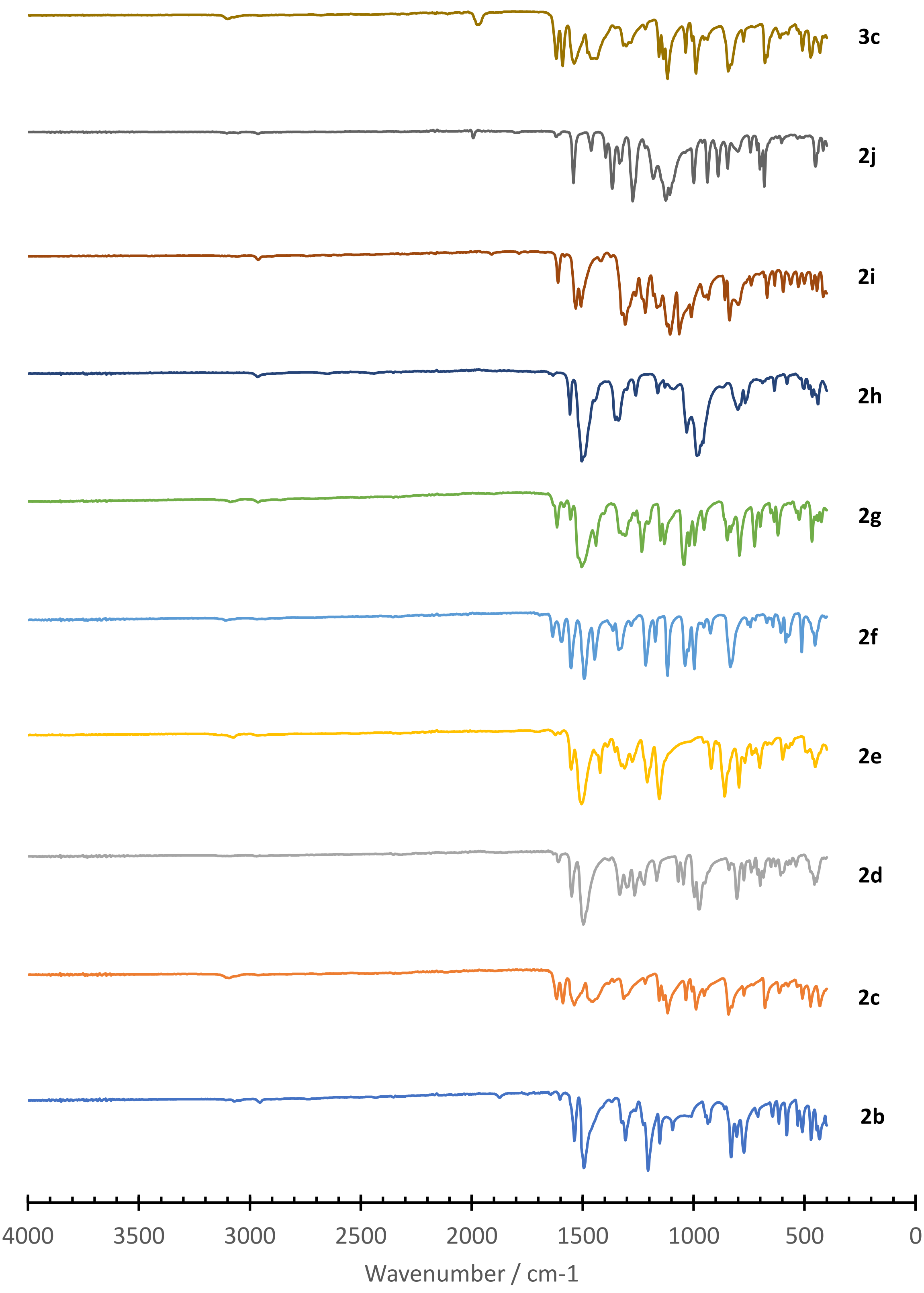

Figure S67: ATR IR spectra of complexes **2b**-**2j** and **3c**.

Crystallography Data

Crystallography data for compounds reported in this study are provided below. Hydrogen atoms (with the exception of N-H in compounds **1c-f**, shown in white) have been removed for clarity. Carbon atoms are shown in grey, nitrogen atoms are shown in lilac, fluorine atoms are shown in green, molybdenum atoms are shown in turquoise. Solvents of recrystallisation have been removed for clarity. Thermal ellipsoids are reported at 50 % probability.

Diffractometer control, data collection, initial unit cell determination, frame integration and unit-cell refinement was carried out with "Crysalis".[1] Face-indexed absorption corrections were applied using spherical harmonics, implemented in SCALE3 ABSPACK scaling algorithm.[2] OLEX2[3] was used for overall structure solution, refinement and preparation of computer graphics and publication data. Within OLEX2, the algorithm used for structure solution was "ShelXT dual-space".[4] Refinement by full-matrix least-squares used the SHELXL-97[5] algorithm within OLEX2.

Crystal Data for 1c

Empirical formula	$C_{13}H_8F_4N_2$
Formula weight	268.21
Temperature/K	110.00(10)
Crystal system	orthorhombic
Space group	Pcca
a/Å	22.3048(6)
b/Å	14.2721(4)
c/Å	6.9916(2)
α/°	90
β/°	90
γ/°	90
Volume/Å^3	2225.68(11)
Z	8
ρ_{calc}g/cm^3	1.601
μ/mm^{-1}	0.144
F(000)	1088.0
Crystal size/mm^3	0.19 × 0.14 × 0.11
Radiation	Mo Kα (λ = 0.71073)
2Θ range for data collection/°	6.742 to 54.914
Index ranges	-25 ≤ h ≤ 28, -10 ≤ k ≤ 17, -5 ≤ l ≤ 9
Reflections collected	5850
Independent reflections	2154 [R_{int} = 0.0300, R_{sigma} = 0.0409]
Data/restraints/parameters	2154/0/172
Goodness-of-fit on F^2	1.068
Final R indexes [I>=2σ (I)]	R_1 = 0.0456, wR_2 = 0.0975
Final R indexes [all data]	R_1 = 0.0678, wR_2 = 0.1129
Largest diff. peak/hole / e Å^{-3}	0.24/-0.22

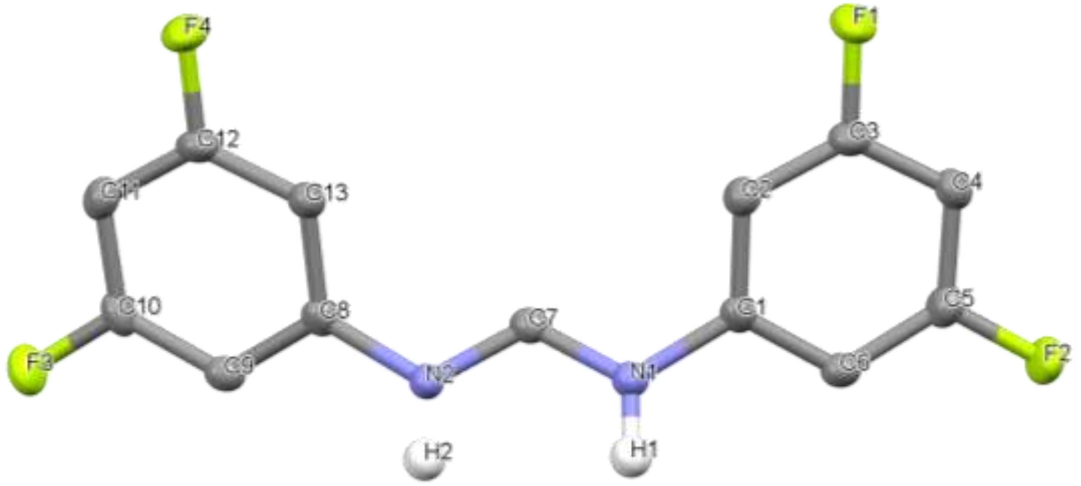

Figure S68: Single crystal XRD structure of **1c**. Occupancy of H1:H2 = 0.5:0.5.

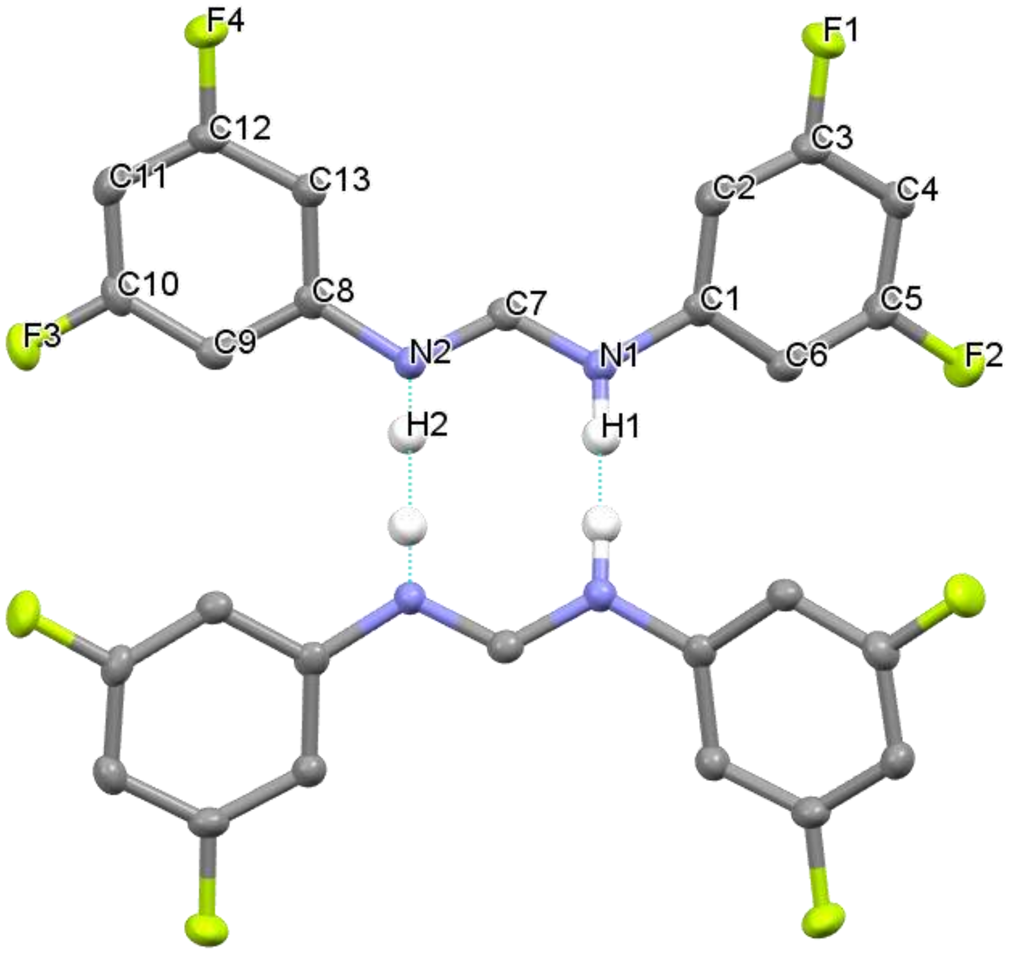

Figure S69: Dimeric unit observed in the single crystal XRD structure of **1c**. Hydrogen atoms have been removed for clarity. Occupancy of H1:H2 = 0.5:0.5.

Crystal Data for 1d

Empirical formula	$C_{13}H_6F_6N_2$
Formula weight	304.20
Temperature/K	110.00(10)
Crystal system	monoclinic
Space group	$P2_1/n$
a/Å	6.8144(6)
b/Å	12.3050(10)
c/Å	14.0595(13)
α/°	90
β/°	100.340(8)
γ/°	90
Volume/Å^3	1159.75(17)
Z	4
ρ_{calc}g/cm^3	1.742
μ/mm^{-1}	1.554
F(000)	608.0
Crystal size/mm^3	0.163 × 0.104 × 0.091
Radiation	Cu Kα (λ = 1.54184)
2Θ range for data collection/°	9.62 to 134.134
Index ranges	-5 ≤ h ≤ 8, -14 ≤ k ≤ 13, -14 ≤ l ≤ 16
Reflections collected	3846
Independent reflections	2065 [R_{int} = 0.0213, R_{sigma} = 0.0306]
Data/restraints/parameters	2065/0/215
Goodness-of-fit on F^2	1.065
Final R indexes [I>=2σ (I)]	R_1 = 0.0447, wR_2 = 0.1130
Final R indexes [all data]	R_1 = 0.0551, wR_2 = 0.1202
Largest diff. peak/hole / e Å^{-3}	0.27/-0.30

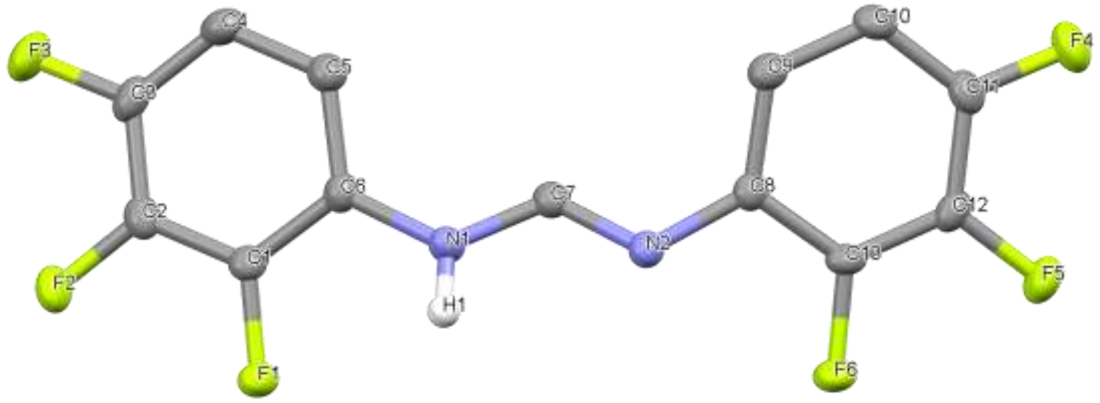

Figure S70: Single crystal XRD structure of **1d**.

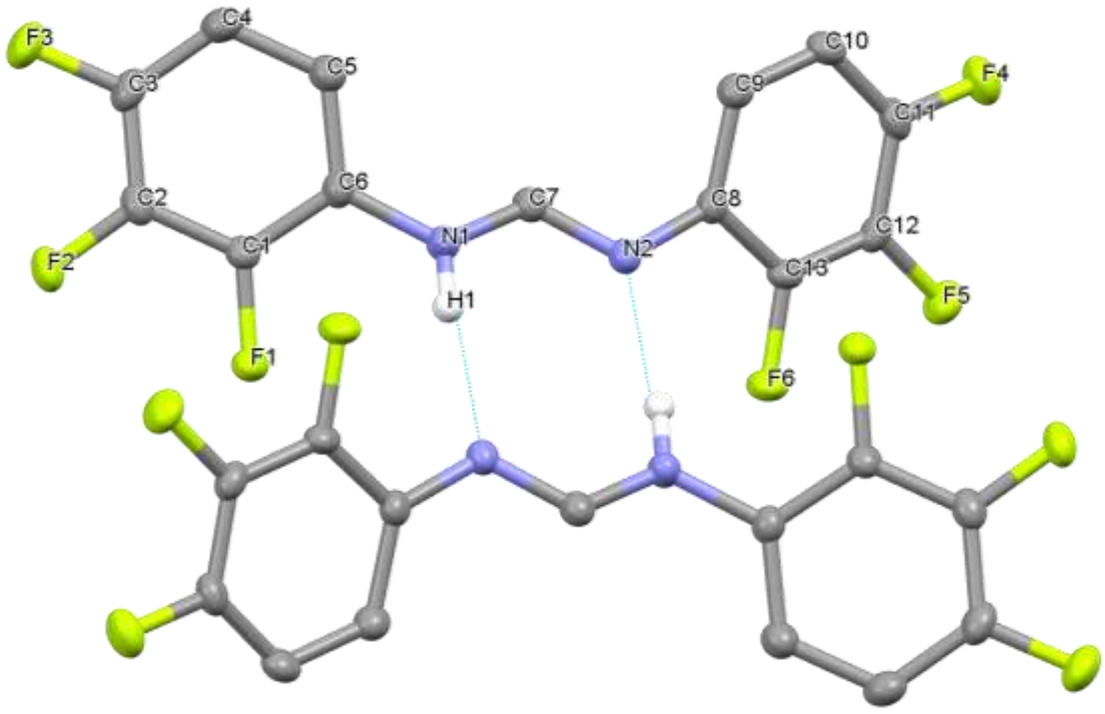

Figure S71: Dimeric unit observed in the single crystal XRD structure of **1d**. Hydrogen atoms have been removed for clarity.

Crystal Data for 1e

Empirical formula	$C_{13}H_6F_6N_2$
Formula weight	304.20
Temperature/K	110.00(10)
Crystal system	monoclinic
Space group	$P2_1/n$
a/Å	6.33512(19)
b/Å	14.6108(5)
c/Å	12.4972(4)
α/°	90
β/°	90.176(3)
γ/°	90
Volume/Å^3	1156.75(6)
Z	4
ρ_{calc}g/cm^3	1.747
μ/mm^{-1}	1.558
F(000)	608.0
Crystal size/mm^3	0.238 × 0.071 × 0.039
Radiation	Cu Kα (λ = 1.54184)
2Θ range for data collection/°	9.312 to 134.16
Index ranges	-7 ≤ h ≤ 6, -17 ≤ k ≤ 16, -13 ≤ l ≤ 14
Reflections collected	7363
Independent reflections	2070 [R_{int} = 0.0292, R_{sigma} = 0.0261]
Data/restraints/parameters	2070/0/216
Goodness-of-fit on F^2	1.052
Final R indexes [I>=2σ (I)]	R_1 = 0.0329, wR_2 = 0.0818
Final R indexes [all data]	R_1 = 0.0407, wR_2 = 0.0886
Largest diff. peak/hole / e Å^{-3}	0.21/-0.18

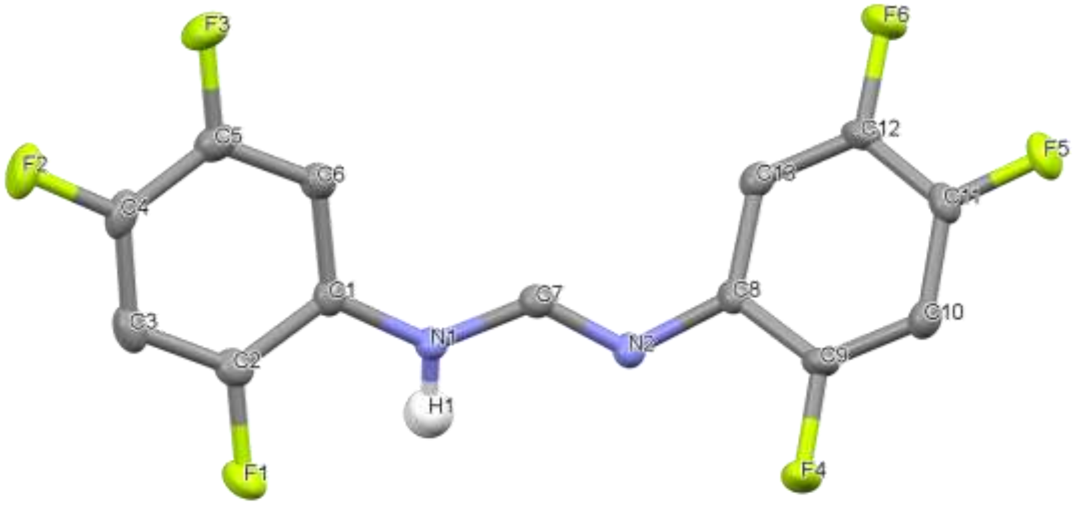

Figure S72: Single crystal XRD structure of **1e**.

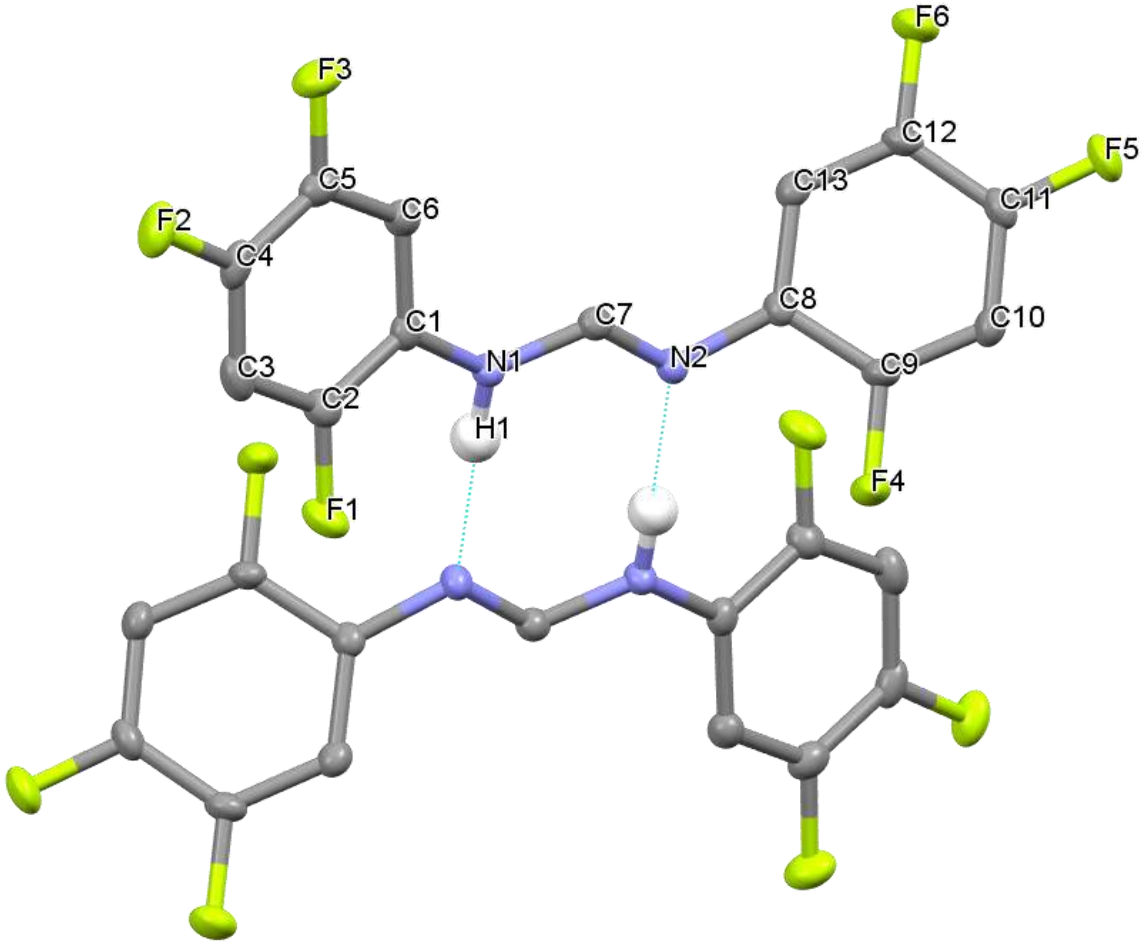

Figure S73: Dimeric unit observed in the single crystal XRD structure of **1e**. Hydrogen atoms have been removed for clarity.

Crystal Data for 1f

Empirical formula	$C_{13}H_6F_6N_2$
Formula weight	304.20
Temperature/K	109.95(10)
Crystal system	monoclinic
Space group	$P2_1/c$
a/Å	11.8233(7)
b/Å	12.5392(7)
c/Å	8.0290(5)
α/°	90
β/°	99.750(6)
γ/°	90
Volume/Å^3	1173.15(12)
Z	4
ρ_{calc}g/cm^3	1.722
μ/mm^{-1}	1.537
F(000)	608.0
Crystal size/mm^3	0.354 × 0.042 × 0.023
Radiation	Cu Kα (λ = 1.54184)
2Θ range for data collection/°	7.586 to 134.128
Index ranges	-14 ≤ h ≤ 7, -14 ≤ k ≤ 13, -8 ≤ l ≤ 9
Reflections collected	3977
Independent reflections	2095 [R_{int} = 0.0220, R_{sigma} = 0.0316]
Data/restraints/parameters	2095/0/214
Goodness-of-fit on F^2	1.030
Final R indexes [I>=2σ (I)]	R_1 = 0.0345, wR_2 = 0.0864
Final R indexes [all data]	R_1 = 0.0447, wR_2 = 0.0942
Largest diff. peak/hole / e Å^{-3}	0.22/-0.23

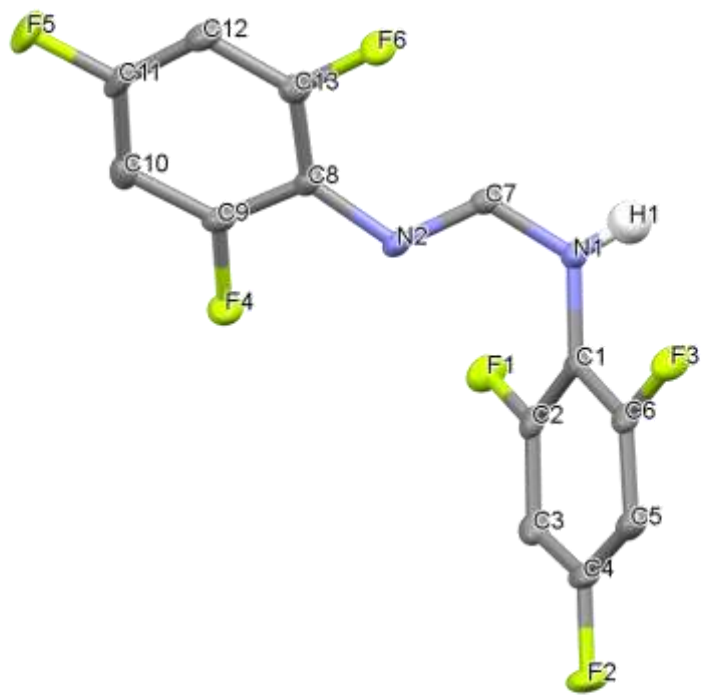

Figure S74: Single crystal XRD structure of **1f**.

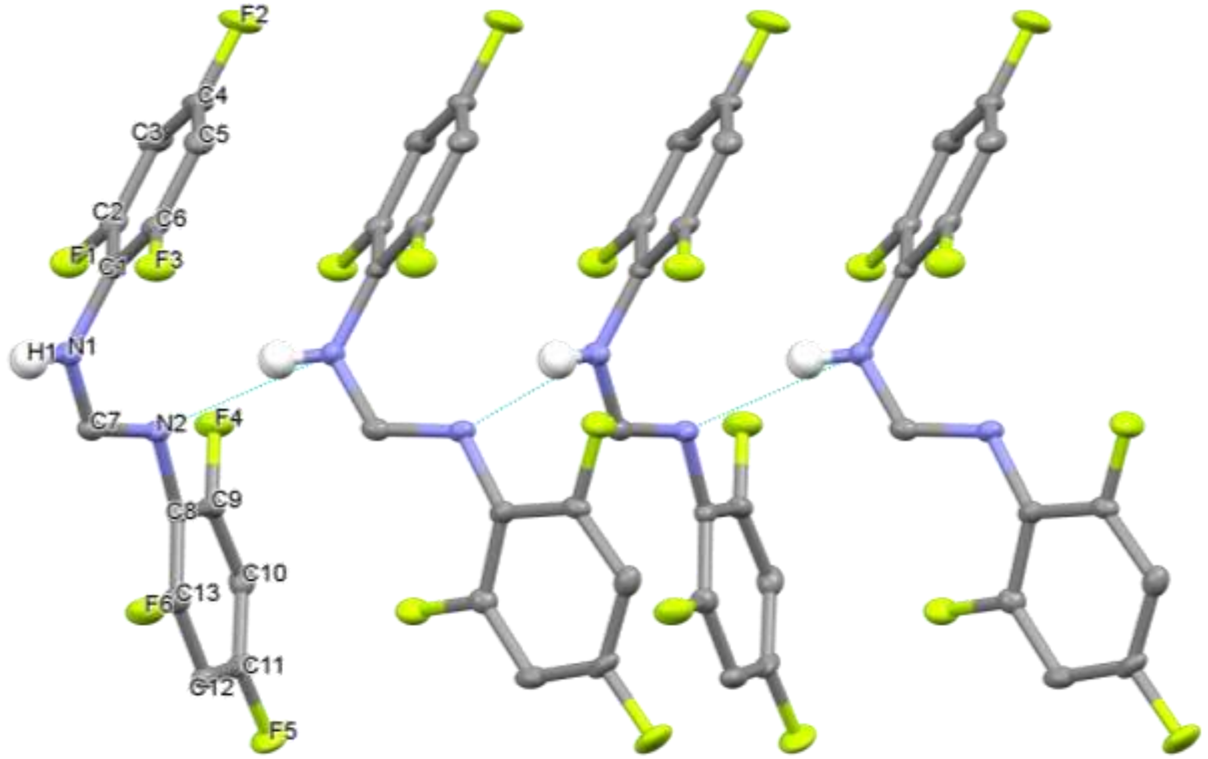

Figure S75: H bonded 1D chain observed in the single crystal XRD structure of **1f**. Hydrogen atoms have been removed for clarity.

Crystal Data for 2b

Empirical formula	$C_{52}H_{36}F_8Mo_2N_8$
Formula weight	1116.77
Temperature/K	110.00(10)
Crystal system	tetragonal
Space group	P4/nnc
a/Å	13.9035(2)
b/Å	13.9035(2)
c/Å	12.6354(3)
α/°	90
β/°	90
γ/°	90
Volume/Å^3	2442.51(9)
Z	2
ρ_{calc}g/cm^3	1.518
μ/mm^{-1}	4.853
F(000)	1120.0
Crystal size/mm^3	0.21 × 0.14 × 0.09
Radiation	Cu Kα (λ = 1.54184)
2Θ range for data collection/°	8.994 to 142.996
Index ranges	-16 ≤ h ≤ 13, -17 ≤ k ≤ 17, -15 ≤ l ≤ 15
Reflections collected	15792
Independent reflections	1198 [R_{int} = 0.0472, R_{sigma} = 0.0162]
Data/restraints/parameters	1198/0/147
Goodness-of-fit on F^2	1.223
Final R indexes [I>=2σ (I)]	R_1 = 0.0547, wR_2 = 0.1548
Final R indexes [all data]	R_1 = 0.0593, wR_2 = 0.1594
Largest diff. peak/hole / e Å^{-3}	1.61/-0.69

Refinement Special Details: The para-fluoro phenyl group in the asymmetric unit was disordered around the Cpara-Cmeta axis. The disordered groups were modelled in two positions. The nitrogen (N1), and the disordered carbons in the ring (C2, C3, C4) were modelled in two positions, with occupancy ratio 0.489:0.511. The para carbon, C5 and the para-fluoro group (F1) were modelled in three positions, with occupancies 0.489:0.255:0.255. The ADPs of C5, C5A and C5B were set to be equal. The bridging carbon, C1, was modelled into two positions with occupancies 0.245:0.255

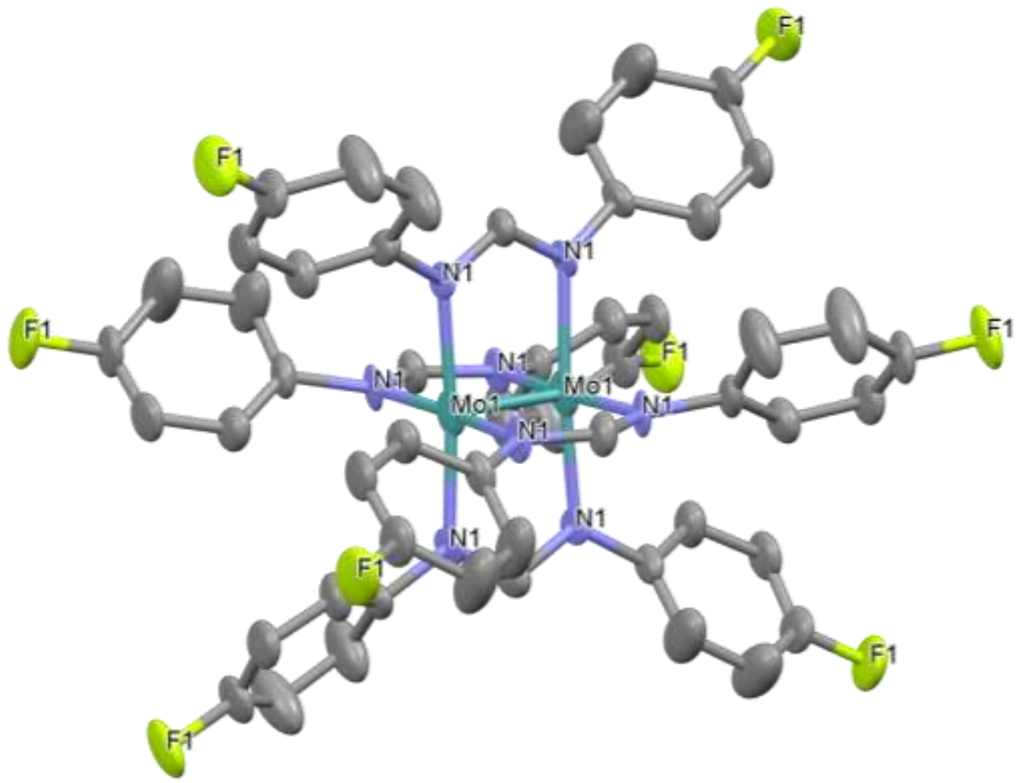

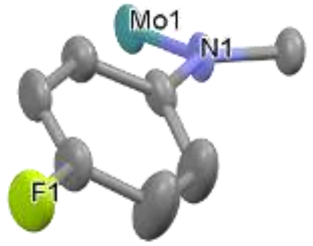

Figure S76: Single crystal XRD structure of **2b** (Top) and asymmetric unit only (Bottom). A second paddlewheel motif was observed rotated 41.83 ° about the Mo-Mo axis which has been removed for clarity.

Crystal Data for 2c

Empirical formula	$C_{52}H_{28}N_8F_{16}Mo_2$
Formula weight	1260.70
Temperature/K	110.00(10)
Crystal system	monoclinic
Space group	$P2_1/c$
a/Å	9.6858(2)
b/Å	25.0090(5)
c/Å	9.8860(2)
α/°	90
β/°	100.626(2)
γ/°	90
Volume/Å^3	2353.66(9)
Z	2
ρ_{calc}g/cm^3	1.779
μ/mm^{-1}	0.645
F(000)	1248.0
Crystal size/mm^3	0.276 × 0.231 × 0.107
Radiation	Mo Kα (λ = 0.71073)
2Θ range for data collection/°	6.722 to 60.148
Index ranges	-13 ≤ h ≤ 13, -22 ≤ k ≤ 35, -13 ≤ l ≤ 13
Reflections collected	14191
Independent reflections	6897 [R_{int} = 0.0259, R_{sigma} = 0.0393]
Data/restraints/parameters	6897/0/353
Goodness-of-fit on F^2	1.073
Final R indexes [I>=2σ (I)]	R_1 = 0.0322, wR_2 = 0.0693
Final R indexes [all data]	R_1 = 0.0398, wR_2 = 0.0734
Largest diff. peak/hole / e Å^{-3}	0.54/-0.37

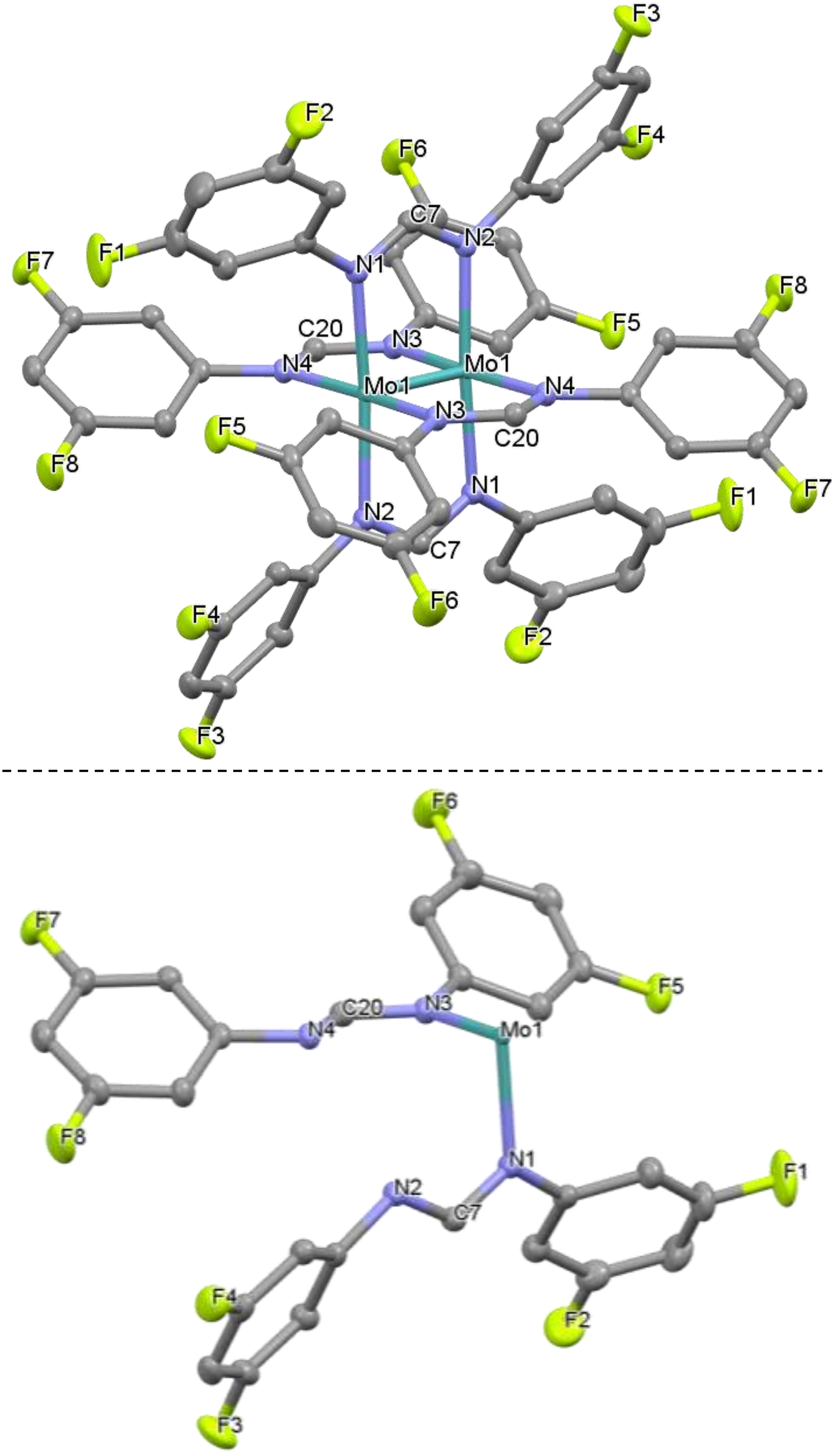

Figure S77: Single crystal XRD structure of **2c** (Top) and asymmetric unit only (Bottom).

Crystal Data for 2d

Empirical formula	$C_{104}H_{40}F_{48}Mo_4N_{16}$
Formula weight	2809.28
Temperature/K	110.00(10)
Crystal system	monoclinic
Space group	$P2_1/n$
a/Å	11.6998(3)
b/Å	14.6607(3)
c/Å	18.4878(3)
α/°	90
β/°	107.805(2)
γ/°	90
Volume/Å^3	3019.27(12)
Z	1
ρ_{calc}g/cm^3	1.545
μ/mm^{-1}	4.453
F(000)	1376.0
Crystal size/mm^3	0.184 × 0.141 × 0.118
Radiation	Cu Kα (λ = 1.54184)
2Θ range for data collection/°	7.848 to 142.106
Index ranges	-13 ≤ h ≤ 14, -13 ≤ k ≤ 17, -22 ≤ l ≤ 18
Reflections collected	7206
Independent reflections	4374 [R_{int} = 0.0151, R_{sigma} = 0.0230]
Data/restraints/parameters	4374/0/388
Goodness-of-fit on F^2	1.048
Final R indexes [I>=2σ (I)]	R_1 = 0.0222, wR_2 = 0.0582
Final R indexes [all data]	R_1 = 0.0241, wR_2 = 0.0596
Largest diff. peak/hole / e Å^{-3}	0.39/-0.52

Diffraction special details: Due to misidentified symmetry in pre-experiment, data for an orthorhombic cell was collected. Cell of final solution was monoclinic, leading to missing data. Θ coverage = 0.77

Refinement special details: Solvent channels through the structure could not be modelled explicitly, and a SQUEEZE solvent mask was used to implicitly model solvent

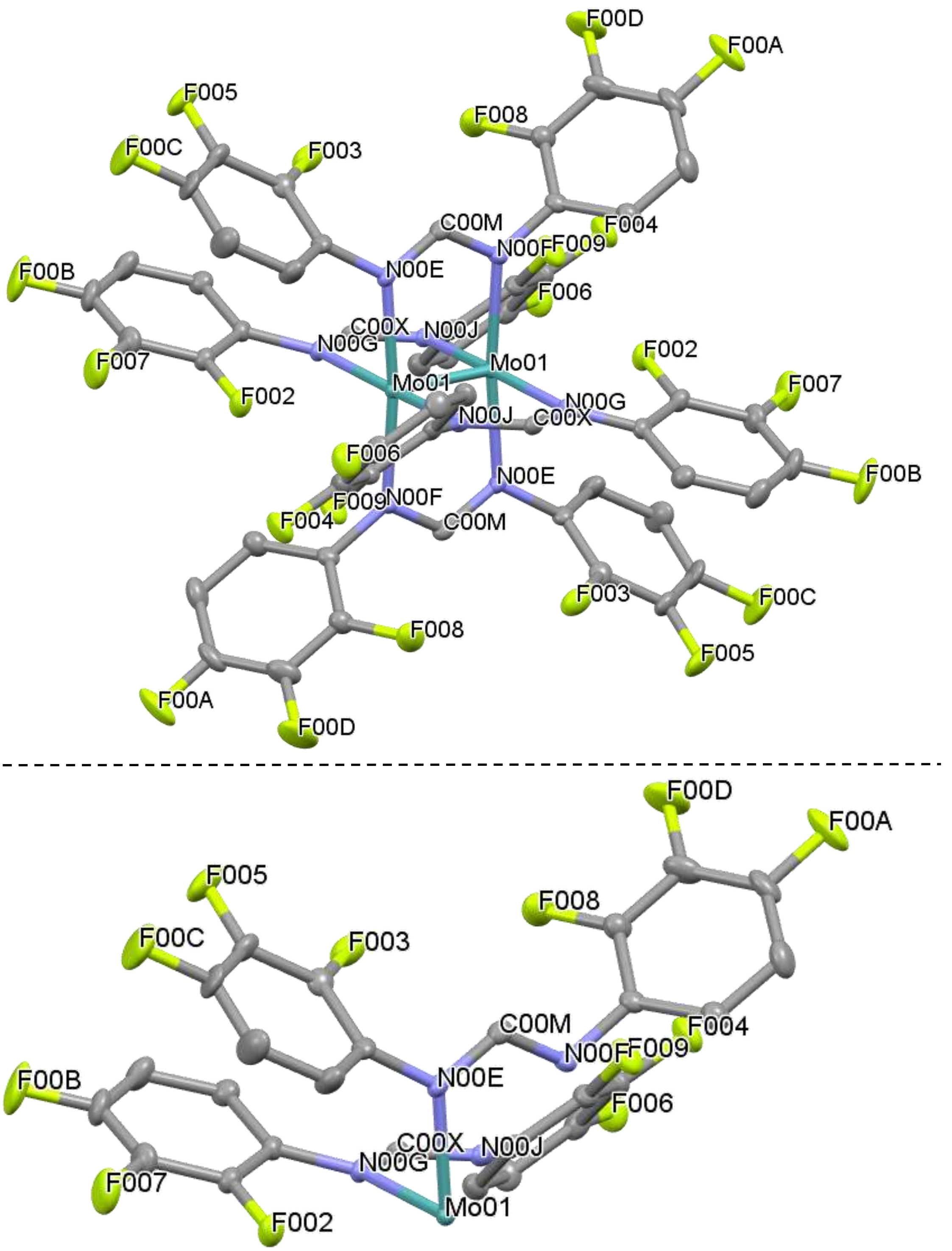

Figure S78: Single crystal XRD structure of **2d** (top) and asymmetric unit only (bottom).

Crystal Data for 2e

Empirical formula	$C_{52}H_{20}F_{24}Mo_2N_8$
Formula weight	1404.64
Temperature/K	110.00(14)
Crystal system	triclinic
Space group	P-1
a/Å	12.3797(5)
b/Å	19.1386(6)
c/Å	21.9281(7)
α/°	73.700(3)
β/°	76.805(3)
γ/°	87.364(3)
Volume/Å^3	4854.0(3)
Z	4
ρ_{calc}g/cm^3	1.922
μ/mm^{-1}	5.540
F(000)	2752.0
Crystal size/mm^3	0.052 × 0.031 × 0.025
Radiation	Cu Kα (λ = 1.54184)
2Θ range for data collection/°	7.298 to 134.16
Index ranges	-11 ≤ h ≤ 14, -22 ≤ k ≤ 22, -26 ≤ l ≤ 26
Reflections collected	34716
Independent reflections	17310 [R_{int} = 0.0369, R_{sigma} = 0.0520]
Data/restraints/parameters	17310/12/1575
Goodness-of-fit on F^2	1.010
Final R indexes [I>=2σ (I)]	R_1 = 0.0334, wR_2 = 0.0686
Final R indexes [all data]	R_1 = 0.0506, wR_2 = 0.0756
Largest diff. peak/hole / e Å^{-3}	0.65/-0.61

Refinement Special Details

Two of the trifluorophenyl groups were disordered and modelled in two positions differing by a 180 degree rotation about the Cipso-Cpara axis. For these the 2,5 fluorines and 3,6 hydrogen atoms were modelled in two positions. The occupancies refined to 0.955:0.045(4) for F19 & F21, and 0.936:0.064(4) for F37 & F39. The ADPs of F19A and F21A were constrained to be equal, as were the ADPs of F37a and F39a.

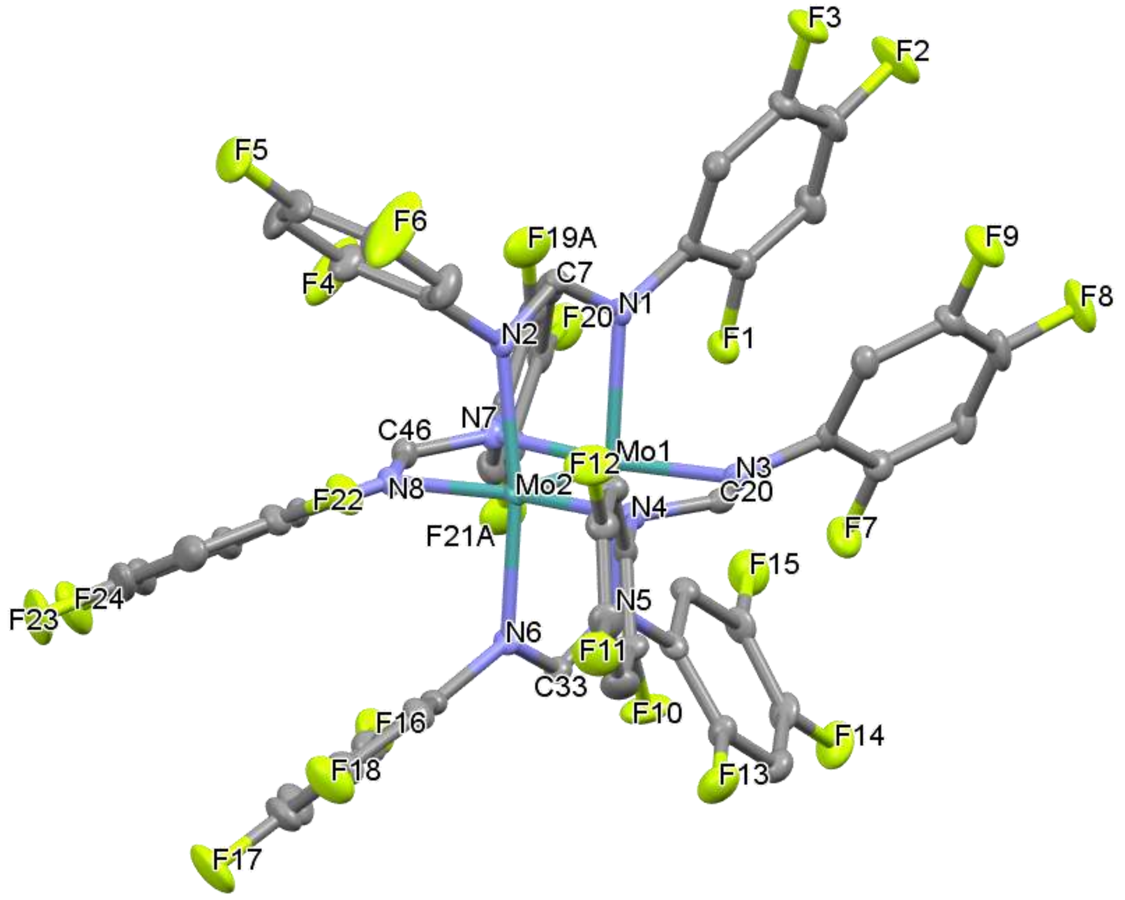

Figure S79: Single crystal XRD structure of **2e**. A single moiety of **2e** was selected from the unit cell for visualization.

Crystal Data for 2f

Empirical formula	$C_{59.99}H_{35.98}F_{24}Mo_2N_8O_2$
Formula weight	1548.71
Temperature/K	110.00(10)
Crystal system	triclinic
Space group	P-1
a/Å	11.1746(7)
b/Å	11.3264(6)
c/Å	11.9436(6)
α/°	71.875(4)
β/°	82.228(5)
γ/°	80.445(5)
Volume/Å^3	1411.09(14)
Z	1
ρ_{calc}g/cm^3	1.822
μ/mm^{-1}	4.859
F(000)	768.0
Crystal size/mm^3	0.22 × 0.159 × 0.025
Radiation	Cu Kα (λ = 1.54184)
2Θ range for data collection/°	7.82 to 134.156
Index ranges	-13 ≤ h ≤ 13, -11 ≤ k ≤ 13, -14 ≤ l ≤ 12
Reflections collected	9380
Independent reflections	5044 [R_{int} = 0.0230, R_{sigma} = 0.0347]
Data/restraints/parameters	5044/8/536
Goodness-of-fit on F^2	1.031
Final R indexes [I>=2σ (I)]	R_1 = 0.0265, wR_2 = 0.0626
Final R indexes [all data]	R_1 = 0.0320, wR_2 = 0.0660
Largest diff. peak/hole / e Å^{-3}	0.52/-0.52

Refinement Special Details

The tetrahydrofuran (THF) was disordered and modelled in three positions in a refined ratio of 0.519(5):0.297(6):0.182(5). For the minor form of the THF, the C-O bond lengths were restrained to be 1.42 angstroms, the C-C bond lengths restrained to be 1.52 angstroms and the C27c-C29c and C28c-C30c distances restrained to be 2.36 angstroms. The ADP of closely proximal atoms were constrained to be equal as follows:

C30a, C30b & C30c

C29a & C29c

C28a & C28c

O1a & O1c

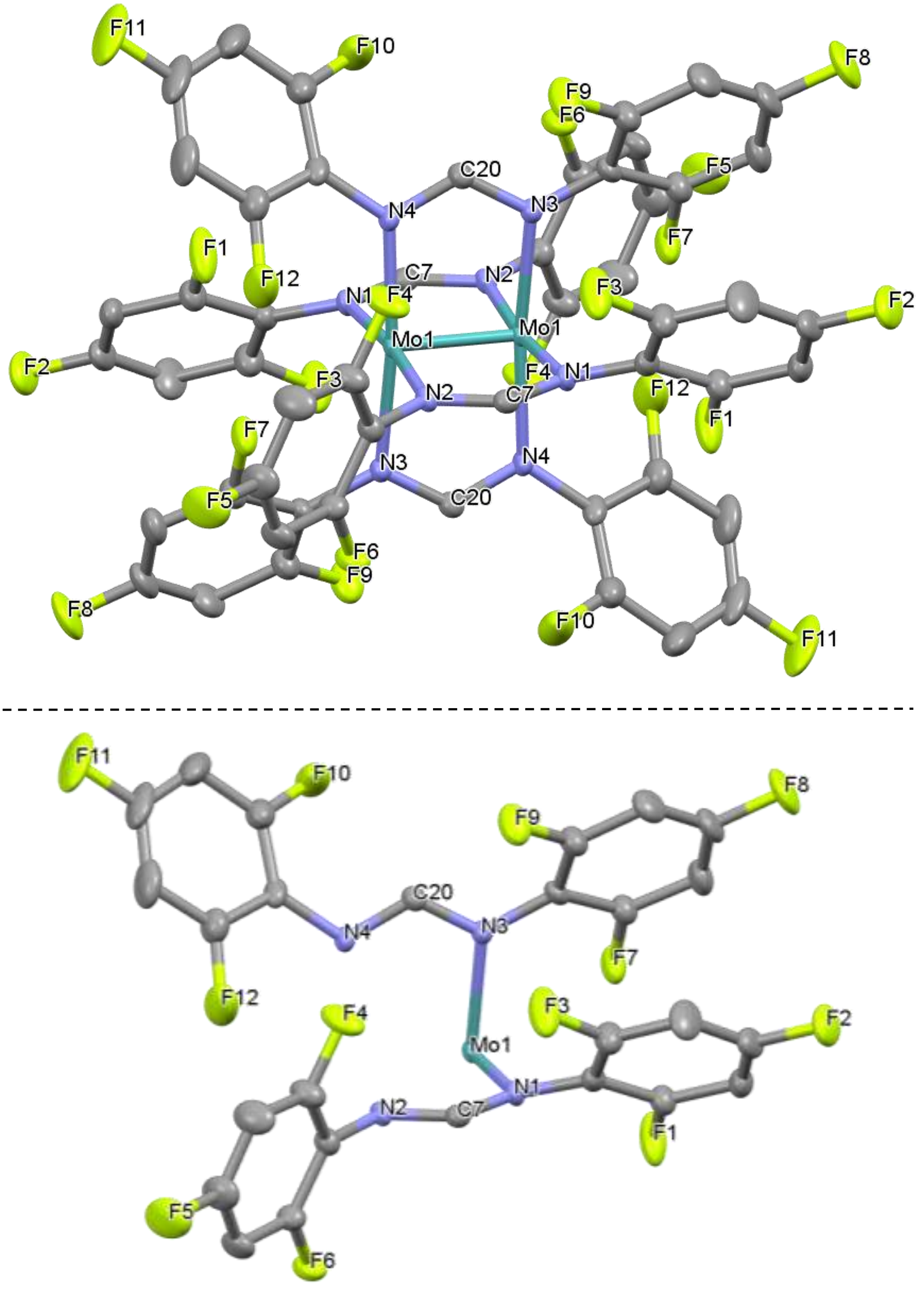

Figure S80: Single crystal XRD structure of **2f** (top) and asymmetric unit only (bottom).

Crystal Data for 2g

Empirical formula	$C_{64}H_{28}F_{28}Mo_2N_8$
Formula weight	1632.82
Temperature/K	110.00(10)
Crystal system	monoclinic
Space group	$P2_1/n$
a/Å	13.8788(3)
b/Å	25.1728(5)
c/Å	18.6471(5)
α/°	90
β/°	108.714(3)
γ/°	90
Volume/Å^3	6170.3(3)
Z	4
ρ_{calc}g/cm^3	1.758
μ/mm^{-1}	4.557
F(000)	3216.0
Crystal size/mm^3	0.134 × 0.083 × 0.056
Radiation	Cu Kα (λ = 1.54184)
2Θ range for data collection/°	6.976 to 134.158
Index ranges	-16 ≤ h ≤ 14, -30 ≤ k ≤ 27, -21 ≤ l ≤ 22
Reflections collected	24349
Independent reflections	11018 [R_{int} = 0.0562, R_{sigma} = 0.0687]
Data/restraints/parameters	11018/12/932
Goodness-of-fit on F^2	1.076
Final R indexes [I>=2σ (I)]	R_1 = 0.0638, wR_2 = 0.1574
Final R indexes [all data]	R_1 = 0.0865, wR_2 = 0.1751
Largest diff. peak/hole / e Å^{-3}	2.14/-0.89

Refinement Special Details

The crystal showed evidence of a minor non-merohederal twin estimated to be ca. 4% of the main crystal. Attempts to satisfactorily model this twinning failed due to the low abundance of the minor component. The presence of the twin is indicated by two large residual density peaks in structure of 2.14 and 1.57 electrons per cubic angstrom (Q1 & Q4) 2.111 angstroms apart which correspond to the molybdenums in the twin.

One of the difluorobenzenes of crystallisation was disordered and modelled in two positions with refined occupancies of 0.610:0.390(9). The aromatic rings were constrained to be regular hexagons with a C-C distance of 1.39 angstroms. The ADP of proximal carbons were constrained to be equal (C59 & C63A, C60 & C64A, C61 & C59A, C62 & C60A, C63 & C61A, C64 & C62A).

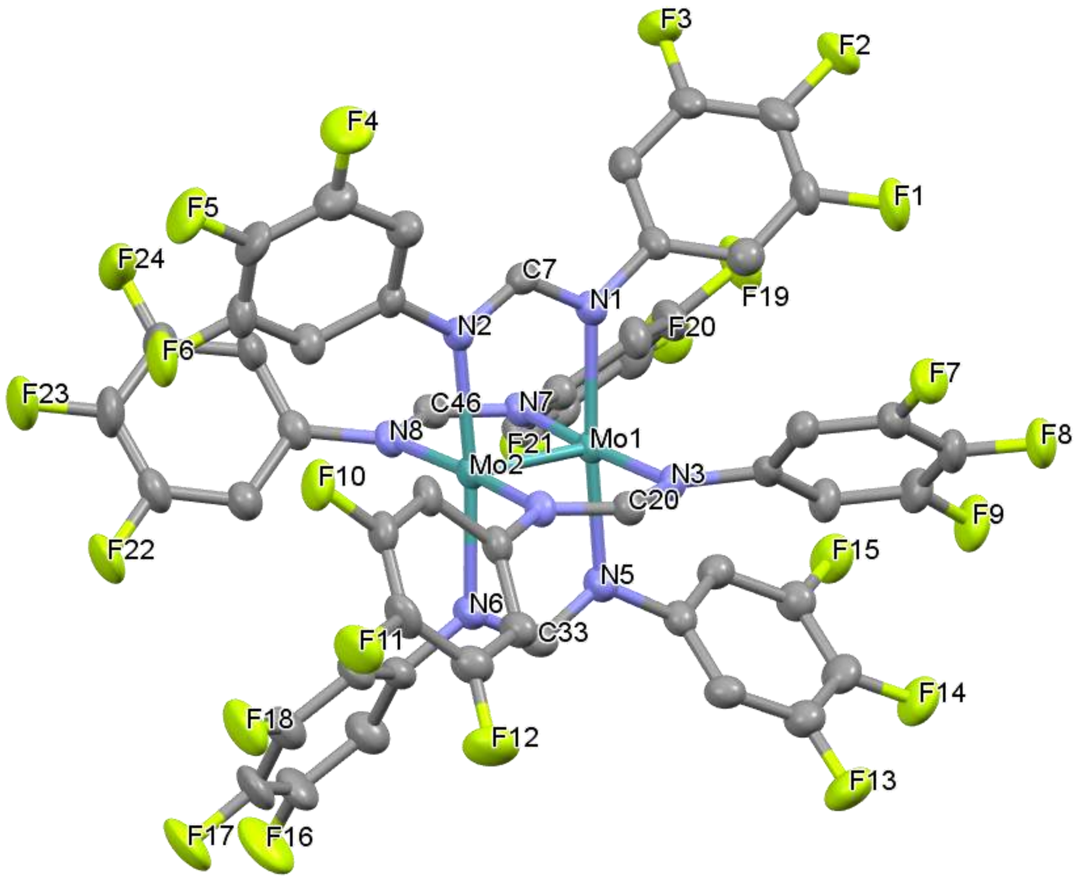

Figure S81: Single crystal XRD structure of **2g**.

Crystal Data for 2h

Empirical formula	$C_{64}H_{28}F_{40}Mo_2N_8O_3$
Formula weight	1908.82
Temperature/K	110.00(10)
Crystal system	monoclinic
Space group	$P2_1/n$
a/Å	12.4705(3)
b/Å	21.2790(4)
c/Å	13.4515(3)
α/°	90
β/°	114.253(3)
γ/°	90
Volume/Å^3	3254.44(15)
Z	2
ρ_{calc}g/cm^3	1.948
μ/mm^{-1}	4.744
F(000)	1872.0
Crystal size/mm^3	0.167 × 0.127 × 0.07
Radiation	Cu Kα (λ = 1.54184)
2Θ range for data collection/°	8.148 to 134.142
Index ranges	-14 ≤ h ≤ 13, -25 ≤ k ≤ 25, -11 ≤ l ≤ 16
Reflections collected	22300
Independent reflections	5806 [R_{int} = 0.0266, R_{sigma} = 0.0231]
Data/restraints/parameters	5806/2/557
Goodness-of-fit on F^2	1.015
Final R indexes [I>=2σ (I)]	R_1 = 0.0291, wR_2 = 0.0739
Final R indexes [all data]	R_1 = 0.0340, wR_2 = 0.0772
Largest diff. peak/hole / e Å^{-3}	1.19/-0.55

Refinement Special Details

The asymmetric unit contained 0.5 of the complex and 1.5 THFs. One THF was disordered with 4 atoms (C28-C30 & O1) modelled in two positions with refined occupancies of 0.708:0.282(6). Each pair of C-O bonds were restrained to be equal (C27-O1 & C27-O1a, C30-O1 & C30a-O1a). The ADP of each pair of disordered atoms were constrained to be equal (C28 & C28a, etc). The other THF was half occupied per asymmetric unit being disordered about an inversion centre. The ADPs of C31 and C33 were constrained to be equal.

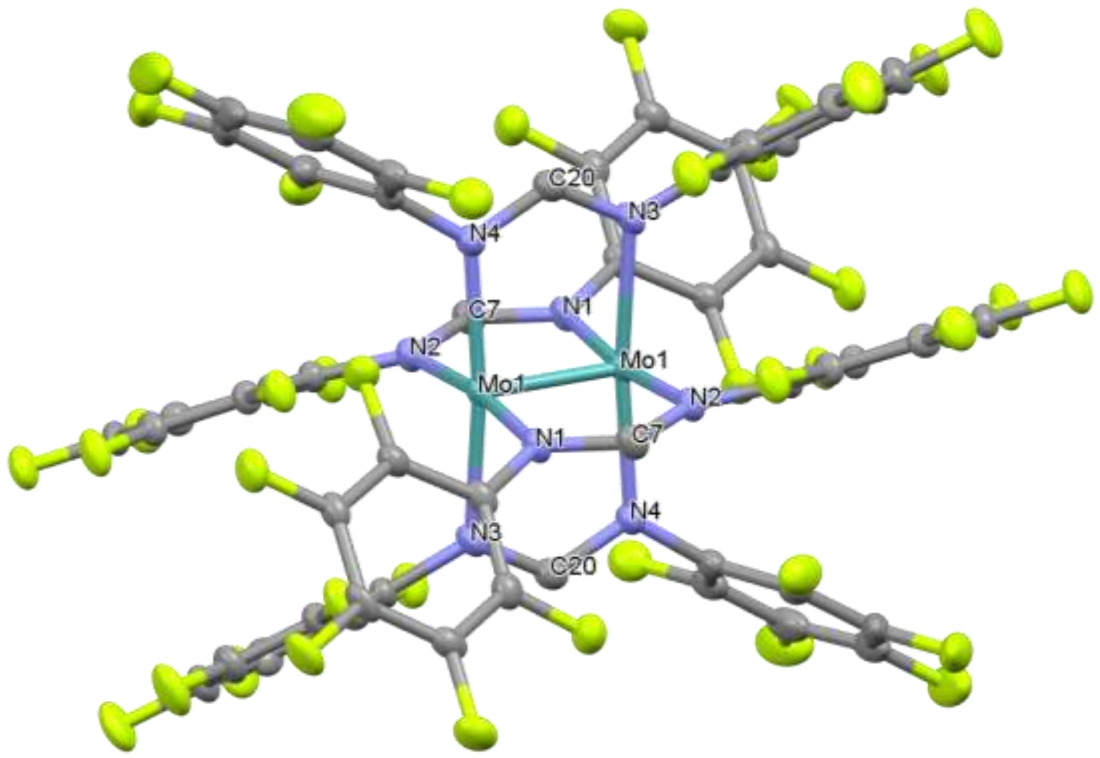

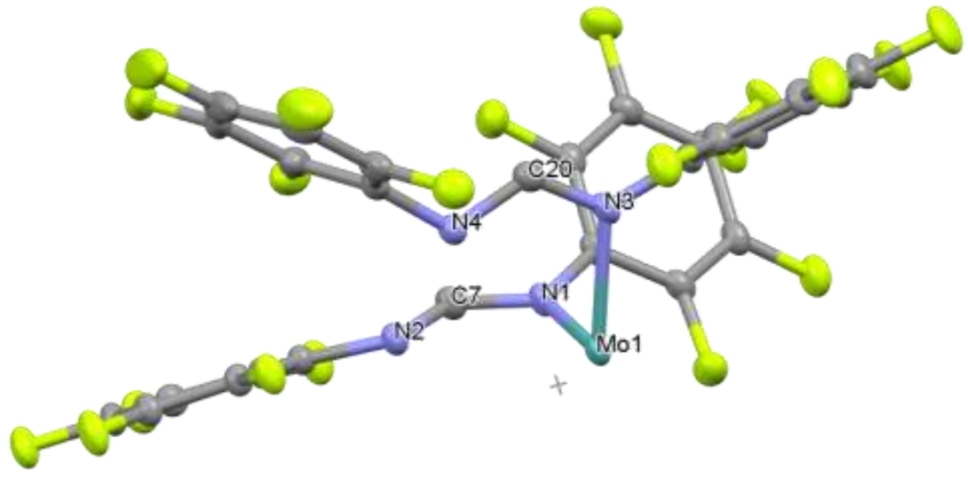

Figure S82: Single crystal XRD structure of **2h** (top) and asymmetric unit only (bottom).

Crystal Data for 2i

Empirical formula	$C_{64}H_{44}F_{24}Mo_2N_8O$
Formula weight	1588.95
Temperature/K	110.00(10)
Crystal system	tetragonal
Space group	I-42d
a/Å	29.0026(2)
b/Å	29.0026(2)
c/Å	29.8755(4)
α/°	90
β/°	90
γ/°	90
Volume/Å^3	25129.7(5)
Z	16
ρ_{calc}g/cm^3	1.680
μ/mm^{-1}	4.369
F(000)	12672.0
Crystal size/mm^3	0.223 × 0.155 × 0.144
Radiation	Cu Kα (λ = 1.54184)
2Θ range for data collection/°	7.322 to 134.154
Index ranges	-34 ≤ h ≤ 26, -30 ≤ k ≤ 32, -35 ≤ l ≤ 27
Reflections collected	44059
Independent reflections	10960 [R_{int} = 0.0351, R_{sigma} = 0.0302]
Data/restraints/parameters	10960/354/979
Goodness-of-fit on F^2	1.059
Final R indexes [I>=2σ (I)]	R_1 = 0.0915, wR_2 = 0.1532
Final R indexes [all data]	R_1 = 0.1317, wR_2 = 0.1745
Largest diff. peak/hole / e Å^{-3}	1.38/-1.22
Flack parameter	0.47(5)

Refinement Special Details

The structure was twinned and disordered. The twinning was modelled with an inversion matrix with a ratio of 0.53:0.47(5) for the components. Four of the trifluoromethyl groups were disordered. For two, the whole group was modelled in two positions in refined occupancies of 0.661:0.339(18) and 0.659: 0.341(12). For the other two, the trifluoromethyl group was modelled in two positions in refined ratios of 0.658:0.342(18) and 0.519:0.481(18).

The ADP of several groups of atoms were constrained to be equal (C14 & C14A, C31 & C31A, C32 & C32A, C33 & C33A, C34 & C34A, C35 & C35A, C36 & C36A, C37 & C37A, C38 & C38A, C39 & C39A, C40 & C40A, C41 & C41A, C42 & C42A, C43 & C43A, C44A F16 & C44, F17A & C61A, C63B & C64B, C63A & C61B, C61A C64B C63B & O1B, F4 & F4A, F5 & F5A, F18 & F18A, F17 & F16A, F6 & F6A, F14A & F15, N2 & N4). The phenyl rings C31A-C36A, C41A-C46A were constrained to be regular hexagons with a C-C bond length of 1.39 angstroms. Some C-F bond lengths were restrained to be equal (those for

C14, C14a, C22, C22a, C37a, C44a) with the F-F distances restrained to be equal with each of the trifluoromethyl groups. The C-CF3 bond lengths were restrained to be 1.54 angstroms for C19-C22, C41-C44, C41a-C44A. Atoms C17, C18, C19, C20, C21 & C22 were restrained to be in the same plane. The THF was modelled in two positions with equal occupancy. C-C bond lengths were constrained to be 1.5 angstroms, C-O bond lengths to 1.42 angstroms, C-O-C distances to be 2.32 angstroms, C-C-O distances to 2.34 angstroms and C-C-C bond distances to 2.36 angstroms.

The ADP of a large number of atoms were restrained to be approximately isotropic.

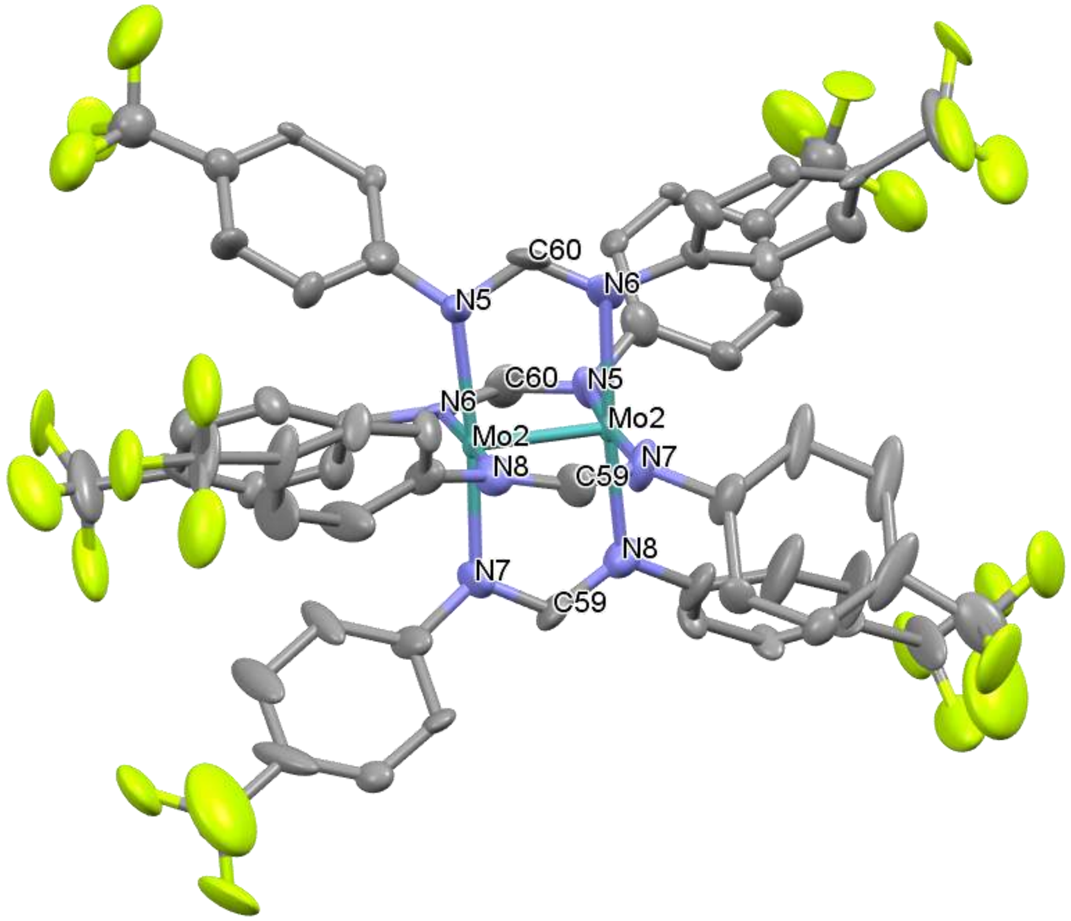

Figure S83: Single crystal XRD structure of **2i**.

Crystal Data for 2j

Empirical formula	$C_{160}H_{104}F_{96}Mo_4N_{16}O_6$
Formula weight	4554.35
Temperature/K	109.95(10)
Crystal system	orthorhombic
Space group	$Pca2_1$
a/Å	26.9128(2)
b/Å	18.83918(14)
c/Å	36.7739(4)
α/°	90
β/°	90
γ/°	90
Volume/Å^3	18644.9(3)
Z	4
ρ_{calc}g/cm^3	1.622
μ/mm^{-1}	3.545
F(000)	9024.0
Crystal size/mm^3	0.18 × 0.123 × 0.083
Radiation	Cu Kα (λ = 1.54184)
2Θ range for data collection/°	6.996 to 134.154
Index ranges	-32 ≤ h ≤ 29, -16 ≤ k ≤ 22, -43 ≤ l ≤ 43
Reflections collected	70061
Independent reflections	27609 [R_{int} = 0.0290, R_{sigma} = 0.0373]
Data/restraints/parameters	27609/125/2615
Goodness-of-fit on F^2	1.047
Final R indexes [I>=2σ (I)]	R_1 = 0.0431, wR_2 = 0.0999
Final R indexes [all data]	R_1 = 0.0473, wR_2 = 0.1024
Largest diff. peak/hole / e Å^{-3}	0.41/-0.83
Flack parameter	0.389(8)

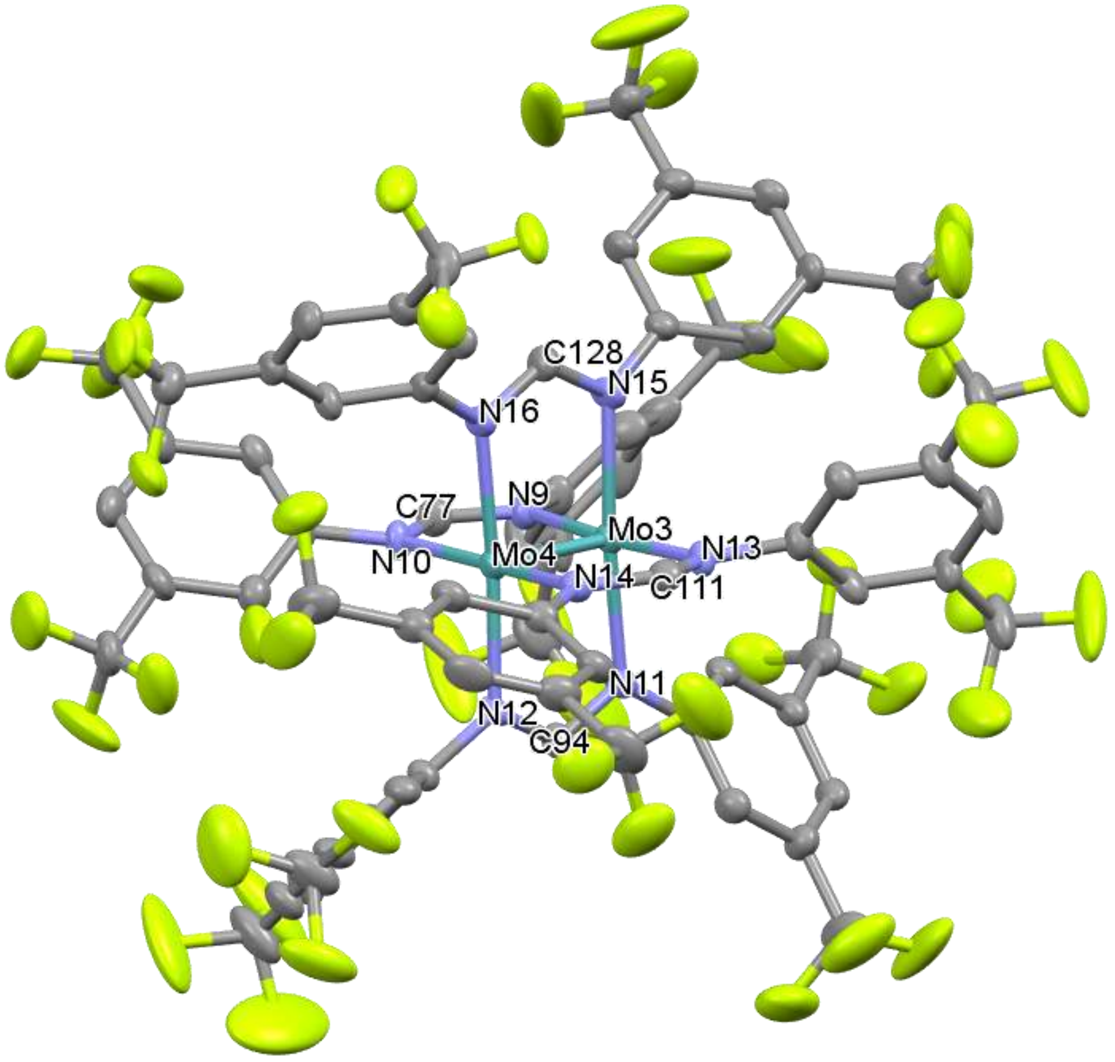

Figure S84: Single crystal XRD structure of **2j**. A single moiety of **2j** was selected from the unit cell for visualization

Electrochemistry

All voltammograms are reported in THF with NBu_4PF_6 (0.1 M) as the supporting electrolyte.

Compound 2b

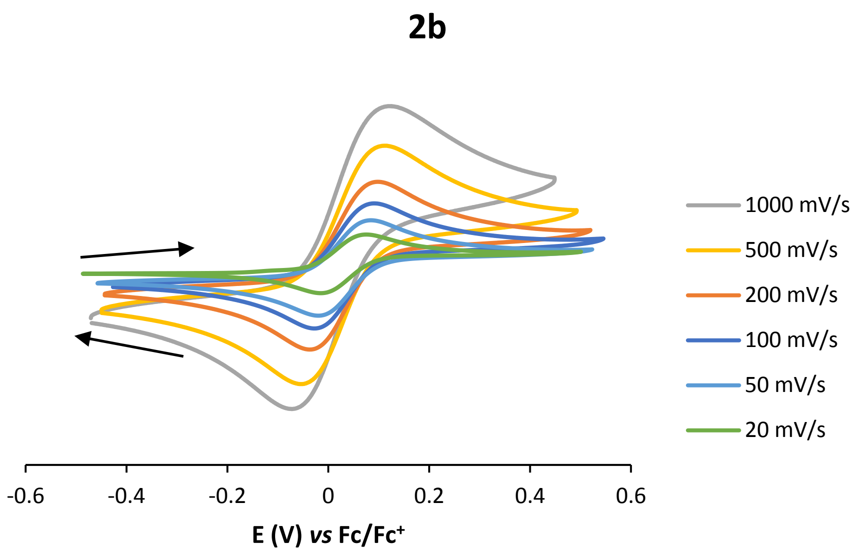

Figure S85: Cyclic voltammogram of compound **2b** at various scan rates

Compound 2c

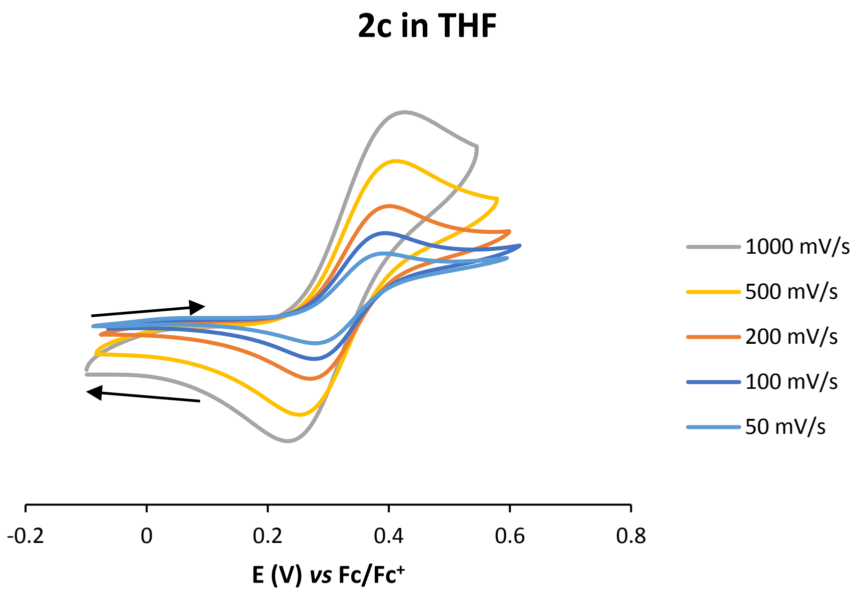

Figure S86: Cyclic voltammogram of compound **2c** at various scan rates

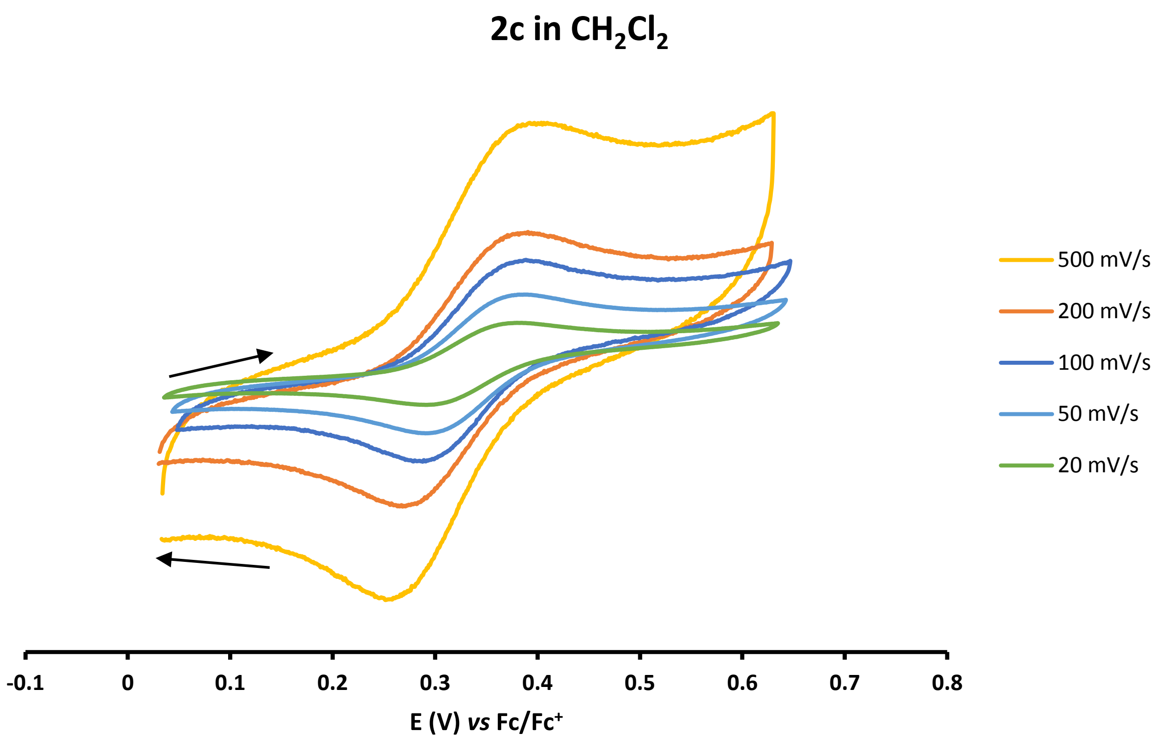

Figure S87: Cyclic voltammogram of compound **2c** in CH_2Cl_2 at various scan rates

Compound 2d

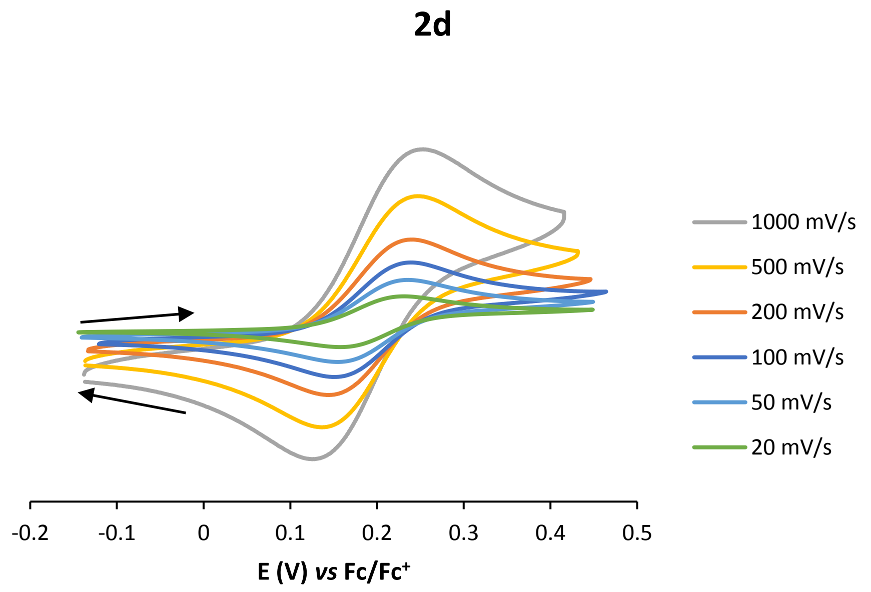

Figure S88: Cyclic voltammogram of compound **2d** at various scan rates

Compound 2e

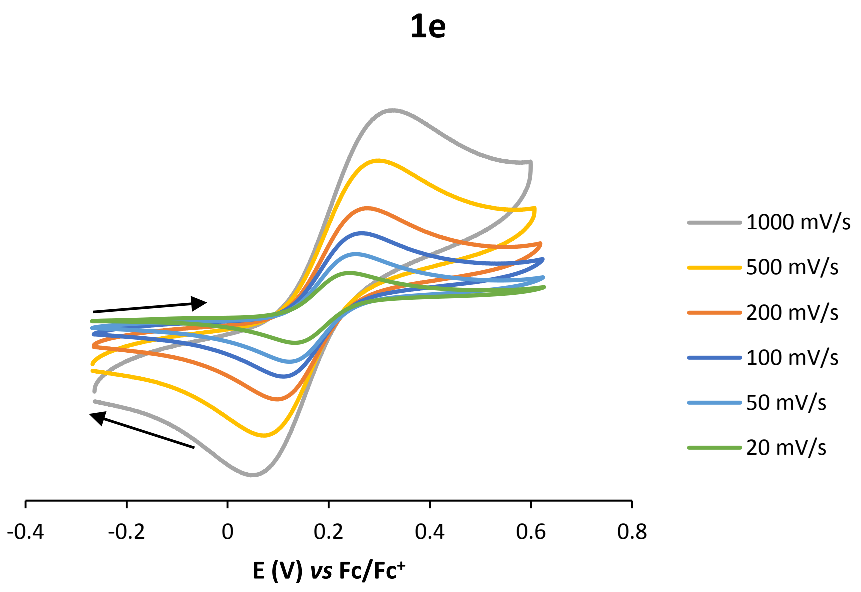

Figure S89: Cyclic voltammogram of compound **2e** at various scan rates

Compound 2f

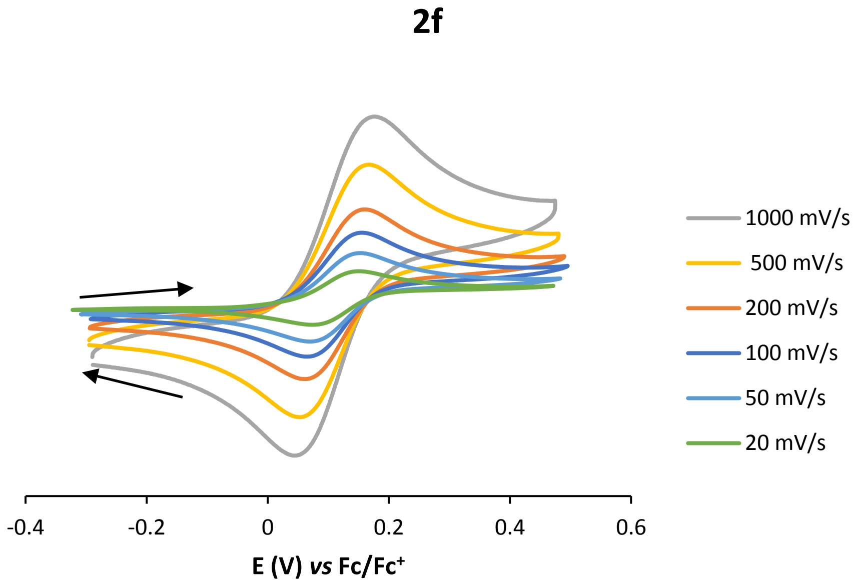

Figure S90: Cyclic voltammogram of compound **2f** at various scan rates

Compound 2g

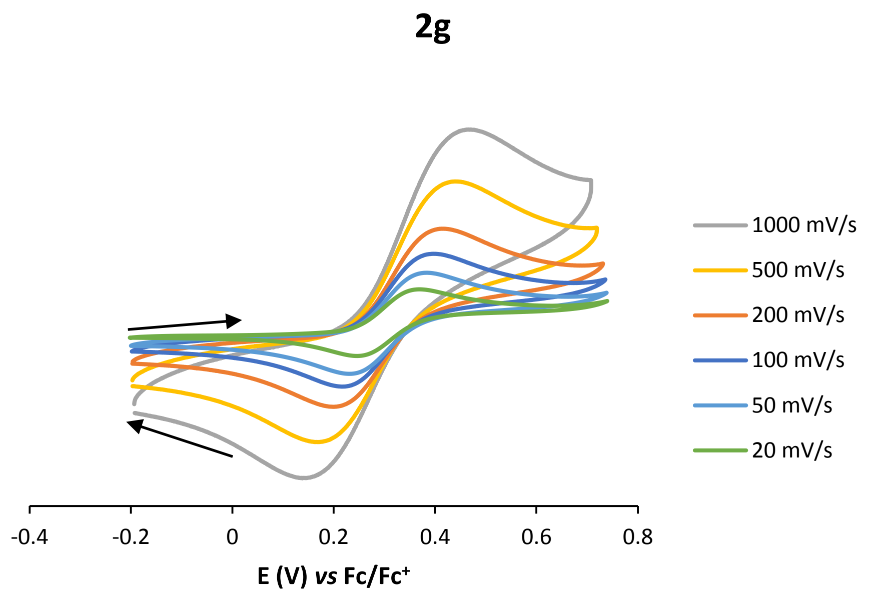

Figure S91: Cyclic voltammogram of compound **2g** at various scan rates

Compound 2i

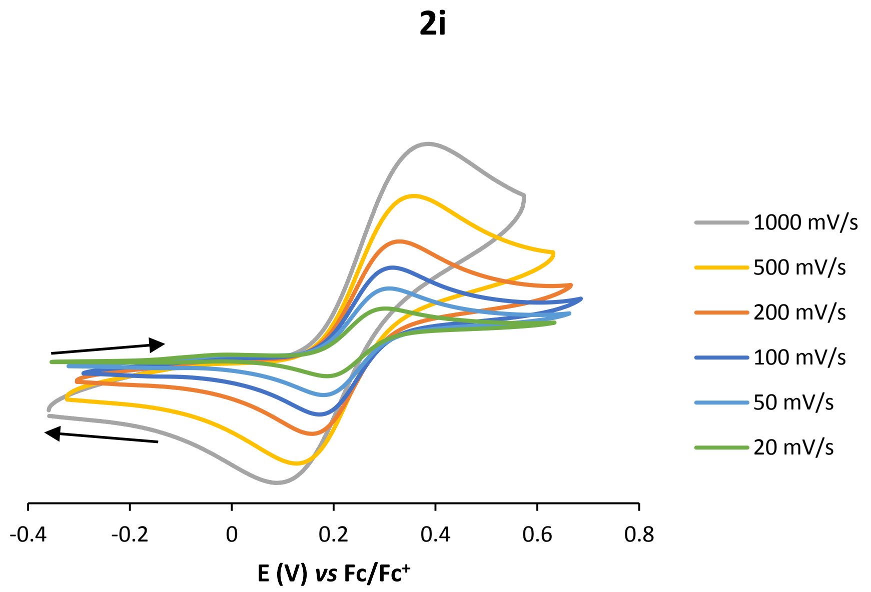

Figure S92: Cyclic voltammogram of compound **2i** at various scan rates

Compound 3c

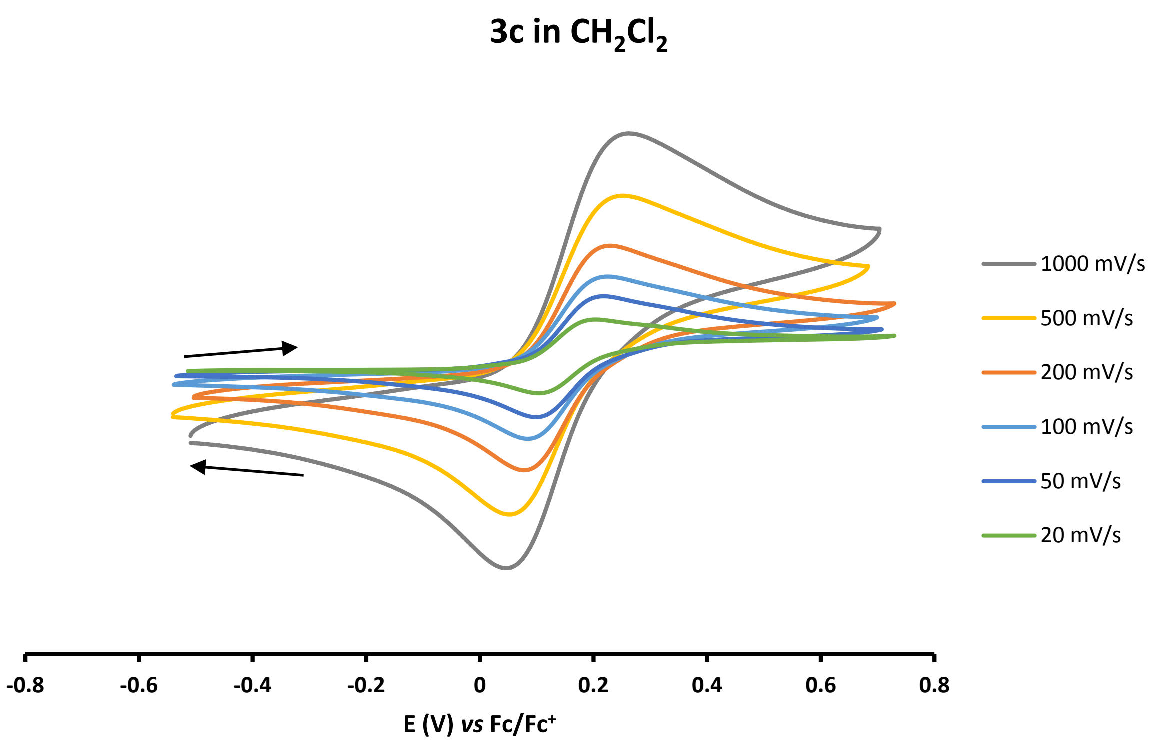

Figure S93: Cyclic voltammogram of compound **3c** at various scan rates

Compound 2c vs 3c in DCM

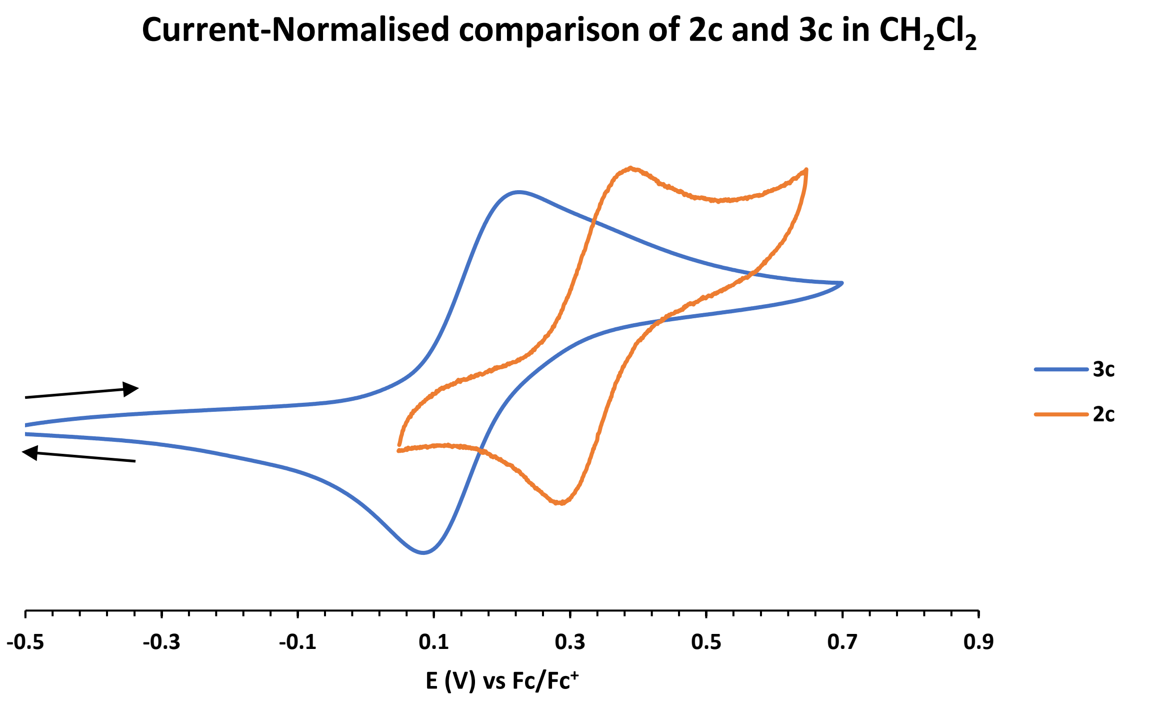

Figure S94: Cyclic voltammogram of compound **2c** and **3c** in CH_2Cl_2. The current response has been normalized to aid interpretation

Quantum Chemical Calculations

General Computational Details

Initial geometry optimisation calculations were performed at the B3LYP/def2_SV(P) level, followed by frequency calculations at the same level. Local minima were identified by the absence of imaginary frequency vibrations. In the (RI-)BP86/def2_SV(P) calculations, a 60 electron quasi-relativistic ECP replaced the core electrons of Mo. No symmetry constraints were applied during optimisations. All optimisation calculations were performed using the Gaussian 16 revision A.03 package.[6]

Single point energy calculations were performed using the Gaussian 16 revision A.03 package. Single-point calculations on the B3LYP/SV(P) optimised geometries were performed using the hybrid PBE0 functional and the flexible def2-TZVPP basis set. NBO analysis performed on the previously optimized geometry at the PBE0/def2_TZVP level of theory using the NBO 7.0 package. [7]

This project was undertaken on the Viking Cluster, which is a high-performance computer facility provided by the University of York. We are grateful for computational support from the University of York High Performance Computing service, Viking and the Research Computing team.

Molecular Orbital Diagram

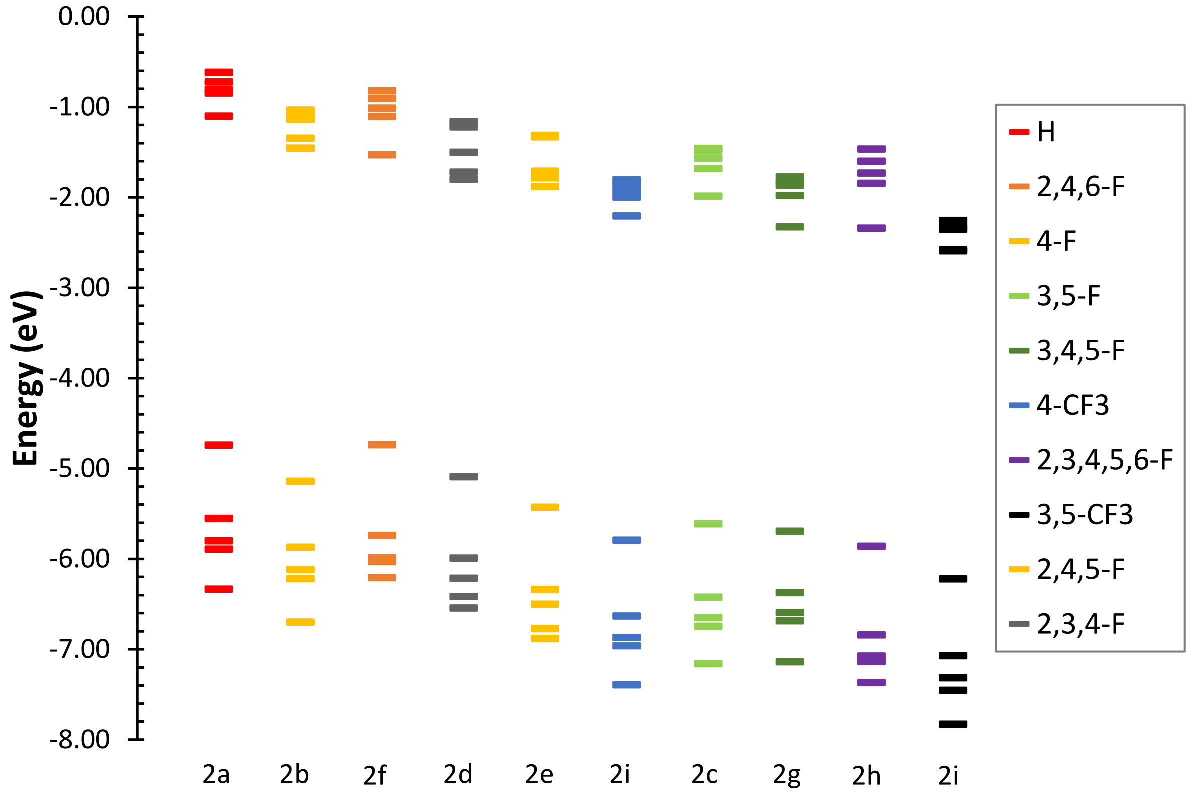

Figure S95: Molecular orbital energy level diagram for compounds **2a-2i**

Compound 2a

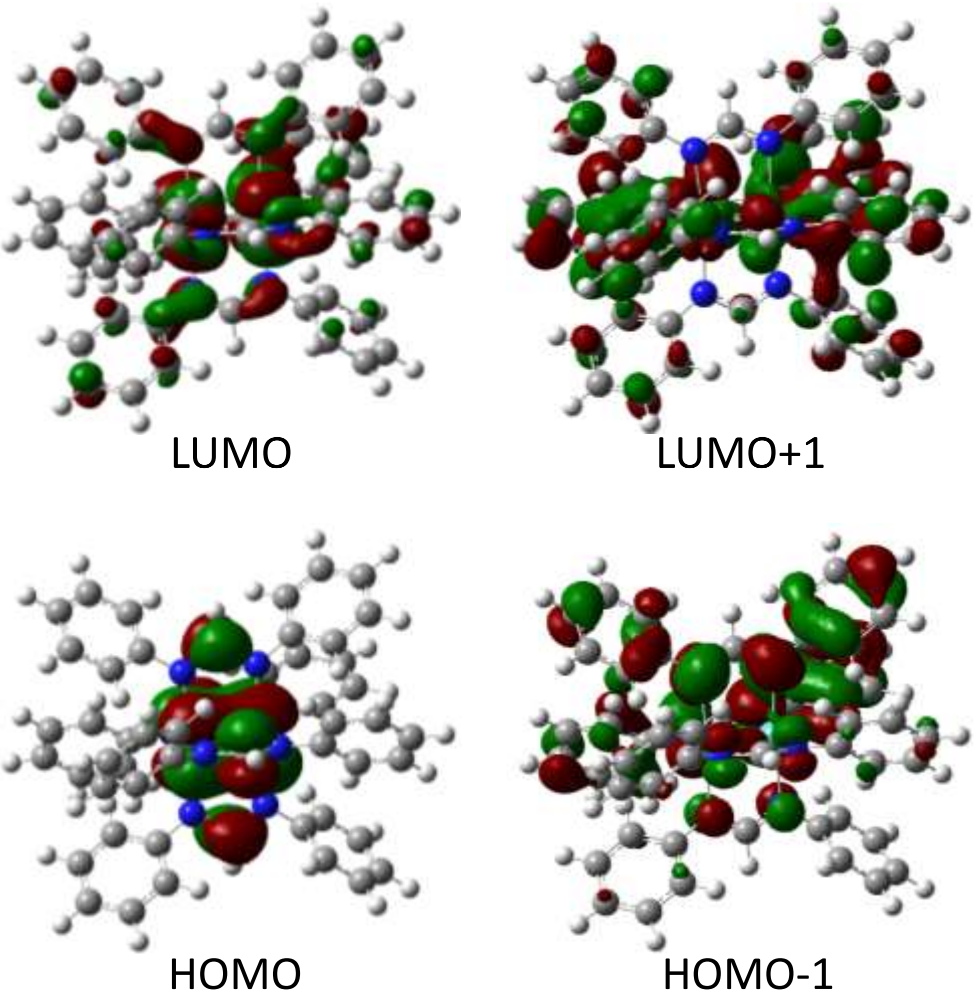

Figure S96: Frontier molecular orbitals for **2a**

Compound 2b

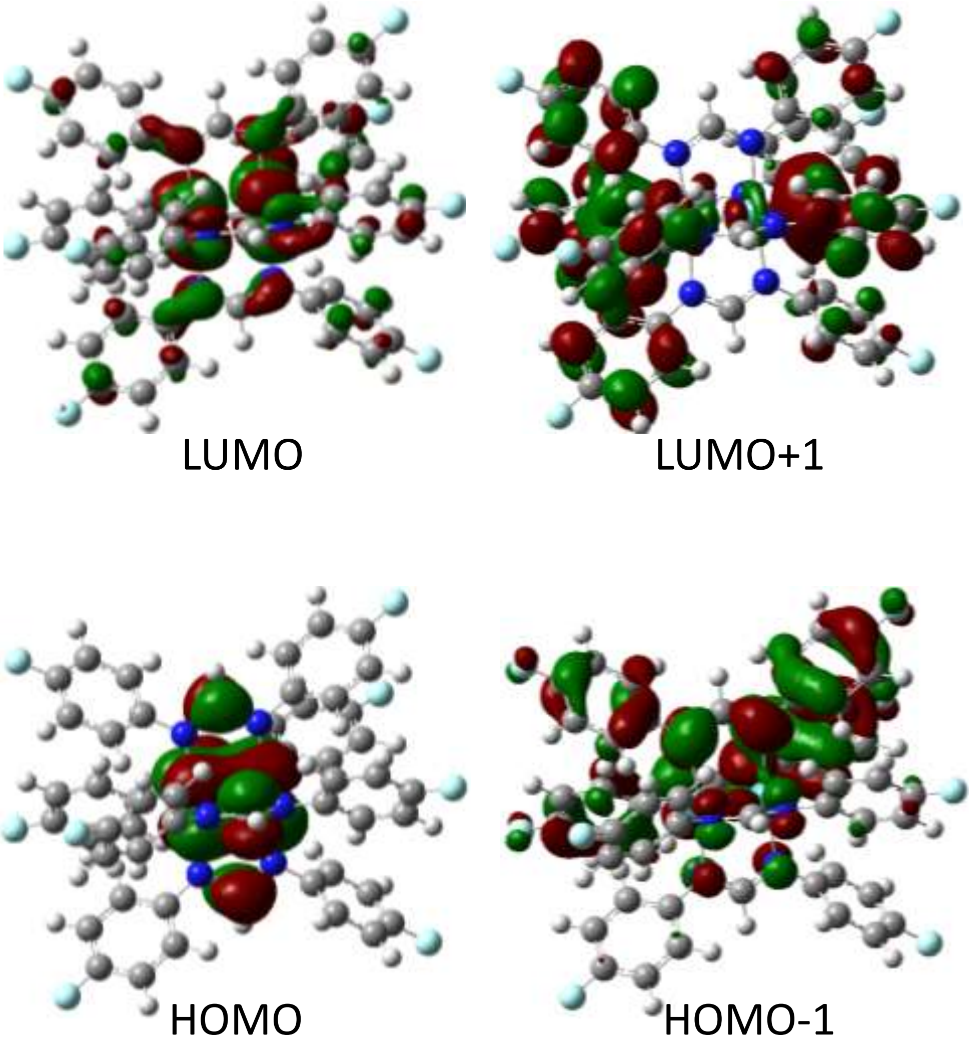

Figure S97: Frontier molecular orbitals for **2b**

Compound 2c

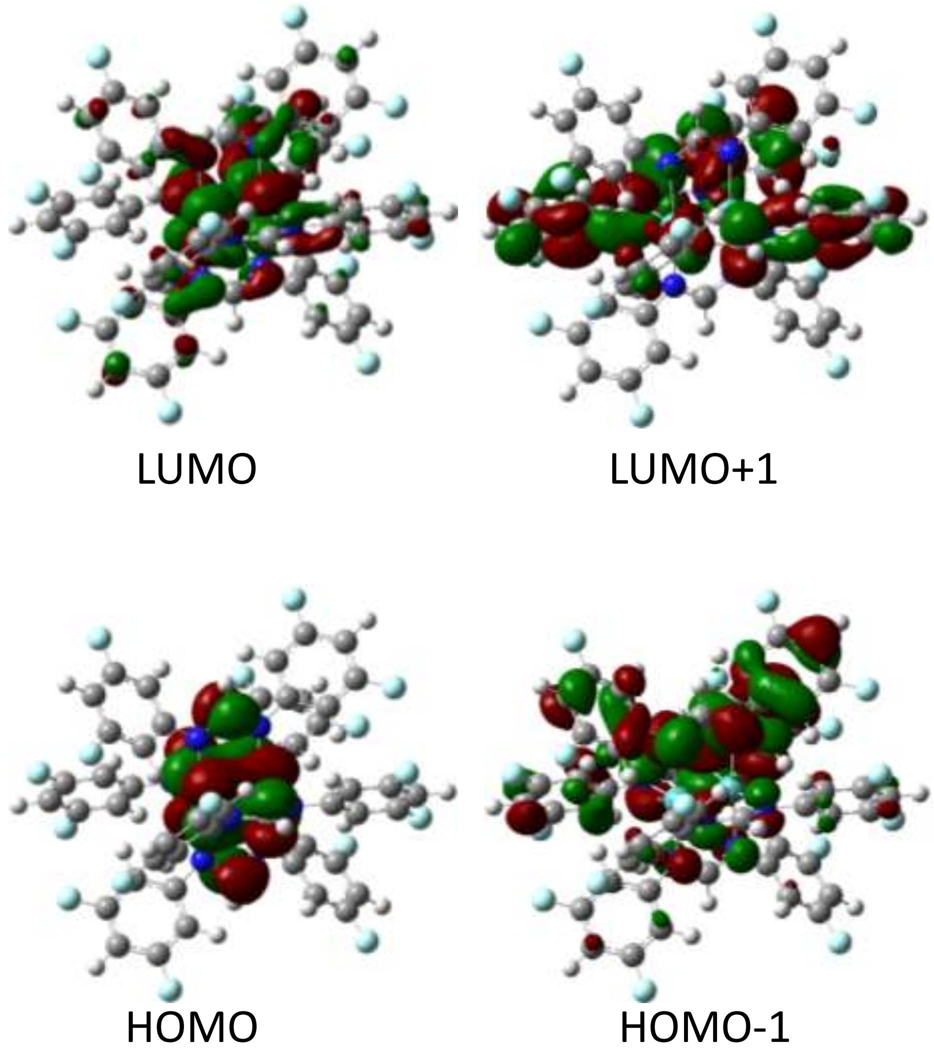

Figure S98: Frontier molecular orbitals for **2c**

Compound 2d

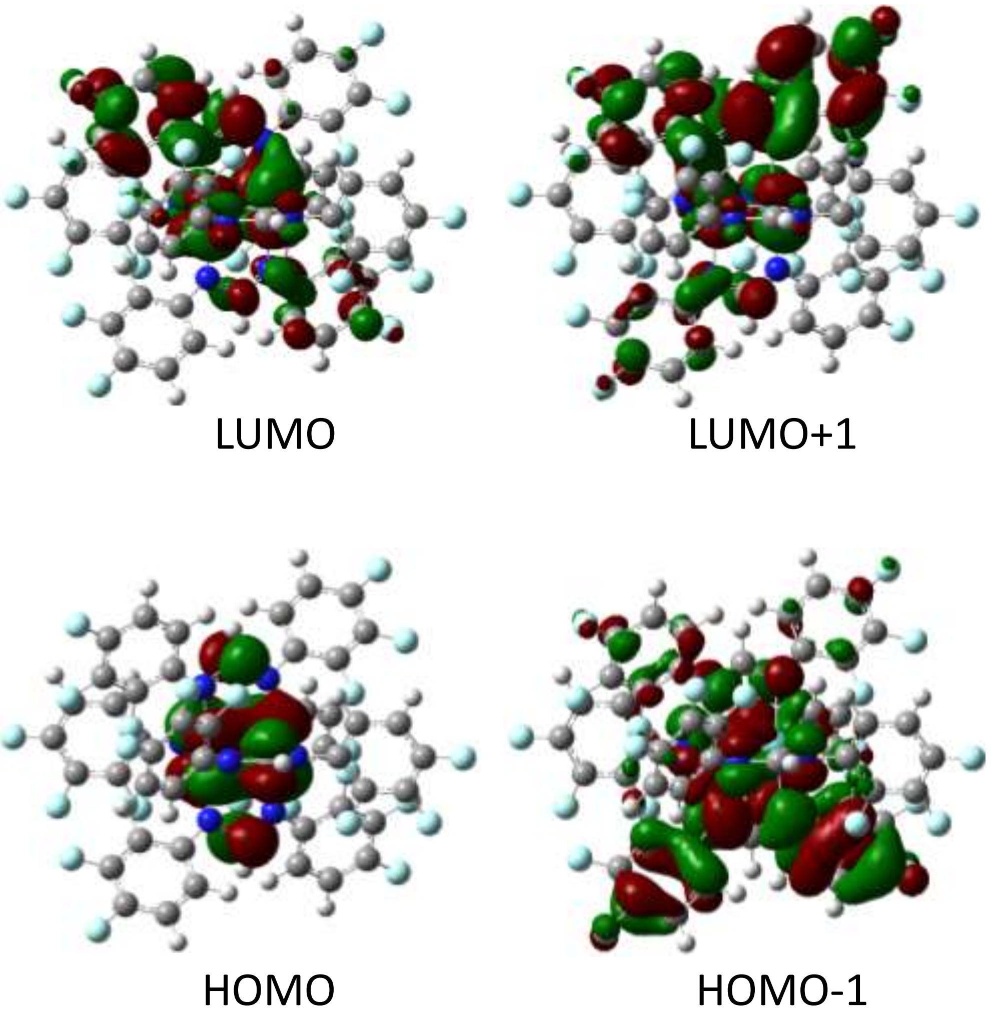

Figure S99: Frontier molecular orbitals for **2d**

Compound 2e

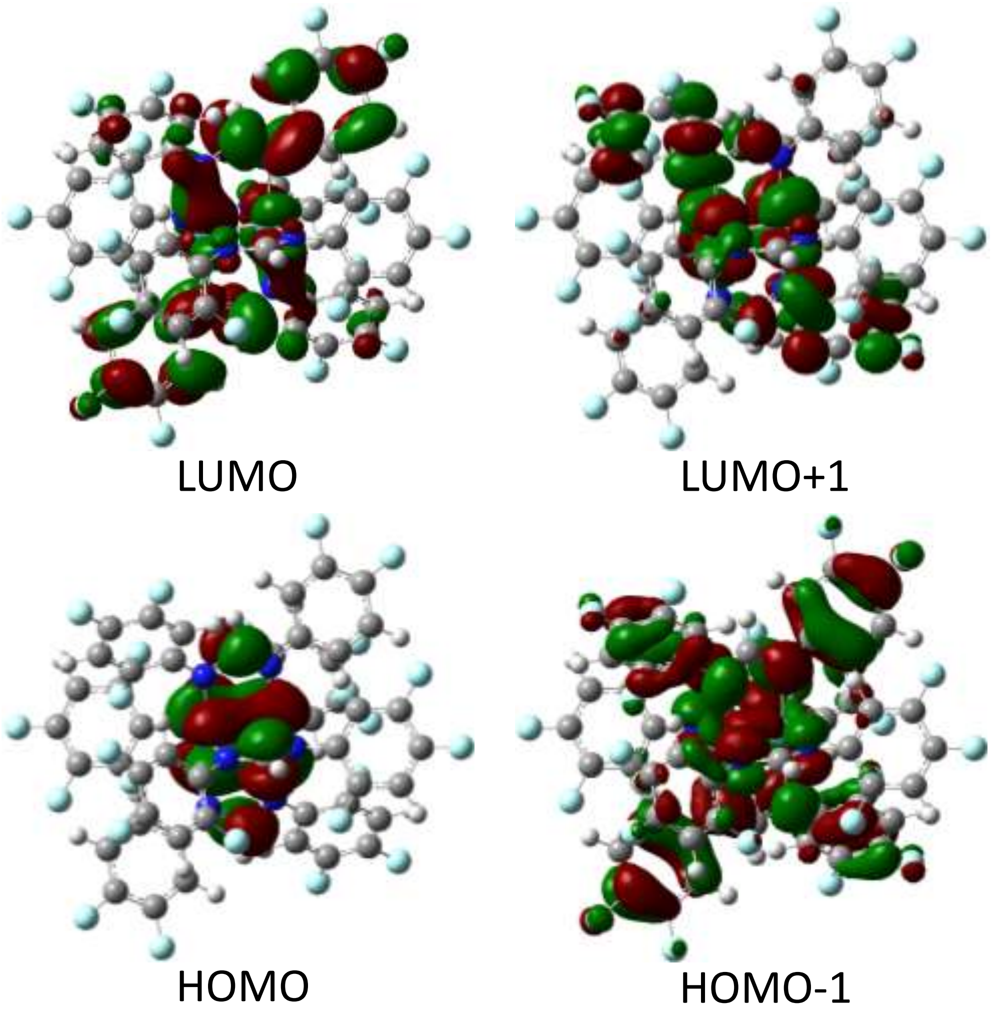

Figure S100: Frontier molecular orbitals for **2e**

Compound 2f

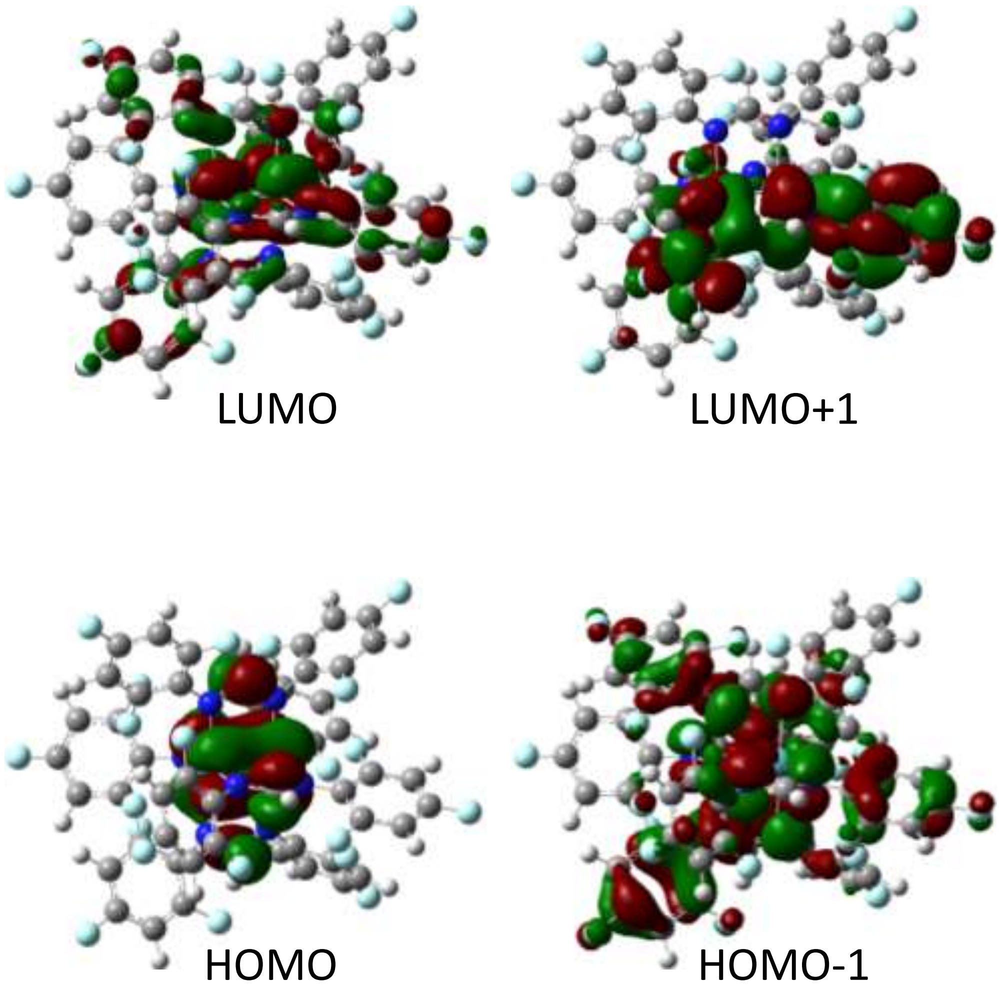

Figure S101: Frontier molecular orbitals for **2f**

Compound 2g

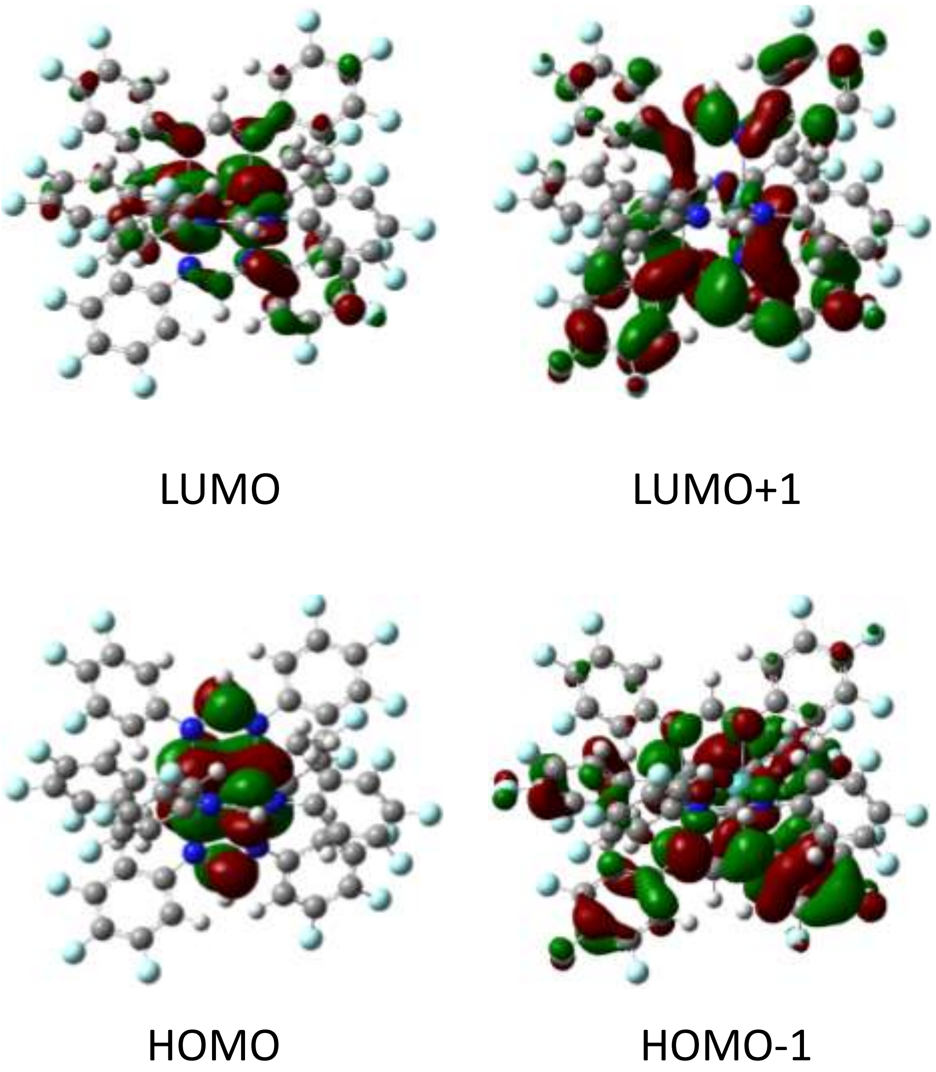

Figure S102: Frontier molecular orbitals for **2g**

Compound 2h

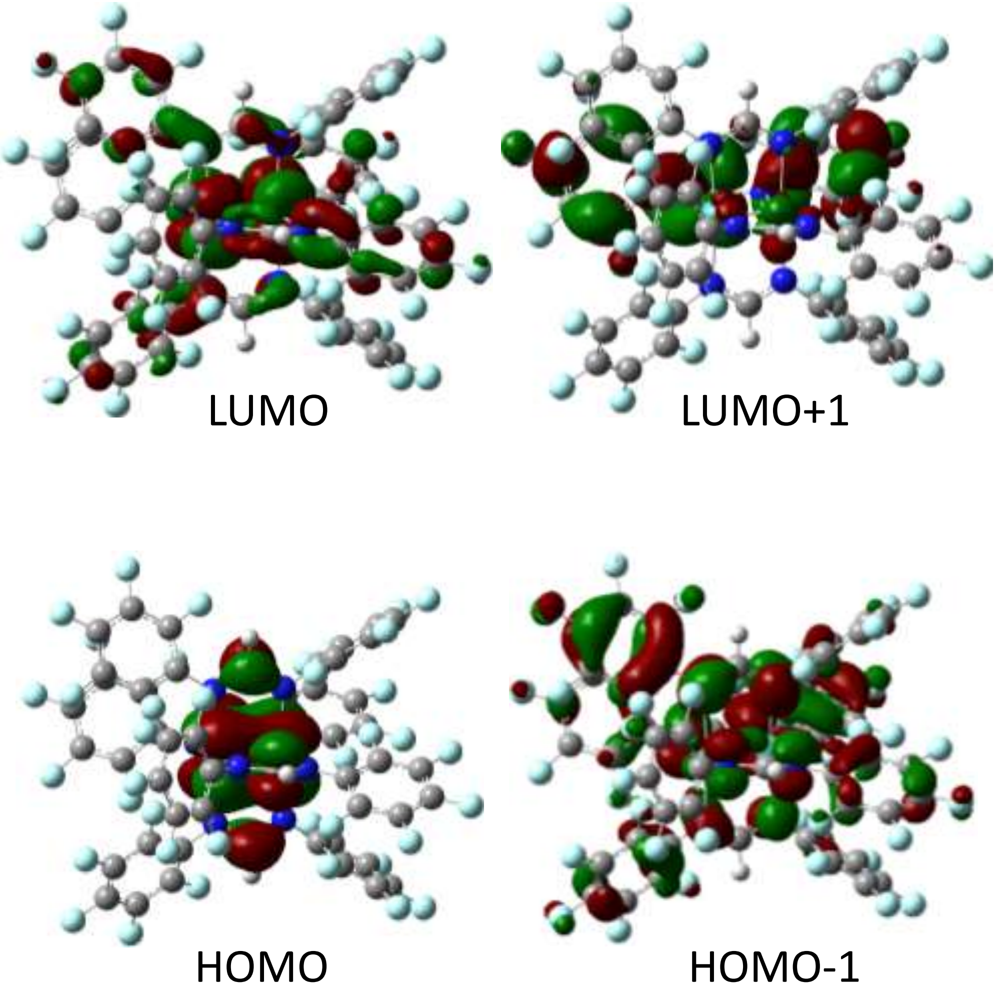

Figure S103: Frontier molecular orbitals for **2h**

Compound 2i

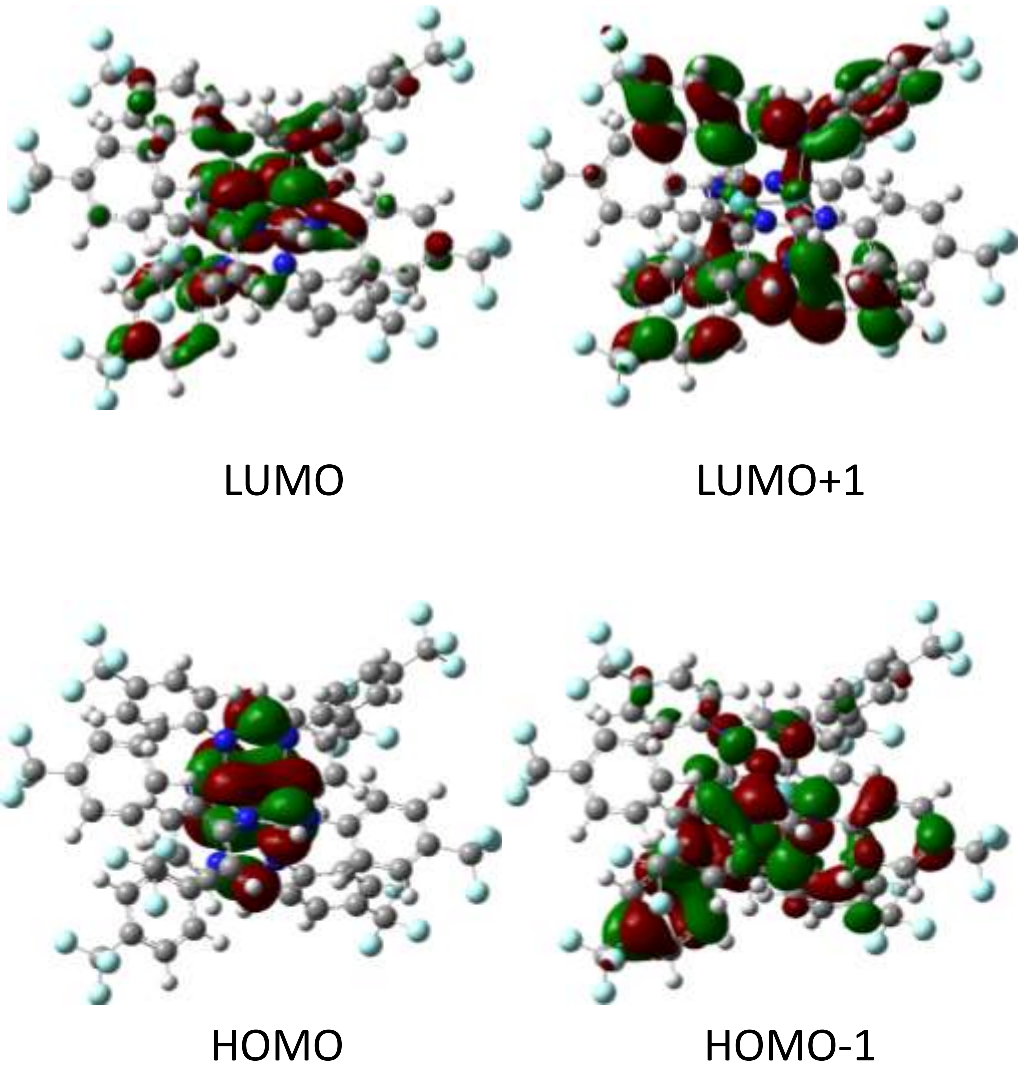

Figure S104: Frontier molecular orbitals for **2i**

Compound 2j

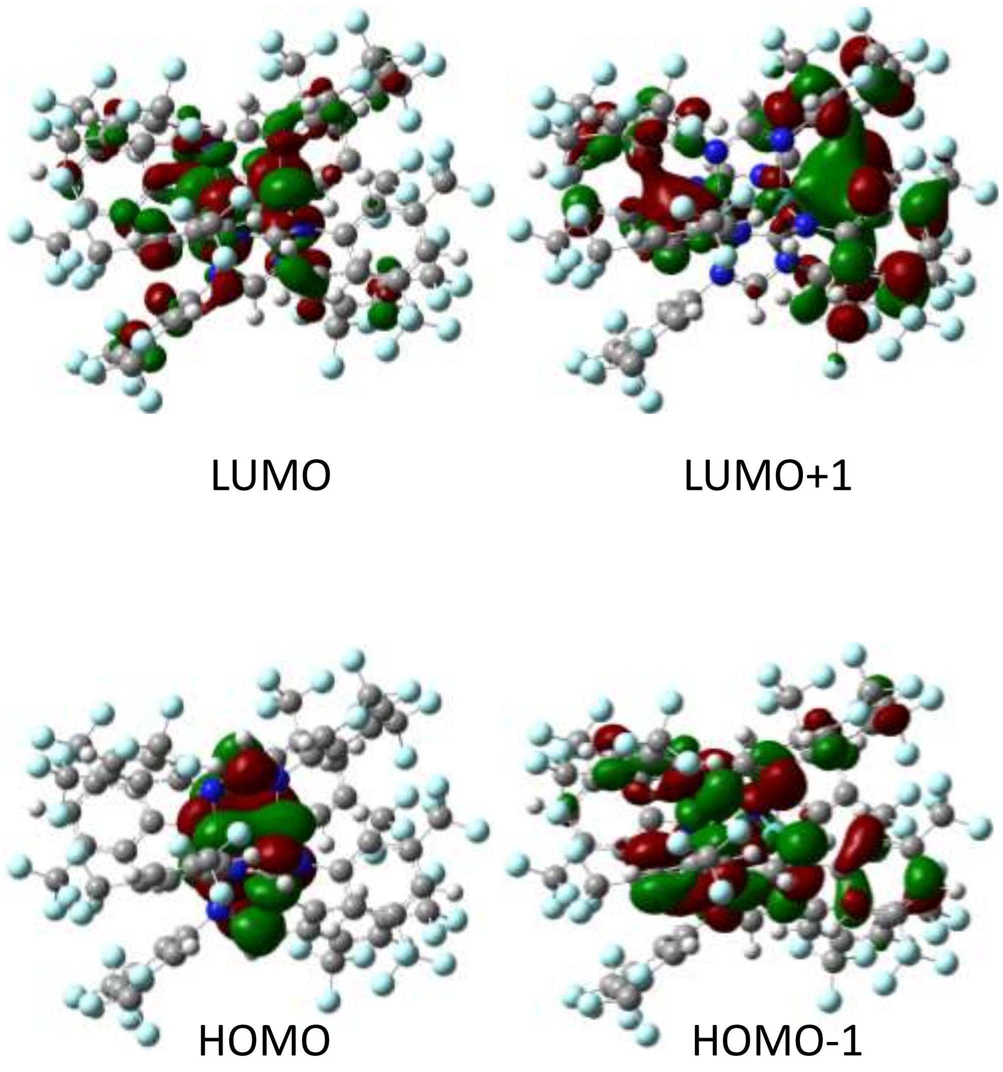

Figure S105: Frontier molecular orbitals for **2j**

Compound 3c

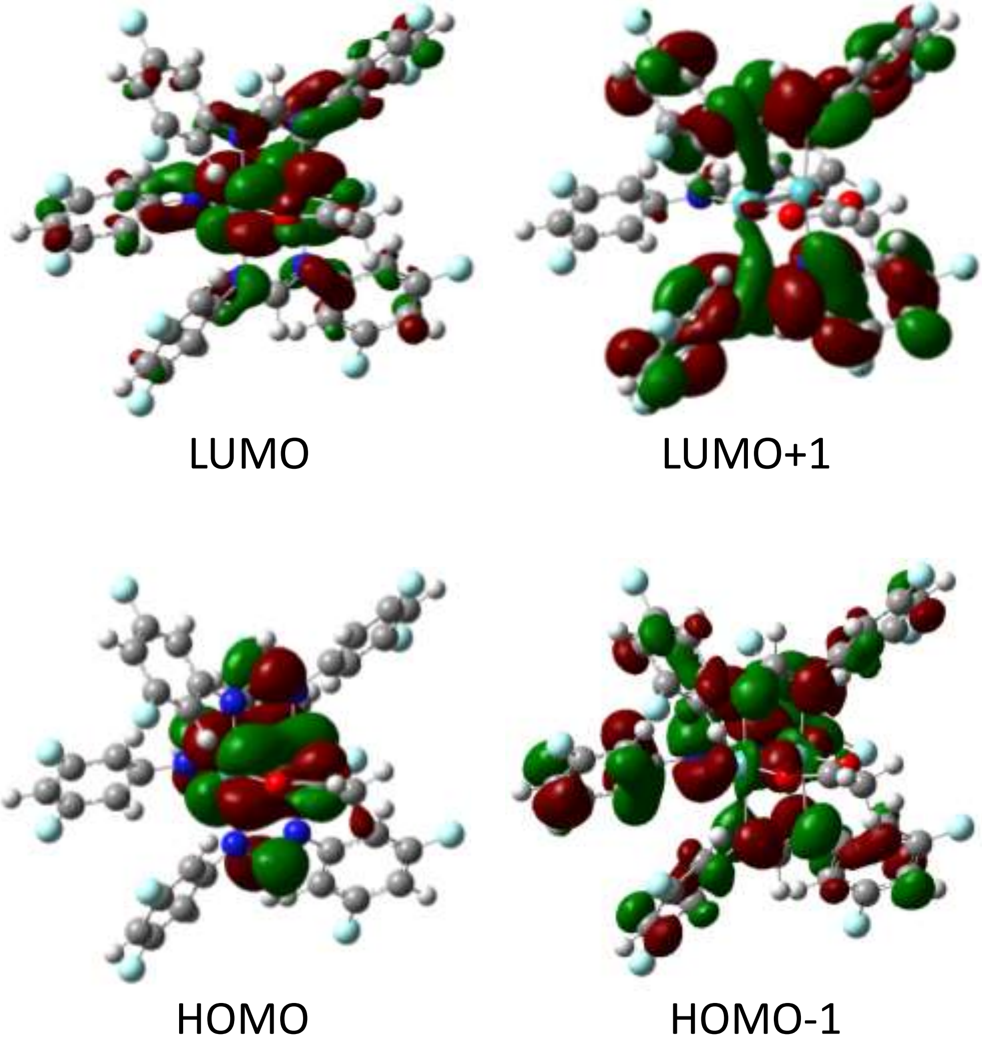

Figure S106: Frontier molecular orbitals for **3c**

Evaluating stability to O_2

Details for calculating concentrations in Figures S109-112 and S105-119

Stock solutions were prepared by adding the internal standard 1,3,5-tris(trifluoromethyl)benzene (2 µL, 1.514 g mL^{-1}, 3.028 mg, 1.073×10^{-5} mol) to 1 mL of either $CDCl_3$ or $(CD_3)_2CO$, giving a concentration of 1.073×10^{-2} mol dm^{-3}.

Each NMR sample was prepared with 0.7 mL of stock solution ($CDCl_3$ or $(CD_3)_2CO$) and therefore contained 7.51 mol of internal standard.

In the NMR spectra, integration of the resonance for the internal standard was always normalized to 1 and the integral for the analyte was compared to this. The integral for the analyte and the internal standard were reduced to their molecular stoichiometries by dividing their integral by the number of nuclei from that environment (denoted in columns C and E, Tables S1-S5).

The ratio between the analyte and internal standard (Column **F**) was obtained by dividing the former by the latter.

The number of moles of analyte in the sample were determined by first relating the integral of the internal standard to the number of moles in the sample. This was then multiplied by the stoichiometry of the analyte; (7.51 mol / Column **C**)*(Column **E**) = moles of analyte

The concentration was then determined knowing that the volume of the sample was 0.7 mL

Table S1: Calculations of concentrations *vs* time for **2b** from ^{1}H NMR spectra

A	B	C	D	E	F	G	H	I
Time (h)	Integral of standard @ 8.44 ppm	Stoic. of standard (3H)	Integral @ 6.76 ppm	Stoic. of analyte (16H)	Ratio analyte :standard	Moles of analyte (mol)	Conc. of analyte (mol dm^{-3})	ln(conc)
1	1	0.333	1.03	0.064	0.193	1.45×10^{-6}	2.07×10^{-3}	-6.18
3	1	0.333	0.82	0.051	0.154	1.16×10^{-6}	1.65×10^{-3}	-6.40
7	1	0.333	0.66	0.041	0.124	9.30×10^{-7}	1.33×10^{-3}	-6.62
29	1	0.333	0.21	0.013	0.039	2.96×10^{-7}	0.42×10^{-3}	-7.77
49	1	0.333	0.20	0.013	0.038	2.82×10^{-7}	$0. 40 \times 10^{-3}$	-7.82

Table S2: Calculations of concentrations *vs* time for **2b** from ^{19}F NMR spectra

Time (h)	Integral of standard @ 63.4 ppm	Stoic. of standard (9F)	Integral @ 121.6 ppm	Stoic. of analyte (8F)	Ratio analyte :standard	Moles of analyte (mol)	Conc. of analyte (mol dm^{-3})	ln(conc)
1	1	0.111	0.112	0.014	0.126	8.52x10^{-6}	1.22x10^{-2}	-4.41
3	1	0.111	0.095	0.012	0.107	7.26x10^{-6}	1.04 x10^{-2}	-4.57
7	1	0.111	0.075	0.009	0.084	5.68x10^{-6}	0.81x10^{-2}	-4.81
29	1	0.111	0.019	0.002	0.022	1.46x10^{-6}	0.21 x10^{-2}	-6.17
49	1	0.111	0.012	0.001	0.013	8.82x10^{-7}	0.13x10^{-6}	-6.68

Table S3: Calculations of concentrations *vs* time for **2j** from ^{1}H NMR spectra

Time (h)	Integral of standard @ 8.44 ppm	Stoic. of standard (3H)	Integral @ 7.70 ppm	Stoic. of analyte (8H)	Ratio analyte :standard	Moles of analyte (mol)	Conc. of analyte (mol dm^{-3})	ln(conc)
3	1	0.333	1.32	0.165	0.495	3.76x10^{-6}	5.37x10^{-3}	-5.23
7	1	0.333	1.29	0.161	0.484	3.67 x10^{-6}	5.24 x10^{-3}	-5.25
12	1	0.333	1.20	0.150	0.450	3.42 x10^{-6}	4.88 x10^{-3}	-5.32
29	1	0.333	1.07	0.134	0.401	3.05 x10^{-6}	4.35 x10^{-3}	-5.44
49	1	0.333	0.49	0.061	0.184	1.39 x10^{-6}	1.99 x10^{-3}	-6.22

Table S4: Calculations of concentrations *vs* time for **2j** from ^{19}F NMR spectra

Time (h)	Integral of standard @ 63.4 ppm	Stoic. of standard (9F)	Integral @ 63.8 ppm	Stoic. of analyte (48F)	Ratio analyte :standard	Moles of analyte (mol)	Conc. of analyte (mol dm^{-3})	ln(conc)
3	1	0.111	2.37	4.94x10^{-2}	0.444	3.34x10^{-6}	4.77x10^{-3}	-5.35
7	1	0.111	2.02	4.21x10^{-2}	0.379	2.85x10^{-6}	4.07x10^{-3}	-5.51
12	1	0.111	1.72	3.58x10^{-2}	0.323	2.42x10^{-6}	3.46x10^{-3}	-5.67
29	1	0.111	1.47	3.06x10^{-2}	0.276	2.07x10^{-6}	2.96x10^{-3}	-5.82
49	1	0.111	1.30	2.71x10^{-2}	0.244	1.83x10^{-6}	2.62x10^{-3}	-5.95

Table S5: Calculations of concentrations *vs* time for **3c** from ^{1}H NMR spectra

Time (h)	Integral of standard @ 8.44 ppm	Stoic. of standard (3H)	Integral @ 6.35 ppm	Stoic. of analyte (4H)	Ratio analyte :standard	Moles of analyte (mol)	Conc. of analyte (mol dm^{-3})	ln(conc)
1	1	0.33	0.38	0.190	0.570	4.28x10^{-6}	6.12x10^{-3}	-5.10
2.5	1	0.33	0.37	0.185	0.555	4.17x10^{-6}	5.96x10^{-3}	-5.12
5	1	0.33	0.33	0.165	0.495	3.71x10^{-6}	5.31x10^{-3}	-5.24
9	1	0.33	0.31	0.155	0.465	3.49x10^{-6}	4.99x10^{-3}	-5.30
26	1	0.33	0.21	0.105	0.315	2.37x10^{-6}	3.38x10^{-3}	-5.69
30	1	0.33	0.22	0.110	0.330	2.48x10^{-6}	3.54x10^{-3}	-5.64
50	1	0.33	0.14	0.070	0.210	1.58x10^{-6}	2.25x10^{-3}	-6.10
120	1	0.33	0.06	0.030	0.090	6.76x10^{-7}	9.66x10^{-4}	-6.94

Table S6: Calculations of concentrations *vs* time for **3c** from ^{19}F NMR spectra

Time (h)	Integral of standard @ 63.4 ppm	Stoic. of standard (3H)	Integral @ 107.8 ppm	Stoic. of analyte (4H)	Ratio analyte :standard	Moles of analyte (mol)	Conc. of analyte (mol dm^{-3})	ln(conc)
1	1	0.111	0.36	0.045	0.405	2.43x10^{-5}	3.48x10^{-2}	-3.39
2.5	1	0.111	0.3	0.038	0.338	2.03x10^{-5}	2.90x10^{-2}	-3.54
5	1	0.111	0.33	0.041	0.371	2.23x10^{-5}	3.19x10^{-2}	-3.45
9	1	0.111	0.28	0.035	0.315	1.89x10^{-5}	2.70x10^{-2}	-3.61
26	1	0.111	0.22	0.028	0.248	1.49x10^{-5}	2.13x10^{-2}	-3.85
30	1	0.111	0.22	0.028	0.248	1.49x10^{-5}	2.13x10^{-2}	-3.85
50	1	0.111	0.15	0.019	0.169	1.01x10^{-5}	1.45x10^{-2}	-4.23
120	1	0.111	0.03	0.004	0.034	2.03x10^{-6}	0.29x10^{-2}	-5.84

Table S7: Calculations of concentrations *vs* time for $Mo_2(DAniF)_3(OAc)$ from ^1H NMR spectra [NB concentration of internal standard = 7.51x10^{-5} mol dm^{-6}]

Time (h)	Integral of standard @ 8.44 ppm	Stoic. of standard	Integral @ 6.33 ppm	Stoic. of analyte	Ratio analyte :standard	Moles of analyte (mol)	Conc. of analyte (mol dm^{-3})	ln(conc)
0.37	1	0.333	0.24	0.06	0.18	1.35x10^{-5}	1.93x10^{-2}	-1.71
0.57	1	0.333	0.23	0.0575	0.1725	1.30x10^{-5}	1.85x10^{-2}	-1.76
1.82	1	0.333	0.15	0.0375	0.1125	8.45x10^{-6}	1.21x10^{-2}	-2.18
3.22	1	0.333	0.1	0.025	0.075	5.64x10^{-6}	8.05x10^{-3}	-2.59
4	1	0.333	0.04	0.01	0.03	2.25x10^{-6}	3.22x10^{-3}	-3.51
11.9	1	0.333	0.00	0.00	0.00	0.00	0.00	-18.71

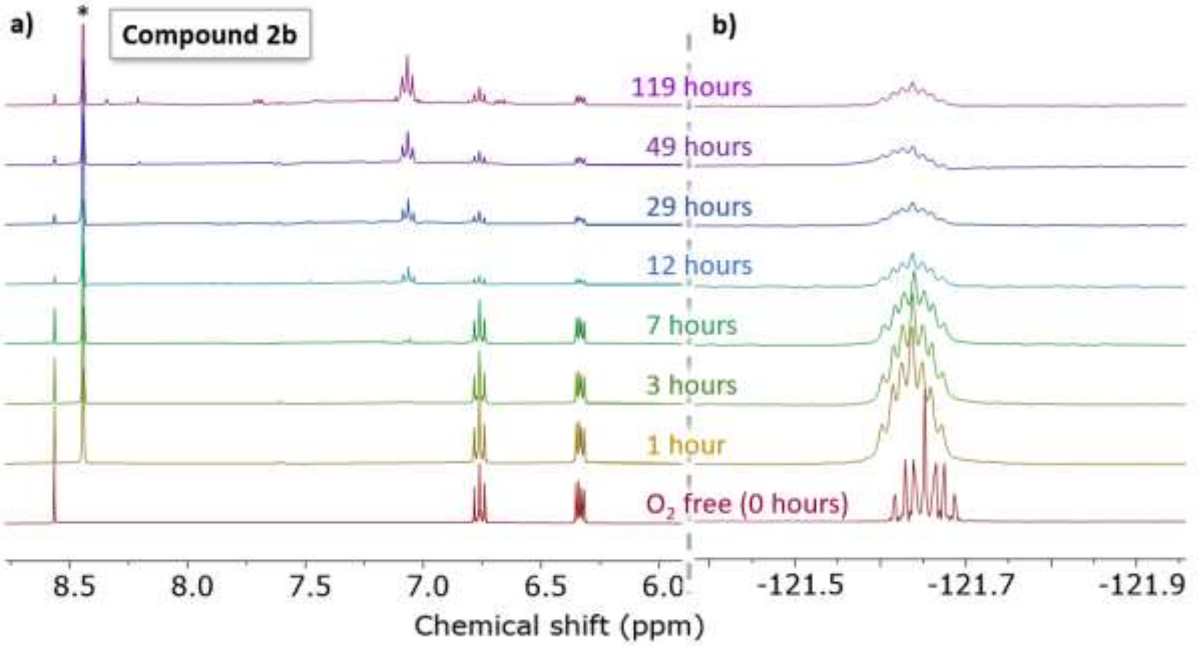

Figure S107: (a) ^1H and (b) ^{19}F NMR spectra of compound **2b** in "oxygenated" $CDCl_3$ *marks internal standard 1,3,5-tris(trifluoromethyl)benzene (0.852 mol dm^{-3})

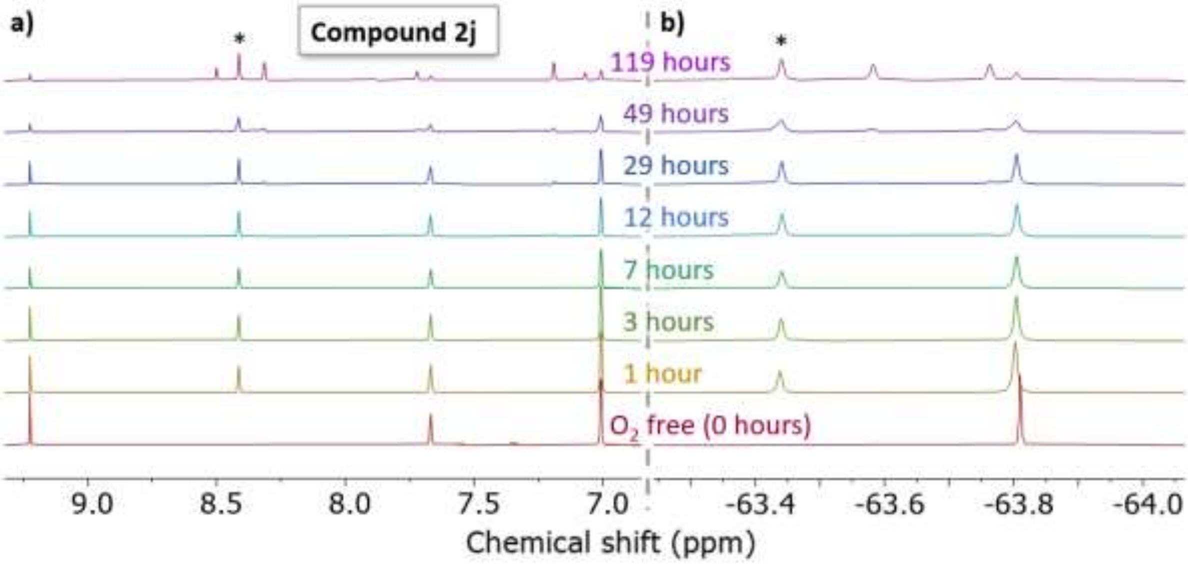

Figure S108: (a) ^{1}H and (b) ^{19}F NMR spectra of compound **2j** in “oxygenated” $CDCl_3$ *marks internal standard 1,3,5-tris(trifluoromethyl)benzene (0.852 mol dm^{-3})

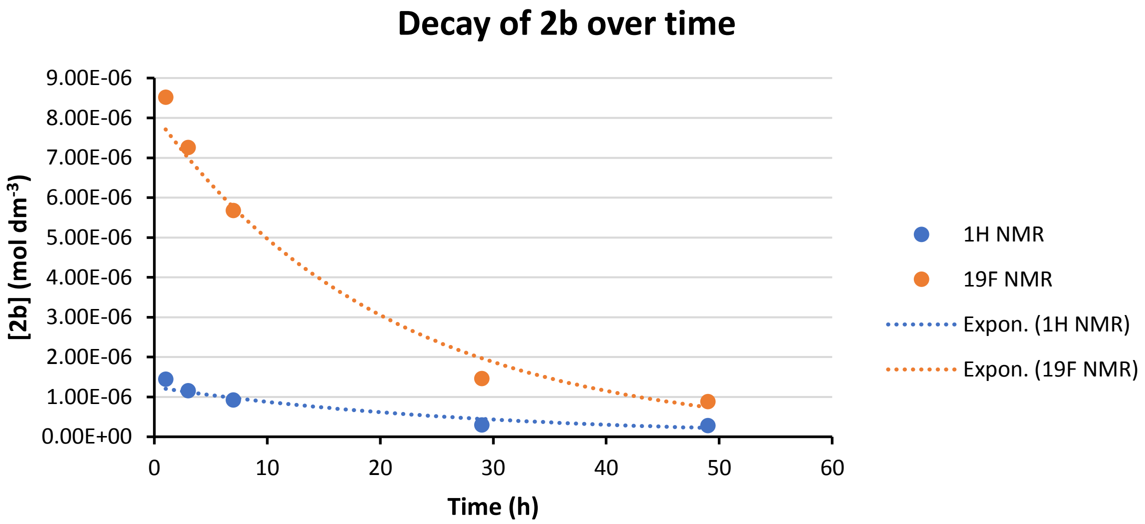

Figure S109: Graph to show the decay of **2b** over time. Orange trace taken from ^{19}F NMR spectra, blue trace taken from ^{1}H NMR spectra. The discrepancy between the two traces can be attributed variation in the ^{19}F NMR baseline due to the presence of PTFE in the probe.

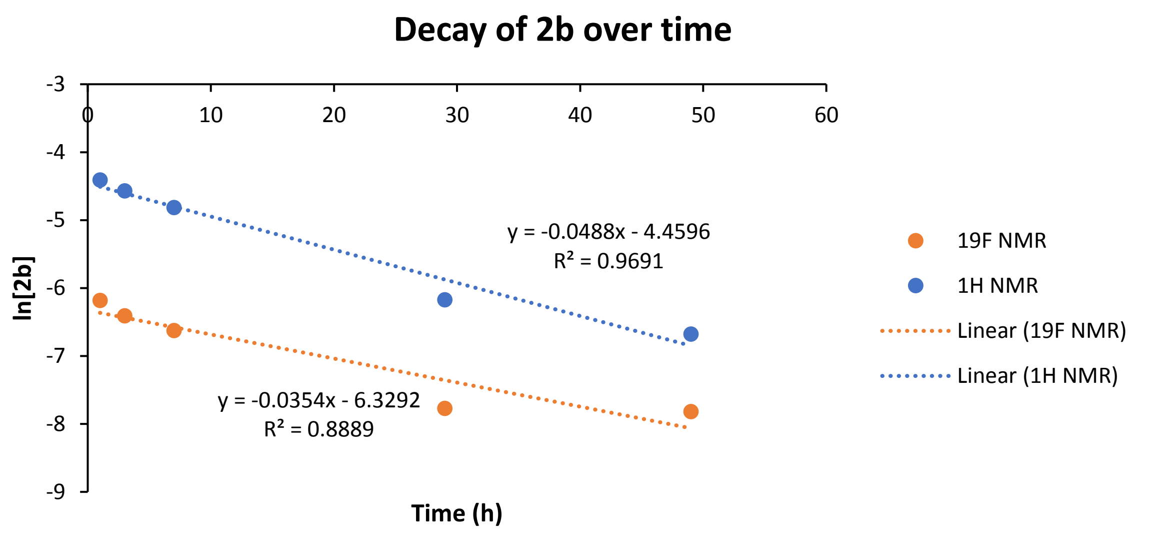

Figure S110: Graph to show the decay of **2b** over time. Orange trace taken from ^{19}F NMR spectra, blue trace taken from ^{1}H NMR spectra

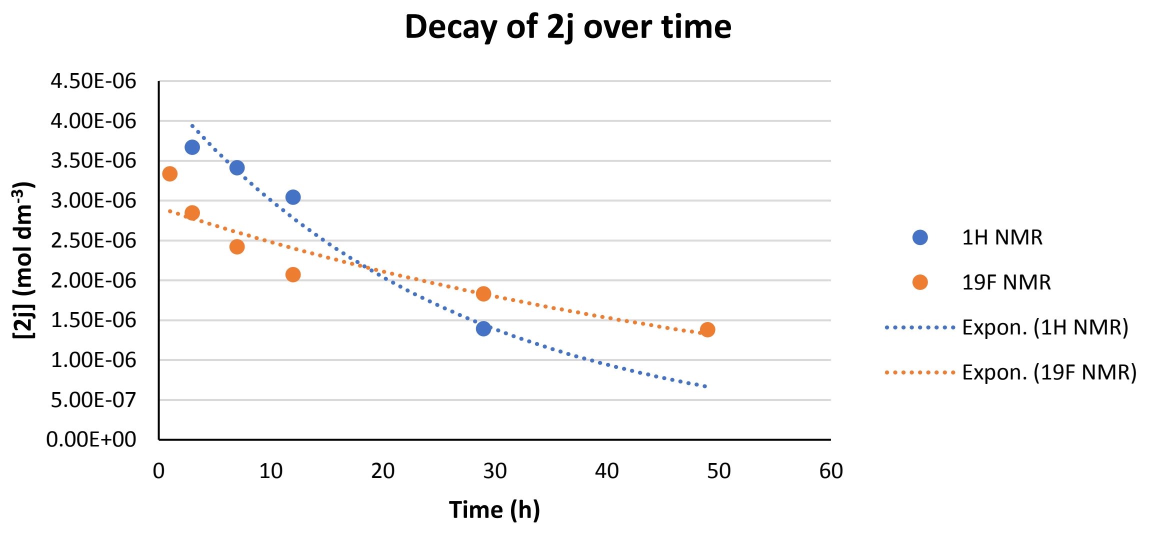

Figure S111: Graph to show the decay of **2j** over time. Orange trace taken from ^{19}F NMR spectra, blue trace taken from ^{1}H NMR spectra

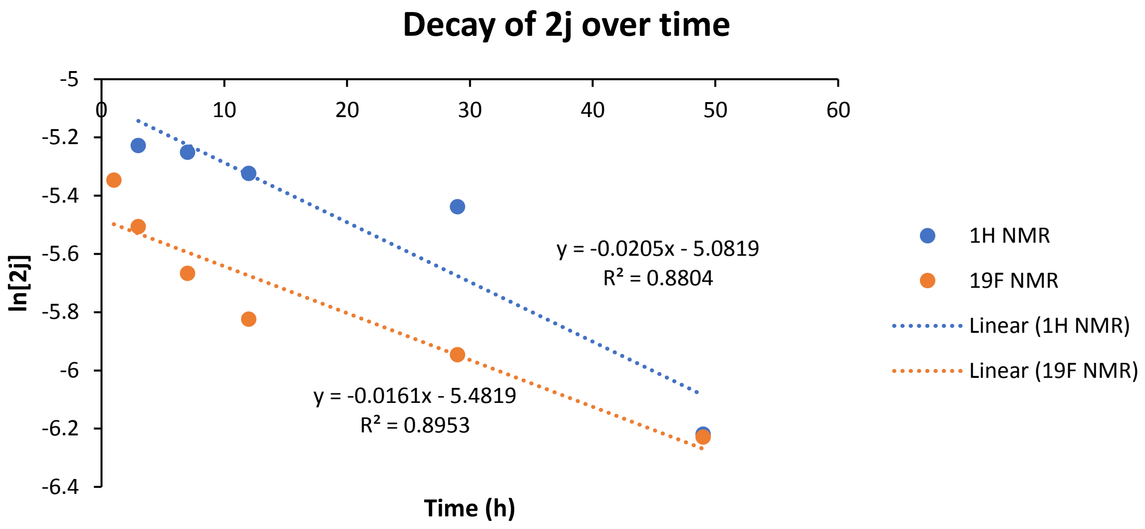

Figure S112: Graph to show the decay of **2j** over time. Orange trace taken from ^{19}F NMR spectra, blue trace taken from ^{1}H NMR spectra

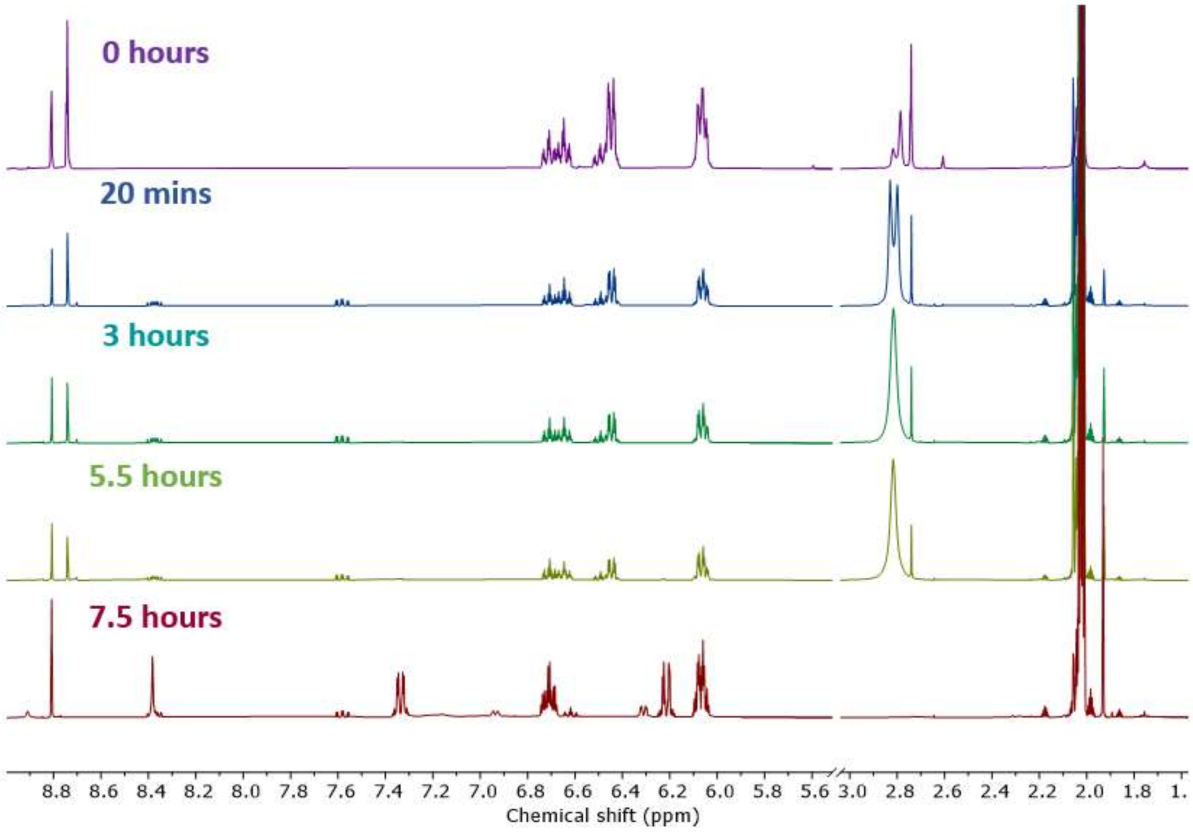

Figure S113: ^{1}H NMR spectra of compound **3c** in "oxygenated" Acetone-d_6

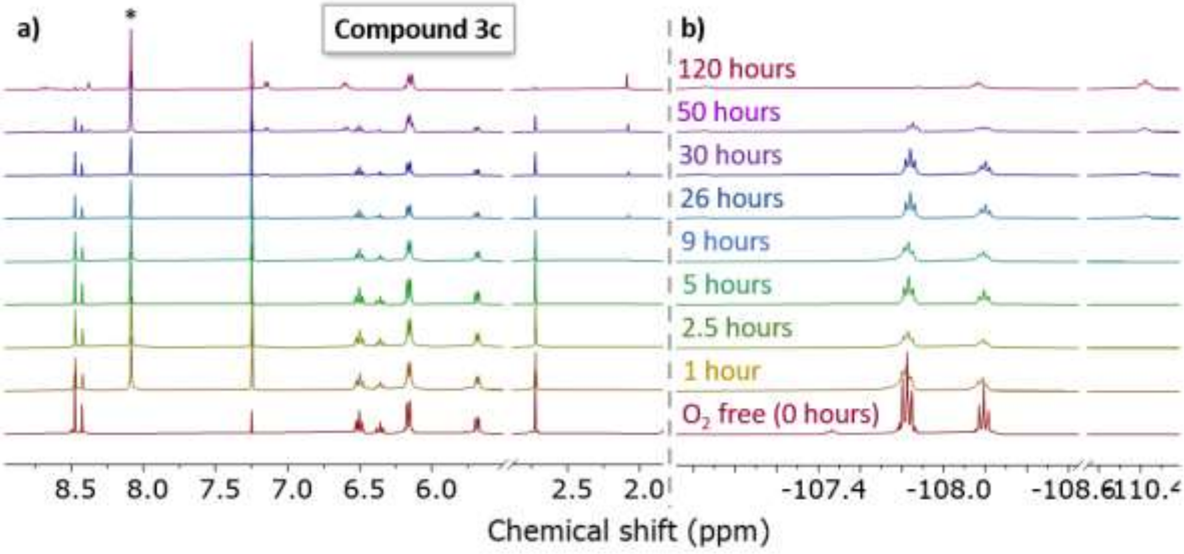

Figure S114: a) ^{1}H and b) ^{19}F NMR spectra of compound **3c** in "oxygenated" $CDCl_3$

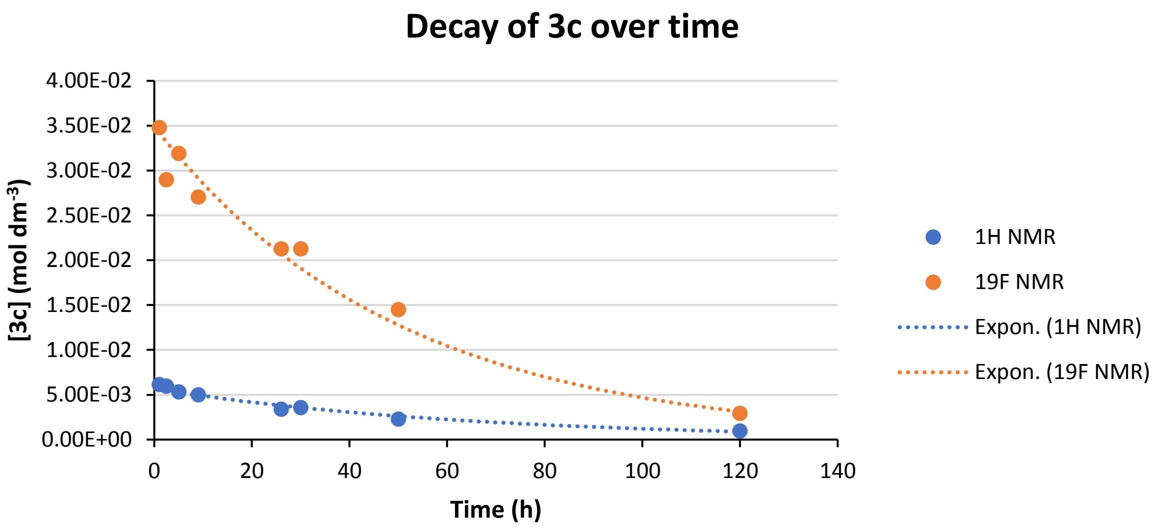

Figure S115: Graph to show the decay of **3c** over time. Orange trace taken from ^{19}F NMR spectra, blue trace taken from ^{1}H NMR spectra. The discrepancy between the two traces can be attributed variation in the ^{19}F NMR baseline due to the presence of PTFE in the probe.

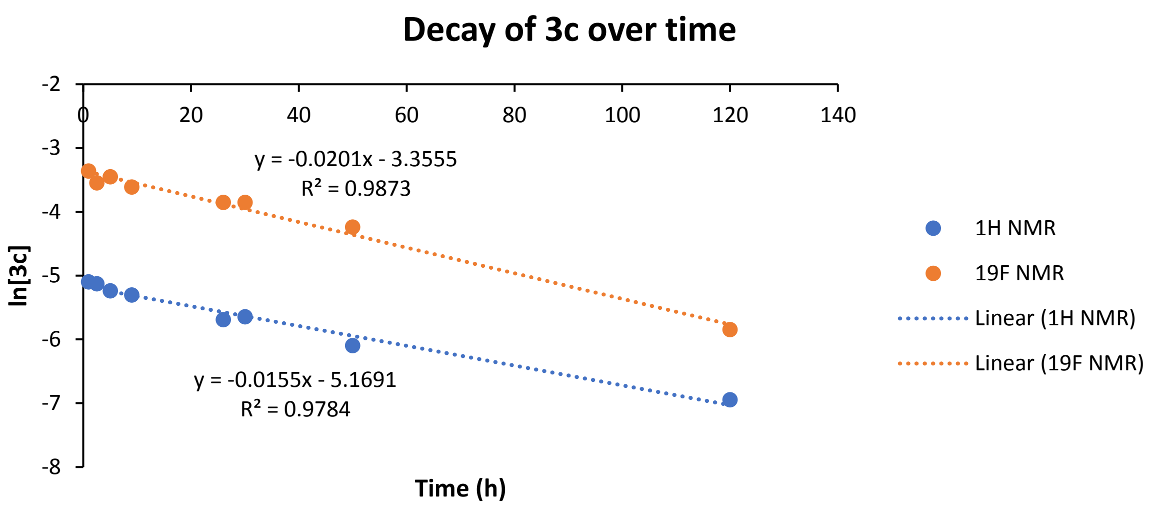

Figure S116: Graph to show the decay of **3c** over time. Orange trace taken from ^{19}F NMR spectra, blue trace taken from ^{1}H NMR spectra

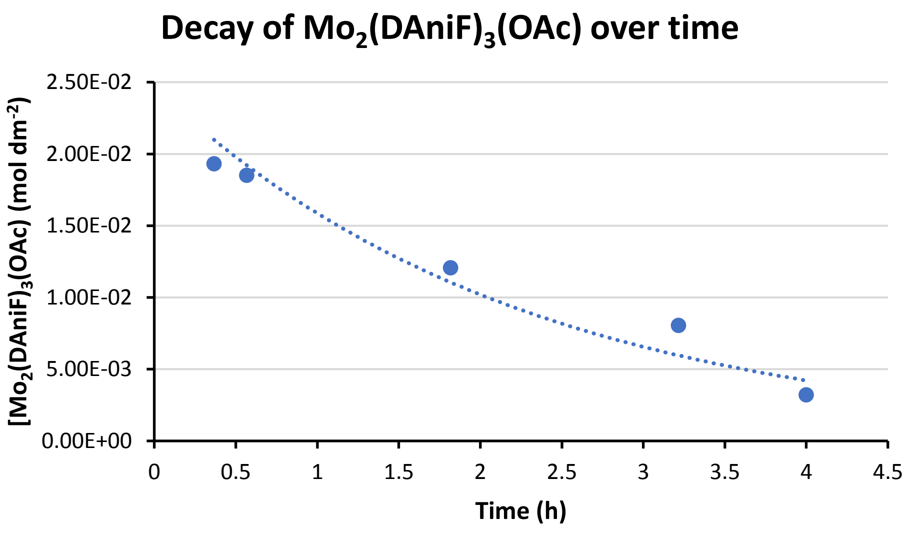

Figure S117: Graph to show the decay of **$Mo_2(DAniF)_3OAc$** over time taken from ^{1}H NMR spectra

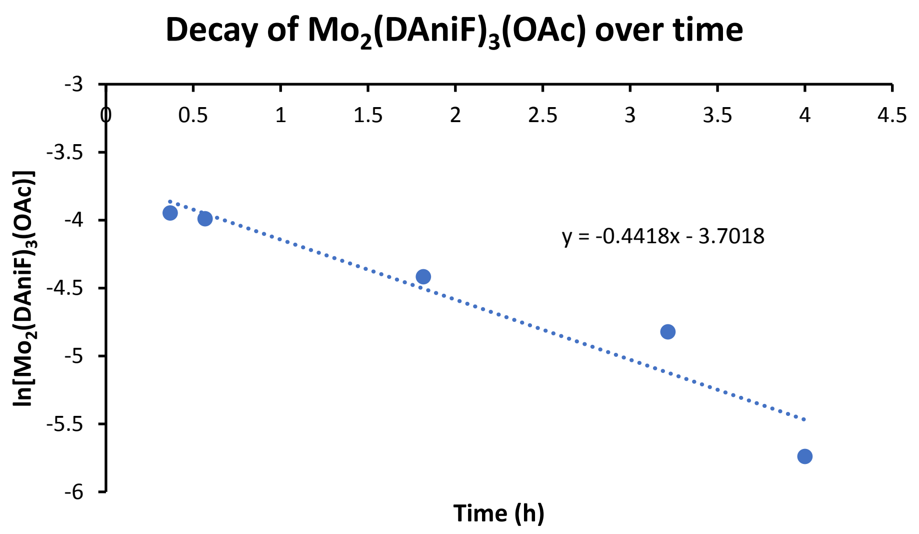

Figure S118: Graph to show the decay of **$Mo_2(DAniF)_3OAc$** over time taken from ^{1}H NMR spectra

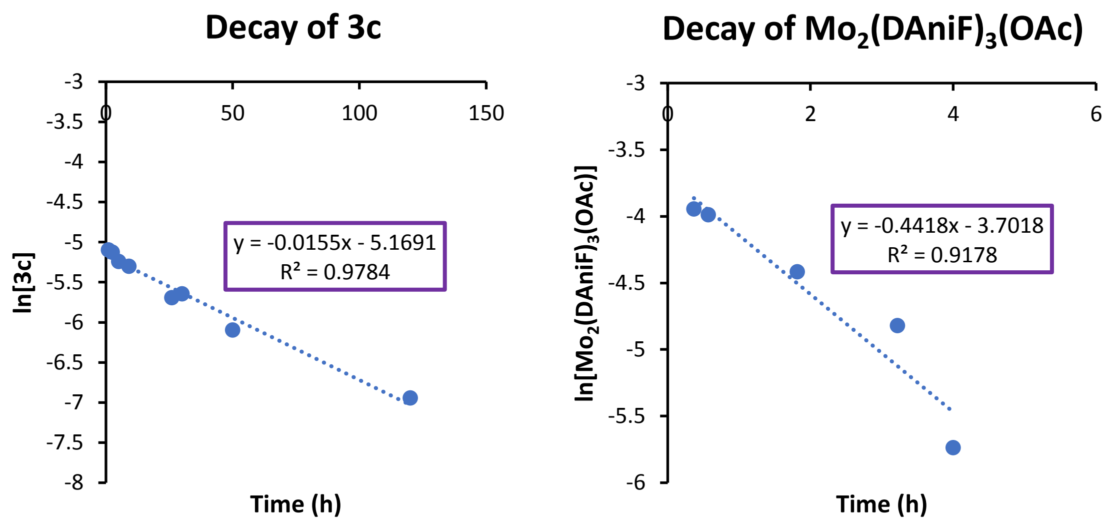

Figure S119: A comparison of the decay of **$Mo_2(DAniF)_3OAc$** *vs*. **3c** over time.

Solid-state decomposition of 3c compared to $Mo_2(DAniF)_3OAc$.

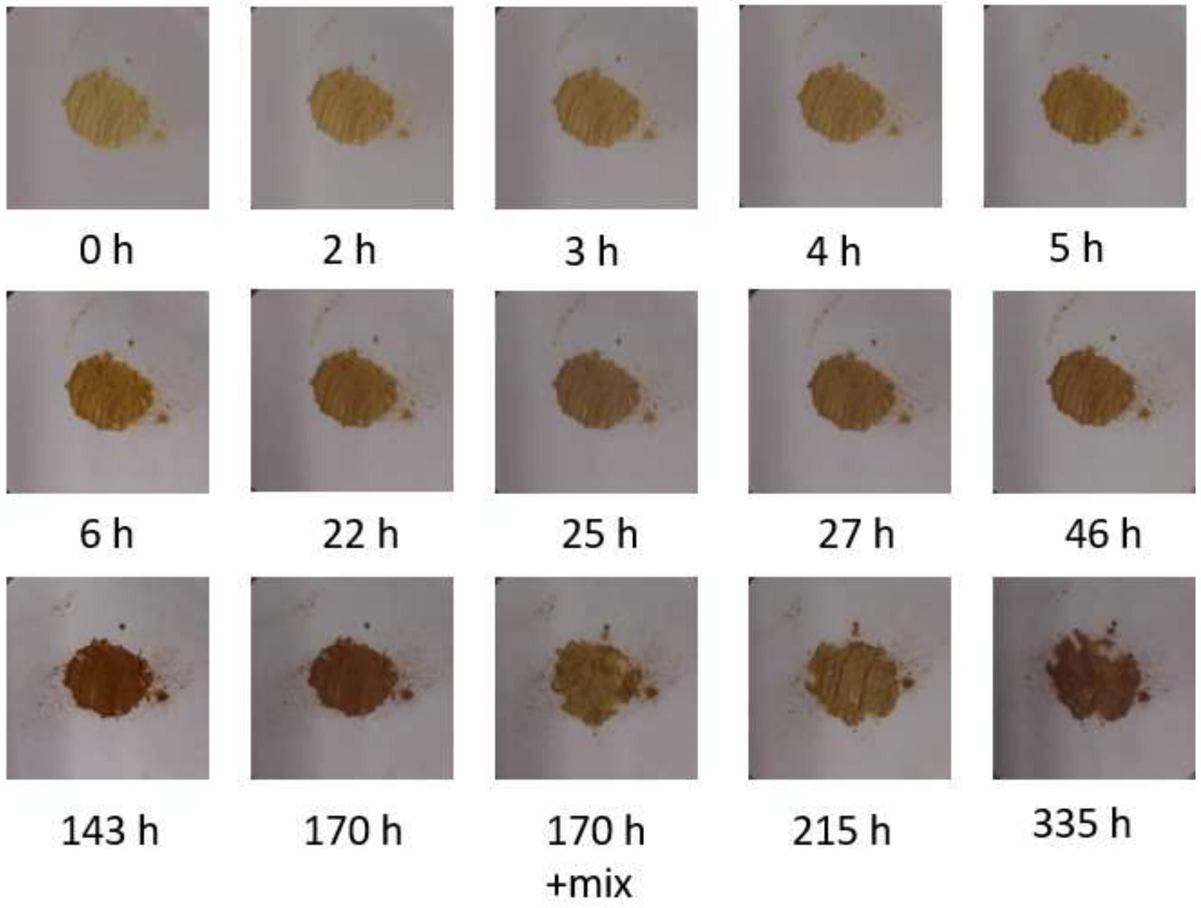

Figure S120: A visual representation of the decomposition of solid **3c** in air over time. After 170 h the solid sample was agitated (170 h +mix) to uncover "un-oxidized" material below the surface

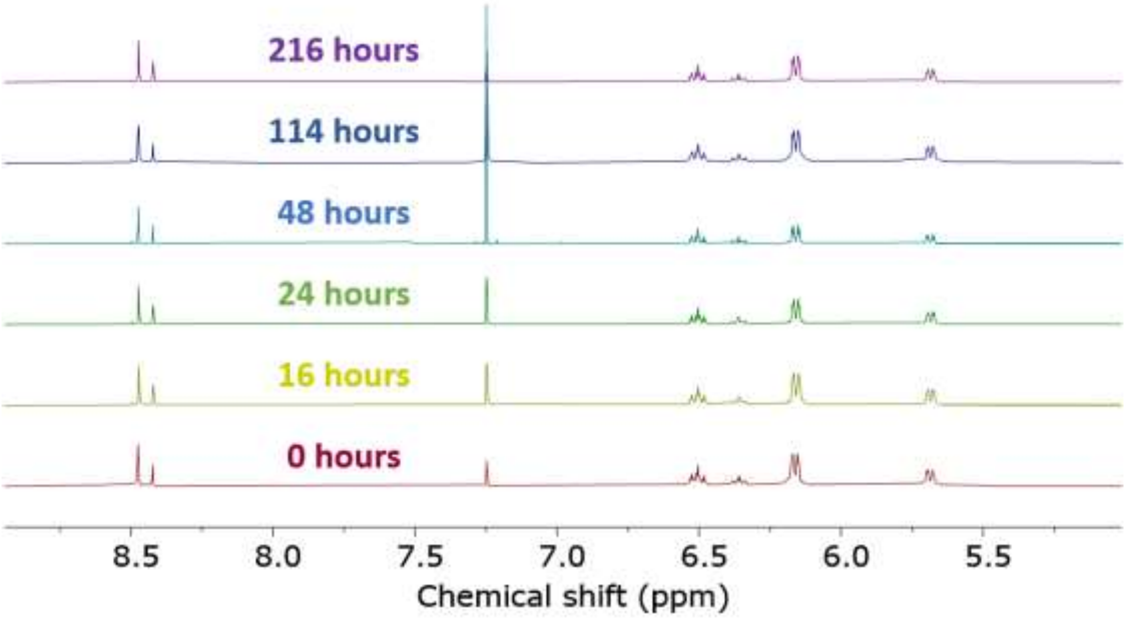

Figure S121: ^{1}H NMR spectra of **3c** in deoxygenated $CDCl_3$ after the solid had been exposed to air for the corresponding number of hours.

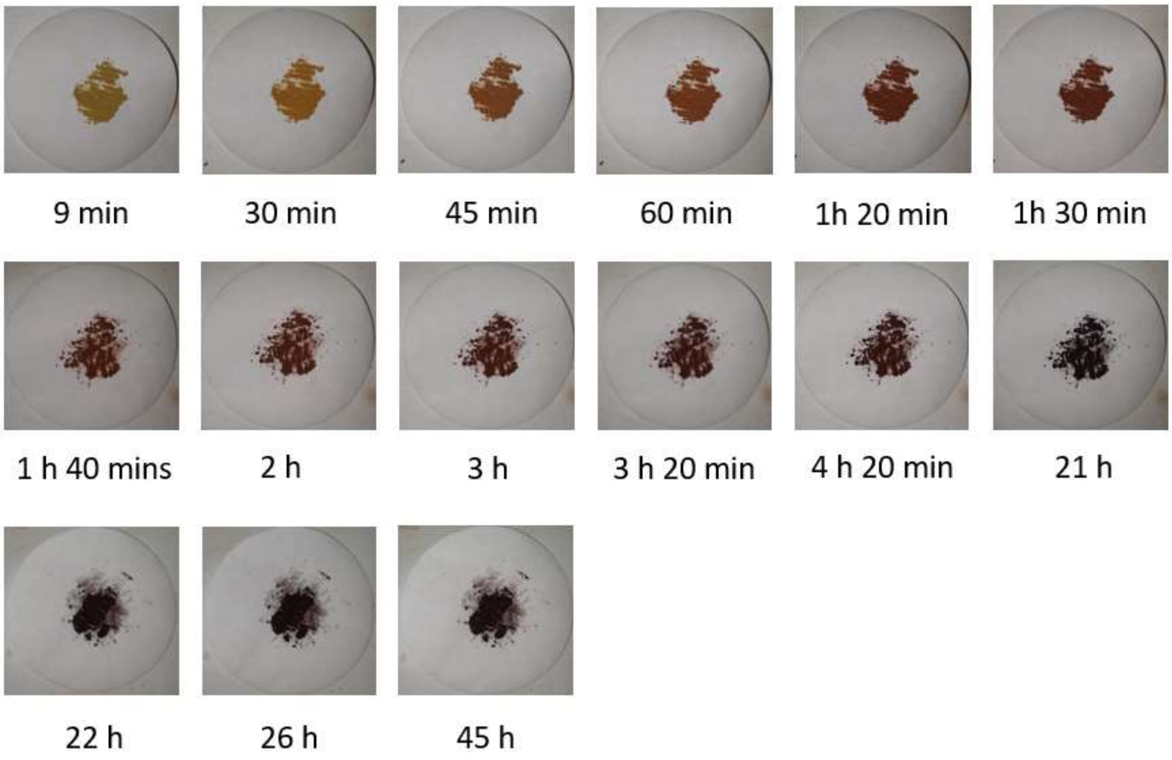

Figure S122: A visual representation of the decomposition of solid **Mo_2(DAniF)$_3$OAc** in air over time. A small portion of the sample was removed for NMR spectra at 1 h 40 mins and again at 22 h and 45h.

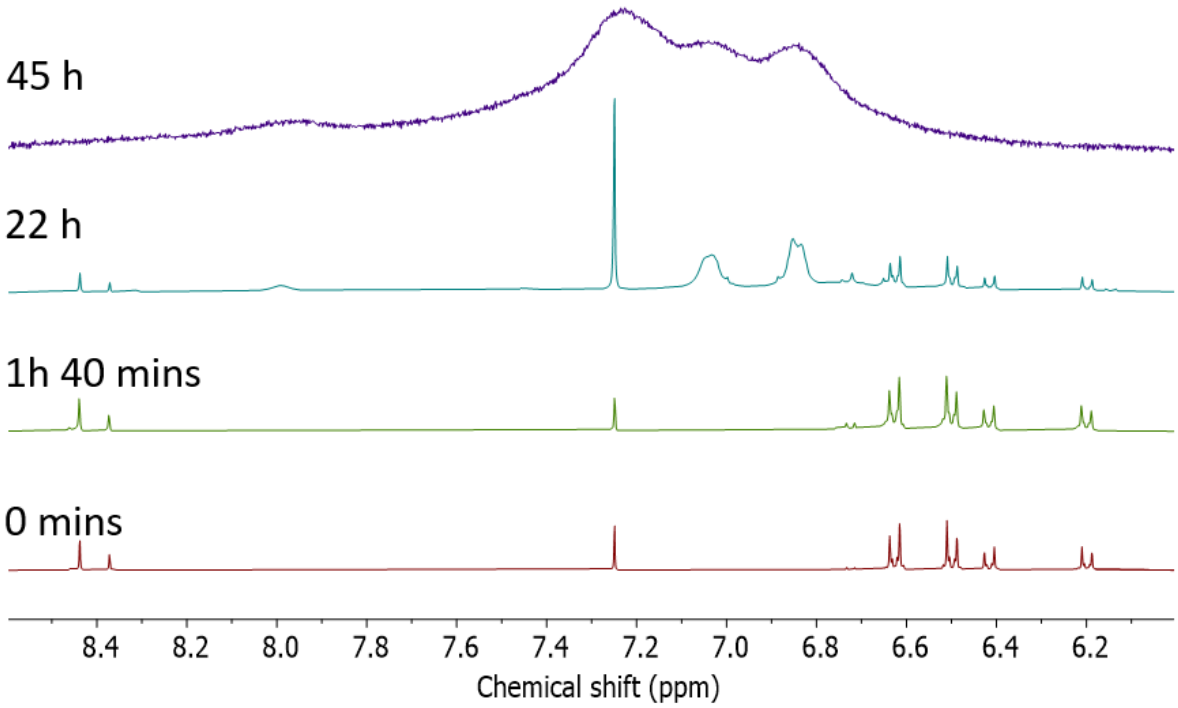

Figure S123: ^{1}H NMR spectra of Mo_2(DAniF)$_3$OAc in deoxygenated $CDCl_3$ after the solid had been exposed to air.

References

1) CrysAlisPro, Oxford Diffraction Ltd. Version 1.171.40.84
2) Empirical absorption correction using spherical harmonics, implemented in SCALE3 ABSPACK scaling algorithm within CrysAlisPro software, Oxford Diffraction Ltd. Version 1.171.34.40
3) Dolomanov, O.V., Bourhis, L.J., Gildea, R.J, Howard, J.A.K. & Puschmann, H. (2009), J. Appl. Cryst. 42, 339-341
4) SHELXT – Integrated space-group and crystal-structure determination G. M. Sheldrick, Acta Cryst. 2015, A71, 3-8
5) “Crystal structure refinement with SHELXL” G.M. Sheldrick, Acta Cryst. 2015, C71, 3-8.
6) Gaussian 16, Revision A.03, M. J. Frisch, G. W. Trucks, H. B. Schlegel, G. E. Scuseria, M. A. Robb, J. R. Cheeseman, G. Scalmani, V. Barone, G. A. Petersson, H. Nakatsuji, X. Li, M. Caricato, A. V. Marenich, J. Bloino, B. G. Janesko, R. Gomperts, B. Mennucci, H. P. Hratchian, J. V. Ortiz, A. F. Izmaylov, J. L. Sonnenberg, D. Williams-Young, F. Ding, F. Lipparini, F. Egidi, J. Goings, B. Peng, A. Petrone, T. Henderson, D. Ranasinghe, V. G. Zakrzewski, J. Gao, N. Rega, G. Zheng, W. Liang, M. Hada, M. Ehara, K. Toyota, R. Fukuda, J. Hasegawa, M. Ishida, T. Nakajima, Y. Honda, O. Kitao, H. Nakai, T. Vreven, K. Throssell, J. A. Montgomery, Jr., J. E. Peralta, F. Ogliaro, M. J. Bearpark, J. J. Heyd, E. N. Brothers, K. N. Kudin, V. N. Staroverov, T. A. Keith, R. Kobayashi, J. Normand, K. Raghavachari, A. P. Rendell, J. C. Burant, S. S. Iyengar, J. Tomasi, M. Cossi, J. M. Millam, M. Klene, C. Adamo, R. Cammi, J. W. Ochterski, R. L. Martin, K. Morokuma, O. Farkas, J. B. Foresman, and D. J. Fox, Gaussian, Inc., Wallingford CT, 2016.
7) ***NBO 7.0.*** E. D. Glendening, J, K. Badenhoop, A. E. Reed, J. E. Carpenter, J. A. Bohmann, C. M. Morales, P. Karafiloglou, C. R. Landis, and F. Weinhold, Theoretical Chemistry Institute, University of Wisconsin, Madison (2018).
